# Promising Anticancer Activity of Novel Hydroxyethyloxy and Bromo derivatives of Curcumin and Its Complexes with BF_2_

**DOI:** 10.3390/molecules30234609

**Published:** 2025-11-30

**Authors:** Eduard Potapskyi, Dawid Łażewski, Julian Myszkiewicz, Gabriela Korzańska, Joanna Kuźmińska, Łukasz Popenda, Artur Korzański, Agnieszka Zgoła-Grześkowiak, Agnieszka Gielara-Korzańska, Karolina Chmaj-Wierzchowska, Nataliya Finiuk, Yuliia Kozak, Iryna Ivasechko, Rostyslav Stoika, Roman Lesyk, Marcin Wierzchowski

**Affiliations:** 1 Department of Chemical Technology of Drugs, Poznan University of Medical Sciences, Rokietnicka Street 3, 60-806 Poznan, Poland; potapskiyed@gmail.com (E.P.);; 2 Doctoral School, Poznan University of Medical Sciences, Bukowska Street 70, 60-812 Poznan, Poland; 3 Department of Pharmaceutical Chemistry, Poznan University of Medical Sciences, Rokietnicka Street 3, 60-806 Poznan, Poland; 4 NanoBioMedical Centre, Adam Mickiewicz University, Wszechnicy Piastowskiej Street 3, 61-614 Poznan, Poland; 5 Department of Chemistry, Adam Mickiewicz University, Uniwersytetu Poznanskiego Street 8, 61-614 Poznan, Poland; 6 Institute of Chemistry and Technical Electrochemistry, Poznan University of Technology, Berdychowo Street 4, 60-965 Poznan, Poland; 7 Department of Maternal and Child Health, Poznan University of Medical Sciences, 60-701 Poznan, Poland; 8 Department of Regulation of Cell Proliferation and Apoptosis, Institute of Cell Biology of the National Academy of Sciences of Ukraine, Drahomanov Street 14/16, 79005 Lviv, Ukraine; nataliyafiniuk@gmail.com (N.F.);; 9 Molecular Design Center, State Non-Commercial Enterprise, Danylo Halytsky Lviv National Medical University, Pekarska Street 69, 79010 Lviv, Ukraine; roman.lesyk@gmail.com; 10 Department of Pharmaceutical, Organic and Bioorganic Chemistry, Danylo Halytsky Lviv National Medical University, Pekarska Street 69, 79010 Lviv, Ukraine; 11 Department of Biotechnology and Cell Biology, Medical College, University of Information Technology and Management in Rzeszow, Sucharskiego 2, 35-225 Rzeszow, Poland

**Keywords:** curcumin, curcumin derivatives, bromine, anticancer activity

## Abstract

Curcumin has long been used for health purposes and is currently attracting significant research interest. In this study, we present a series of curcumin derivatives featuring structural modifications, including methoxy groups, short alcohol chains, and bromine atoms. The cytotoxic activity of the compounds obtained was tested against BA/F3 wt, BA/F3 del52, BA/F3 ins5, K562, Jurkat, HCT-116, and MDA-MB-231 cell lines and non-cancerous Balb/3T3 fibroblast lines. The most promising compounds **2a**, **6a**, and **9a** demonstrated anticancer activity comparable to that of doxorubicin, while exhibiting toxicity toward fibroblasts similar to natural curcumin. In addition, thanks to microscopic fluorescence analysis, a mechanism of action was proposed for the most active compounds against the HCT-116 cell line. Some compounds exhibit moderate or strong proapoptotic activity, while others are characterized by cytostatic activity. Studied compounds demonstrated the DNA-intercalation ability and increased the content of cellular ROS in treated HCT-116 cells.

## 1. Introduction

**Figure 1:**
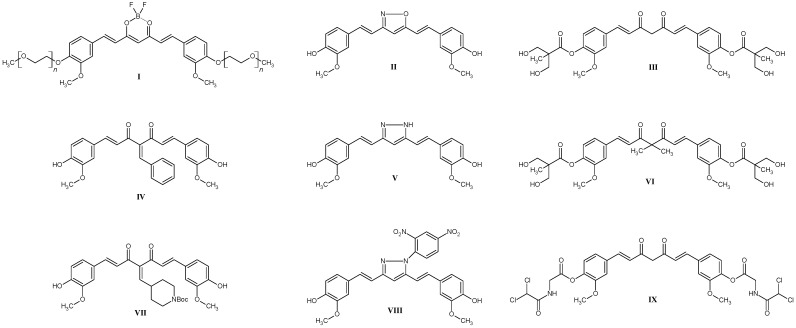
The most interesting and promising curcumin derivatives being the object of interest of scientists from the literature review.

Curcumin derivatives are the subject of intense research in the context of cancer therapy due to their enhanced biological activity and more favorable pharmacokinetic properties relative to natural curcumin (Figure 1) [1,2]. Numerous chemical modifications—such as PEGylation, encapsulation in nanocarrier structures, and isotope labeling—have been shown to enhance water solubility, chemical stability, and bioavailability, resulting in markedly more potent anticancer activity [3,4,5,6]. In colorectal cancer studies, it has been demonstrated that select curcumin derivatives can inhibit the activity of the NF-κB signaling pathway and induce apoptosis through caspase activation. At the same time, a reduction in tumor cell proliferation and modulation of the expression of pro- and anti-apoptotic proteins such as Bax and Bcl-2 have been observed [2,3,7]. In breast cancer models, it has been noted that curcumin derivatives can modulate the activity of key signaling pathways involved in tumor development and progression, including the PI3K/Akt/mTOR, MAPK, Wnt/β-catenin, and JAK/STAT pathways. Effects on these molecular mechanisms have been linked to reducing the proliferation, migration, and invasive capacity of cancer cells, as well as potentially overcoming drug resistance (Structures **III**, **VI**, **IX**, Figure 1) [1]. Curcumin derivatives have shown therapeutic potential in the treatment of prostate cancer, primarily through modulation of key signaling pathways linked to factors such as NF-κB, EGFR, and MAPK, leading to the induction of apoptosis and inhibition of cancer cell proliferation (Structures **IV**, **VII**, Figure 1) [2,7]. In esophageal cancer, curcumin derivatives have been demonstrated to inhibit tumor cell proliferation, lead to cell cycle arrest, and induce apoptosis. The mechanism of these actions involves, among other things, modulation of the expression of proteins such as p53, Bax, and Bcl-2, and inhibition of the activity of the transcription factor NF-κB, which plays a key role in inflammatory reactions and cancer progression [8]. In liver tumors, curcumin derivatives exhibit anti-inflammatory effects by inhibiting the expression of cytokines, such as TNF-α and IL-6, as well as the activity of key signaling kinases (MAPK, JAK/STAT), leading to suppression of processes that contribute to carcinogenesis. Additionally, these compounds inhibit the process of epithelial–mesenchymal transition (EMT), which translates into reduced invasiveness and migration of tumor cells [4]. It is also worth noting the importance of radioisotope-labeled derivatives, which allow imaging of tumors and delivery of therapeutic compounds directly to the affected tissues [5]. PEGylated curcumin derivatives (Structure **I**, Figure 1) exhibit enhanced cytotoxicity against tumor cells, due to both the induction of oxidative stress and the modulation of signaling pathways involved in cell cycle regulation and cellular stress response mechanisms [6]. Another mechanism of anticancer activity is oxidative DNA breakage involving copper ions and simultaneous ROS generation. Additionally, curcumin and its derivatives can also inhibit copper transporters, which are essential for cell survival. Moreover, targeting NF-κB and DYRK2 factors in synergy with ROS ROS-driven pathway suppresses tumor cell proliferation [9]. Importantly, some curcumin derivatives act synergistically with cytostatic agents such as cisplatin—not only enhancing the anticancer effect, but also reducing toxicity to healthy tissues [10]. The diketone moiety, which is a central part of the curcumin molecule, can be modified easily in many different ways. Most commonly, it is changed into either a pyrazole or an oxazole ring (Structures **II**, **V**, and **VIII**, Figure 1) by employing various hydrazine or hydroxylamine derivatives [1,11]. The modification we have decided to utilize—the introduction of the BF_2_ moiety is steadily gaining more attention. It appeared first as a reaction steering group to increase the yield of curcumin synthesis when compared to the Pabon method [12]. Soon after, however, it turned out that its presence can have a beneficial effect on the anticancer activity of curcuminoid [13,14]. While it is unknown if the presence of the BF_2_ moiety changes any mechanism or target, the presence of fluorine is important. Kim et al. have shown that substituting fluorine, for example, with phenyl rings does not change the antiproliferative activity when compared to curcumin [15]. A common modification employed when searching for new biologically active molecules is halogenation [16]. Most of the halogenated curcumins and curcuminoid derivatives incorporate fluorine in their structure, leaving other halogens underrepresented [1,17,18,19]. Thus, in this work, we have focused on testing whether bromine could offer any benefits. The broad spectrum of activity exhibited by curcumin derivatives—including their modulation of molecular pathways and demonstrated therapeutic potential across various cancer types—positions them as one of the most promising avenues in the development of contemporary oncological treatment strategies.

## 2. Results and Discussion

### 2.1. Synthesis

**Figure 2:**
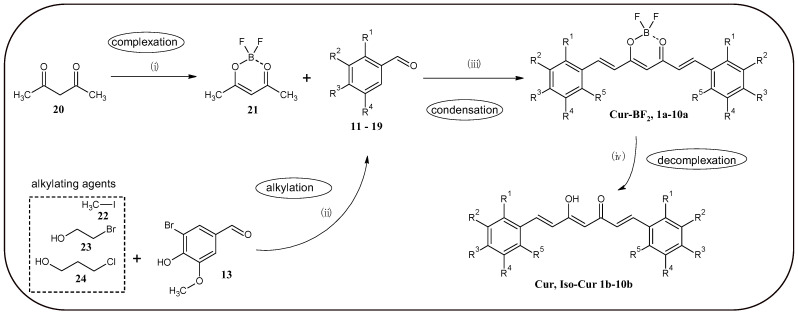
General synthesis plan of curcuminoids obtained in this study and precursors for their synthesis. (i) BF_3_·Et_2_O, DCM, 24 h, 20 °C; (ii) LiOH·H_2_O or K_2_CO_3_ DMF, 24h 80 °C; (iii) n-BuNH_2_, tributyl borate, toluene, 24 h, 65 °C; (iv) Na_2_C_2_O_4_, MeOH:H_2_O 4:1, MW, 8 min, 140 °C. The numbered substances: **11**—vanillin; **12**—isovanillin; **13**—5-bromovanillin; **14**—2-bromoisovanillin; **15**—3,4,5-trimethoxybenzaldehyde; **16**—syringaldehyde, **20**—acetylacetone, **21**—acetylacetone-BF_2_ complex, **22**—iodomethane, **23**—2-bromoethanol, **24**—3-chloropropanol. Abbreviations: DCM, dichloromethane; DMF, dimethylformamide; MW, microwave.

In this work, the method for obtaining curcuminoid complexes with BF_2_, which was developed by Liu et al. [12], was used (Figure 2). First, the corresponding aromatic aldehydes (**17**–**19**) were obtained by nucleophilic substitution reaction, using 5-bromovanillin and halogenated aliphatic compounds as substrates. Then, the previously obtained acetylacetone complex (**21**) with BF_2_ underwent an aldol condensation reaction to obtain curcuminoid complexes with BF_2_ **1a**–**10a**. The final step was a hydrolysis reaction in a microwave reactor to obtain curcuminoids with a free keto-enol moiety **1b**–**10b**. This reaction was developed by Abonia et al. [13]. Such a synthetic pathway makes it possible not only to increase the total yield of the process, but also to obtain an additional series of compounds with high anticancer potential [6,13,20]. Table 1 shows the position of substituents in final compounds and substrates.

### 2.2. NMR Study

**Figure 3:**
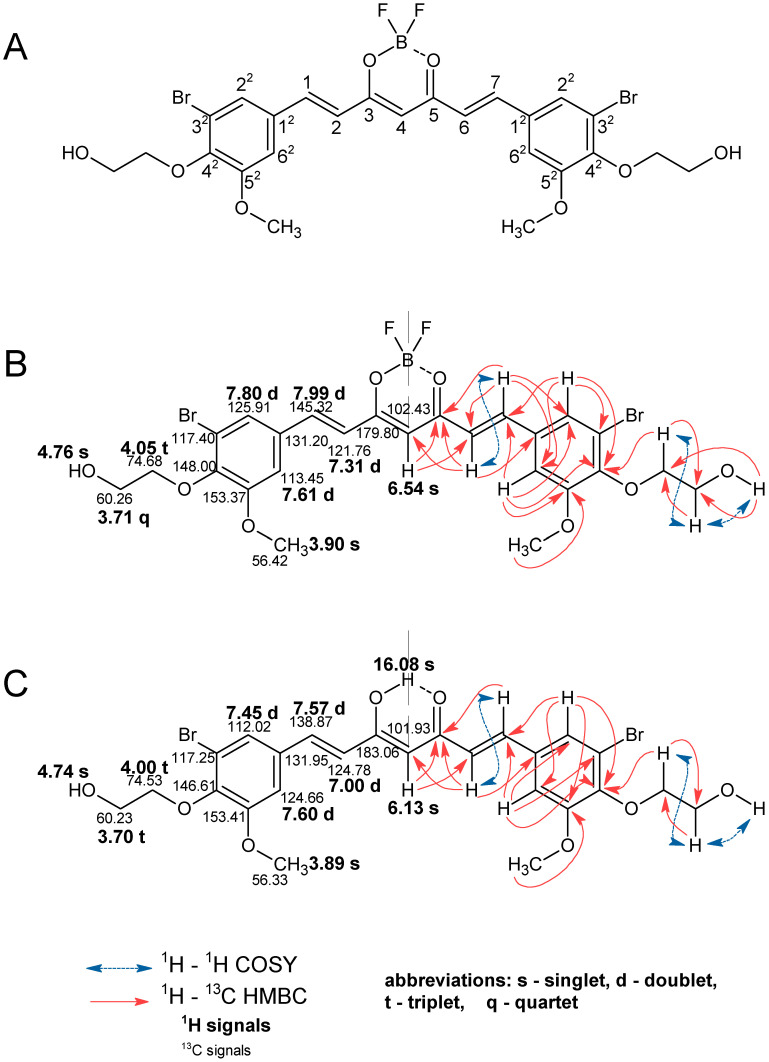
^1^H and ^13^C NMR data for compounds **5a** and **5b** in DMSO. (**A**) Numbering of atoms in the curcuminoid molecule. (**B**) Annotated ^1^H and ^13^C NMR signals for **5a**. (**C**) Annotated ^1^H and ^13^C NMR signals for compound **5b**.

The identity of the obtained compounds was confirmed by 1D ^1^H and ^13^C NMR techniques. To assign the observed signals in the 1D NMR experiments to the individual structural elements of the molecules, 2D correlation experiments (^1^H-^1^H COSY, ^1^H-^13^C HSQC, and ^1^H-^13^C HMBC) were also carried out, what is demonstrated in Figure 3 using the example of derivatives **5a** and **5b**.

As expected, NMR data confirmed that curcuminoids are present in the enol form. Based on the NMR spectra, the structure of compound **5a** was confirmed by observing a characteristic signal of the CH group at the C4 position singlet with integration of one proton at 6.54 ppm. For compound **5b**, the corresponding signal is observed at 6.13 ppm, and a broad signal (singlet) from the OH group is also visible at 16.08 ppm as a result of tautomerization. The presence of carbonyl groups at the C3 and C5 positions is confirmed by the signal in the ^13^C NMR spectrum at 179.80 ppm for **5a** and 183.06 ppm for **5b**. The presence of -HC=CH- vinylene groups was confirmed by the presence of signals from protons and carbons at positions 1, 2, 6, and 7. Signals for H1 and H7 are observed as doublets for compounds **5a** and **5b** at 7.99 ppm and 7.57 ppm, respectively. Similarly, doublets for protons H2 and H6 are also observed at 7.31 ppm for **5a** and 7.00 ppm for **5b**. In all the curcuminoids obtained, the vinylene protons appear in *trans* configuration, as confirmed by the value of the J_H,H_ constant at ca. 16 Hz. For compound **5a**, signals from C1 and C7 were observed at 145.32 ppm, and for **5b** at 138.87 ppm. Signals from C2 and C6 are observed at 121.76 ppm for **5a** and 124.78 ppm for **5b**. With these data, the presence of the BF_2_ group is confirmed, as shifts from 1H and 13C signals are observed at positions 1, 2, 6, and 7 of compound **5a** compared to **5b**. In aromatic rings, signals from protons are observed as two doublets. For compound **5a**, these are peaks at 7.80 ppm and 7.61 ppm for H6^2^ and H2^2^, respectively. For compound **5b**, these are signals at 7.45 ppm and 7.60 ppm. In the 13C NMR spectra, the signals for C12-C62 are represented by values of 131.20 ppm, 113.45 ppm, 153.37 ppm, 148.00 ppm, 117.40 ppm, and 125.91 ppm for compound **5a** and 131.95 ppm, 124.66 ppm, 153.41 ppm, 146.61 ppm, 117.25 ppm, and 112.02 ppm for compound **5b**. In the ^1^H NMR spectrum, signals from the methoxy group are represented by singlets at 3.90 ppm for compound **5a** and 3.89 ppm for compound **5b**. In the ^13^C NMR spectrum, these signals are at 56.42 ppm and 56.33 ppm for compounds **5a** and **5b**, respectively. For an alcohol chain linked by an ether group to an aromatic ring at the C4^2^ position, the characteristic signals in the ^1^H NMR spectrum are one singlet, one quartet, and one triplet for compound **5a**. A proton from the OH group is observed at 4.76 ppm. Protons from the chain are observed at 3.71 ppm and 4.05 ppm, that closer to the alcohol and ether groups, respectively. In the ^13^C MNR spectrum, there are peaks at 60.23 ppm and 74.53 ppm, respectively. For compound **5b**, the singlet from the proton of the alcohol group is observed at 4.74 ppm. The signal from protons closer to the alcohol group is observed as a triplet at 3.70 ppm, and for protons closer to the ether group is observed at 4.00 ppm as a triplet. On the ^13^C NMR spectrum, these are the signals at 60.23 ppm and 74.53 ppm, respectively. All NMR spectra from the other compounds are included in the Supplementary Materials (Figures S1–S132).

### 2.3. UV-Vis Study

**Figure 4:**
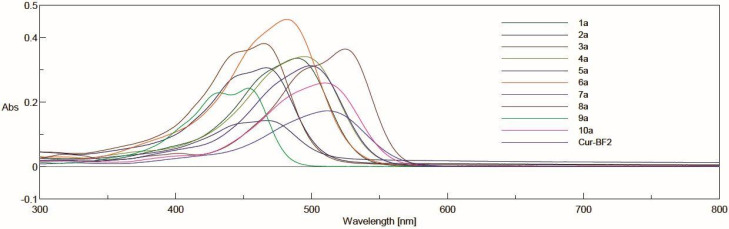
The absorption spectra in acetonitrile for **Cur-BF_2_** and derivatives **1a**–**10a**. The compounds were dissolved at a concentration of 5 µM in acetonitrile, and acetonitrile was used as a blank. The spectra were recorded using a standard cuvette port for single-sample absorbance.

**Figure 5:**
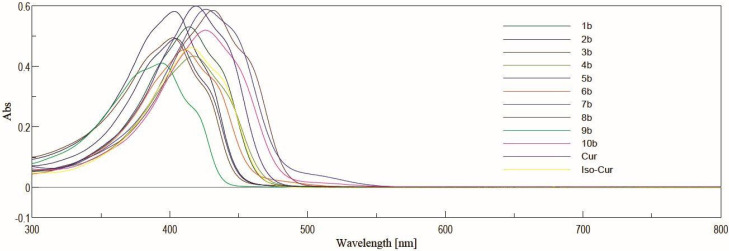
The absorption spectra in acetonitrile for **Cur**, **Iso-Cur,** and derivatives **1b**–**10b**. The compounds were dissolved at a concentration of 10 µM in acetonitrile, and acetonitrile was used as a blank. The spectra were recorded using a standard cuvette port for single-sample absorbance.

UV-Vis spectra were performed for solutions in acetonitrile at the concentrations 5 µM and 10 µM for complexes with BF_2_ and derivatives after decomplexation, respectively (Figure 4 and Figure 5). In general, the range of absorbance maxima is wide for each series. In the series of curcuminoid complexes with BF_2_, the absorbance maxima for these compounds are in the range from 453 nm to 525 nm. For derivatives with a free keto-enol moiety, the values are from 394 to 431 nm. Depending on the derivative, the difference in λ_max_ after decomplexation is from 59 nm to 94 nm.

### 2.4. X-Ray Diffraction Studies

**Figure 6:**
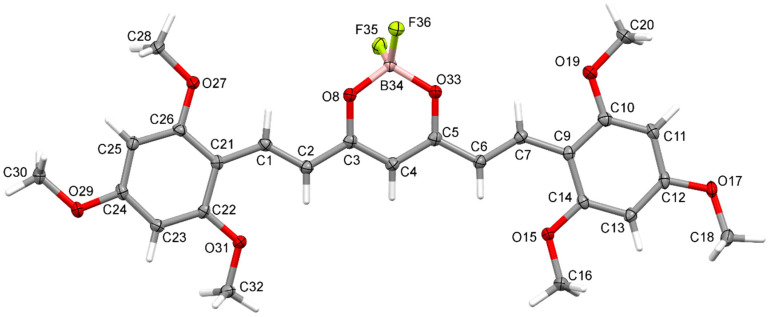
Anisotropic ellipsoid representation of (1E,4Z,6E)-5-((difluoroboranyl)oxy)-1,7-bis(2,4,6-trimethoxyphenyl)hepta-1,4,6-trien-3-one **8a**, showing the atom-labeling scheme. Displacement ellipsoids are drawn at the 50% probability level, and H atoms are shown as small spheres of arbitrary radii.

**Figure 7:**
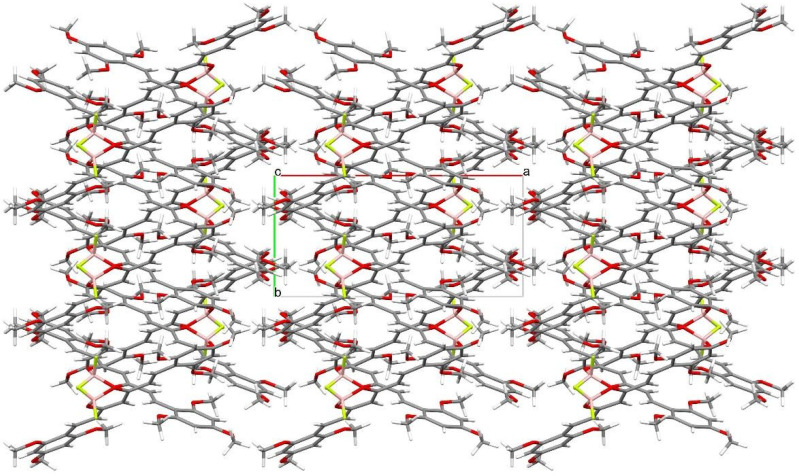
Packing of molecules (1E,4Z,6E)-5-((difluoroboranyl)oxy)-1,7-bis(2,4,6-trimethoxyphenyl)hepta-1,4,6-trien-3-one, along the direction [001]. Atomic colors according to the convention adopted for Figure 6.

**Figure 8:**
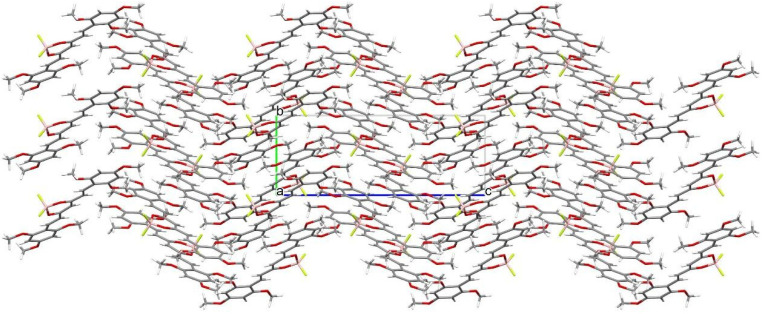
Packing of molecules (1E,4Z,6E)-5-((difluoroboranyl)oxy)-1,7-bis(2,4,6-trimethoxyphenyl)hepta-1,4,6-trien-3-one, along the direction [100]. Atomic colors according to the convention adopted for Figure 6.

Crystal structure of (1E,4Z,6E)-5-((difluoroboranyl)oxy)-1,7-bis(2,4,6-trimethoxyphenyl)-hepta-1,4,6-trien-3-one was determined by single-crystal X-ray diffraction. Crystals were grown from DMF by slow evaporation of the solution. The crystal structure of C_23_H_27_BF_2_O_8_ is monoclinic with P21/c space group. The asymmetric unit of C_23_H_27_BF_2_O_8_ contains one molecule in the asymmetric unit. Atom labeling of C_23_H_27_BF_2_O_8_ is shown in Figure 6. Packing of molecule **8a** along the directions [001] and [100] are shown in Figure 7 and Figure 8.

Analysis of the X-ray data showed that the C_23_H_27_BF_2_O_8_ molecule is not quite planar. The C(9)-C(14) and C(21)-C(26) rings are deviated from the plane of the molecule by 5.73(7) and 12.59(6) degrees, respectively. CH···O and CH···F interactions cause a bending of the molecule as shown by the increased valence angles C(21)-C(1)-C(2) 131.0(1) and C(6)-C(7)-C(9) 130.7(1). Intermolecular CH-F interactions to the F(35) atom also cause the boron atom to deviate from the plane formed by the O(8)-C(3)-C(4)-C(5)-O(33) atoms. The angle to the plane formed by the O(33)-B(34)-O(8) atoms is 22.3(1) degrees. In the crystal structure of C_23_H_27_BF_2_O_8_, weak interactions of C-H···O and CH···F-type connect the molecules in a three-dimensional network. The rest of the crystallographic data is included in Table 2 and in the Supplementary Materials (Tables S1–S6).

### 2.5. Biological Activity of Tested Compounds

The results of cell assays show that all obtained derivatives, irrespective of their activity against pathological cells, show low cytotoxicity to healthy cells (Balb/3T3 fibroblast line). When comparing IC_50_ values to natural curcumin, which is considered safe and non-toxic, we can see that most derivatives display comparable or even lower cytotoxicity. The exception is compound **6b** with an IC_50_ value of 20.88 + −3.84 µM. In contrast, doxorubicin, which was used as a reference compound, showed the same cytotoxicity against both normal and cancerous cells.

Considering activity against abnormal cells, the presence of the BF_2_ group in the central part of the molecule appears to be crucial. The absence of this group resulted in a decrease in anticancer activity of about 2–10 times in most cases. In extreme instances (derivative **9a**/**9b**), the lack of this group resulted in an approximately 45-fold decrease in activity against the BA/F3 wt and BA/F3 ins5 lines and an approximately 70-fold decrease against the BA/F3 del52 line (Table 3). Activity charts in the 72 h MTT test are included in the Supplementary Materials (Figure S165).

#### 2.5.1. Structure–Activity Relationships for Curcuminoids with Methoxyl Groups

**Figure 9:**
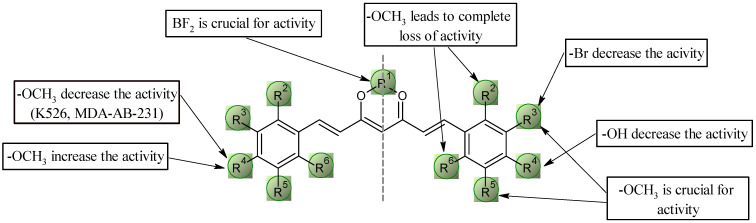
Key structural elements for the methoxyl curcuminoids group.

**Figure 10:**
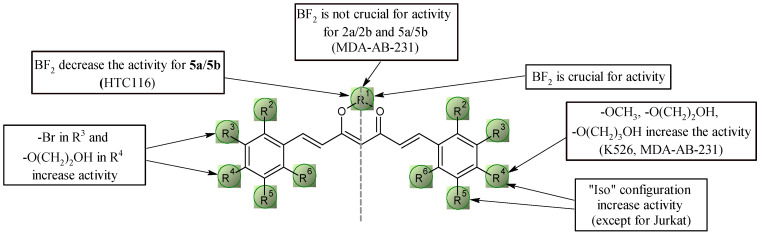
Key structural elements for curcuminoids and bromo-curcuminoids.

A preliminary analysis of the structure–activity relationship analysis grouped the results by cell line into five categories. The most important conclusions are presented graphically in Figure 9 and Figure 10. In the first group, which includes the BA/F3 wt, BA/F3 del52, and BA/F3 ins5 cell lines, and the second group represented by the Jurkat cell line, compounds **6a** and **9a** exhibited the highest activity. These two derivatives have very similar IC_50_ values to doxorubicin. The most favorable configuration seems to be the presence of methoxyl groups at positions 3^2^, 5^2^, and/or 4^2^. Given that the most active derivatives are **6a** and **9a**, the absence of the -OH group in position 4^2^ is crucial, as this resulted in an approximately 10-fold increase in activity against these cell lines. It is interesting to note that changing the position of the methoxyl group from 3^2^ or 5^2^ to 2^2^ or 6^2^ results in an almost complete loss of activity against all lines tested. The replacement of one of the methoxyl groups with bromine at position 3^2^ also appears to be an unfavorable change, resulting in an approximately 10-fold decrease in activity.

In the case of the K562 leukemia cell line, the most favorable configuration is the presence of methoxy groups at positions 3^2^ and 5^2^ (compound **9a**), resulting in the same IC_50_ value as for doxorubicin. The presence of a methoxyl or -OH group at position 4^2^ leads to a decrease in activity by four- and nine-fold, respectively. Similarly, substituting the methoxy group with bromine at position 3^2^ leads to a significant reduction in activity. Concerning the presence of the BF_2_ group, the relationships follow the general trend except for the pair of compounds **6a**–**6b**, where the differences in the activity of these compounds are not significant.

In the case of HTC116 colon cancer cells, structure–activity relationships are similar—the presence of methoxyl groups at positions 3^2^, 5^2^, and/or 4^2^ seems to be the most favorable configuration, leading to IC_50_ values the same as doxorubicin. As in the above-mentioned group in the case of pair **6a**–**6b**, the absence of the BF_2_ group does not lead to a decrease in activity.

For the MDA-MB-231 cell line, the structure–activity relationship remains consistent—the replacement of methoxyl for bromine at position 3^2^ and the unblocking of the -OH group at position 4^2^ results in an approximately 10-fold decrease in activity. Methoxyl groups at positions 2^2^, 4^2^, and/or 6^2^ are an undesirable configuration, as it is characterized by a complete lack of activity against this cell line. The most favorable configuration is the presence of methoxy groups at positions 3^2^ and 5^2^ (compound **9a**).

#### 2.5.2. Structure–Activity Relationships for Curcuminoids and Bromo-Curcuminoids

In case of BA/F3 wt, BA/F3 del52, Ba/F3 ins5 cell lines **2a** and **Cur-BF_2_** have the highest activity (comparable to doxorubicin). In the context of brominated curcumin derivatives, the key is the presence of bromine at position 3^2^ and the substitution of the alcohol chain via an ether bond at position 4^2^. The length of the chain is also important; extending it by one -CH_2_- group resulted in an approximately 10-fold decrease in activity. Blocking the -OH group at position 4^2^ with a methyl group is also a favorable modification, which resulted in a two–four-fold increase in activity. In the case of curcumin, the “iso” configuration (methoxyl group in the *para* position, hydroxyl group in the *meta* position) is more desirable. This leads to a 2–5-fold increase in activity. In contrast, for brominated curcuminoids, no significant change in activity was observed.

Very similar relationships can be observed for activity against cells of the Jurkat line. Although in the case of the “iso” configuration, no significant increase in activity is observed (Figure 10).

In the case of the K562 leukemia cell line, compounds **Cur-BF_2_** and **2a** showed high levels of activity, although weaker than doxorubicin. As is the case for natural curcumin, for the series of brominated derivatives, the more favorable configuration is the “iso”, in which an approximately four-fold increase in activity is observed. The most favorable structural modification within this group of compounds is the introduction of an alcohol chain at position 4^2^ (compound **2a**), with neither elongation of the chain by an additional -CH_2_- group nor substitution with a methyl group significantly affecting anticancer activity.

In the case of HTC116 colon cancer cells, we can observe a slightly different correlation. First of all, the “iso” configuration is not characterized by greater activity against this cell line. Although the most active compounds are **2a**, **Cur-BF_2_**, **6a**, **6b**, and **9a**, an inverse relationship is characterized by the pair of compounds **5a** and **5b**. Namely, the absence of the BF_2_ group results in an approximately four-fold increase in activity.

In the case of the MDA-MB-231 breast cancer cell line, only one compound (**9a**) has a similar level of activity to doxorubicin. Most of the compounds in the series show good cytotoxic activity at around 4–7 µM. As before, the “iso” configuration for both natural curcumin and its derivative contribute to an approximately five-fold increase in activity. For a series of brominated derivatives, the key is to block the -OH group at position 4^2^ with a methyl group or a short alcohol chain. Interestingly, in this case, the chain length does not matter. It is also interesting to note the level of activity of the derivatives with the alcohol group, particularly in terms of the presence of the BF_2_ group; the loss of this group does not result in a decrease in activity.

#### 2.5.3. Fluorescence Microscopic Analysis of Cell Death Induced by the Most Active Compounds

**Figure 11:**
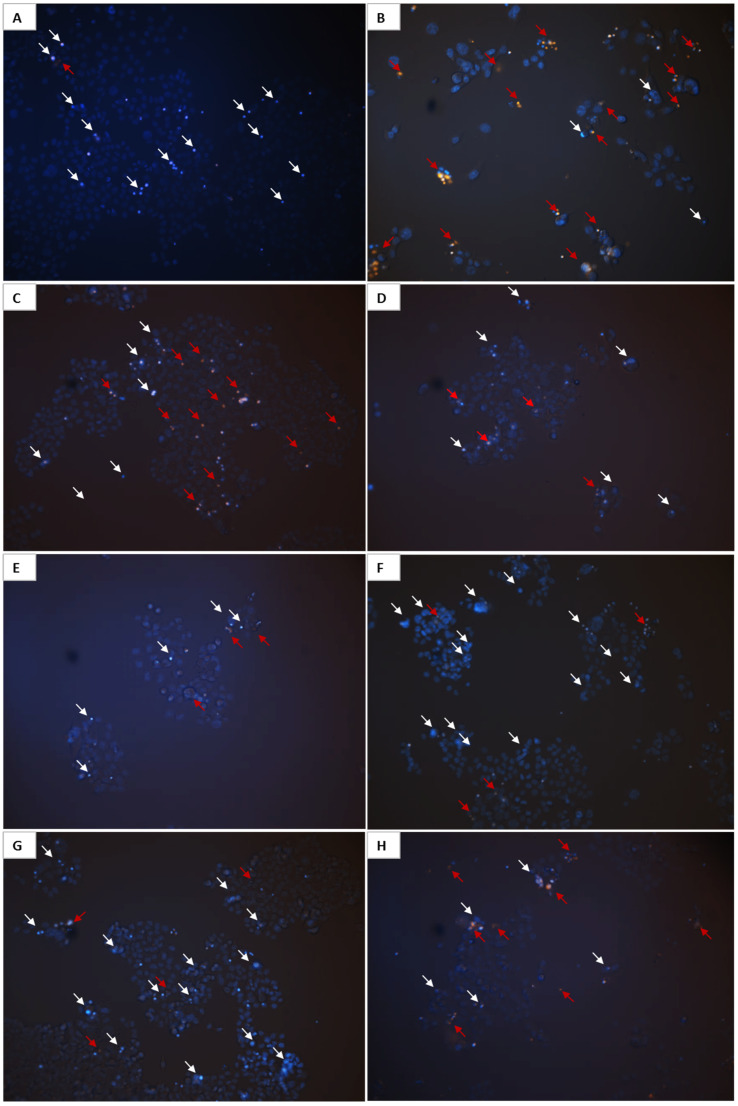
Representative fluorescent images of HCT-116 cells treated with 0.5 μM doxorubicin (**B**), 1 μM **Cur** (**C**), **5b** (**D**), **6a** (**E**), **6b** (**F**), **Cur-BF_2_** (**G**), **9a** (**H**), and control cells (**A**) for 72 h, stained with Hoechst 33342/EtBr. White arrows indicate chromatin condensation; red arrows indicate dead cells. Magnification is 200×.

The relationship between the structure and activity of curcuminoids and bromo-curcuminoids, supported by cytotoxicity data demonstrating their varied impact on cell survival, is illustrated through fluorescence images of HCT-116 cells. These cells, treated with the most potent compounds for 72 h and stained with Hoechst 33342/EtBr, reveal how structural differences affect cell shape and condition. Doxorubicin (0.5 µM), used as a positive control, shows the highest level of cell death. In the doxorubicin-treated sample (Figure 11B), the intense orange/red fluorescence of EtBr indicates significant membrane damage and cell death, due to its well-known mechanism of intercalating into DNA and inhibiting topoisomerase II, leading to DNA damage and apoptosis or necrosis, as evidenced by fragmented or diffusely stained nuclei reflecting this cytotoxic effect. The control cells (A) show normal nuclear morphology with uniform blue Hoechst 33342 staining and low amounts of orange/red EtBr signal, indicating healthy, intact cells with no significant chromatin condensation or cell death. Curcumin (C, 1 µM) exhibits a moderate cytotoxic effect with fewer dead cells marked by orange/red EtBr fluorescence than in doxorubicin. Additionally, several cells display condensed chromatin indicated by white arrows, suggesting a mild induction of apoptosis. Samples treated with 1 µM **5b** (D), **6a** (E), and **9a** (H) show a markedly reduced cell number compared to the control and other samples along with a small amount of orange/red EtBr staining, suggesting that these compounds may exert a cytostatic effect. In contrast, **6b** (F) and **Cur-BF2** (G) induce significant chromatin condensation as evidenced by white arrows and prominent Hoechst 33342 staining, suggesting a strong pro-apoptotic activity, with some cells transitioning to death as indicated by minor orange/red EtBr signals.

**Figure 12:**
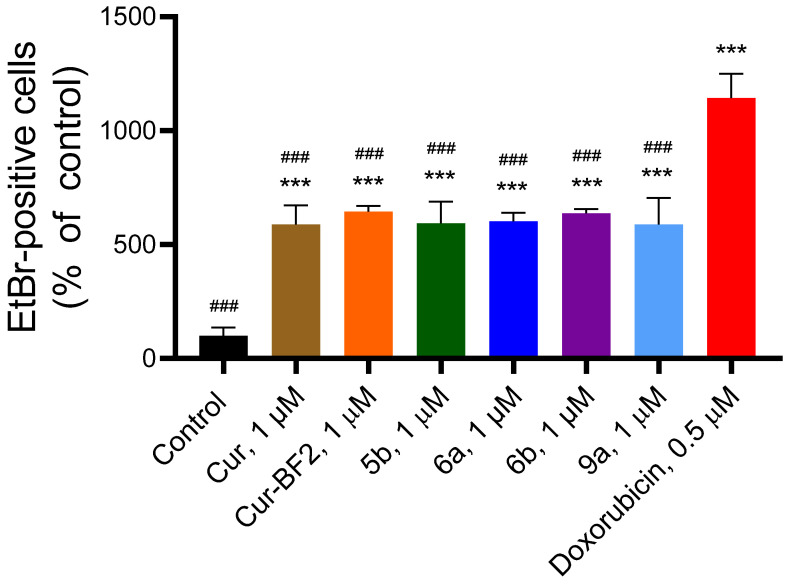
Compounds **Cur**, **5b**, **6a**, **6b**, **Cur-BF_2_**, and **9a** (1 μM) influenced the level of EtBr-positive cells in the HCT-116 cells after 72 h treatment. The data are presented as mean ± standard deviation (M ± SD). ***—*p* < 0.001 (significant changes compared to the control cells; ###—*p* < 0.001 significant changes compared to the effect of doxorubicin (1 μM).

The studied compounds significantly increased the fluorescence of EtBr in treated HCT-116 cells (Figure 12).

Compounds **Cur**, **5b**, **6a**, **6b**, **Cur-BF_2_**, and **9a** (1 μM) elevated the content of EtBr-positive cells by 5.9–6.4 times as compared to the control (non-treated) HCT-116 cells.

#### 2.5.4. The Affinity of Studied Compounds Towards DNA

DNA fragmentation and chromatin condensation are terminal hallmarks of apoptosis and are frequently induced by anticancer compounds. We used the spectroscopic diphenylamine assay to evaluate the amount of DNA fragmentation in HCT-116 cells under their treatment with the studied compounds and doxorubicin.

**Figure 13:**
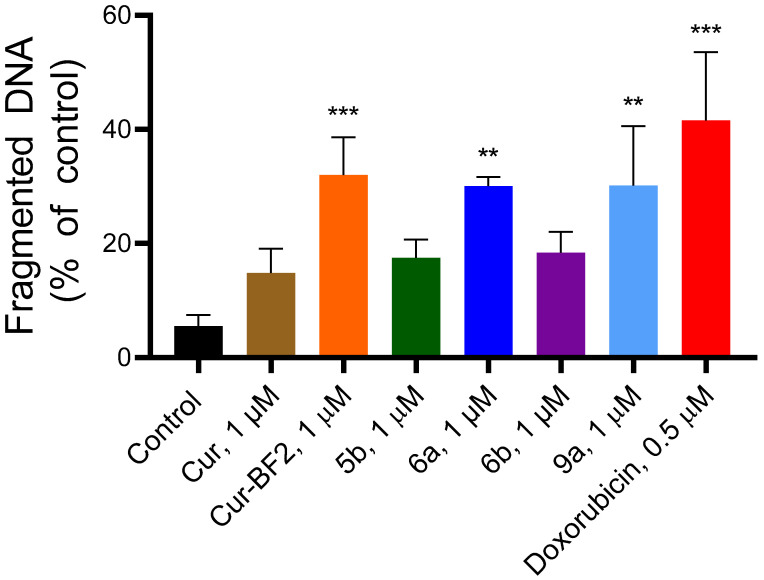
The percentage of fragmentation DNA in the HCT-116 cells after 72 h treatment with compounds **Cur**, **5b**, **6a**, **6b**, **Cur-BF_2_**, and **9a** (1 μM), and doxorubicin (0.5 μM). The data are presented as M ± SD. **—*p* < 0.01 and ***—*p* < 0.001 (significant changes compared to the control cells).

An increased content of the fragmented DNA was observed in the treated cells under the action of the **Cur**, **5b**, **6a**, **6b**, **Cur-BF_2_**, and **9a** and Dox (Figure 13). The **Cur** at 1 µM induced DNA fragmentation at the level of 14.81 ± 4.24%, **5b** induced 17.49 ± 3.19% of DNA fragmentation, and **6b** induced 18.39 ± 3.61% of DNA fragmentation. Compounds **Cur-BF_2_**, **6a**, and **9a** at 1 µM induced a higher DNA fragmentation level (32.02 ± 6.62%, 30.09 ± 1.60%, and 30.20 ± 10.36%, respectively). Doxorubicin at 0.5 µM caused 41.55 ± 12.00% of DNA fragmentation in HCT-116 cells (Figure 12).

**Figure 14:**
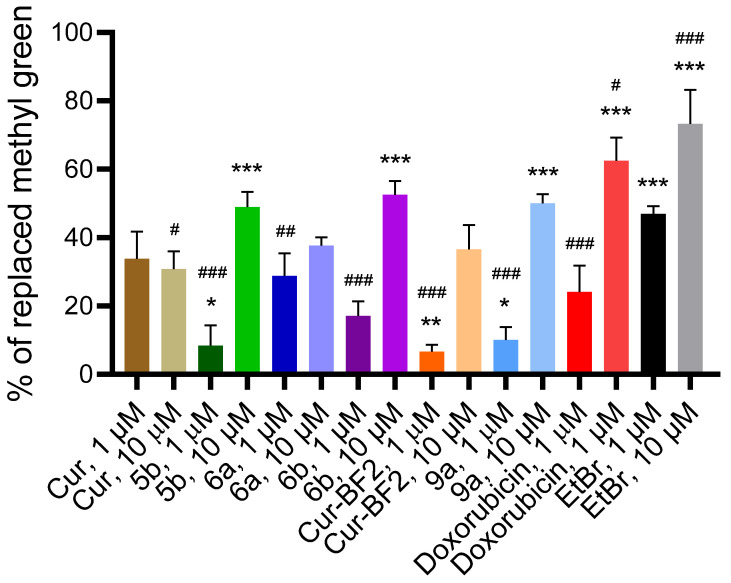
The percentage of methyl green displaced from its DNA complex by the tested compounds, as well as by the reference agents doxorubicin and EtBr, at 1 and 10 μM. *—*p* < 0.05, **—*p* < 0.01, ***—*p* < 0.001 (significant changes compared with the effect of doxorubicin (1 μM); #—*p* < 0.05, ##—*p* < 0.01, ###—*p* < 0.001 significant changes compared with the impact of EtBr (1 µM). Data are presented as mean ± standard deviation.

When a compound directly interacts with DNA (through intercalation, alkylation, or inhibition of topoisomerases), the link to these apoptotic events is direct. We used the spectroscopic DNA/methyl green displacement assay to identify the ability of the tested compounds to intercalate into the DNA molecule (Figure 14). When an intercalator or groove-binding compound is introduced, it competes with methyl green for DNA binding sites. A higher displacement of methyl green indicates a stronger interaction of the compound with DNA.

The known intercalator doxorubicin displaced 19.52–58.33% of methyl green from its DNA complex (Figure 14). Another intercalator, EtBr, displaced 45.24–61.90% of methyl green from its DNA complex. The tested compounds exhibited DNA-methyl green displacement activity comparable to that of DOX and EtBr. Compounds **Cur**, **5b**, **6a**, **6b**, **Cur-BF_2_**, and **9a** (1 μM) displaced methyl green from the DNA–methyl green complex by 2.50–27.50%. Compounds **Cur**, **5b**, **6a**, **6b**, **Cur-BF_2_**, and **9a** (10 μM) demonstrated higher ability to replace methyl green from the DNA–methyl green complex (25.00–50.00%). These results indicate that compounds **Cur**, **5b**, **6a**, **6b**, **Cur-BF_2_**, and **9a** possess a high affinity for DNA via intercalation. One can say that the studied compounds directly interact with DNA.

#### 2.5.5. Tested Compounds Influence Reactive Oxygen Species Level in HCT-116 Cells

**Figure 15:**
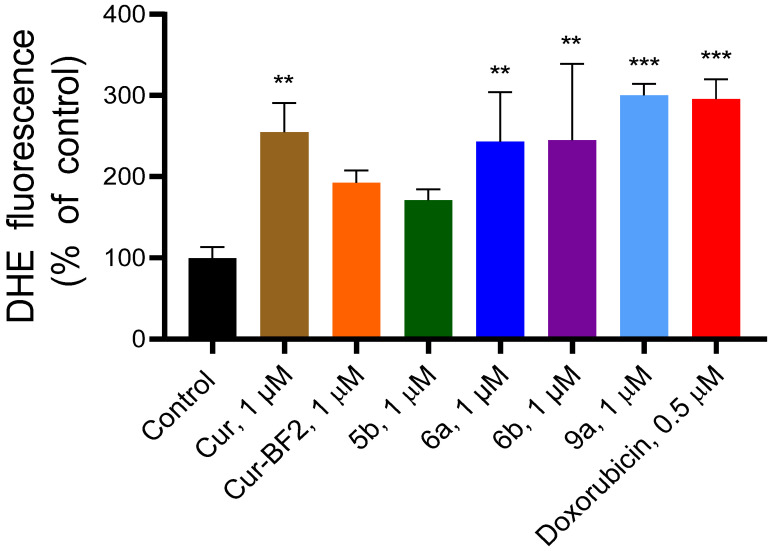
Compounds **Cur**, **5b**, **6a**, **6b**, **Cur-BF_2_**, and **9a** (1 μM) induced the elevation of DHE fluorescence in the HCT-116 cells after 72 h treatment. The data are presented as M ± SD. **—*p* < 0.01 and ***—*p* < 0.001 (significant changes compared to the control cells).

The ability of **Cur**, **5b**, **6a**, **6b**, **Cur-BF_2_**, and **9a** (1 μM) to elevate the reactive oxygen species (ROS) level in HCT-116 cells was found using dihydroethidium (DHE) staining of cells (Figure 15). The fluorescent probe dihydroethidium (DHE) is readily taken up by cells and reacts specifically with superoxide radicals to form the red fluorescent product 2-hydroxyethidium (2-OH-E^+^), allowing selective detection of intracellular O_2_^−^. However, DHE can also undergo nonspecific oxidation to ethidium (E^+^), whose fluorescence overlaps with that of 2-OH-E^+^. Thus, the combined fluorescence of 2-OH-E^+^ and E^+^ provides a reliable measure of overall ROS generation associated with superoxide production [21]. The **Cur** increased the ROS content by 2.55 times, compound **5b**—by 1.71 times, **6a**—by 2.43 times, **6b**—by 2.45 times, **Cur-BF_2_**—by 1.93 times, and **9a**—by 3 times as compared to the control. Doxorubicin at 0.5 µM increased the ROS level in HCT-116 cells by 2.96 times. The obtained results correlate with the data that curcumin, demethoxycurcumin, and bisdemethoxycurcumin induced apoptosis in tumor cells via DNA damage and ROS production [9].

Thus, **Cur**, **5b**, **6a**, **6b**, **Cur-BF_2_**, and **9a** elevated cellular ROS content in HCT-116 cells.

## 3. Materials and Methods

### 3.1. Materials

All substrates and solvents used in the synthesis were purchased from either Alfa Aesar, TCI, or Merck. NMR spectra were recorded at 298 K either on a Bruker Avance III 500 spectrometer (Bruker Daltonics, Bremen, Germany) using a PABBO BBF/1Hand19F 5 mm probe or an Agilent DD2 800 spectrometer (Agilent Technologies, Santa Clara, CA, USA) equipped with a 5 mm ^1^H(^13^C/^15^N) probe head. UV-Vis spectra were recorded on a UV-Vis Jasco V-770 spectrometer (JASCO, Tokyo, Japan). The chromatographic analysis of new compounds purity was performed on an Agilent 1260 Infinity II LC System (Agilent Technologies, Bolinem, Germany) equipped with a quaternary pump (model G7111B) and degasser, a vial sampler (model G7129A) set at 25 °C, multicolumn thermostat (model G7116A) set at 25 °C, and diode array (DAD WR, model G7115A) detector. The DAD detector was used for the purity test, and the detection wavelength for the DAD detector was adjusted for each compound tested at its absorption maxima. The ESI-MS analyses were performed on the Q-TRAP 4000 mass spectrometer from AB-Sciex (Foster City, CA, USA). All ESI-MS spectra are included in the Supplementary Materials (Figures S133–S164). The melting point (M.p) was determined using a “Stuart” apparatus (Bibby Sterlin Ltd., Caerphilly Wales, UK), using single-ended capillaries.

#### 3.1.1. Reagents Used for In Vitro Experiments

MTT (3-[4,5-dimethylthiazol-2-yl]-2,5-diphenyl tetrazolium bromide) test, crystal violet, Hoechst-33342, EtBr, DHE, doxorubicin, dimethylsulfoxide (DMSO), salmon sperm DNA, Tris, EDTA, Triton X-100, diphenylamine, and methyl green were purchased from Sigma-Aldrich, St. Louis, MO, USA. Trichloroacetic acid, glacial acetic acid, acetaldehyde solution, and H_2_SO_4_ were purchased from SferaSim (Lviv, Ukraine).

#### 3.1.2. Cell Lines

MDA-MD-231 human breast adenocarcinoma cell line, HCT-116 colon adenocarcinoma cell line, Jurkat human T lymphocyte cell line, K562 human chronic myelogenous leukemia cell line, and BALB/3T3 mouse embryonic fibroblasts were kindly provided by a Collection at the Institute of Molecular Biology and Genetics, National Academy of Sciences of Ukraine (Kyiv, Ukraine). Murine interleukin-3-dependent pro-B cell lines Ba/F3 wt, Ba/F3 Calr del52, and Ba/F3 Calr ins5 were kindly provided by Prof. Robert Kralovics, Medical University of Vienna, Austria. Cells were maintained in DMEM (Biowest, France) or RPMI-1640 medium (Biowest, France), containing 10% of fetal bovine serum (FBS, Biowest, France) according to recommendations of American Type Culture Collection (ATCC), under the incubation conditions of 5% CO_2_ humidity at 37 °C.

### 3.2. Synthesis

#### 3.2.1. Aldehyde Synthesis–General Procedure

Aromatic aldehyde alkylation was performed using the standard nucleophilic substitution method. To a solution of 5-bromovanillin in DMF, 1.05 equivalents of iodomethane, 2-bromoethanol, or 3-chloropropan-1-ol, and 1.05 equivalents of K_2_CO_3_ (for compound **17**) or LiOH monohydrate (for compounds **18** and **19**) were added. The reaction was then heated to 80 °C and stirred for 24 h. After that, the reaction was quenched with distilled water. Then, the product was extracted with chloroform from water alkalized with sodium hydroxide (pH ca. 11) and purified by hot recrystallization from hexane.

##### 3-Bromo-4,5-dimethoxybenzaldehyde (**17**) ESI [M + H]^+^ *m*/*z* 244.98

^1^H NMR (800 MHz, CDCl_3_) δ 9.85 (s, 1H), 7.66 (d, *J* = 1.8 Hz, 1H), 7.39 (d, *J* = 1.8 Hz, 1H), 3.95 (s, 3H), 3.94 (s, 3H).

^13^C NMR (201 MHz, CDCl_3_) δ 189.85, 154.82, 151.82, 133.05, 128.77, 117.94, 110.12, 60.84, 56.26.

R_f_(hexane/ethyl acetate 2:1): 0.75, yield 98.9% white amorphous solid.

##### 3-Bromo-4-(2-hydroxyethoxy)-5-methoxybenzaldehyde (**18**) ESI [M + H]^+^ *m*/*z* 274.99

^1^H NMR (800 MHz, CDCl_3_) δ 9.86 (s, 1H), 7.67 (d, *J* = 1.8 Hz, 1H), 7.41 (d, *J* = 1.8 Hz, 1H), 4.37–4.19 (m, 2H), 3.95 (s, 3H). 3.91–3.79 (m, 2H), 2.74 (t, *J* = 6.5 Hz. 1H).

^13^C NMR (201 MHz, CDCl_3_) δ 189.82, 154.09, 150.70, 133.38, 128.98, 118.20, 110.25, 75.88, 62.05, 56.51.

R_f_(ethyl acetate): 0.74, yield 70.1% white crystals.

##### 3-Bromo-4-(3-hydroxypropoxy)-5-methoxybenzaldehyde (**19**) ESI [M + H]^+^ *m*/*z* 289.00

^1^H NMR (800 MHz, DMSO-*d6*) δ 9.88 (s, 1H), 7.77 (s, 1H), 7.53 (s, 1H), 4.48 (t, *J* = 5.1 Hz, 1H), 4.15 (t, *J* = 6.5 Hz, 2H), 3.90 (s, 3H), 3.59 (dd, *J* = 11.6, 6.3 Hz, 2H), 1.86 (p, *J* = 6.4 Hz, 2H).

^13^C NMR (201 MHz, DMSO-*d6*) δ 190.91, 153.70, 150.07, 132.84, 127.22, 117.16, 111.49, 70.85, 57.52, 56.32, 33.18.

R_f_(dichloromethane/acetone 10:1): 0.68, yield 74.4% white amorphous solid.

#### 3.2.2. Curcuminoids Synthesis

Curcuminoid complexes with BF_2_ (**1**–**10a**) were synthesized by the condensation reaction of a BF_2_ complex of acetylacetone with the corresponding aldehyde (2.1 equivalent) in toluene. Tributyl borate (2 equivalents) was added as a dehydrating agent. Then, *n*-butylamine (0.2 equivalent) was added dropwise to the reaction mixture over 15 min. The reaction was carried out at 65 °C in nitrogen for 24 h. The dark residue was separated by filtration, then washed with a small amount of toluene and dried under vacuum. Derivative **2a** was purified by 3-fold recrystallization. The total was dissolved in THF and then precipitated with 2-fold volume of toluene. The rest of the compounds were purified using column chromatography.

In order to obtain free curcuminoids, compounds from the first series were decomplexed. The curcuminoid complex with BF_2_ was suspended in a methanol/water (4:1) mixture, and disodium oxalate (2 equivalents) was added. The reaction mixture was heated at 140 °C for 15 min under microwave conditions in an Anton Paar Monowave 400 microwave reactor. The precipitate was filtered and purified by column chromatography.

Compounds **Cur-BF_2_**, **Cur**, and **Iso-Cur** were obtained from a library of compounds synthesized earlier in previous research work [6,20].

(1E,4Z,6E)-5-((difluoroboranyl)oxy)-1,7-bis(3-bromo-4-hydroxy-5-methoxyphenyl)hepta-1,4,6-trien-3-one (**1a**) ESI [M − H]^−^ *m*/*z* 572.94, UV-Vis λ_max_ = 489 nm, ε = 67,012 dm^3^·mol^−1^·cm^−1^, M.p. = 238 °C (dec.),

^1^H NMR (800 MHz, DMSO-*d6*) δ 10.48 (s, 2H), 7.89 (d, *J* = 15.5 Hz, 2H), 7.72 (d, *J* = 1.8 Hz, 2H), 7.50 (d, *J* = 1.8 Hz, 2H), 7.12 (d, *J* = 15.5 Hz, 2H), 6.40 (s, 1H), 3.90 (s, 6H).

^13^C NMR (201 MHz, DMSO-*d6*) δ 178.67, 148.58, 148.12, 145.56, 127.32, 126.49, 119.24, 111.67, 109.98, 101.73, 56.44.

R_f_(ethyl acetate): 0.75, yield 95.0% crimson amorphous solid.

(1E,4Z,6E)-5-((difluoroboranyl)oxy)-1,7-bis(3-bromo-4-(2-hydroxyethoxy)-5-methoxyphenyl)hepta-1,4,6-trien-3-one (**2a**) ESI [M-F]^+^ *m*/*z* 642.99, UV-Vis λ_max_ = 466 nm, ε = 61,111 dm^3^·mol^−1^·cm^−1^, M.p. = 215 °C (dec.),

^1^H NMR (800 MHz, DMSO-*d6*) δ 7.99 (d, *J* = 15.6 Hz, 2H), 7.80 (d, *J* = 1.8 Hz, 2H), 7.61 (d, *J* = 1.9 Hz, 2H), 7.31 (d, *J* = 15.7 Hz, 2H), 6.54 (s, 1H), 4.76 (t, *J* = 5.6 Hz, 2H), 4.05 (t, *J* = 5.5 Hz, 4H), 3.90 (s, 6H), 3.71 (q, *J* = 5.5 Hz, 4H).

^13^C NMR (201 MHz, DMSO-*d6*) δ 179.80, 153.37, 148.00, 145.32, 131.20, 125.91, 121.76, 117.40, 113.45, 102.43, 74.68, 60.26, 56.42.

R_f_(ethyl acetate): 0.67, yield 66.2% orange amorphous solid.

(1E,4Z,6E)-5-((difluoroboranyl)oxy)-1,7-bis(3-bromo-4,5-dimethoxyphenyl)hepta-1,4,6-trien-3-one (**3a**) ESI [M + Na]^+^ *m*/*z* 624.96, UV-Vis λ_max_ = 465 nm, ε = 76,119 dm^3^·mol^−1^·cm^−1^, M.p. = 252–254 °C (dec.),

^1^H NMR (800 MHz, DMSO-*d6*) δ 7.99 (d, *J* = 15.7 Hz, 2H), 7.80 (d, *J* = 1.6 Hz, 2H), 7.63 (d, *J* = 1.6 Hz, 2H), 7.31 (d, *J* = 15.7 Hz, 2H), 6.54 (s, 1H), 3.91 (s, 6H), 3.82 (s, 6H).

^13^C NMR (201 MHz, DMSO-*d6*) δ 179.86, 153.52, 148.50, 145.28, 131.48, 125.77, 121.91, 117.23, 113.54, 102.47, 60.39, 56.40.

R_f_(ethyl acetate): 0.67, yield 23.4% light orange amorphous solid.

(1E,4Z,6E)-5-((difluoroboranyl)oxy)-1,7-bis(2-bromo-5-hydroxy-4-methoxyphenyl)hepta-1,4,6-trien-3-one (**4a**) ESI [M + H]^+^ *m*/*z* 575.00, UV-Vis λ_max_ = 495 nm, ε = 68,040 dm^3^·mol^−1^·cm^−1^, M.p. = 257–259 °C (dec.),

^1^H NMR (800 MHz, DMSO-*d6*) δ 9.68 (s, 2H), 8.09 (d, *J* = 15.4 Hz, 2H), 7.44 (s, 2H), 7.30 (s, 2H), 7.04 (d, *J* = 15.4 Hz, 2H), 6.61 (s, 1H), 3.89 (s, 3H).

^13^C NMR (201 MHz, DMSO-*d6*) δ 179.15, 152.33, 146.61, 143.56, 125.32, 121.11, 117.24, 116.19, 114.13, 103.12, 56.33.

R_f_(ethyl acetate): 0.52, yield 42.6% dark maroon amorphous solid.

(1E,4Z,6E)-5-((difluoroboranyl)oxy)-1,7-bis(3-bromo-4-(3-hydroxypropoxy)-5-methoxyphenyl)hepta-1,4,6-trien-3-one (**5a**) ESI [M + H]^+^ *m*/*z* 691.00, UV-Vis λ_max_ = 468 nm, ε = 28,454 dm^3^·mol^−1^·cm^−1^, MP = 222–223 °C,

^1^H NMR (800 MHz, DMSO-*d6*) δ 7.98 (d, *J* = 15.6 Hz, 2H), 7.79 (d, *J* = 1.8 Hz, 2H), 7.61 (d, *J* = 1.9 Hz, 2H), 7.30 (d, *J* = 15.7 Hz, 2H), 6.54 (s, 1H), 4.48 (s, 2H), 4.12 (t, *J* = 6.5 Hz, 4H), 3.90 (s, 6H), 3.59 (t, *J* = 6.4 Hz, 4H), 1.86 (p, *J* = 6.5 Hz, 4H).

^13^C NMR (201 MHz, DMSO-*d6*) δ 179.79, 153.44, 147.92, 145.31, 131.16, 125.92, 121.74, 117.37, 113.47, 102.42, 70.82, 57.59, 56.41, 33.20.

R_f_(ethyl acetate): 0.21, yield 42.9% orange amorphous solid.

(1E,4Z,6E)-5-((difluoroboranyl)oxy)-1,7-bis(3,4,5-trimethoxyphenyl)hepta-1,4,6-trien-3-one (**6a**) ESI [M + H]^+^ *m*/*z* 505.10, UV-Vis λ_max_ = 482 nm, ε = 91,108 dm^3^·mol^−1^·cm^−1^, MP = 276–277 °C,

^1^H NMR (800 MHz, CDCl_3_) δ 7.98 (d, *J* = 15.4 Hz, 2H), 6.84 (s, 4H), 6.62 (d, *J* = 15.4 Hz, 2H), 6.09 (s, 1H), 3.93 (s, 6H), 3.92 (s, 12H).

^13^C NMR (201 MHz, CDCl_3_) δ 179.67, 153.58, 147.46, 141.87, 129.41, 119.62, 106.50, 101.99, 61.11, 56.27.

R_f_(ethyl acetate): 0.79, yield 87.7% burgundy amorphous solid.

(1E,4Z,6E)-5-((difluoroboranyl)oxy)-1,7-bis(4-hydroxy-3,5-dimethoxyphenyl)hepta-1,4,6-trien-3-one (**7a**) ESI [M + H]^+^ *m*/*z* 477.3, UV-Vis λ_max_ = 512 nm, ε = 34,437 dm^3^·mol^−1^·cm^−1^, MP = 238–239 °C (dec.),

^1^H NMR (800 MHz, DMSO-*d6*) δ 7.94 (d, *J* = 15.4 Hz, 2H), 7.22 (s, 4H), 7.07 (d, *J* = 15.5 Hz, 2H), 6.44 (s, 1H), 3.84 (s, 12H).

^13^C NMR (201 MHz, DMSO-*d6*) δ 178.57, 148.19, 147.29, 140.49, 124.71, 118.19, 107.75, 101.10, 56.17.

R_f_(ethyl acetate): 0.48, yield 84.5% dark burgundy amorphous solid.

(1E,4Z,6E)-5-((difluoroboranyl)oxy)-1,7-bis(2,4,6-trimethoxyphenyl)hepta-1,4,6-trien-3-one (**8a**) ESI [M + H]^+^ *m*/*z* 505.30, UV-Vis λ_max_ = 525 nm, ε = 72,714 dm^3^·mol^−1^·cm^−1^, MP = 175–177 °C (dec.),

^1^H NMR (800 MHz, DMSO-*d6*) δ 8.22 (d, *J* = 15.7 Hz, 2H), 7.15 (d, *J* = 15.7 Hz, 2H), 6.40 (s, 1H), 6.33 (s, 4H), 3.93 (s, 12H), 3.89 (s, 6H).

^13^C NMR (201 MHz, DMSO-*d6*) δ 178.82, 164.98, 162.11, 136.39, 119.22, 105.39, 102.19, 91.24, 56.23, 55.83.

R_f_(ethyl acetate): 0.72, yield 85.4% maroon amorphous solid.

(1E,4Z,6E)-5-((difluoroboranyl)oxy)-1,7-bis(3,5-dimethoxyphenyl)hepta-1,4,6-trien-3-one (**9a**) ESI [M + H]^+^ *m*/*z* 445.10, UV-Vis λ_max_ = 453 nm, ε = 48,459 dm^3^·mol^−1^·cm^−1^, MP = 201–202 °C,

^1^H NMR (800 MHz, CDCl_3_) δ 7.92 (d, *J* = 15.5 Hz, 2H), 6.71 (d, *J* = 2.1 Hz, 4H), 6.67 (d, *J* = 15.5 Hz, 2H), 6.55 (t, *J* = 2.1 Hz, 2H), 6.10 (s, 1H), 3.82 (s, 12H).

^13^C NMR (201 MHz, CDCl_3_) δ 180.18, 161.13, 147.60, 135.73, 121.02, 106.94, 104.29, 102.31, 55.51.

R_f_(hexane/ethyl acetate 1:1): 0.72, yield 82.8% neon orange amorphous solid.

(1E,4Z,6E)-5-((difluoroboranyl)oxy)-1,7-bis(2,4-dimethoxyphenyl)hepta-1,4,6-trien-3-one (**10a**) ESI [M + H]^+^ *m*/*z* 445.10, UV-Vis λ_max_ = 510 nm, ε = 48,459 dm^3^·mol^−1^·cm^−1^, MP = 181–182 °C (dec.),

^1^H NMR (800 MHz, CDCl_3_) δ 8.23 (d, *J* = 15.6 Hz, 2H), 7.51 (d, *J* = 8.7 Hz, 2H), 6.75 (d, *J* = 15.6 Hz, 2H), 6.54 (dd, *J* = 8.6, 2.3 Hz, 2H), 6.45 (d, *J* = 2.3 Hz, 2H), 5.99 (s, 1H), 3.91 (s, 6H), 3.87 (s, 6H).

^13^C NMR (201 MHz, CDCl_3_) δ 179.59, 164.18, 161.17, 142.39, 132.07, 118.72, 116.82, 105.98, 101.48, 98.40, 55.59.

R_f_(ethyl acetate): 0.83, yield 87.4% dark purple amorphous solid.

(1E,4Z,6E)-5-hydroxy-1,7-bis(3-bromo-4-hydroxy-5-methoxyphenyl)-hepta-1,4,6-trien-3-one (**1b**) ESI [M + H]^+^ *m*/*z* 526.95, UV-Vis λ_max_ = 414 nm, ε = 53,058 dm^3^·mol^−1^·cm^−1^, MP = 234–235 °C (dec.),

^1^H NMR (800 MHz, DMSO-*d6*) δ 16.27 (s, 1H), 10.08 (s, 2H), 7.54 (s, 2H), 7.52 (d, *J* = 2.0 Hz, 2H), 7.38 (d, *J* = 1.8 Hz, 2H), 6.88 (d, *J* = 15.8 Hz, 2H), 6.06 (s, 1H), 3.90 (s, 6H).

^13^C NMR (201 MHz, DMSO-*d6*) δ 183.09, 148.50, 145.96, 139.41, 127.18, 125.56, 122.66, 110.44, 109.56, 101.36, 56.37.

R_f_(ethyl acetate): 0.91, yield 41.1% orange amorphous solid.

(1E,4Z,6E)-5-hydroxy-1,7-bis(3-bromo-4-(2-hydroxyethoxy)-5-methoxyphenyl)-hepta-1,4,6-trien-3-one (**2b**) ESI [M − H]^−^ *m*/*z* 612.99, UV-Vis λ_max_ = 403 nm, ε = 58,156 dm^3^·mol^−1^·cm^−1^, MP = 107–108 °C,

^1^H NMR (800 MHz, DMSO-*d6*) δ 16.08 (s, 1H), 7.60 (d, *J* = 1.7 Hz, 2H), 7.57 (d, *J* = 15.8 Hz, 2H), 7.45 (d, *J* = 1.8 Hz, 2H), 7.00 (d, *J* = 15.9 Hz, 2H), 6.13 (s, 1H), 4.74 (s, 2H), 4.00 (t, *J* = 5.5 Hz, 4H), 3.89 (s, 6H), 3.70 (t, *J* = 5.3 Hz, 4H).

^13^C NMR (201 MHz, DMSO-*d6*) δ 183.06, 153.41, 146.61, 138.87, 131.95, 124.78, 124.66, 117.25, 112.02, 101.93, 74.53, 60.23, 56.33.

R_f_(ethyl acetate): 0.76, yield 38.7% light orange amorphous solid.

(1E,4Z,6E)-5-hydroxy-1,7-bis(3-bromo-4,5-dimethoxyphenyl)-hepta-1,4,6-trien-3-one (**3b**) ESI [M + H]^+^ *m*/*z* 554.98, UV-Vis λ_max_ = 402 nm, ε = 49,437 dm^3^·mol^−1^·cm^−1^, MP = 145–147 °C,

^1^H NMR (800 MHz, DMSO-*d6*) δ 16.07 (s, 1H), 7.59 (d, *J* = 1.9 Hz, 2H), 7.56 (d, *J* = 15.9 Hz, 2H), 7.45 (d, *J* = 1.9 Hz, 2H), 6.99 (d, *J* = 15.9 Hz, 2H), 6.12 (s, 1H), 3.88 (s, 6H), 3.76 (s, 6H).

^13^C NMR (201 MHz, DMSO-*d6*) δ 183.06, 153.54, 147.16, 138.83, 132.23, 124.91, 124.54, 117.08, 112.08, 101.97, 60.24, 56.30.

R_f_(hexane/ethyl acetate 2:1): 0.66, yield 45.3% yellow amorphous solid.

(1E,4Z,6E)-5-hydroxy-1,7-bis(2-bromo-5-hydroxy-4-methoxyphenyl)-hepta-1,4,6-trien-3-one (**4b**) ESI [M + H]^+^ *m*/*z* 527.00, UV-Vis λ_max_ = 417 nm, ε = 43,399 dm^3^·mol^−1^·cm^−1^, MP = 242–245 °C,

^1^H NMR (800 MHz, DMSO-*d6*) δ 7.77 (d, *J* = 15.7 Hz, 2H), 7.32 (s, 2H), 7.22 (s, 2H), 6.74 (d, *J* = 15.6 Hz, 2H), 6.14 (s, 1H), 3.85 (s, 6H).

^13^C NMR (201 MHz, DMSO-*d6*) δ 182.70, 150.73, 146.46, 137.81, 125.95, 124.21, 115.94, 115.03, 113.50, 102.63, 56.12.

R_f_(hexane/ethyl acetate 1:1): 0.19, yield 89.6% orange amorphous solid.

(1E,4Z,6E)-5-hydroxy-1,7-bis(3-bromo-4-(3-hydroxypropoxy)-5-methoxyphenyl)-hepta-1,4,6-trien-3-one (**5b**) ESI [M + H]^+^ *m*/*z* 643.2, UV-Vis λ_max_ = 404 nm, ε = 49,188 dm^3^·mol^−1^·cm^−1^, MP = 134–135 °C,

^1^H NMR (800 MHz, DMSO-*d6*) δ 16.08 (s, 1H), 7.60 (d, *J* = 1.8 Hz, 2H), 7.57 (d, *J* = 15.8 Hz, 2H), 7.45 (d, *J* = 1.9 Hz, 2H), 7.00 (d, *J* = 15.9 Hz, 2H), 6.13 (s, 1H), 4.47 (s, 2H), 4.06 (t, *J* = 6.5 Hz, 4H), 3.89 (s, 6H), 3.59 (t, *J* = 6.3 Hz, 4H), 1.86 (p, *J* = 6.5 Hz, 4H).

^13^C NMR (201 MHz, DMSO-*d6*) δ 183.06, 153.48, 146.54, 138.87, 131.90, 124.76, 124.66, 117.24, 112.03, 101.92, 70.65, 57.65, 56.31, 33.18.

R_f_(ethyl acetate): 0.39, yield 74.7% light orange amorphous solid.

(1E,4Z,6E)-5-hydroxy-1,7-bis(3,4,5-trimethoxyphenyl)-hepta-1,4,6-trien-3-one (**6b**) ESI [M + H]^+^ *m*/*z* 457.10, UV-Vis λ_max_ = 410 nm, ε = 45,517 dm^3^·mol^−1^·cm^−1^, MP = 183–184 °C,

^1^H NMR (800 MHz, CDCl_3_) δ 15.91 (s, 1H), 7.58 (d, *J* = 15.7 Hz, 2H), 6.79 (s, 4H), 6.53 (d, *J* = 15.7 Hz, 2H), 5.86 (s, 1H), 3.91 (s, 12H), 3.89 (s, 6H).

^13^C NMR (201 MHz, CDCl_3_) δ 183.11, 153.48, 140.60, 140.14, 130.52, 123.40, 105.33, 101.53, 61.01, 56.20.

R_f_(hexane/ethyl acetate 2:1): 0.4, yield 54.8% orange amorphous solid.

(1E,4Z,6E)-5-hydroxy-1,7-bis(4-hydroxy-3,5-dimethoxyphenyl)-hepta-1,4,6-trien-3-one (**7b**) ESI [M + H]^+^*m*/*z* 429.10, UV-Vis λ_max_ = 426 nm, ε = 58,833 dm^3^·mol^−1^·cm^−1^, M.p. = 115–116 °C,

^1^H NMR (800 MHz, DMSO-*d6*) δ 16.36 (s, 1H), 9.02 (s, 3H), 7.56 (d, *J* = 15.7 Hz, 2H), 7.04 (s, 4H), 6.80 (d, *J* = 15.8 Hz, 2H), 6.08 (s, 1H), 3.82 (s, 12H).

^13^C NMR (201 MHz, DMSO-*d6*) δ 183.15, 148.11, 141.03, 138.40, 125.12, 121.49, 106.28, 100.75, 56.09.

R_f_(hexane/ethyl acetate 1:1): 0.19, yield 48.0% black-purple amorphous solid.

(1E,4Z,6E)-5-hydroxy-1,7-bis(2,4,6-trimethoxyphenyl)-hepta-1,4,6-trien-3-one (**8b**) ESI [M + H]^+^*m*/*z* 457.10, UV-Vis λ_max_ = 431 nm, ε = 58,515 dm^3^·mol^−1^·cm^−1^, M.p. = 195–196 °C,

^1^H NMR (800 MHz, CDCl_3_) δ 16.41 (s, 1H), 8.05 (d, *J* = 16.1 Hz, 2H), 6.99 (d, *J* = 16.1 Hz, 2H), 6.12 (s, 4H), 5.78 (s, 1H), 3.88 (s, 12H), 3.85 (s, 6H).

^13^C NMR (201 MHz, CDCl_3_) δ 184.74, 162.53, 161.21, 130.94, 124.47, 106.75, 101.55, 90.55, 55.74, 55.38.

R_f_(hexane/ethyl acetate 2:1): 0.33, yield 57.0% orange amorphous solid.

(1E,4Z,6E)-5-hydroxy-1,7-bis(3,5-dimethoxyphenyl)-hepta-1,4,6-trien-3-one (**9b**) ESI [M + H]^+^*m*/*z* 397.10, UV-Vis λ_max_ = 394 nm, ε = 41,030 dm^3^·mol^−1^·cm^−1^, M.p. = 143–145 °C,

^1^H NMR (800 MHz, CDCl_3_) δ 15.82 (s, 1H), 7.58 (d, *J* = 15.8 Hz, 2H), 6.70 (d, *J* = 2.1 Hz, 4H), 6.59 (d, *J* = 15.8 Hz, 2H), 6.50 (t, *J* = 2.2 Hz, 2H), 5.85 (s, 1H), 3.83 (s, 12H).

^13^C NMR (201 MHz, CDCl_3_) δ 183.20, 161.05, 140.65, 136.88, 124.58, 106.04, 102.43, 101.80, 55.45.

R_f_(hexane/ethyl acetate 2:1): 0.73, yield 87.4% yellow amorphous solid.

(1E,4Z,6E)-5-hydroxy-1,7-bis(2,4-dimethoxyphenyl)-hepta-1,4,6-trien-3-one (**10b**) ESI [M + H]^+^*m*/*z* 397.00, UV-Vis λ_max_ = 425 nm, ε = 51,918 dm^3^·mol^−1^·cm^−1^, M.p. = 147–148 °C,

^1^H NMR (800 MHz, DMSO-*d6*) δ 16.41 (s, 1H), 7.80 (d, *J* = 16.0 Hz, 2H), 7.67 (d, *J* = 8.6 Hz, 2H), 6.78 (d, *J* = 15.9 Hz, 2H), 6.63 (d, *J* = 2.4 Hz, 2H), 6.61 (dd, *J* = 8.6, 2.4 Hz, 2H), 6.00 (s, 1H), 3.89 (s, 6H), 3.83 (s, 6H).

^13^C NMR (201 MHz, DMSO-*d6*) δ 183.33, 162.71, 159.52, 134.74, 129.79, 121.65, 115.99, 106.37, 101.32, 98.37, 55.77, 55.51.

R_f_(hexane/ethyl acetate 2:1): 0.57, yield 58.8% dark orange amorphous solid.

### 3.3. HPLC Purity and Chromatographic Conditions for Determining the Purity of the Tested Compounds

All analyses were performed using an Agilent 1260 Infinity II LC System coupled to a diode array detector (DAD WR, model G7115A) (Agilent Technologies, Böblingen, Germany). Chromatographic separation was conducted at 25 °C in gradient mode, using a reverse phase column (Luna^®^ C18(2), 100 Å, 150 × 4.6 mm ID, 5 µm; Phenomenex, Torrance, CA, USA) as the stationary phase, at a flow rate of 1.0 mL/min. The mobile phase consisted of 0.1% formic acid in water (Solvent A) and acetonitrile (Solvent B). The gradient elution program was as follows: 0–10.0 min (90–70% B), 10.0–13.0 min (70–50% B), and 13.0–15.0 min (50–90% B). Acetonitrile was used as a wash solvent between injections. For each analysis, 10.0 μL of sample was injected using an autosampler maintained at 25 °C. The total run time was 15.0 min per sample. The DAD detection wavelength was individually adjusted to the absorption maximum of each compound. The gradient conditions are reported in Table 4. The determined purity of all new compounds exceeded the value of 95.0% (Table S8 of Supplementary Materials).

### 3.4. Single Crystal X-Ray Diffraction Studies

Crystals of (1E,4Z,6E)-5-((difluoroboranyl)oxy)-1,7-bis(2,4,6-trimethoxyphenyl)hepta-1,4,6-trien-3-one were grown from DMF by slow evaporation technique. Reflection intensities were collected with Oxford Diffraction Xcalibur diffractometer using graphite-monochromated MoKα radiation, at 100,0(1)K. Data were processed with the Agilent Technologies CrysAlis Pro 1.171.43.93a software. The structures were solved by direct methods (Olex2 [22]) and refined by the full-matrix least-squares techniques based on F^2^ with SHELXL [23]. All non-H atoms were refined anisotropically. *Hydrogen atoms were* placed at *calculated positions* and refined using a riding model. Interpretation of the results has been performed using SHELXTL [23] and Mercury [24] programs. The crystal and refinement data are given in Table 2. The CIFs files have been deposited with the Cambridge Crystallographic Data Centre (www.ccdc.cam.ac.uk, deposition date 16 may 2025) CCDC 2451690.

### 3.5. Biological Studies

#### 3.5.1. MTT Assay for Measuring Cell Viability

The sensitivity of tumor and pseudo-normal cells to the studied compounds was evaluated using the MTT (3-[4,5-dimethylthiazol-2-yl]-2,5-diphenyl tetrazolium bromide) test (Sigma-Aldrich, USA). The 3500–4000 adherent or 15,000 suspension cells per well were seeded in 96-well plates according to ATCC recommendations in 100 μL DMEM or RPMI-1640 complete medium (Sigma-Aldrich, Burlington, MA, USA), and incubated for 72 h at 37 °C in a CO_2_ incubator with the studied compounds. After incubation, MTT reagents were added to the cells in accordance with the manufacturer’s recommendation and incubated for the next 4 h. Crystals of formazan were dissolved in dimethylsulfoxide, and the reaction absorbance was measured by an Absorbance Reader BioTek ELx800 (BioTek Instruments, Inc., Winooski, VT, USA). The half maximal inhibitory concentration value (IC50) was calculated by GraphPad Prism 8 software (San Diego, CA, USA) [25].

#### 3.5.2. The Fluorescent Microscopy of HCT-116 Cells

The HCT-116 cells were seeded in 24-well plates at 50,000 cells/mL and then allowed to adhere overnight. After, cells were treated for 24 h with compounds (1 μM) and doxorubicin (0.5 μM). Cells were stained with 0.2–0.5 μg/mL of Hoechst-33342 and 1 μg/mL of EtBr. The fluorescent microscope (LIM-400, LABEX INSTRUMENT Limited, NingBo, China), MTR3CCD camera, and Image View analysis software were used for HCT-116 cells examination [25]. The fluorescence of EtBr was measured with the microplate reader (Varioskan LUX, Thermo Fisher Scientific Inc., Waltham, MA, USA) at 520 nm excitation and 600 nm emission wavelength. The results were presented as a percentage of the non-treated cells, which served as the control. Data were analyzed using GraphPad Prism 8 (GraphPad Software, San Diego, CA, USA). The experiments were performed in triplicate. One-way ANOVA followed by Dunnett’s test was used for statistical analysis. A *p*-value of <0.05 was considered statistically significant.

#### 3.5.3. Methyl Green Displacement Assay

An aliquot of salmon sperm DNA (485 μL; 50 μg/mL) was incubated with 15 μL of methyl green solution (1 mg/mL) at 37 °C for 1 h. Subsequently, 500 μL of the tested compounds, doxorubicin, or ethidium bromide (each at final concentrations of 1 μM and 10 μM) were added to the preformed DNA–methyl green complex. The samples were further incubated at 37 °C for 2 h in the dark. Absorbance was then recorded at 630 nm. Data were analyzed using GraphPad Prism 8 (GraphPad Software, San Diego, CA, USA). The experiments were performed in triplicate. One-way ANOVA followed by Dunnett’s test was used for statistical analysis. A *p*-value of <0.05 was considered statistically significant.

#### 3.5.4. DHE Staining of HCT-116 Cells

The HCT-116 cells were seeded in 96-well plates at 5000 cells/mL and then allowed to adhere overnight. After, cells were treated for 24 h with compounds (1 μM) and doxorubicin (0.5 μM). Cells were stained with 10 μM DHE for 30 min [21]. The fluorescence was measured with the microplate reader (Varioskan LUX, Thermo Fisher Scientific Inc., Waltham, MA, USA) at 495 nm excitation and 617 nm emission wavelength. The results were presented as a percentage of the non-treated cells, which served as the control. Data were analyzed using GraphPad Prism 8 (GraphPad Software, San Diego, CA, USA). The experiments were performed in triplicate. One-way ANOVA followed by Dunnett’s test was used for statistical analysis. A *p*-value of <0.05 was considered statistically significant.

#### 3.5.5. Diphenylamine Assay

DNA fragmentation was quantified using the diphenylamine assay. HCT-116 cells were exposed for 72 h to the tested compounds at concentrations of 1 µM and doxorubicin at 0.5 µM. Following treatment, cells were lysed in 0.5 mL of Tris–EDTA buffer (pH 7.4) containing 0.2% Triton X-100 and centrifuged at 12,000× *g* for 10 min at 4 °C. The supernatant containing fragmented DNA (fraction “B”) was transferred to a separate tube, while the pellet containing intact chromatin was designated as fraction “A”. Both fractions received 0.5 mL of 25% trichloroacetic acid (Sfera Sim, Lviv, Ukraine), were mixed, and incubated for 1 h at 56 °C, followed by centrifugation at 14,000× *g* for 10 min at 4 °C. The resulting pellets (“A” and “B”) were treated with 1 mL of freshly prepared diphenylamine reagent (150 mg diphenylamine [Sigma-Aldrich] dissolved in 10 mL glacial acetic acid, 150 mL concentrated H_2_SO_4_, and 50 mL acetaldehyde solution, all from SferaSim, Lviv, Ukraine) and incubated overnight at 37 °C [26].

Absorbance was recorded at 630 nm using a BioTek ELx800 Absorbance Reader (BioTek Instruments, Winooski, VT, USA). DNA fragmentation (%) was calculated as: OD(B)/[OD(A) + OD(B)] × 100%. Data were analyzed using GraphPad Prism 8 (GraphPad Software, San Diego, CA, USA). The experiments were performed in triplicate. One-way ANOVA followed by Dunnett’s test was used for statistical analysis. A *p*-value of <0.05 was considered statistically significant.

## 4. Conclusions

This work presents a series of BF_2_, bromine, and methoxy-modified curcumin derivatives, which were subjected to physicochemical and biological studies. The cytotoxicity tests performed on the series of compounds allowed us to select the most active molecules. It was demonstrated that the cytotoxic activities of the obtained derivatives could be increased by inserting the BF_2_ moiety into their chemical structures. In general, the presence of the BF_2_ moiety is crucial for increased cytotoxic activity. Bromine substituent influence is specific to the cell line. For high activity, the preferred configuration is “isocurcumin” with a free hydroxyl group and bromine in the meta position and a methoxyl group in the para position. As such, future modifications should be focused primarily on the central part of the molecule, such as the alkyl chain or the diketone moiety. Interestingly, in the case of derivatives **2a**/**2b** and **5a**/**5b**, no significant increase in activity was observed after the introduction of this moiety, which may suggest active metabolites in potential therapeutic applications. It is also worth noting that none of the derivatives obtained show significantly higher cytotoxicity towards normal cells compared to natural curcumin. In general, compounds **2a**, **6a**, and **9a** show the best activity, which makes those interesting lead compounds for further research. Compounds **Cur**, **5b**, **6a**, **6b**, **Cur-BF_2_**, and **9a** interact with DNA through intercalation. These compounds elevated cellular ROS in treated HCT-116 cells.

## Figures and Tables

**Table 1:** The structure of the synthetized aldehydes (**11**–**19**) and curcuminoid compounds (**1a**–**10b**, **Cur**, **Cur-BF_2_**, **Iso-Cur**) is described by the type and position of substituents.

Compounds	Aromatic Ring Substituents (Positions)				
	R^1^ (pos. 2)	R^2^ (pos. 3)	R^3^ (pos. 4)	R^4^ (pos. 5)	R^5^ (pos.6)
**11**	-H	-H	-OH	-OCH_3_	-H
**12**	-H	-H	-OCH_3_	-OH	-H
**13**	-H	-Br	-OH	-OCH_3_	-H
**14**	-Br	-H	-OCH_3_	-OH	-H
**15**	-H	-OCH_3_	-OCH_3_	-OCH_3_	-H
**16**	-H	-OCH_3_	-OH	-OCH_3_	-H
**17**	-H	-Br	-OCH_3_	-OCH_3_	-H
**18**	-H	-Br	-O(CH_2_)_2_OH	-OCH_3_	-H
**19**	-H	-Br	-O(CH_2_)_3_OH	-OCH_3_	-H
**1a, 1b**	-H	-Br	-OH	-OCH_3_	-H
**2a, 2b**	-H	-Br	-O(CH_2_)_2_OH	-OCH_3_	-H
**3a, 3b**	-H	-Br	-OCH_3_	-OCH_3_	-H
**4a, 4b**	-Br	-H	-OCH_3_	-OH	-H
**5a, 5b**	-H	-Br	-O(CH_2_)_3_OH	-OCH_3_	-H
**6a, 6b**	-H	-OCH_3_	-OCH_3_	-OCH_3_	-H
**7a, 7b**	-H	-OCH_3_	-OH	-OCH_3_	-H
**8a, 8b**	-OCH_3_	-H	-OCH_3_	-H	-OCH_3_
**9a, 9b**	-H	-OCH_3_	-H	-OCH_3_	-H
**10a, 10b**	-OCH_3_	-H	-OCH_3_	-H	-H
**Cur-BF_2_, Cur**	-H	-H	-OH	-OCH_3_	-H
**Iso-Cur**	-H	-OH	-OCH_3_	-H	-H

**Table 2:** Experimental details for compound **8a**.

Crystal Data	
Chemical formula	C_25_H_27_BF_2_O_8_
*M*_r_	504.27
Crystal system, space group	Monoclinic, P2_1_/c
Temperature (K)	100.0(1)
*a*, *b*, *c* (Å)	15.8025(3), 7.6000(1), 20.1625(3)
α, β, γ (°)	90.0, 99.795(2), 90.0
*V* (Å^3^)	2386.20(7)
*Z*	4
Radiation type	Mo *K*α
μ (mm^−1^)	0.113
Crystal size (mm)	0.4 × 0.4 × 0.3
Data collection	
Diffractometer	Xcalibur, Eos four-circle diffractometer
Absorption correction	Multi-scan *CrysAlisPro 1.171.43.93a (Rigaku* Oxford Diffraction*, 2023*). Empirical absorption correction using spherical harmonics, implemented in SCALE3 ABSPACK scaling algorithm.
*T*_min_, *T*_max_	0.972, 1.000
No. of measured, independent and observed [*I* > 2σ(*I*)] reflections	9256, 5038, 4632
*R*_int_	0.019
(sin θ/λ)_max_ (Å^−1^)	0.634
Refinement	
*R*[*F*^2^ > 2σ(*F*^2^)], *wR*(*F*^2^), *S*	0.034, 0.085, 1.046
No. of reflections	4632
No. of parameters	332
H-atom treatment	H-atom parameters constrained
Δ⟩_max_, Δ⟩_min_ (e Å^−3^)	0.25, −0.21

**Table 3:** Toxicity of compounds towards tumor and non-tumor cells (MTT data on 72 h of cell exposure, M ± SD). Values of IC_50_ below 2 µM were highlighted by bold text in green.

Compound	Parameter IC_50_ [µM]							
	Cell Line Type							
	BA/F3 wt	BA/F3 Calr del52	BA/F3 Calr ins5	K562	Jurkat	HCT-116	MDA-MB-231	Balb/3T3
**Cur-BF2**	**0.65 ± 0.09**	**0.65 ± 0.09**	**0.52 ± 0.09**	3.00 ± 0.53	**0.57 ± 0.09**	**0.62 ± 0.09**	6.09 ± 0.06	36.82 ± 0.36
**Cur**	26.36 ± 0.83	13.54 ± 1.72	28.57 ± 0.96	27.76 ± 0.06	5.08 ± 0.72	6.96 ± 0.15	27.58 ± 0.08	36.99 ± 0.85
**Iso-Cur**	5.32 ± 0.76	6.46 ± 0.57	7.13 ± 0.12	8.20 ± 0.38	6.14 ± 0.41	5.31 ± 0.57	5.05 ± 0.52	26.20 ± 3.45
**1a**	11.03 ± 0.57	21.68 ± 2.61	7.05 ± 0.72	24.22 ± 2.01	5.35 ± 0.81	4.71 ± 0.75	30.27 ± 0.69	36.19 ± 0.11
**1b**	33.90 ± 2.10	34.25 ± 1.64	37.03 ± 2.79	39.15 ± 1.97	36.91 ± 0.38	21.94 ± 0.20	30.45 ± 0.68	38.07 ± 0.35
**2a**	**0.52 ± 0.09**	**0.49 ± 0.08**	**0.48 ± 0.08**	3.84 ± 0.65	**0.66 ± 0.09**	**1.70 ± 0.21**	4.17 ± 0.55	35.30 ± 0.06
**2b**	5.69 ± 0.06	5.78 ± 0.02	5.90 ± 0.04	6.38 ± 0.11	5.87 ± 0.37	4.10 ± 0.61	5.09 ± 0.24	35.06 ± 0.54
**3a**	6.13 ± 0.07	5.84 ± 0.02	5.89 ± 0.04	5.46 ± 0.53	5.70 ± 0.66	5.73 ± 0.16	4.86 ± 0.40	36.95 ± 0.16
**3b**	>50	>50	>50	33.01 ± 0.29	37.04 ± 1.29	12.83 ± 1.54	>50	>50
**4a**	6.18 ± 0.08	5.52 ± 0.01	5.91 ± 0.04	5.97 ± 0.04	2.36 ± 0.40	4.11 ± 0.62	4.90 ± 0.30	31.93 ± 1.75
**4b**	34.43 ± 1.80	33.92 ± 1.24	22.56 ± 0.47	35.01 ± 2.05	36.80 ± 1.98	27.00 ± 0.22	27.25 ± 0.59	37.62 ± 0.18
**5a**	5.93 ± 0.05	3.53 ± 0.58	5.83 ± 0.03	5.93 ± 0.04	3.95 ± 0.70	3.51 ± 0.60	5.97 ± 0.06	36.19 ± 4.19
**5b**	29.82 ± 0.14	5.74 ± 0.02	7.51 ± 0.33	6.21 ± 0.08	6.98 ± 0.18	**0.89 ± 0.06**	6.14 ± 0.07	38.68 ± 0.03
**6a**	**0.49 ± 0.08**	**0.50 ± 0.09**	**0.52 ± 0.09**	2.97 ± 0.53	**0.53 ± 0.09**	**0.70 ± 0.09**	5.13 ± 0.49	29.70 ± 2.37
**6b**	6.43 ± 0.08	5.63 ± 0.41	4.17 ± 0.63	7.05 ± 0.71	6.16 ± 0.03	**0.62 ± 0.09**	7.89 ± 0.45	20.88 ± 3.84
**7a**	4.35 ± 0.59	5.51 ± 0.18	6.26 ± 0.07	5.80 ± 0.13	3.79 ± 0.75	2.39 ± 0.40	5.26 ± 0.33	35.04 ± 2.95
**7b**	36.59 ± 2.52	31.97 ± 1.12	34.38 ± 2.52	39.12 ± 3.24	24.73 ± 0.09	17.84 ± 0.05	32.21 ± 0.81	42.29 ± 0.94
**8a**	>50	>50	37.48 ± 3.49	>50	>50	>50	>50	>50
**8b**	>50	>50	>50	>50	>50	>50	>50	>50
**9a**	**0.49 ± 0.08**	**0.50 ± 0.09**	**0.48 ± 0.08**	**0.65 ± 0.09**	**0.58 ± 0.09**	**0.53 ± 0.09**	**0.87 ± 0.06**	40.03 ± 0.29
**9b**	22.16 ± 0.17	35.05 ± 2.38	26.69 ± 0.16	7.06 ± 0.71	7.37 ± 0.23	4.96 ± 0.40	13.79 ± 1.95	32.91 ± 5.69
**10a**	>50	>50	>50	>50	>50	>50	>50	>50
**10b**	>50	>50	48.84 ± 0.26	>50	37.14 ± 2.34	30.71 ± 0.67	>50	>50
**Doxorubicin**	0.55 ± 0.03	0.54 ± 0.01	0.55 ± 0.02	0.62 ± 0.02	0.51 ± 0.16	0.60 ± 0.03	0.72 ± 0.02	0.73 ± 0.02

**Table 4:** HPLC gradient used for the analysis of a newly synthesized compound.

Time [min]	Phase A [%]	Phase B [%]
0	10	90
10	30	70
13	50	50
15	10	90

## Data Availability

The original contributions presented in this study are included in the article/Supplementary Material. Further inquiries can be directed to the corresponding author.

## Supplementary Materials

The following supporting information can be downloaded at: https://www.mdpi.com/article/10.3390/molecules30234609/s1.
