# Supplementary material for: Promising Anticancer Activity of Novel Hydroxyethyloxy and Bromo derivatives of Curcumin and Its Complexes with BF2
Source: Molecules. 2025 Nov 30;30(23):4609. doi: 10.3390/molecules30234609 (PMC12693189; doi:10.3390/molecules30234609)

Article

Promising Anticancer Activity of Novel Hydroxyethyloxy and Bromo Derivatives of Curcumin and Its Complexes with BF_2

Eduard Potapskyi [1,2], Dawid Łażewski [1], Julian Myszkiewicz [1], Gabriela Korzańska [1], Joanna Kuźmińska [3], Łukasz Popenda [4], Artur Korzański [5], Agnieszka Zgoła-Grześkowiak [6], Agnieszka Gielara-Korzańska [1], Karolina Chmaj-Wierzchowska [7], Nataliya Finiuk [8,9], Yuliia Kozak [8,9], Iryna Ivasechko [8,9], Rostyslav Stoika [8], Roman Lesyk [9,10,11] and Marcin Wierzchowski [1,*]

1 Department of Chemical Technology of Drugs, Poznan University of Medical Sciences, Rokietnicka Street 3, 60-806 Poznan, Poland; potapskiyed@gmail.com (E.P.)
2 Doctoral School, Poznan University of Medical Sciences, Bukowska Street 70, 60-812 Poznan, Poland
3 Department of Pharmaceutical Chemistry, Poznan University of Medical Sciences, Rokietnicka Street 3, 60-806 Poznan, Poland
4 NanoBioMedical Centre, Adam Mickiewicz University, Wszechnicy Piastowskiej Street 3, 61-614 Poznan, Poland
5 Department of Chemistry, Adam Mickiewicz University, Uniwersytetu Poznanskiego Street 8, 61-614 Poznan, Poland
6 Institute of Chemistry and Technical Electrochemistry, Poznan University of Technology, Berdychowo Street 4, 60-965 Poznan, Poland
7 Department of Maternal and Child Health, Poznan University of Medical Sciences, 60-701 Poznan, Poland
8 Department of Regulation of Cell Proliferation and Apoptosis, Institute of Cell Biology of the National Academy of Sciences of Ukraine, Drahomanov Street 14/16, 79005 Lviv, Ukraine; nataliyafiniuk@gmail.com (N.F.)
9 Molecular Design Center, State Non-Commercial Enterprise, Danylo Halytsky Lviv National Medical University, Pekarska Street 69, 79010 Lviv, Ukraine; roman.lesyk@gmail.com
10 Department of Pharmaceutical, Organic and Bioorganic Chemistry, Danylo Halytsky Lviv National Medical University, Pekarska Street 69, 79010 Lviv, Ukraine
11 Department of Biotechnology and Cell Biology, Medical College, University of Information Technology and Management in Rzeszow, Sucharskiego 2, 35-225 Rzeszow, Poland
* Correspondence: mwierzch@ump.edu.pl

Supporting Information

Table of contents

1. NMR data 2
2. MS ESI spectrometry data 68
3. Crystallographic data 98
4. HPLC data 103
5. Biological activity of tested compounds 114

1. NMR data

Symbols: * - solvent signals, ~ - water signals.

Figure S1. NMR experiments of aldehyde **1**.

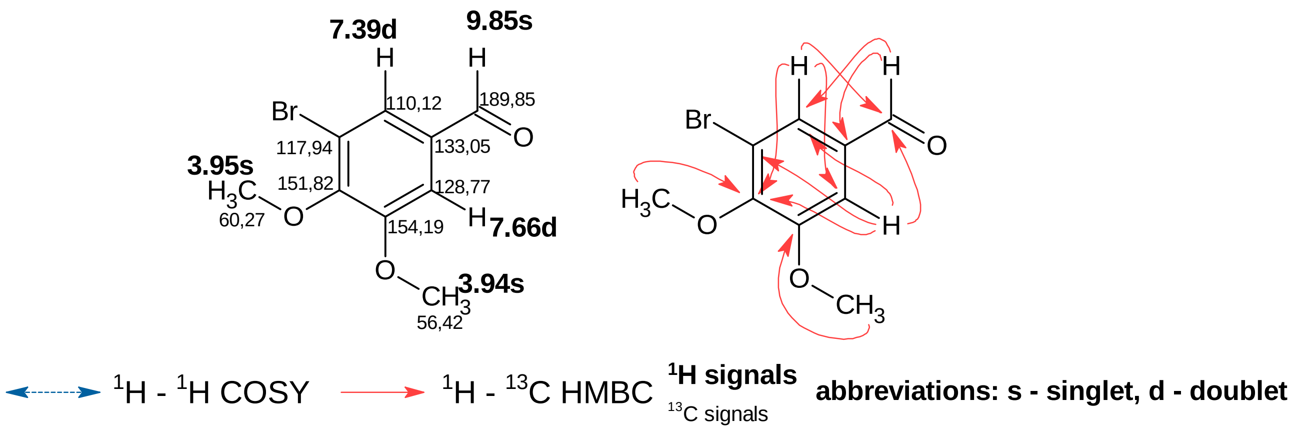

Figure S2. ^{1}H NMR of aldehyde **1**.

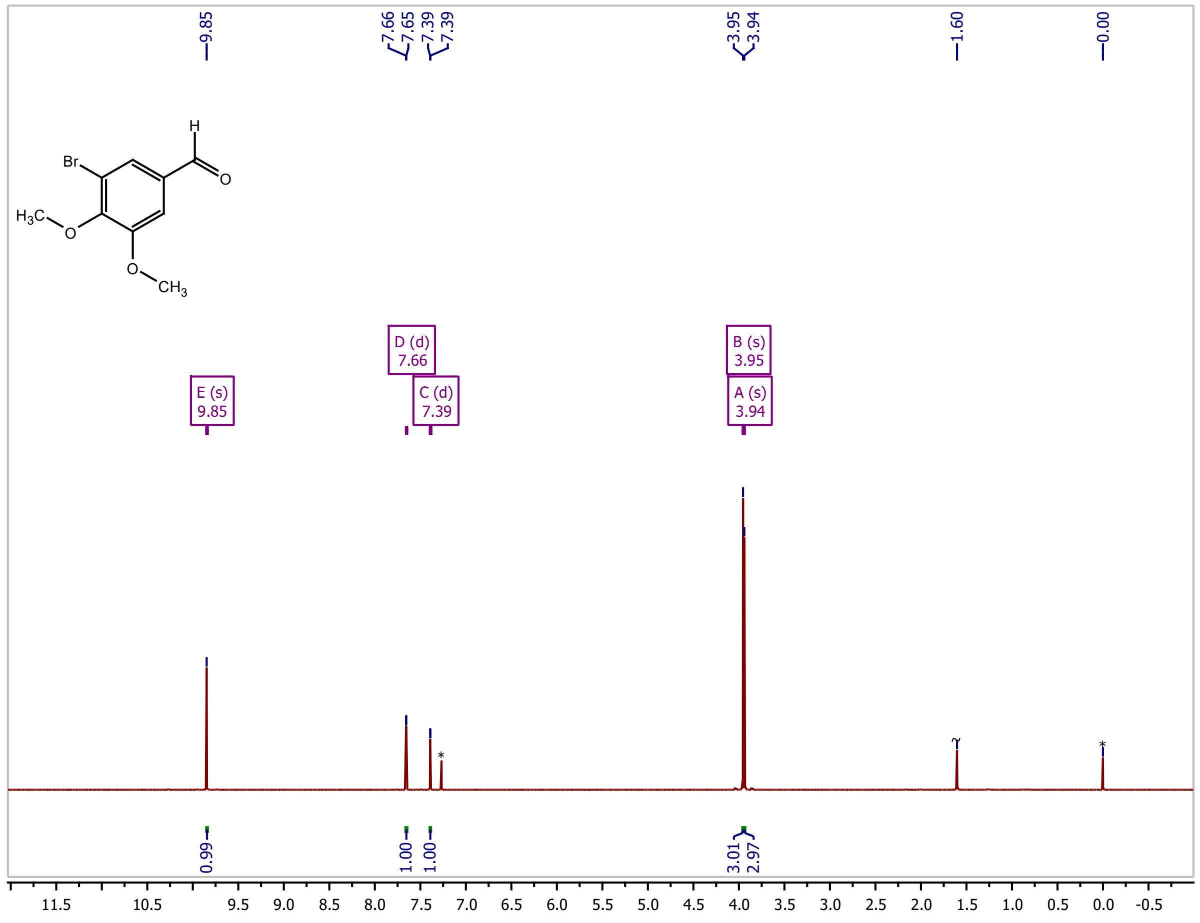

Figure S3. ^{13}C NMR of aldehyde **1**.

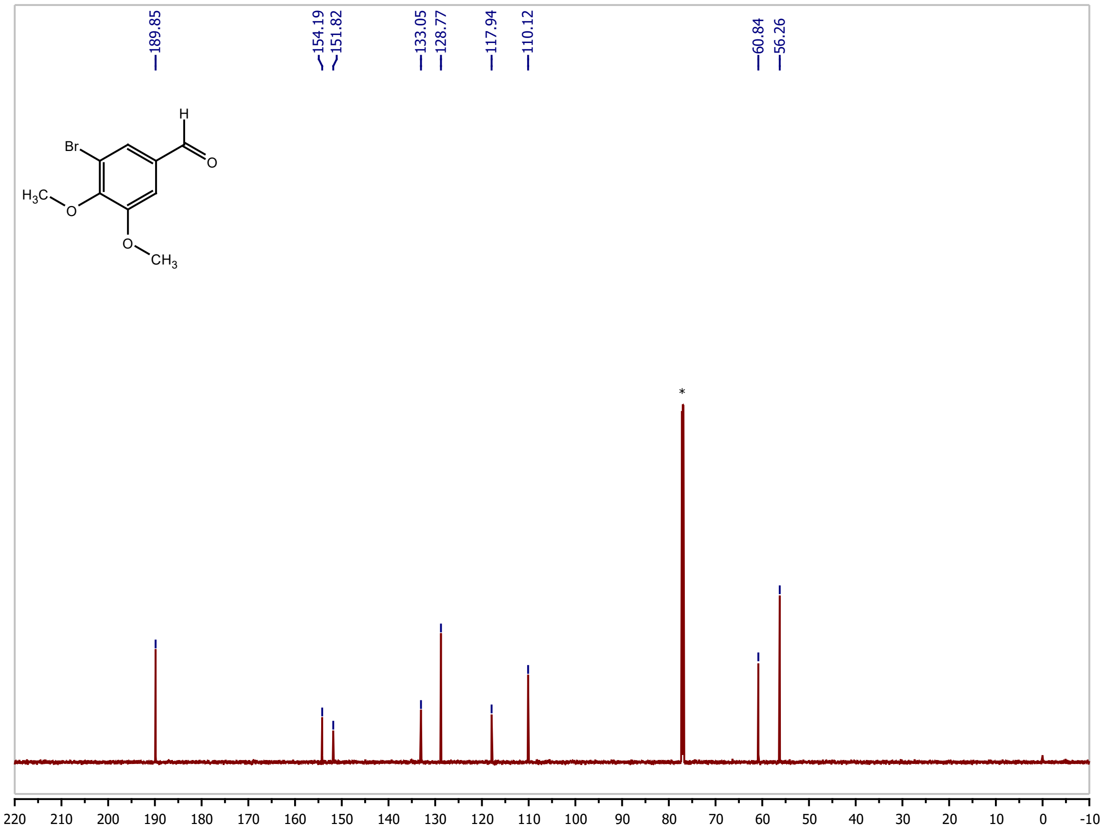

Figure S4. ^{1}H-^{1}H COSY of aldehyde **1**.

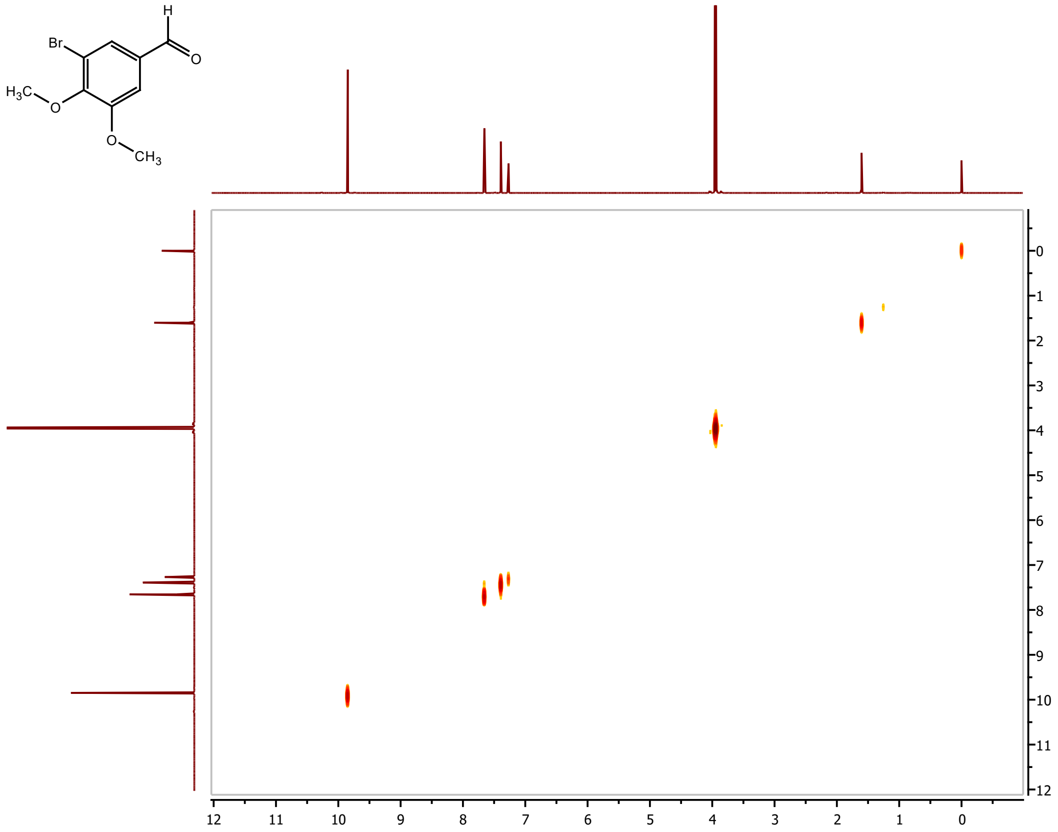

Figure S5. ^{1}H-^{13}C HSQC of aldehyde **1**.

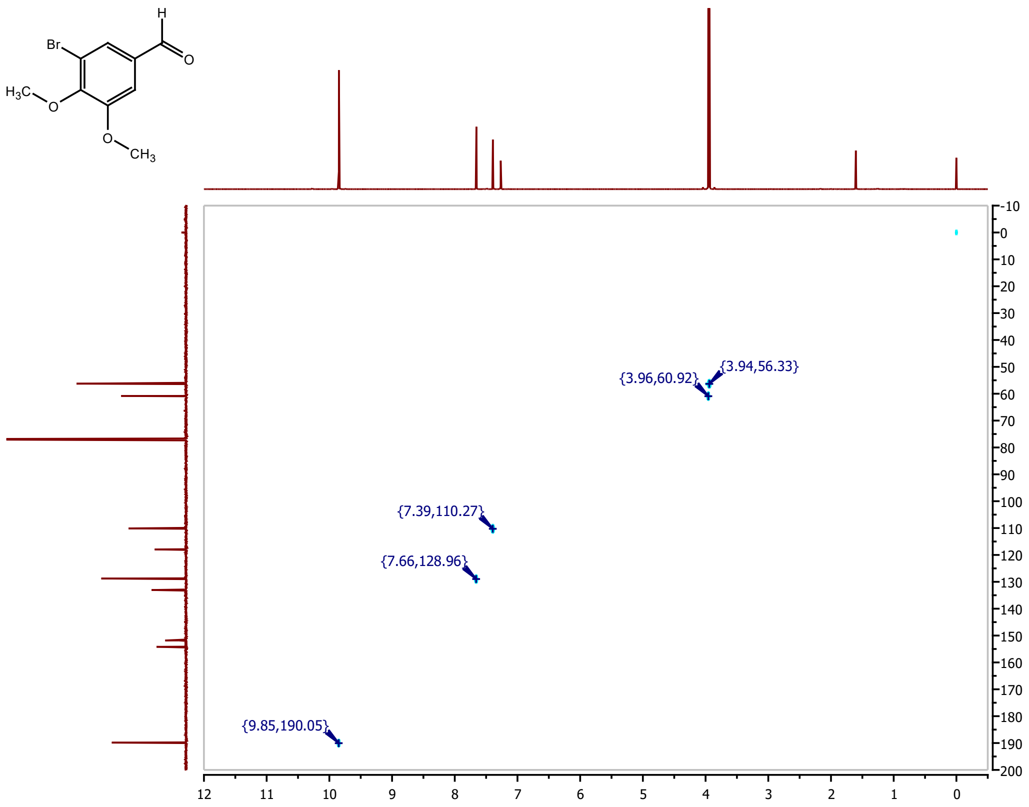

Figure S6. ^{1}H-^{13}C HMBC of aldehyde **1**.

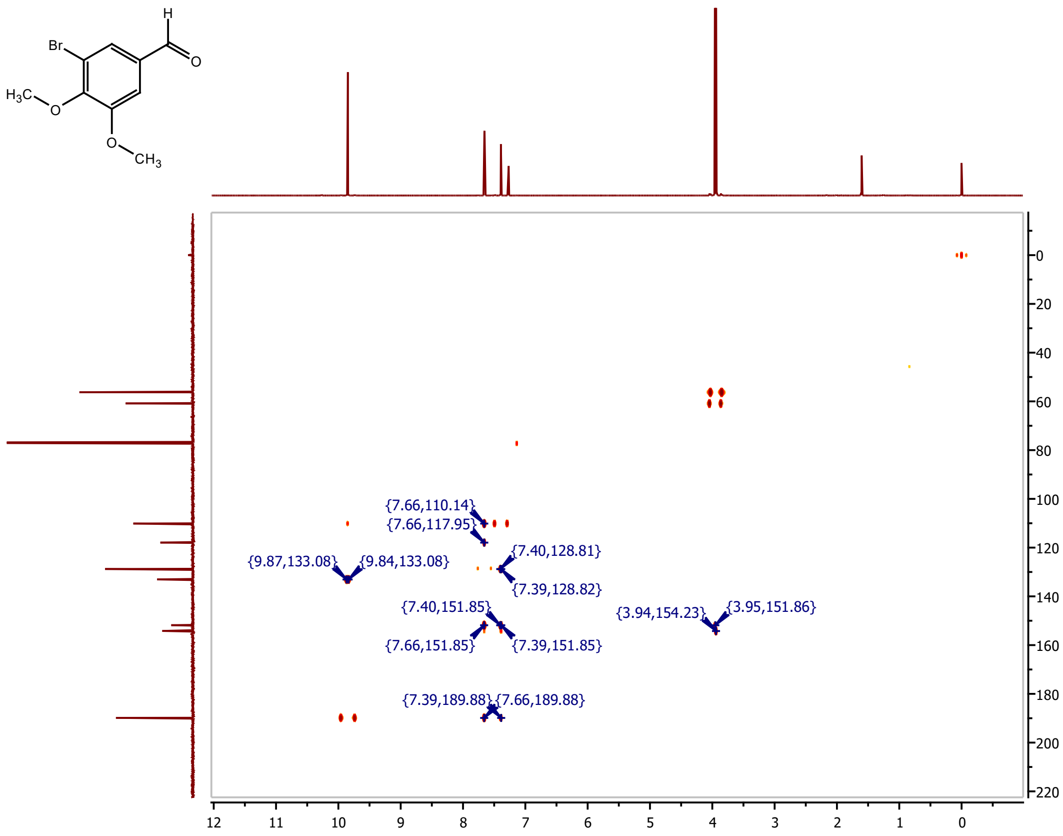

Figure S7. NMR experiments of aldehyde **2**.

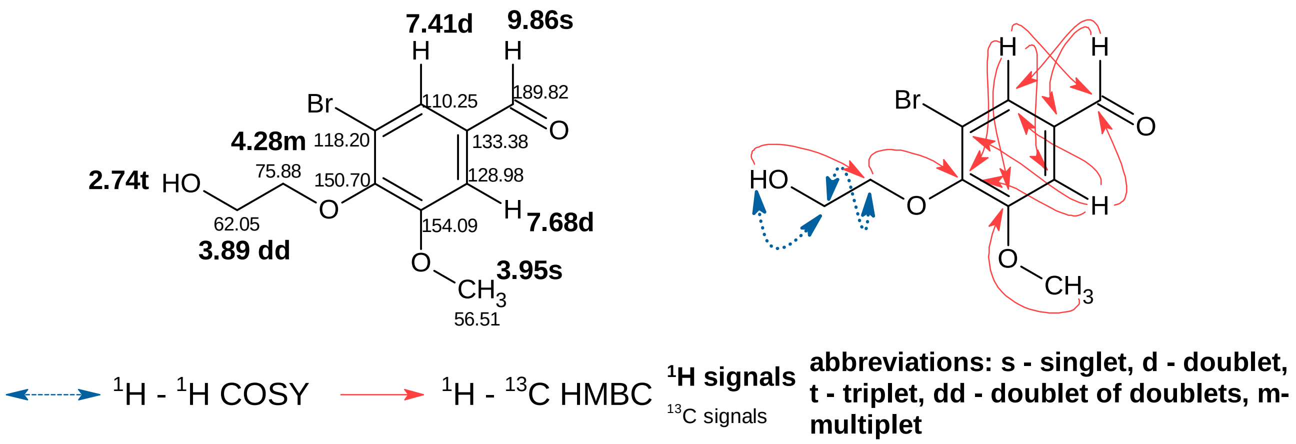

Figure S8. ^{1}H NMR of aldehyde **2**.

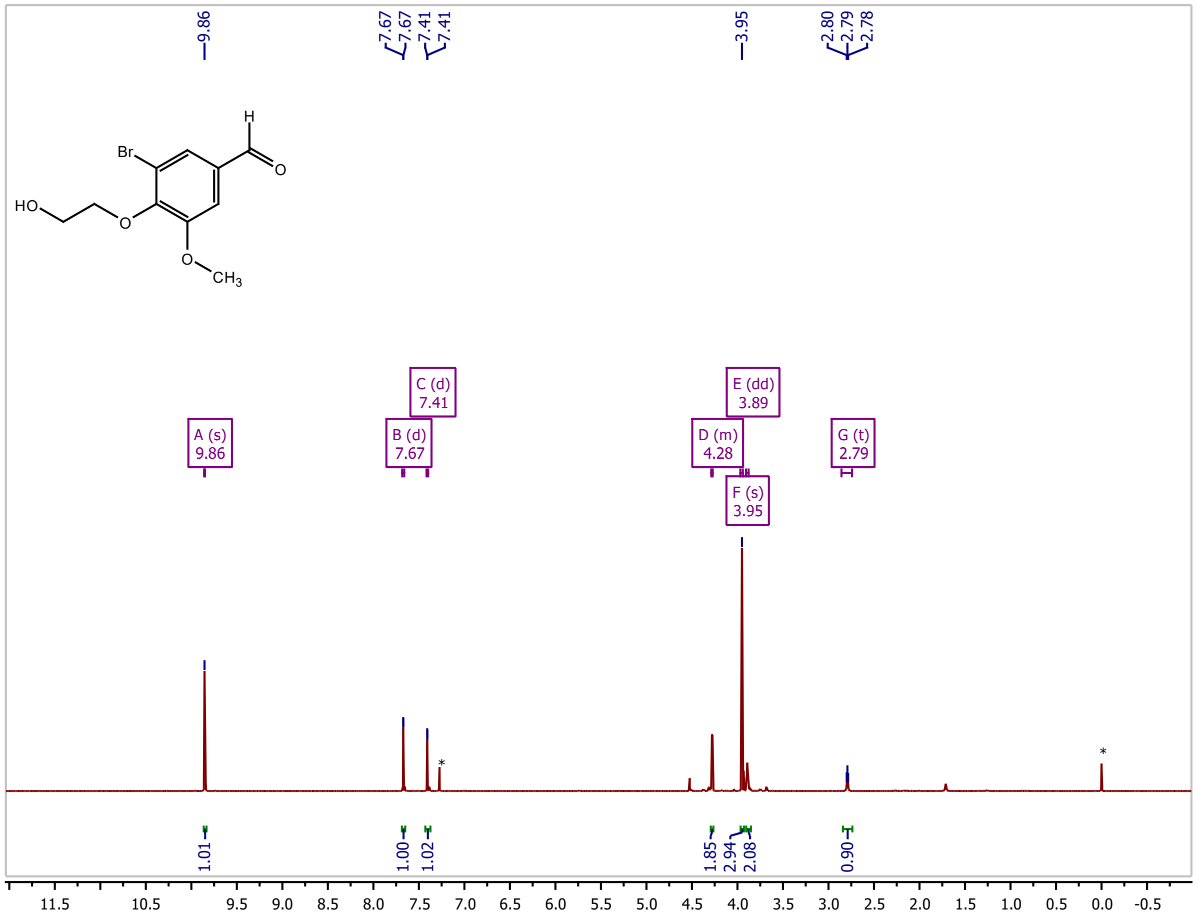

Figure S9. ^{13}C NMR of aldehyde **2**.

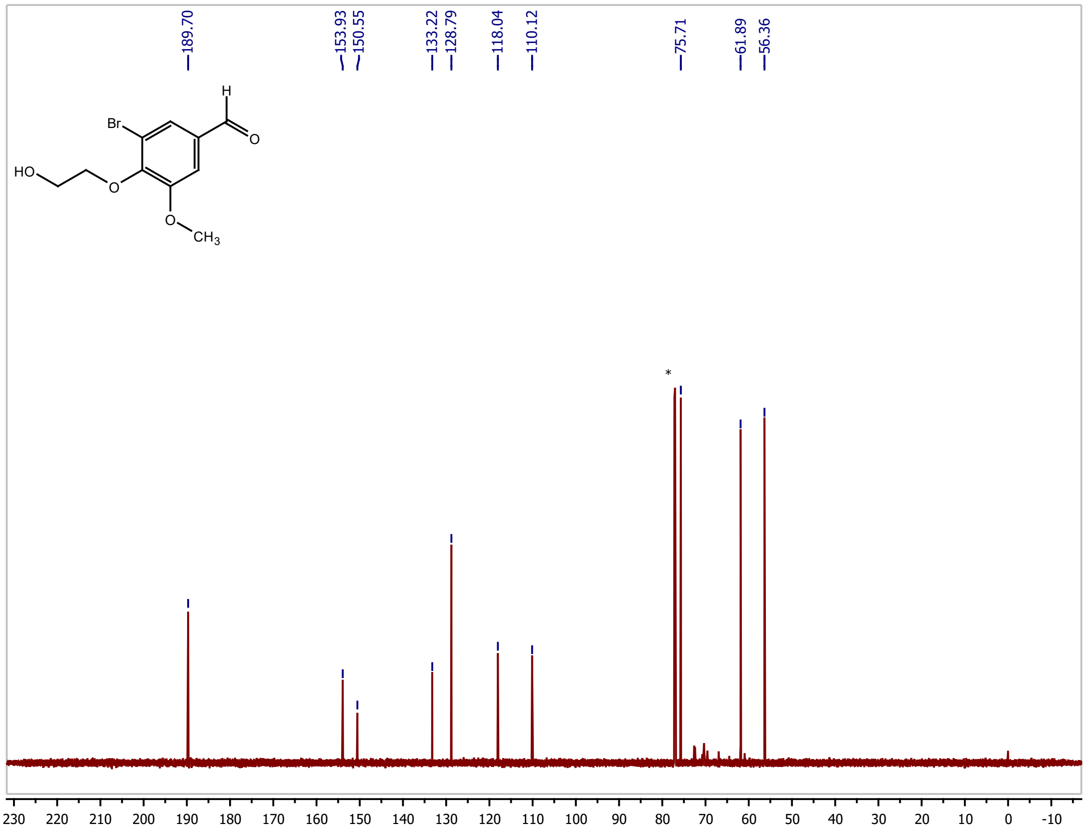

Figure S10. ^{1}H-^{1}H COSY of aldehyde **2**.

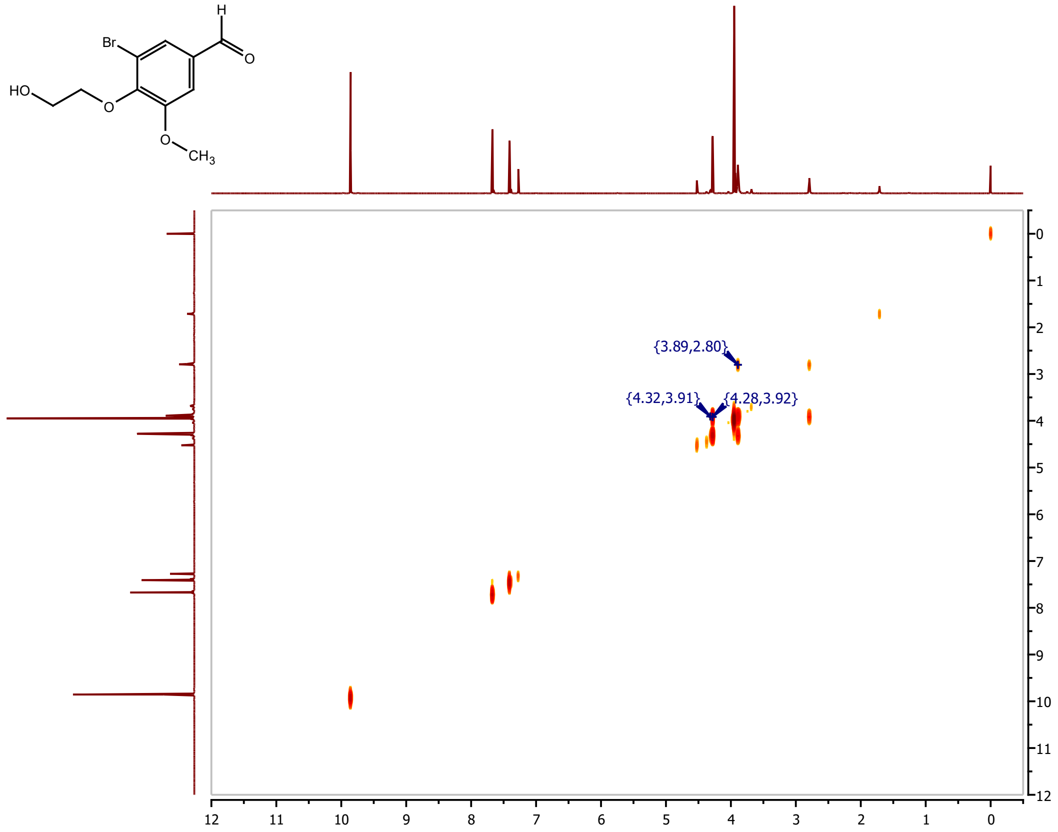

Figure S11. ^{1}H-^{13}C HSQC of aldehyde **2**.

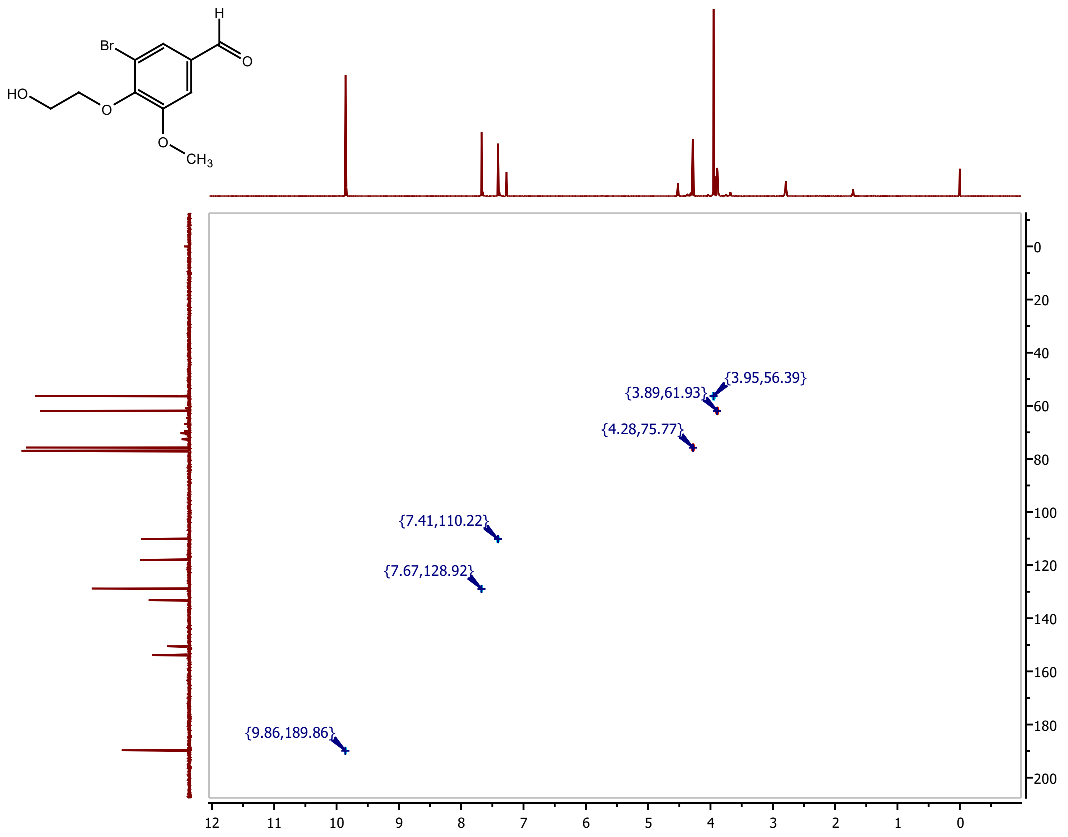

Figure S12. ^{1}H-^{13}C HMBC of aldehyde **2**.

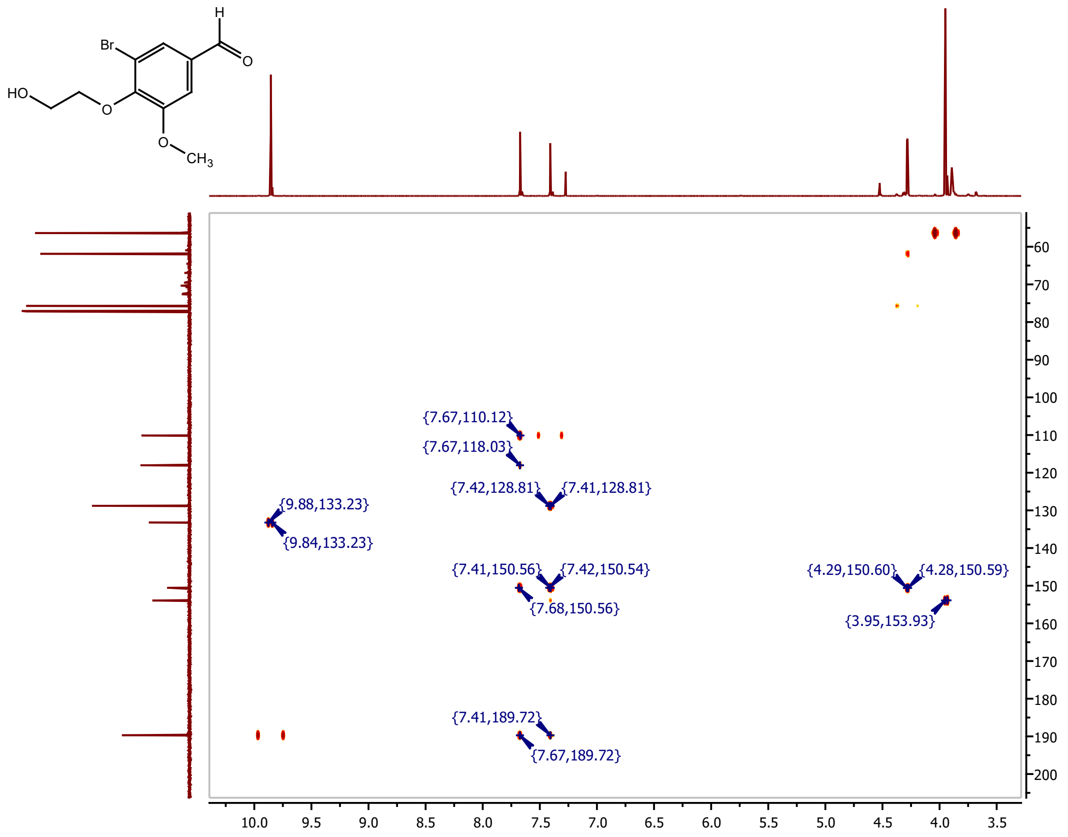

Figure S23. NMR experiments of aldehyde **3**.

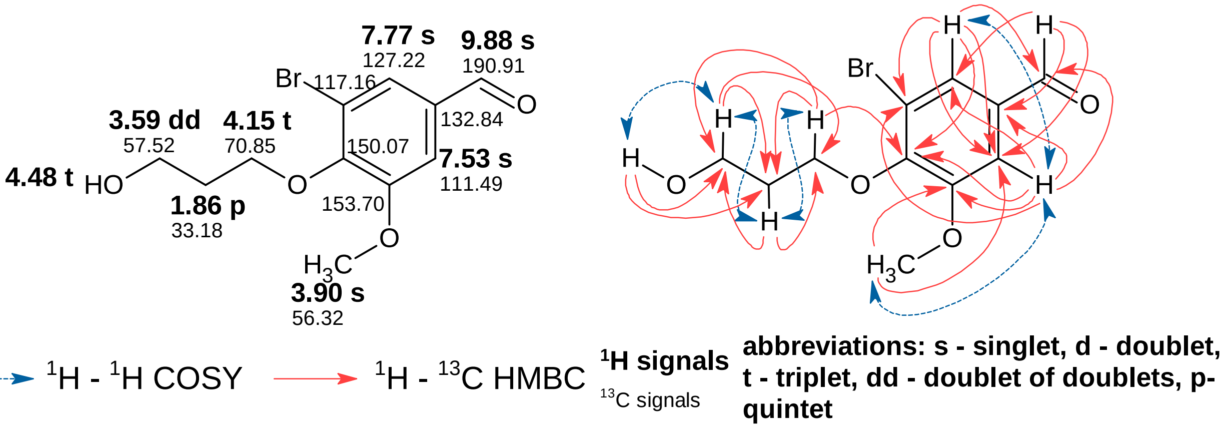

Figure S14. ^{1}H NMR of aldehyde **3**.

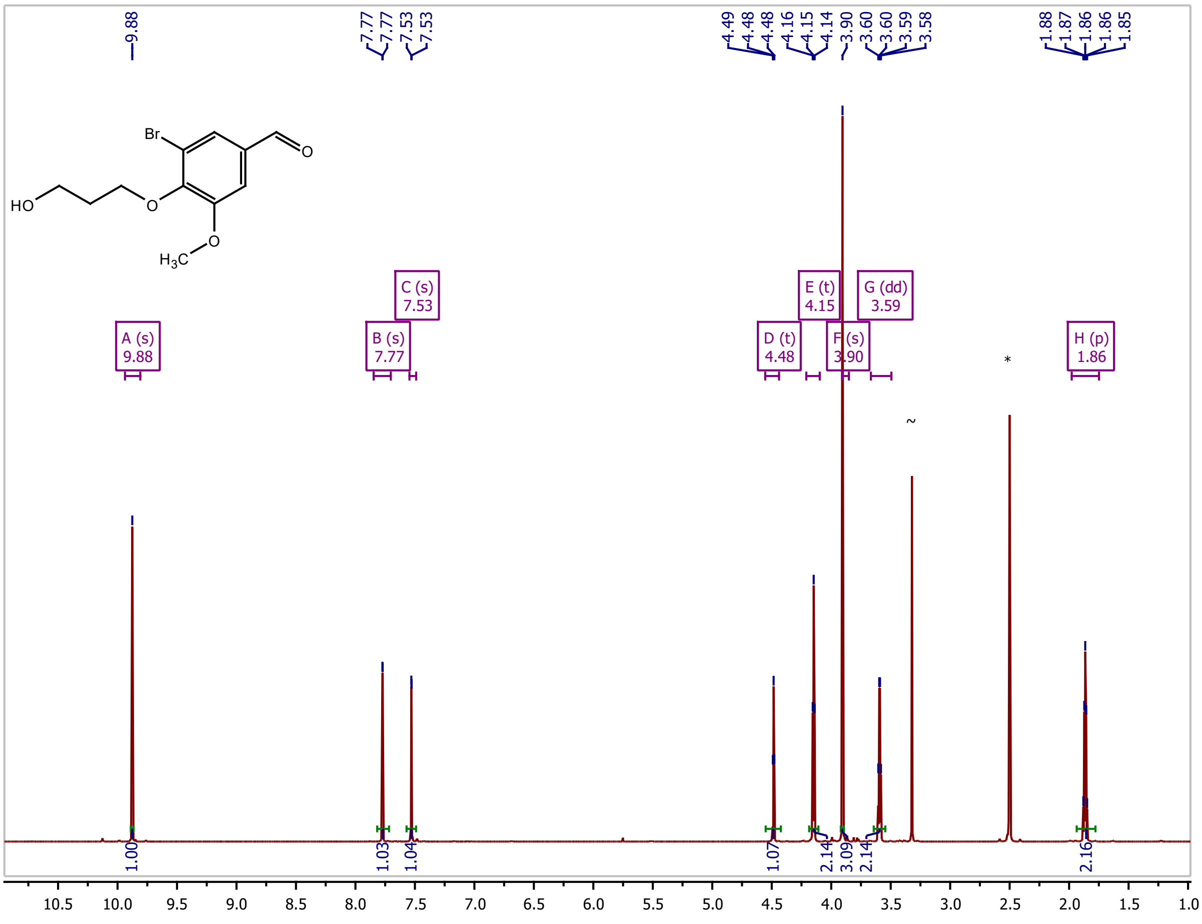

Figure S15. ^{13}C NMR of aldehyde **3**.

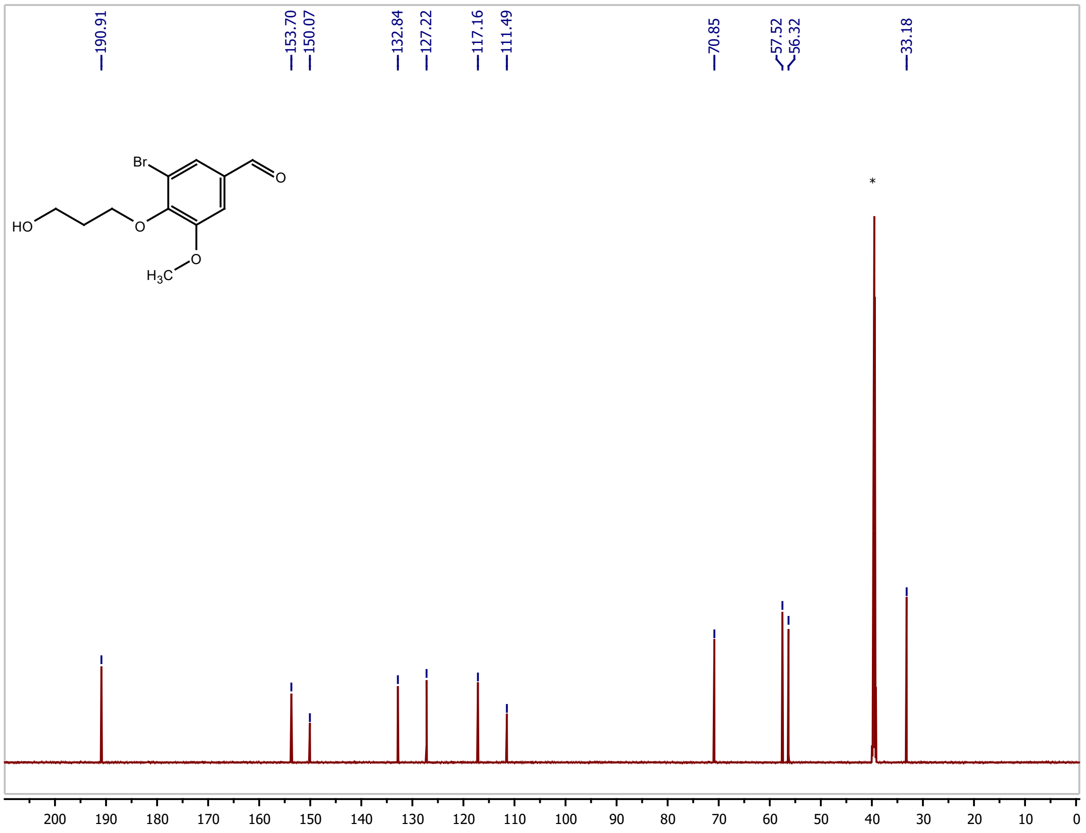

Figure S16. ^{1}H-^{1}H COSY of aldehyde **3**.

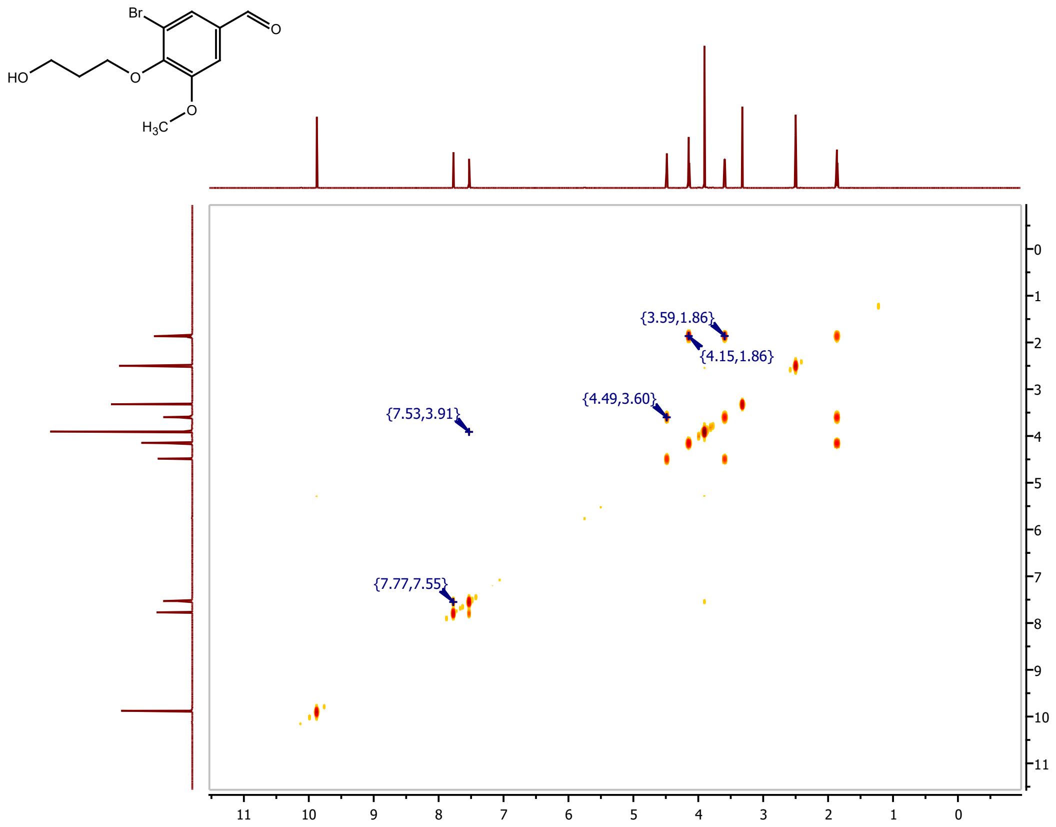

Figure S17. ^{1}H-^{13}C HSQC of aldehyde **3**.

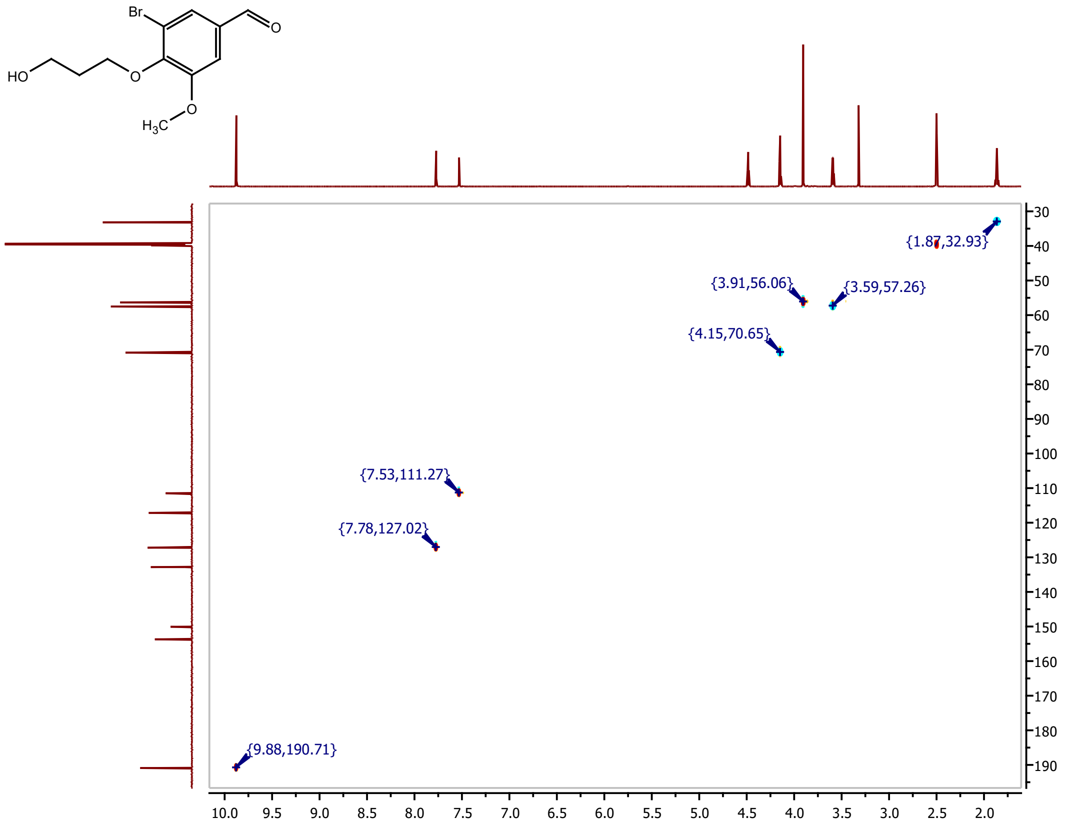

Figure S18. ^{1}H-^{13}C HMBC of aldehyde **3.**

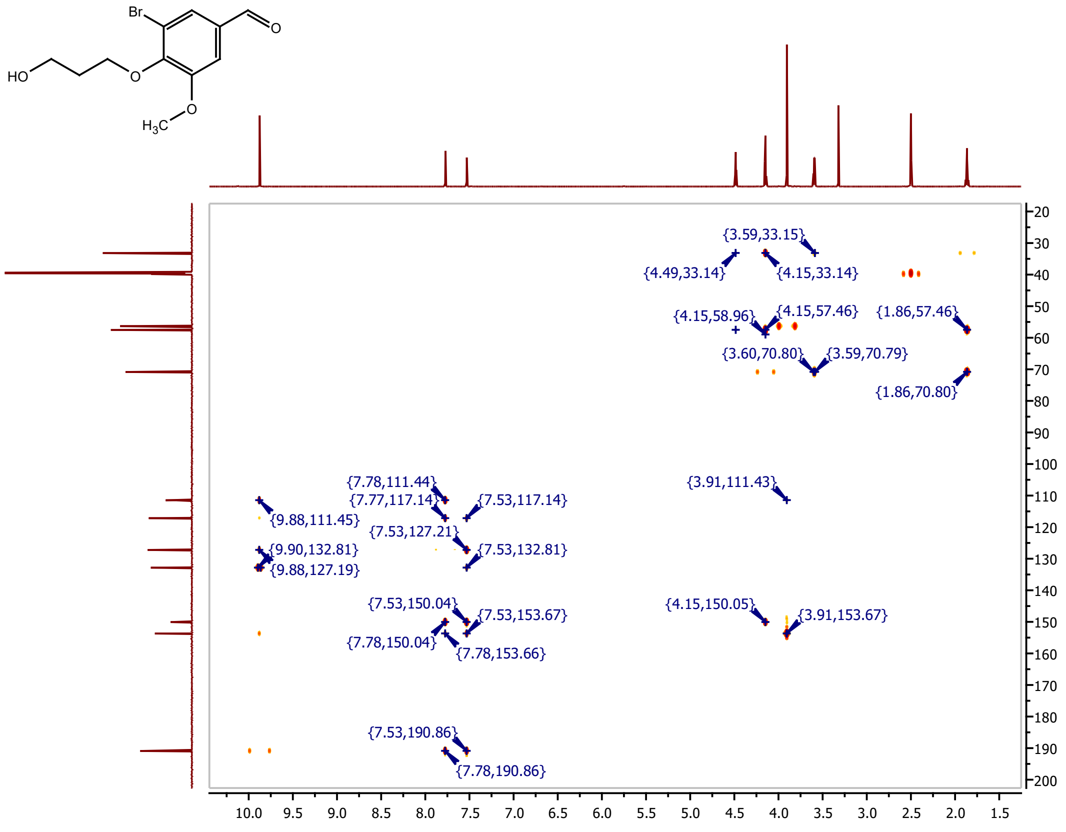

Figure S19. NMR experiments of compound **1a**

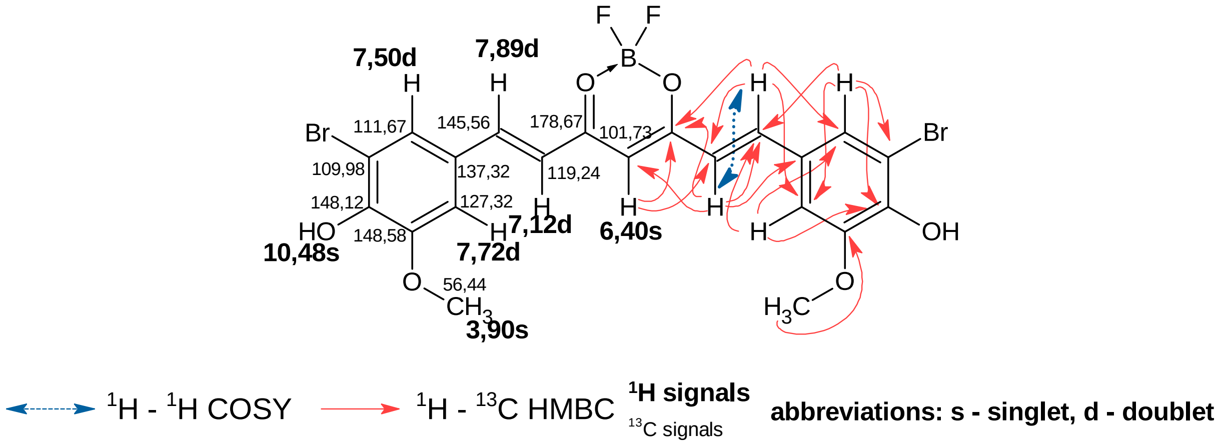

Figure S20. ¹H NMR of curcumin **1a**.

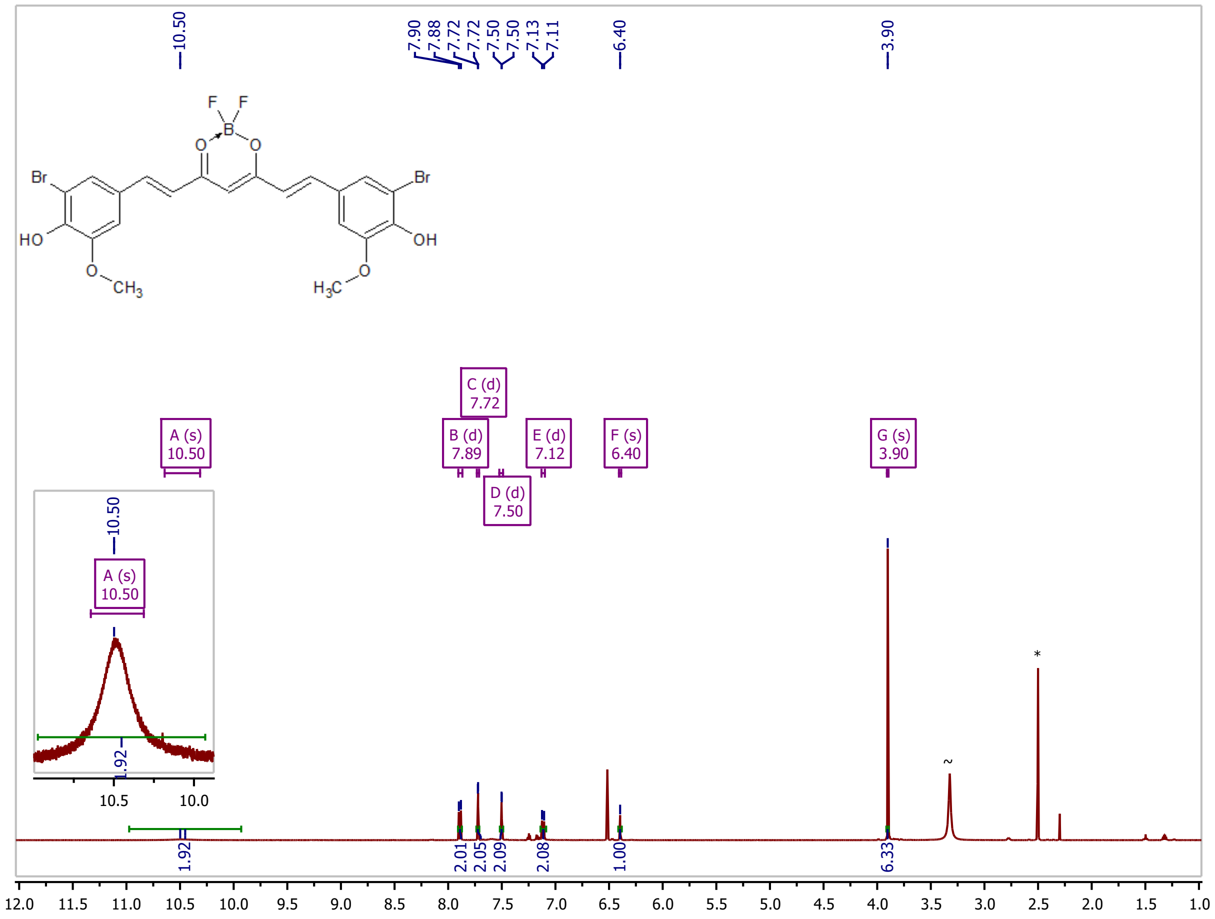

Figure S21. ^{13}C NMR of curcumin **1a**.

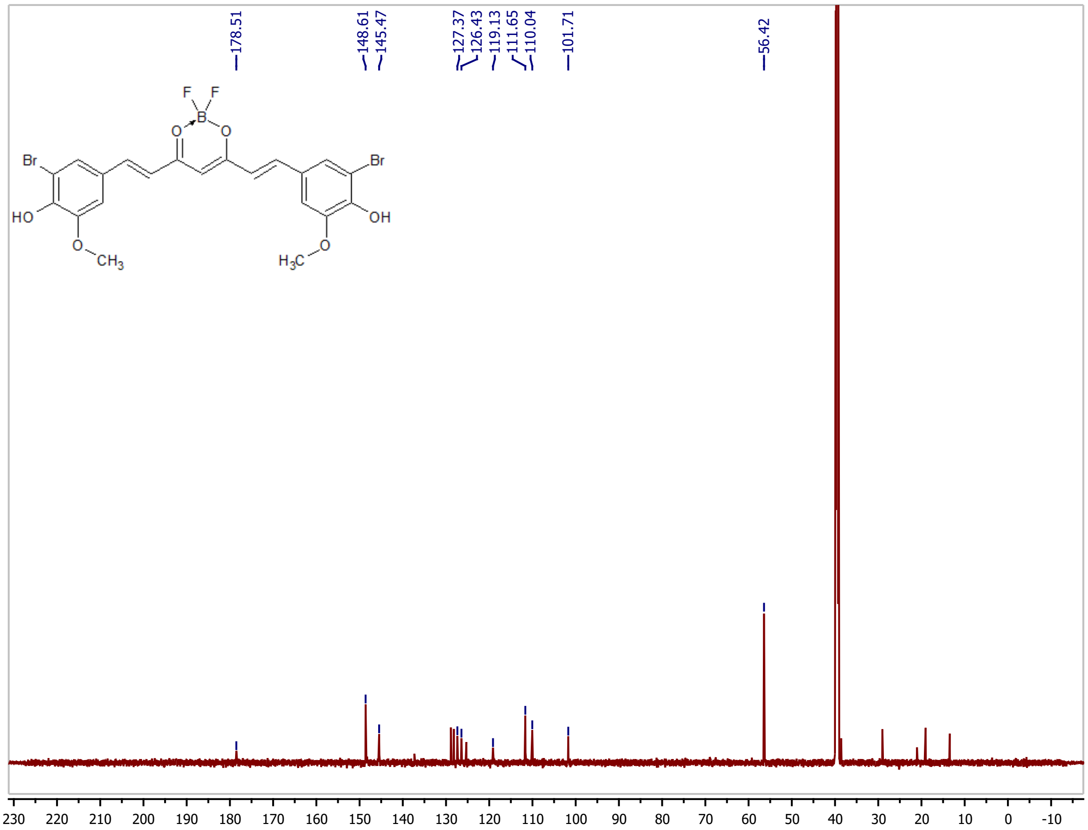

Figure S22. ^{1}H-^{1}H COSY of curcumin **1a**.

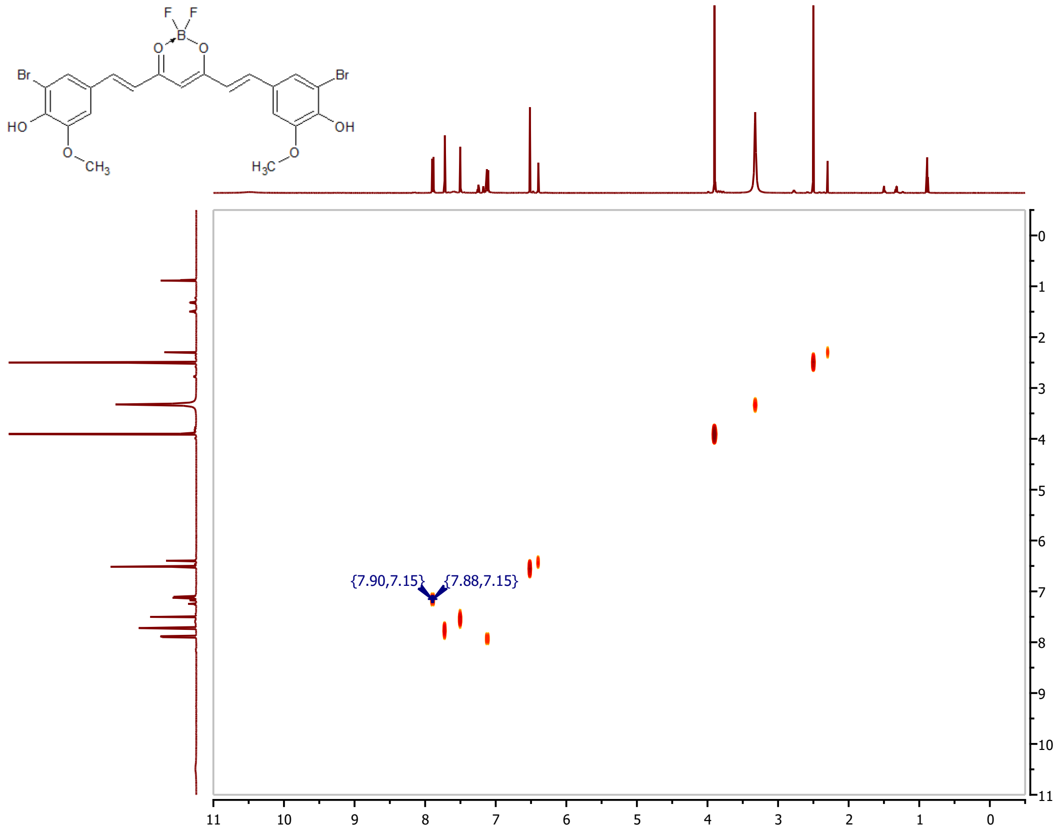

Figure S23. ^{1}H-^{13}C HSQC of curcumin **1a**.

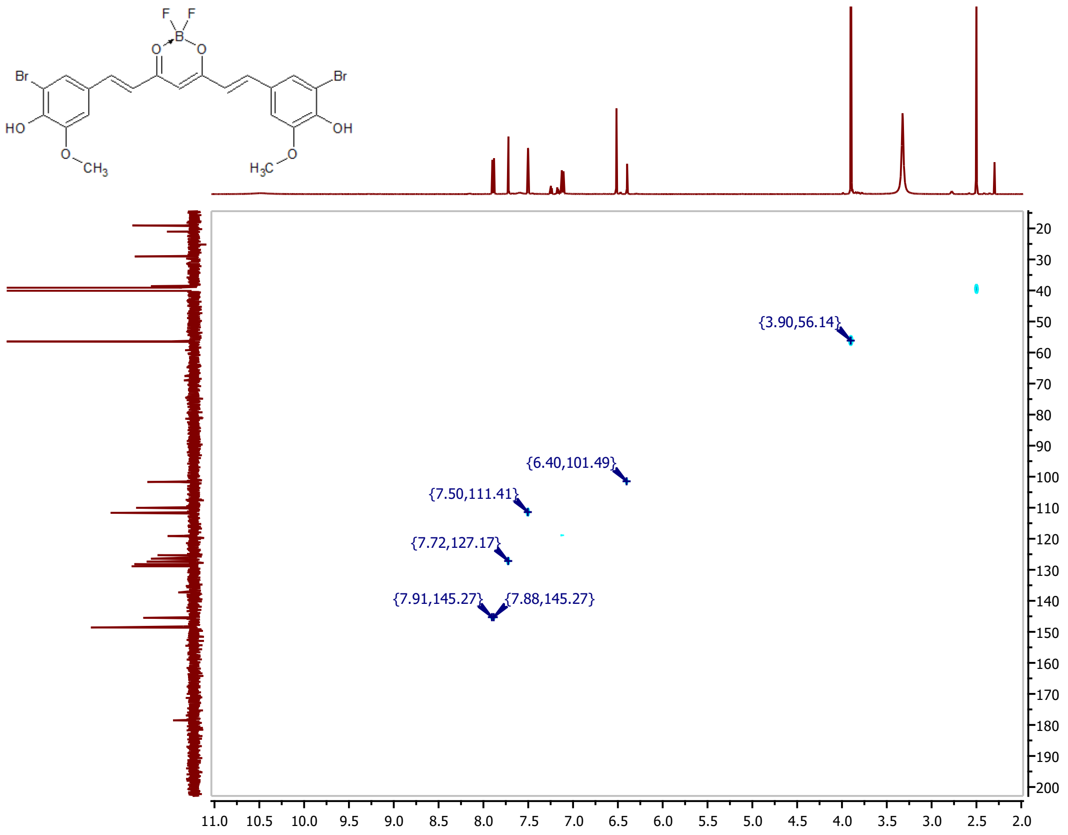

Figure S24. ^{1}H-^{13}C HMBC of curcumin **1a.**

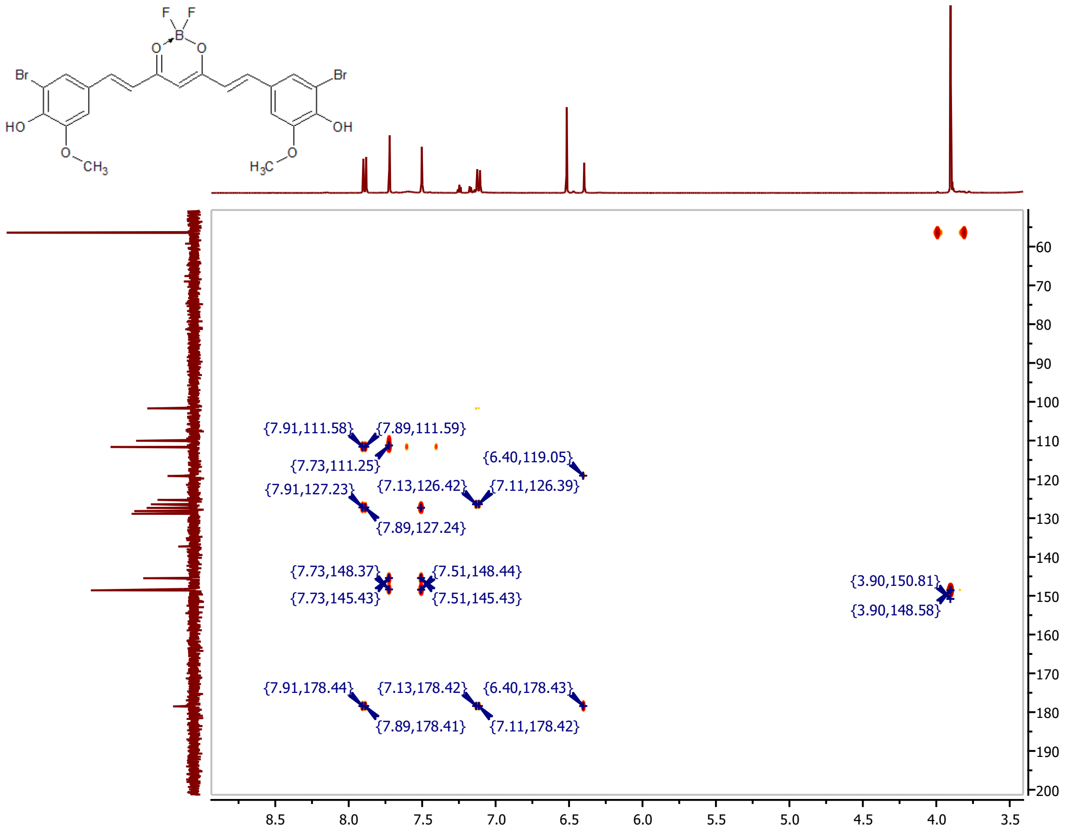

Figure S25. NMR experiments of compound **1b**.

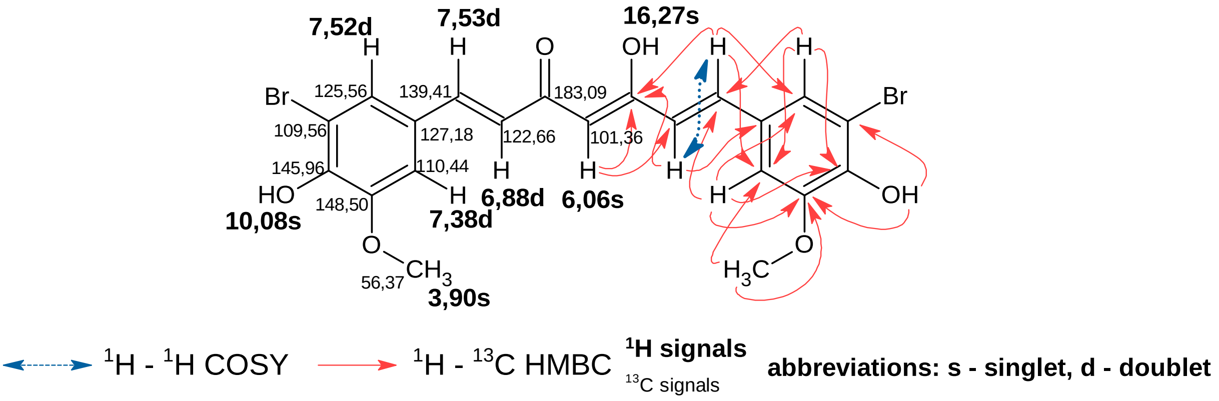

Figure S26. ¹H NMR of curcumin **1b**.

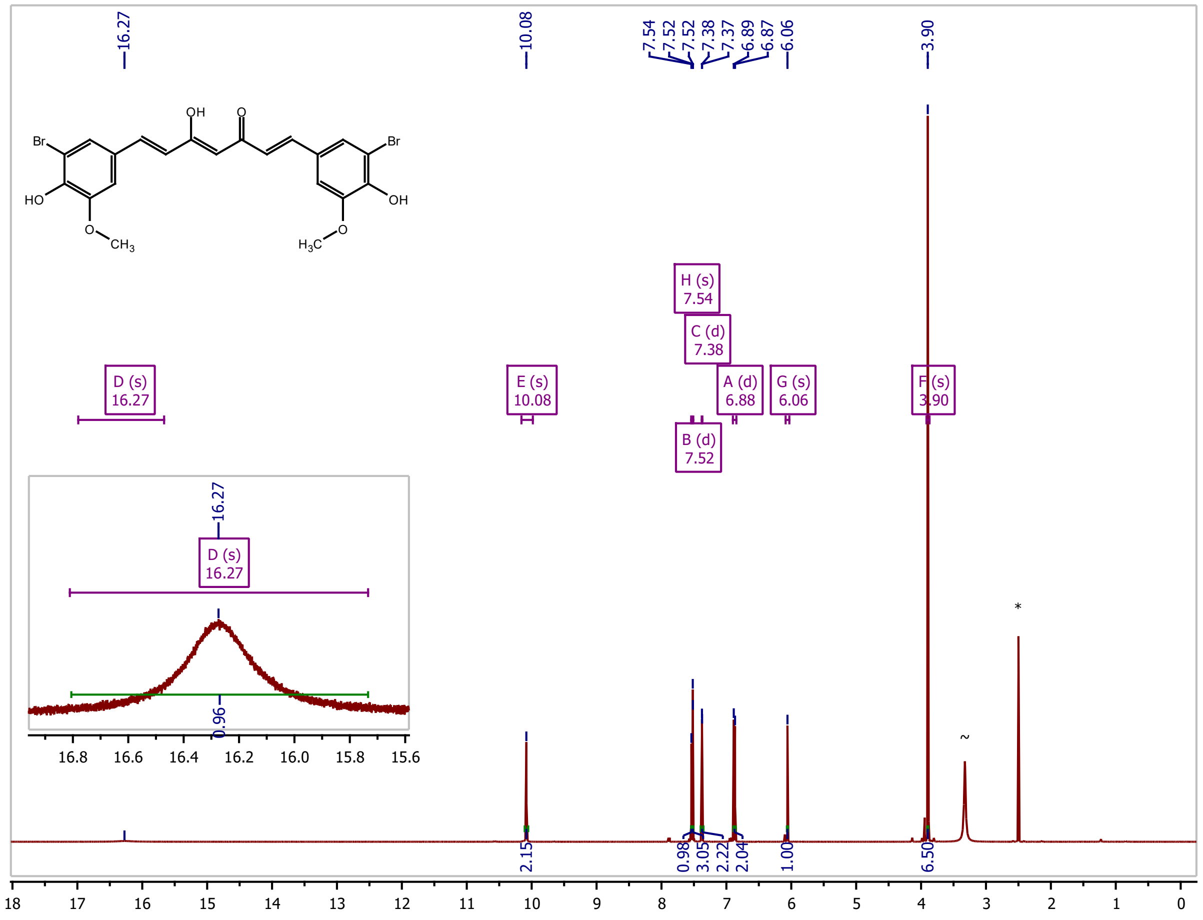

Figure S27. ^{13}C NMR of curcumin **1b**.

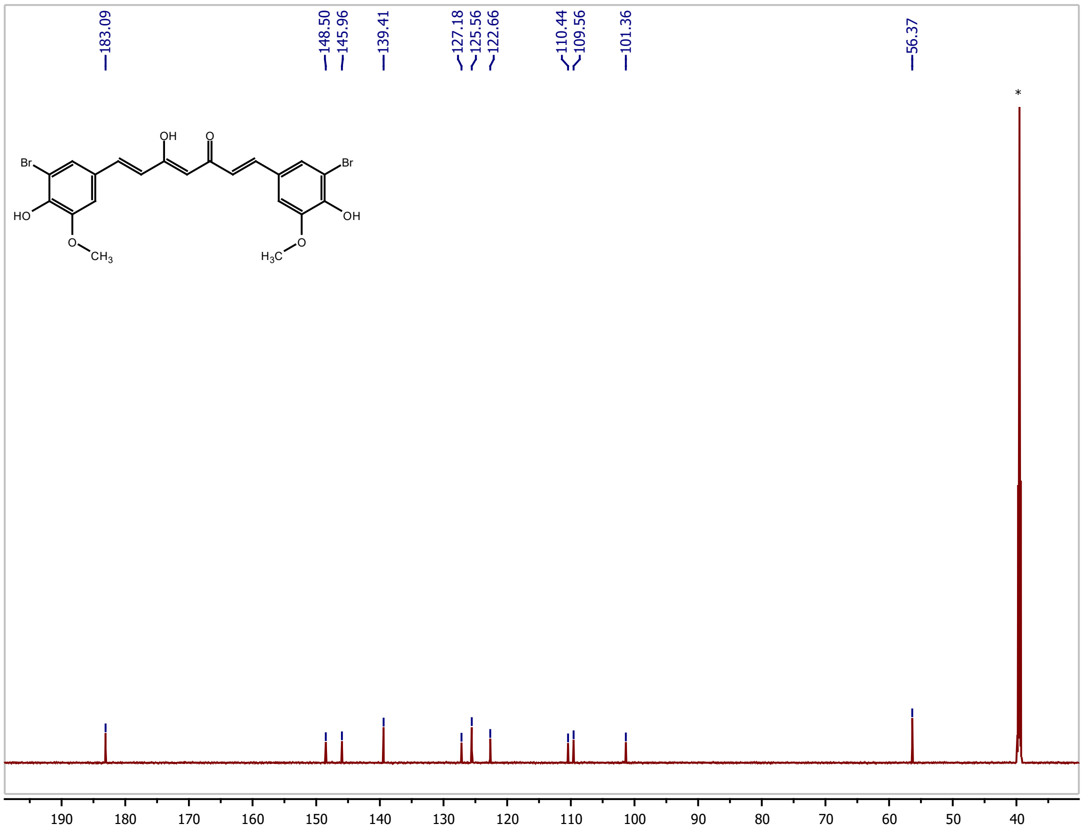

Figure S28. ^{1}H-^{1}H COSY of curcumin **1b**.

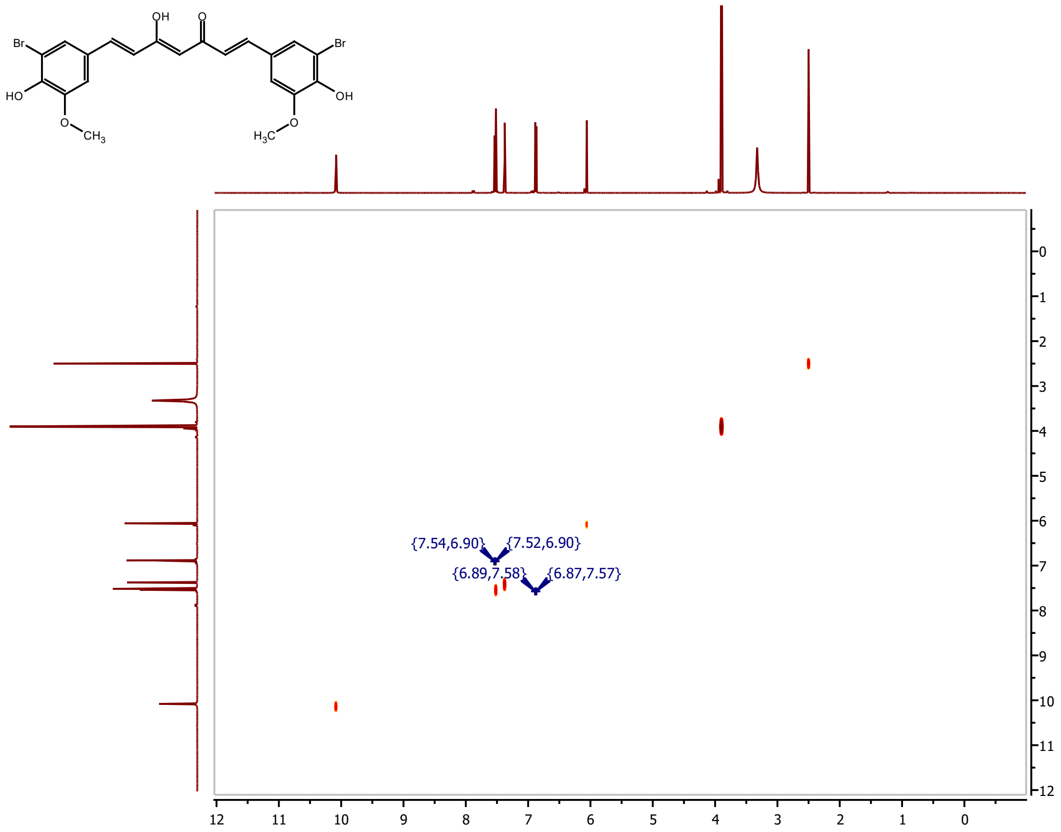

Figure S29. ^{1}H-^{13}C HSQC of curcumin **1b**.

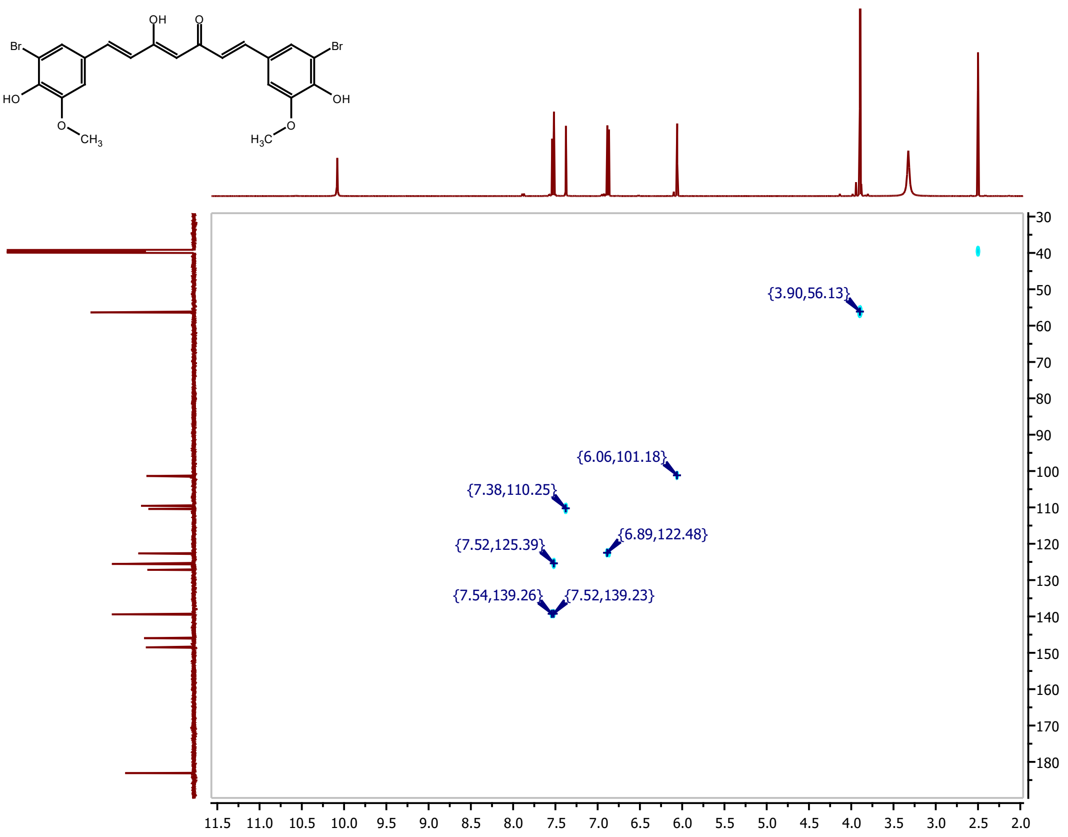

Figure S30. ^{1}H-^{13}C HMBC of curcumin **1b.**

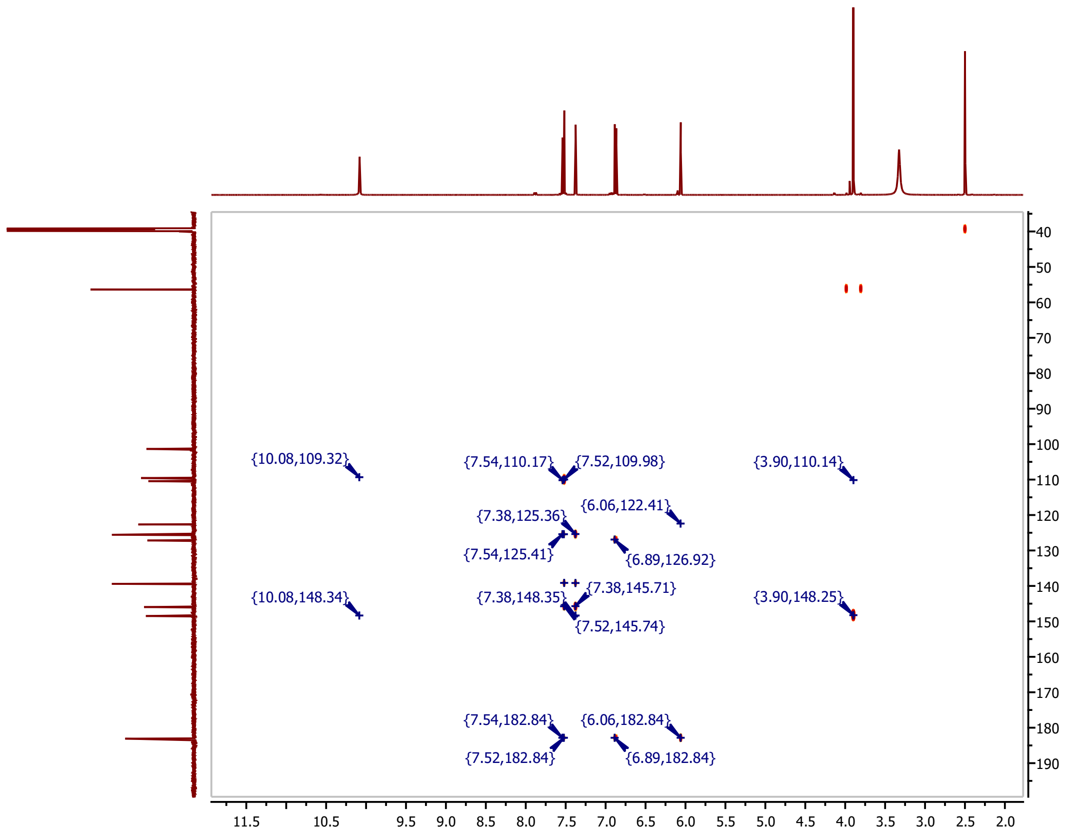

Figure S31. NMR experiments of compound **2a**.

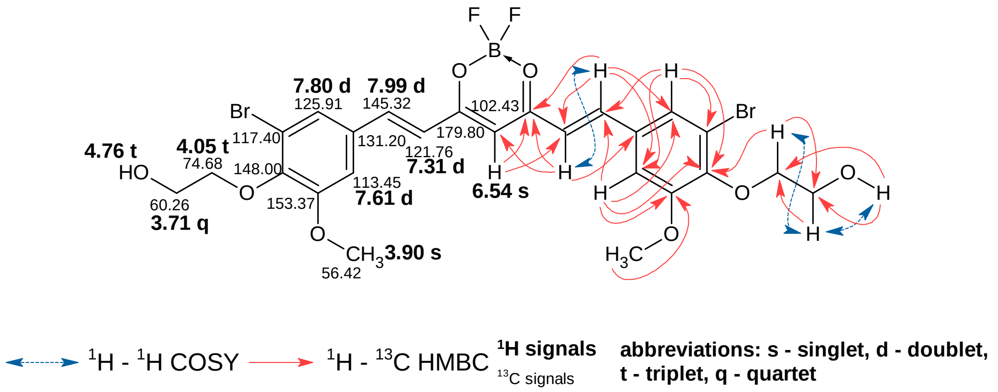

Figure S32. ^{1}H NMR of curcumin **2a**.

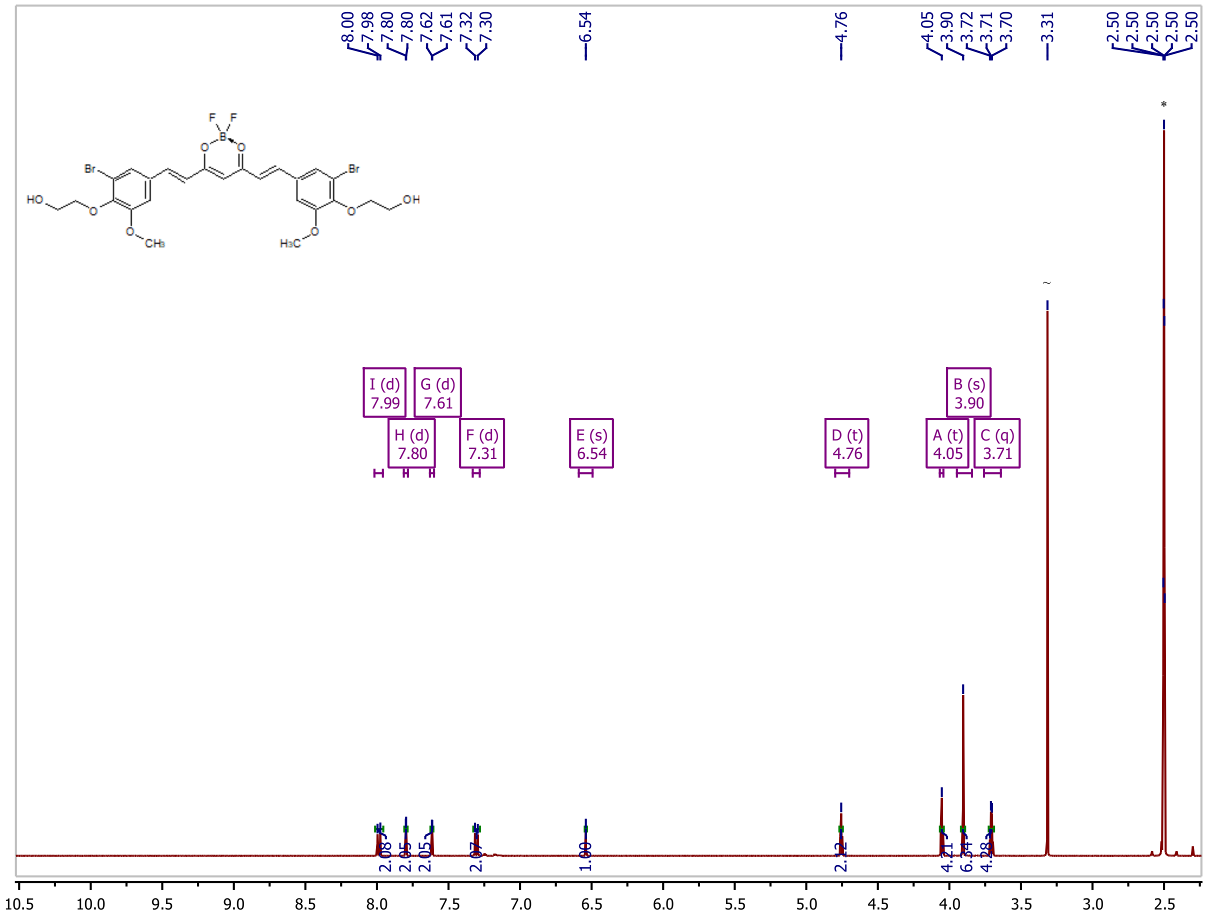

Figure S33. ^{13}C NMR of curcumin **2a**.

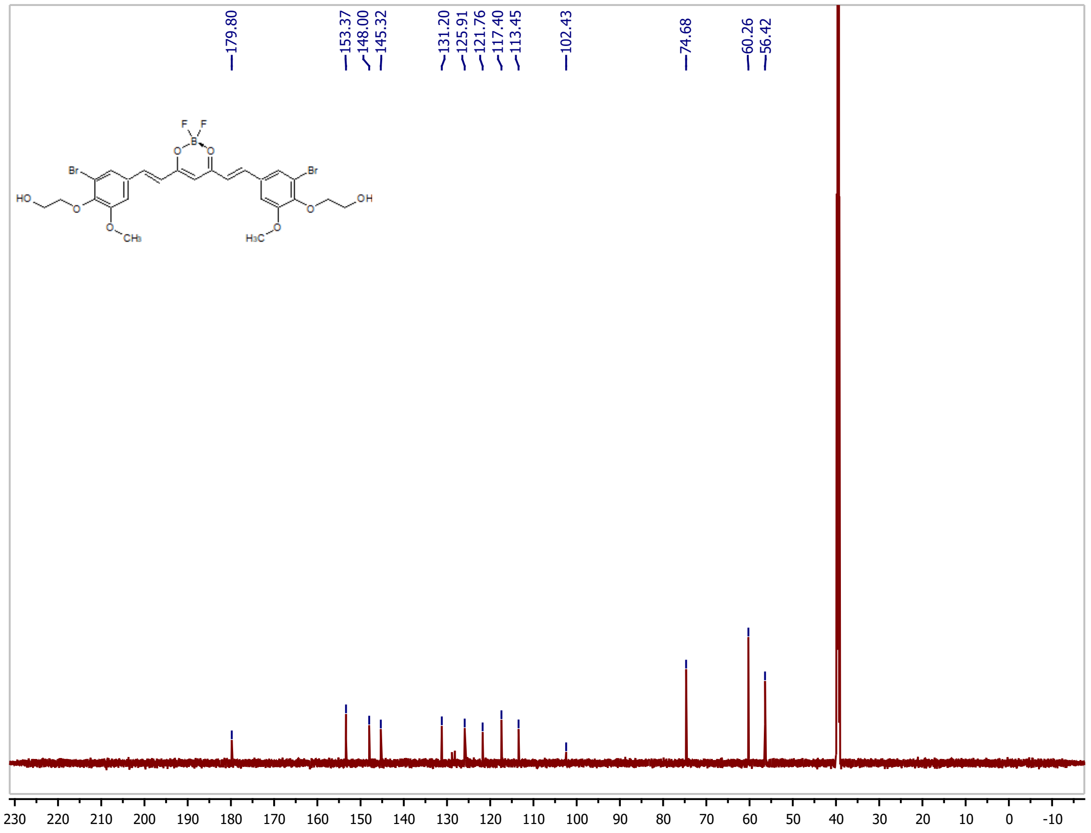

Figure S34. ^{1}H-^{1}H COSY of curcumin **2a**.

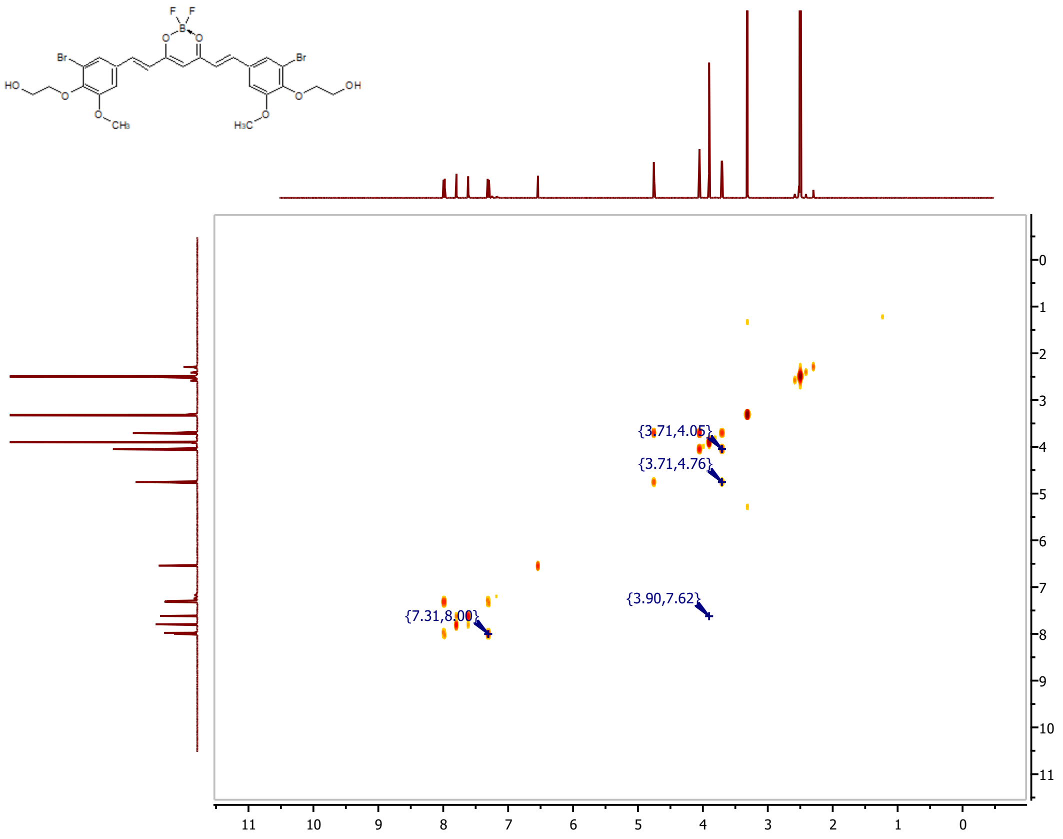

Figure S35. ^{1}H-^{13}C HSQC of curcumin **2a**.

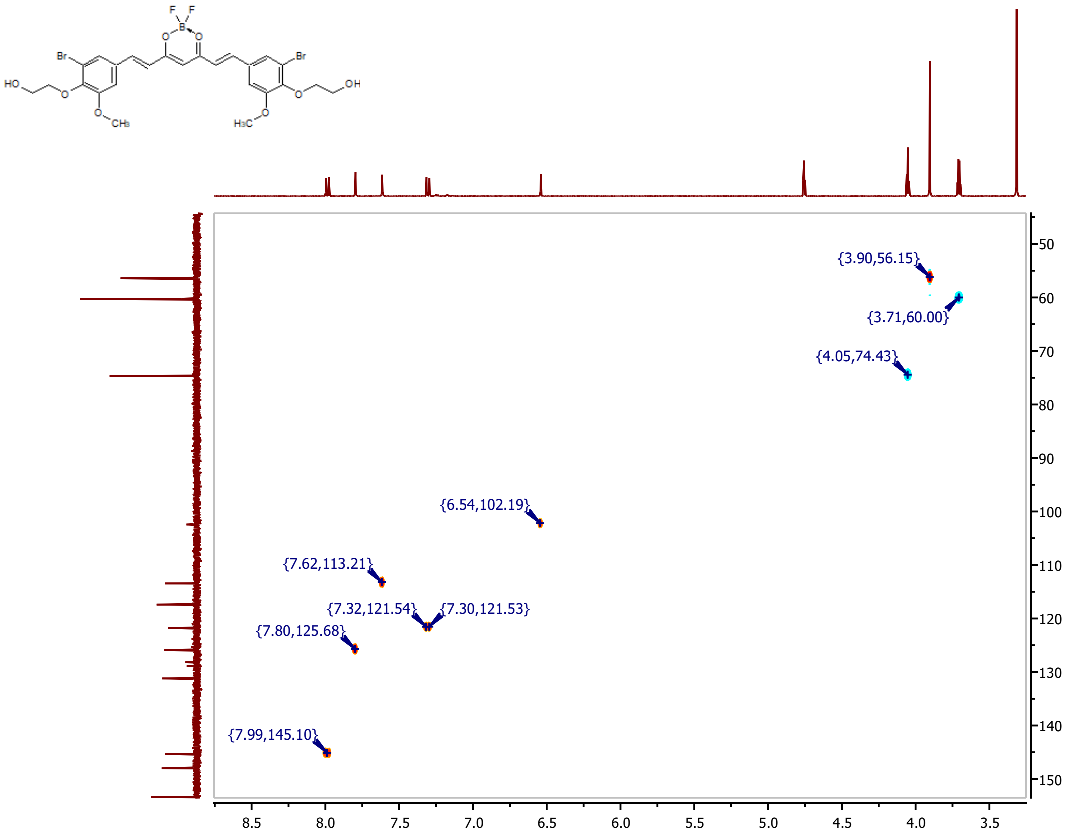

Figure S36. ^{1}H-^{13}C HMBC of curcumin **2a.**

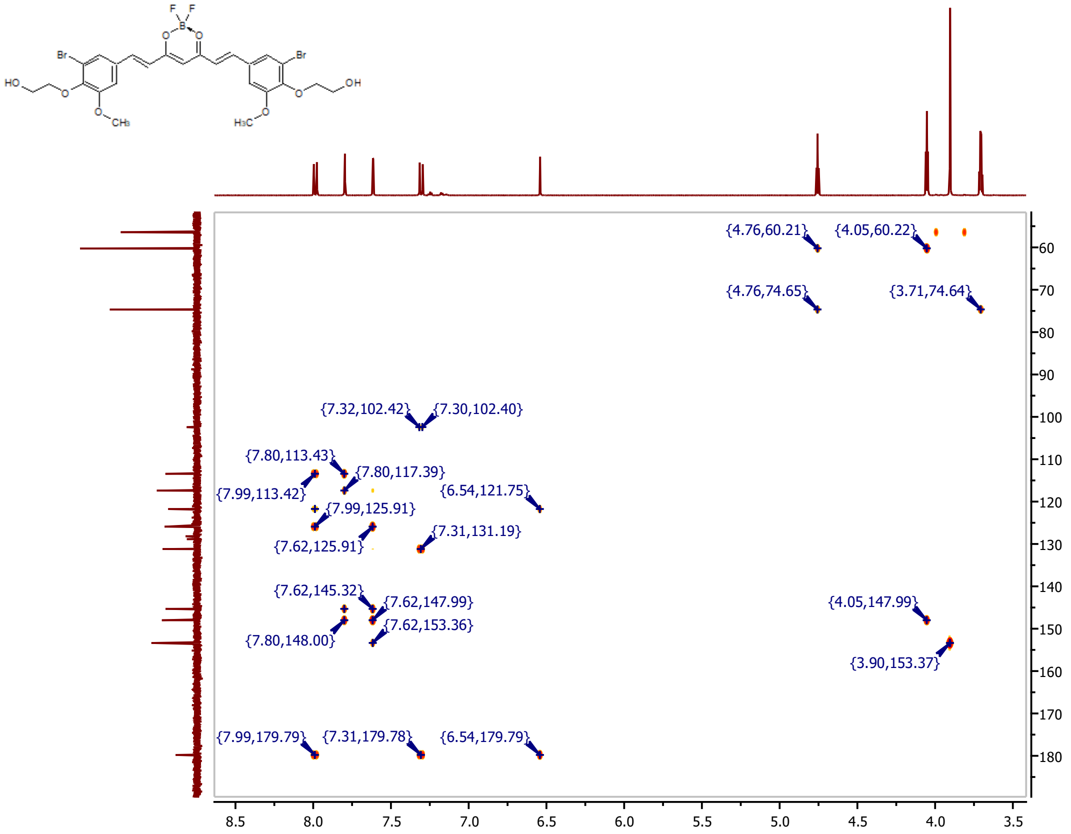

Figure S37. NMR experiments of compound **3a**.

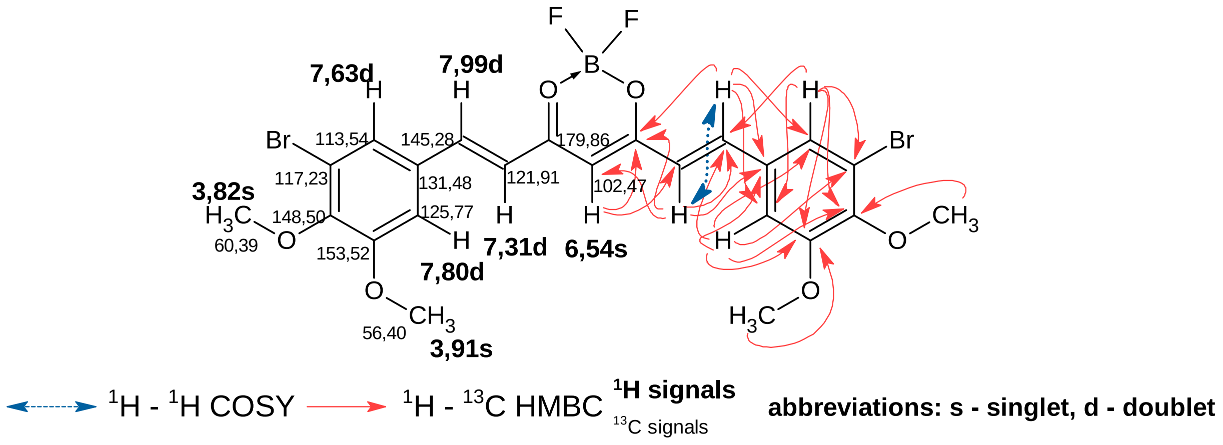

Figure S38. ^1H NMR of curcumin **3a**.

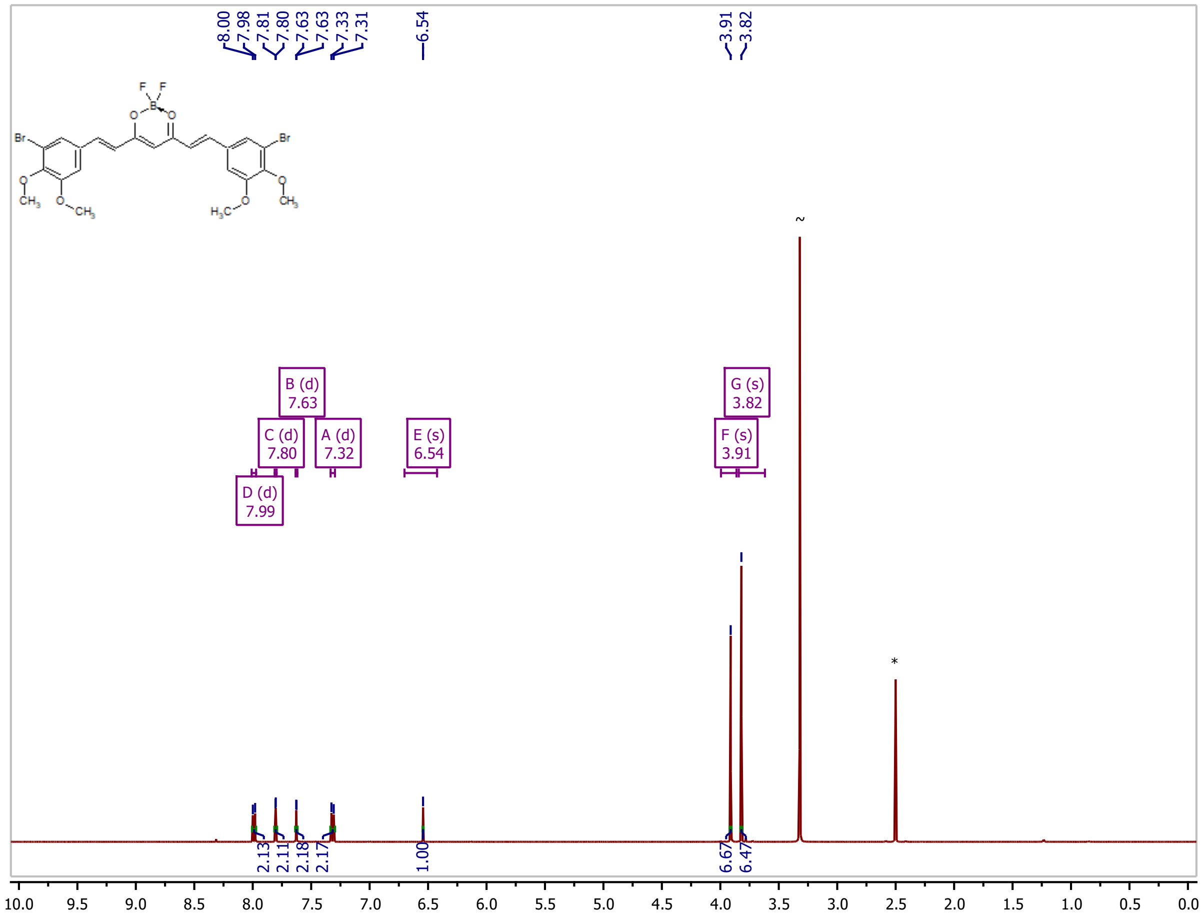

Figure S39. ^{13}C NMR of curcumin **3a**.

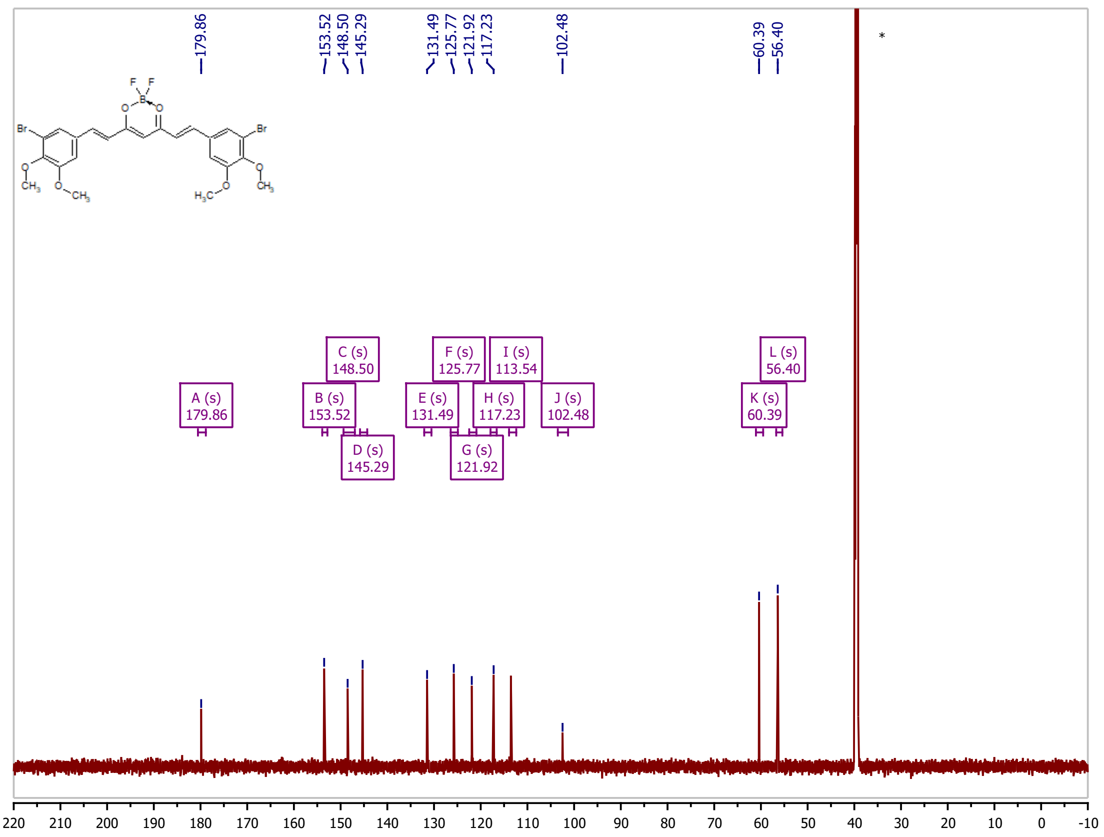

Figure S40. ^{1}H-^{1}H COSY of curcumin **3a**.

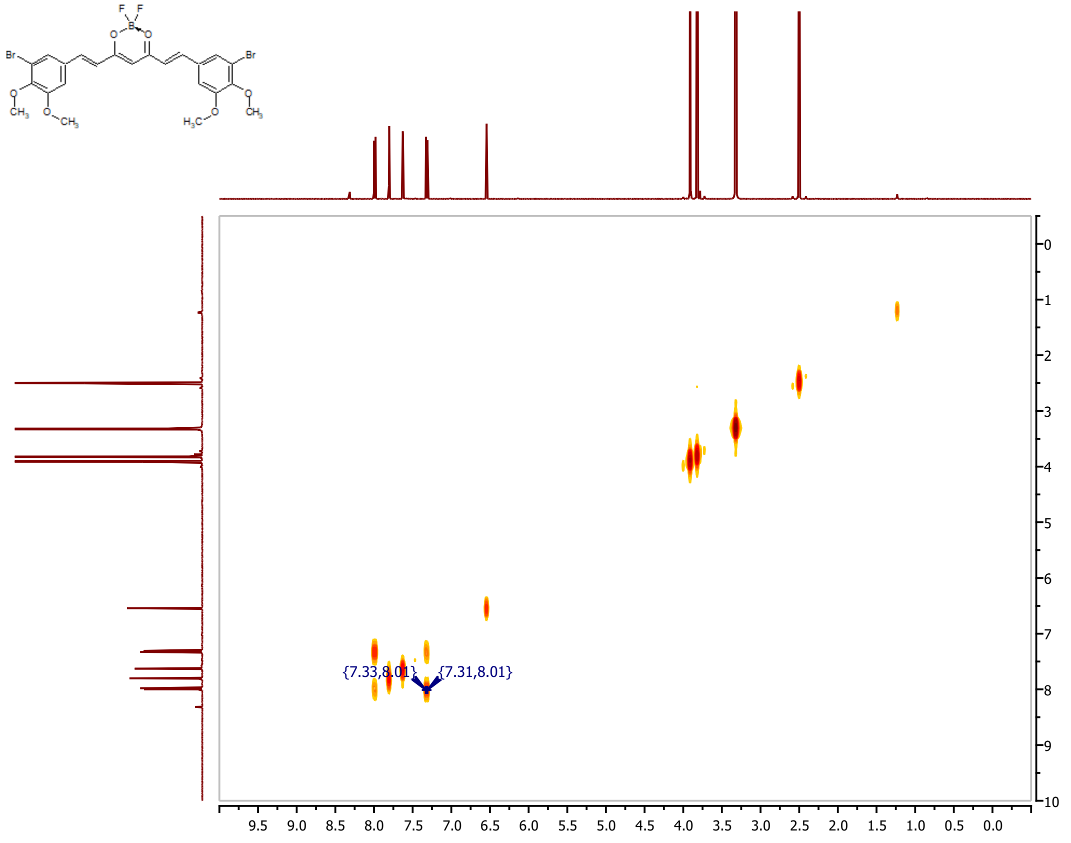

Figure S41. ^{1}H-^{13}C HSQC of curcumin **3a**.

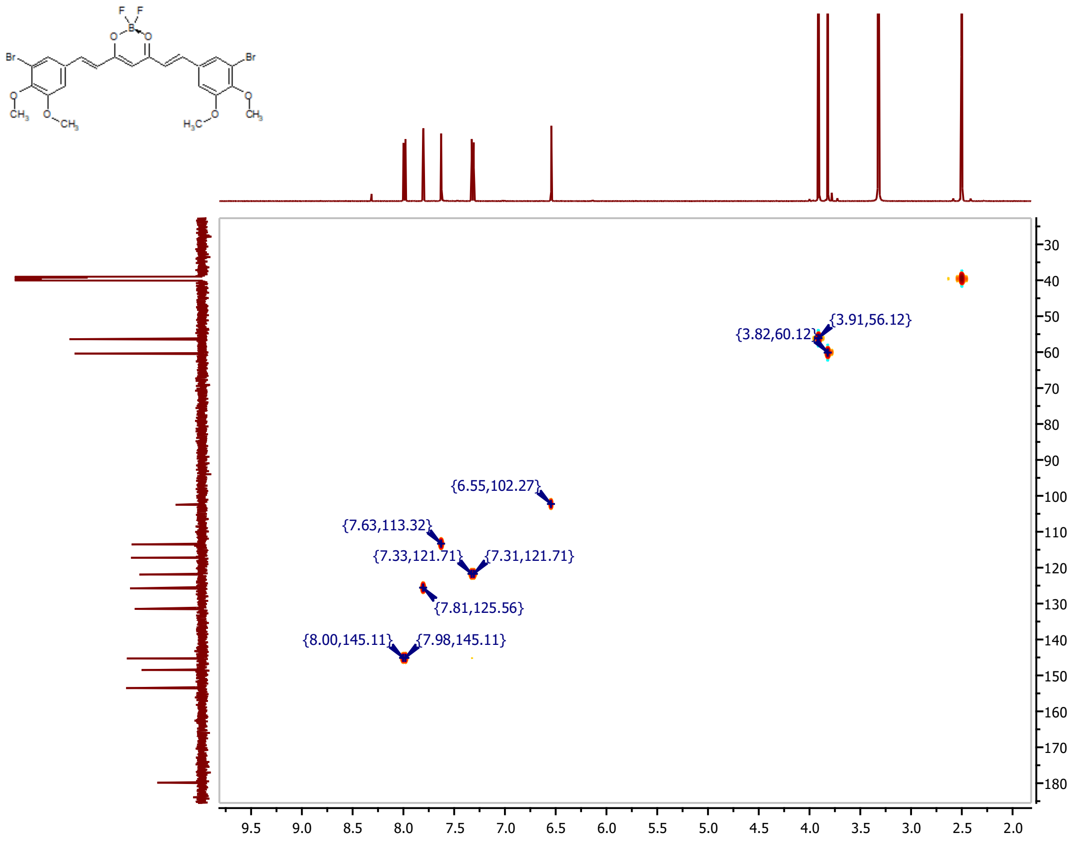

Figure S42. ^{1}H-^{13}C HMBC of curcumin **3a.**

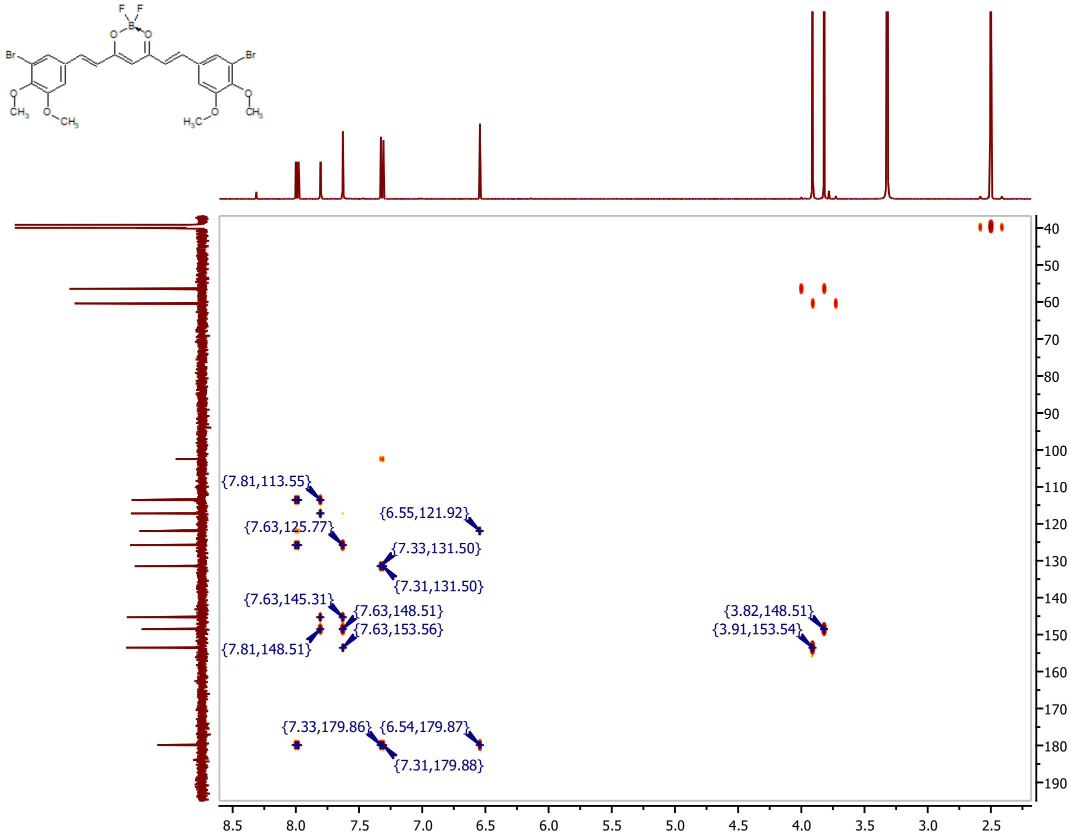

Figure S43. NMR experiments of compound **3a**.

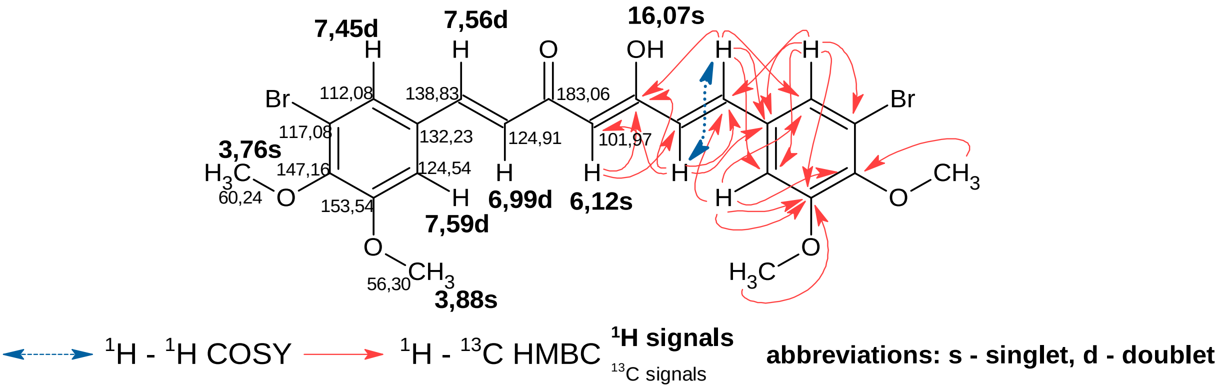

Figure S44. ¹H NMR of curcumin **3b**.

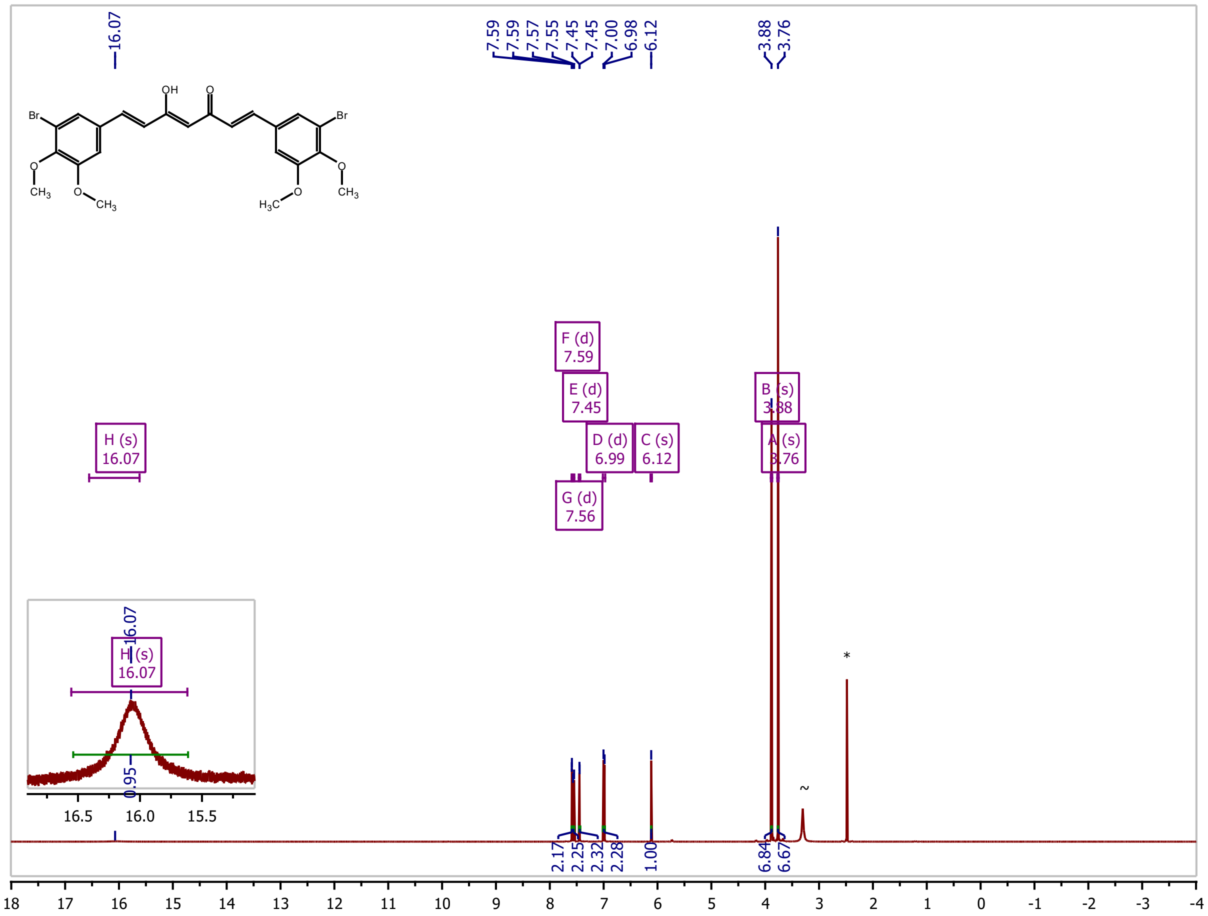

Figure S45. ^{13}C NMR of curcumin **3b**.

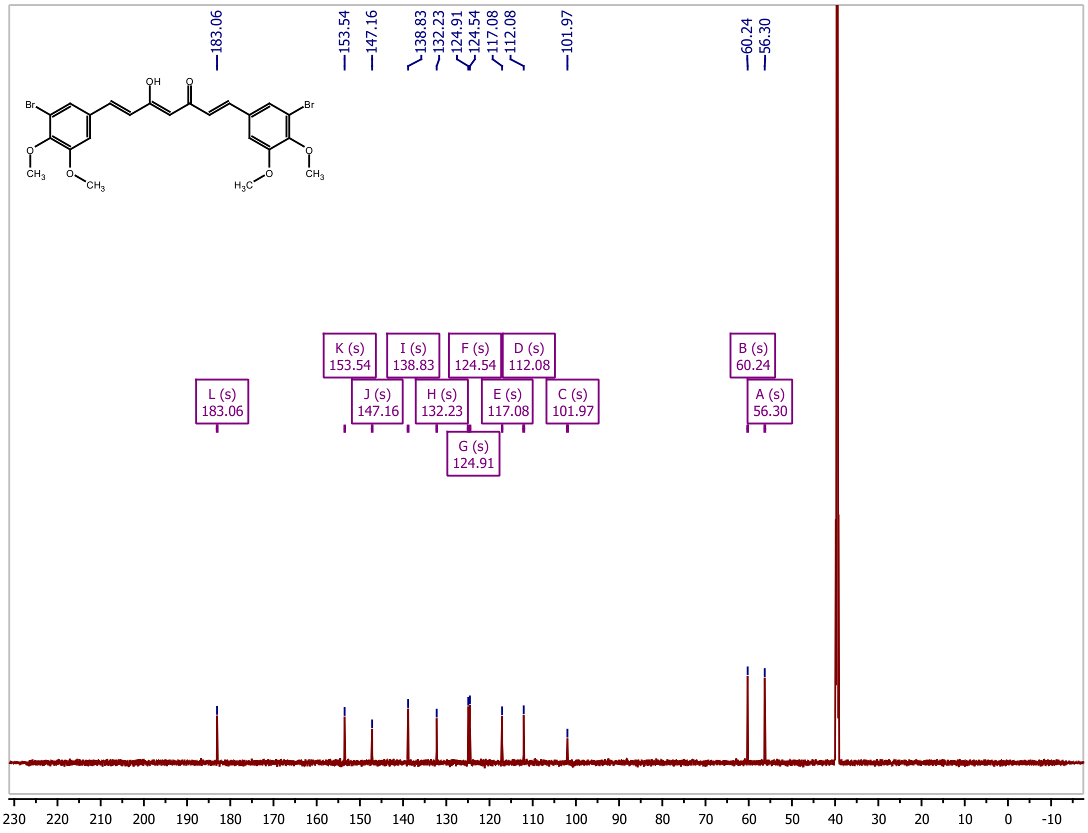

Figure S46. ^{1}H-^{1}H COSY of curcumin **3b**.

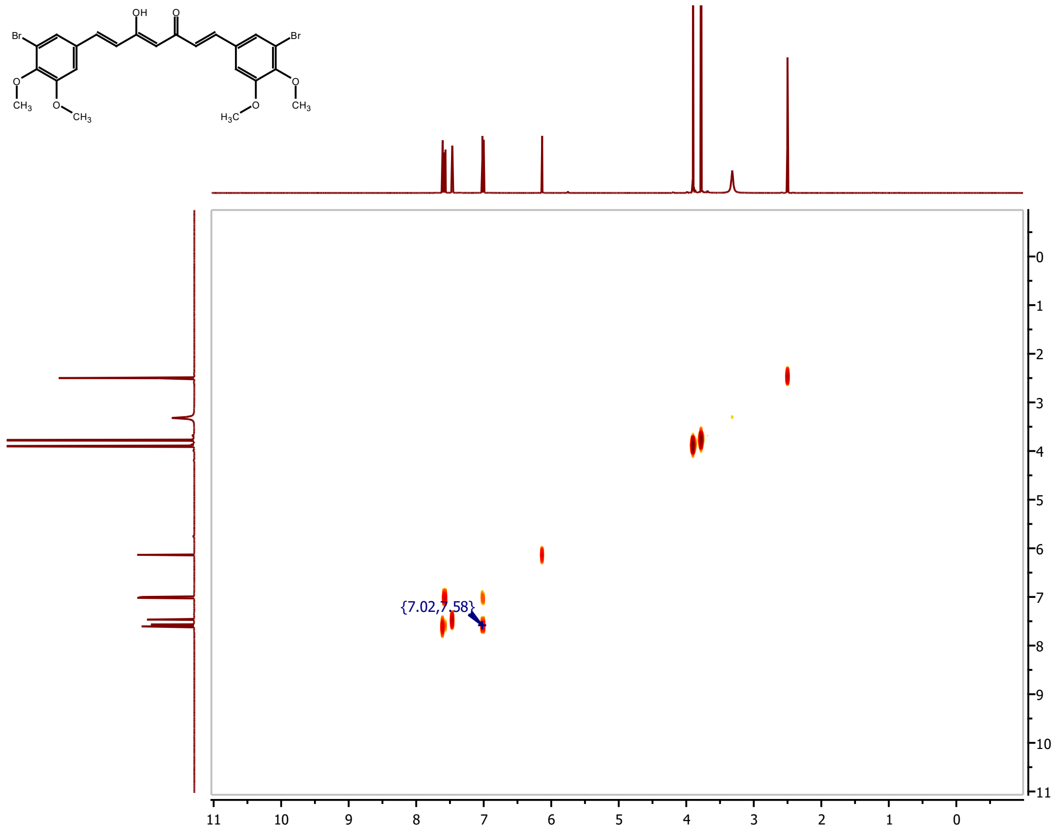

Figure S47. ^{1}H-^{13}C HSQC of curcumin **3b**.

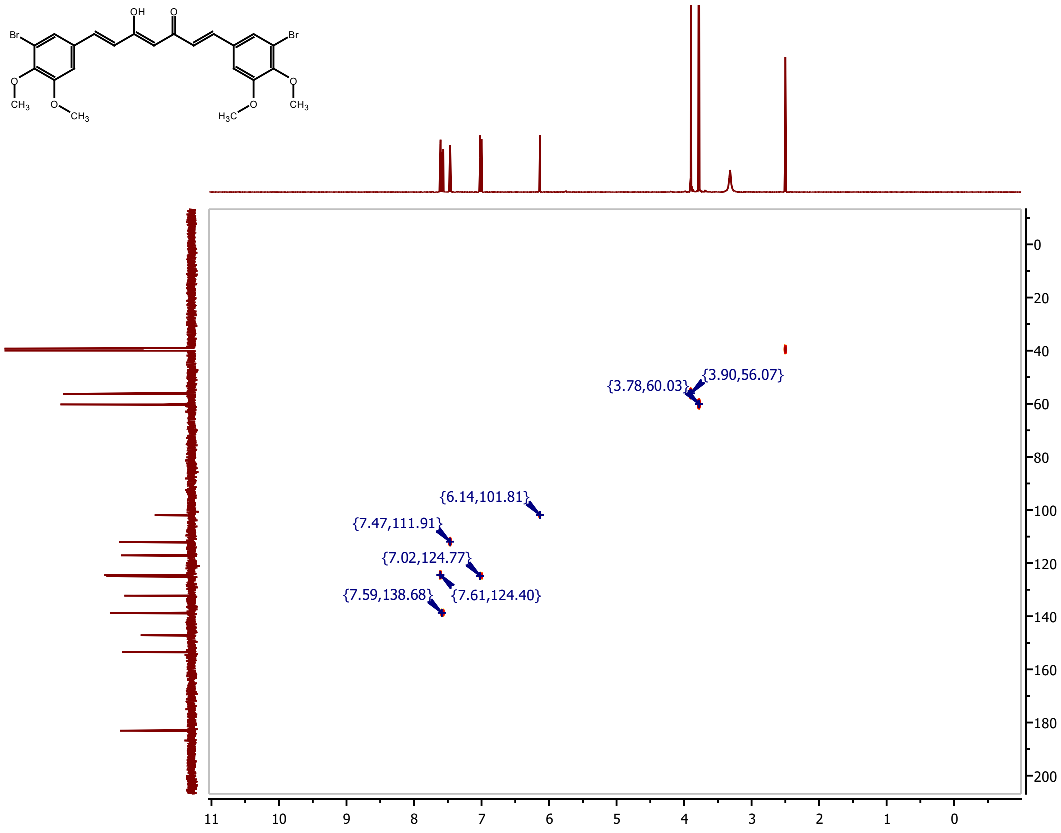

Figure S48. ^{1}H-^{13}C HMBC of curcumin **3b.**

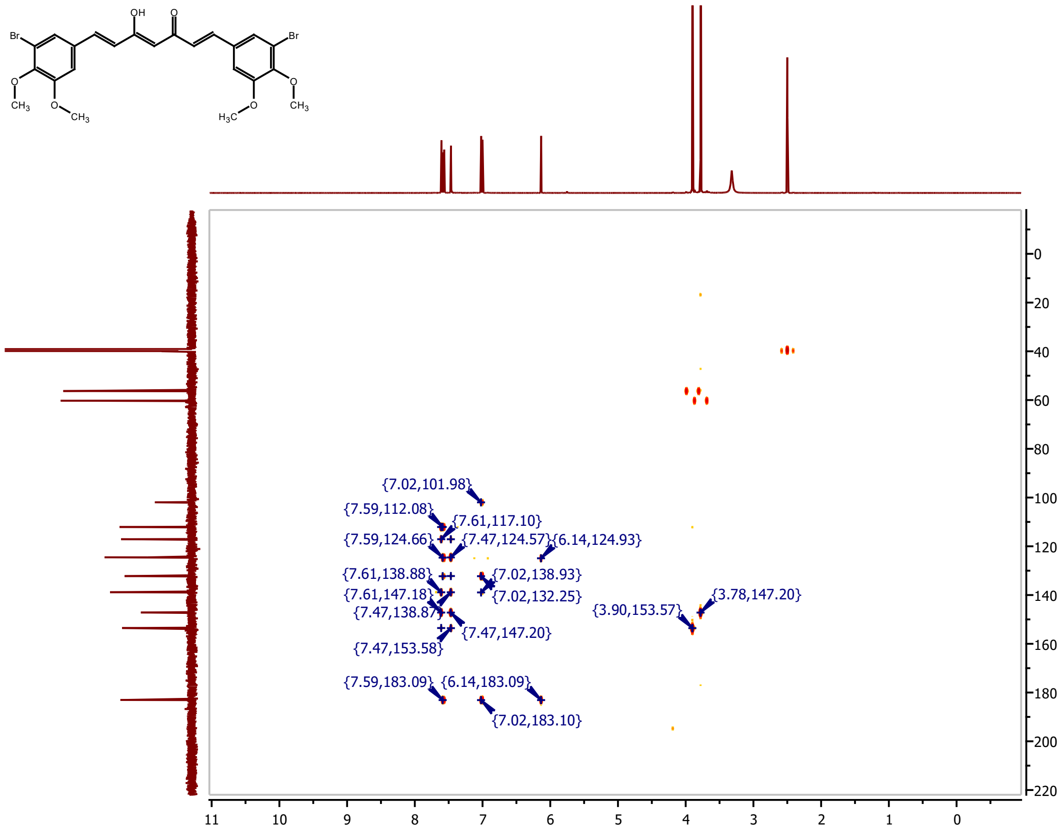

Figure S49. NMR experiments of compound **4a**.

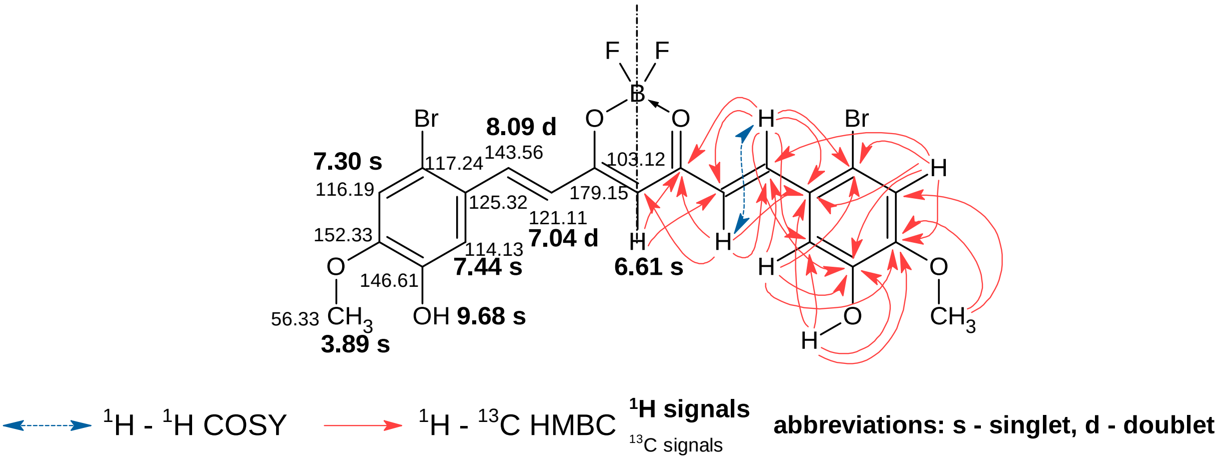

Figure S50. ¹H NMR of curcumin **4a**.

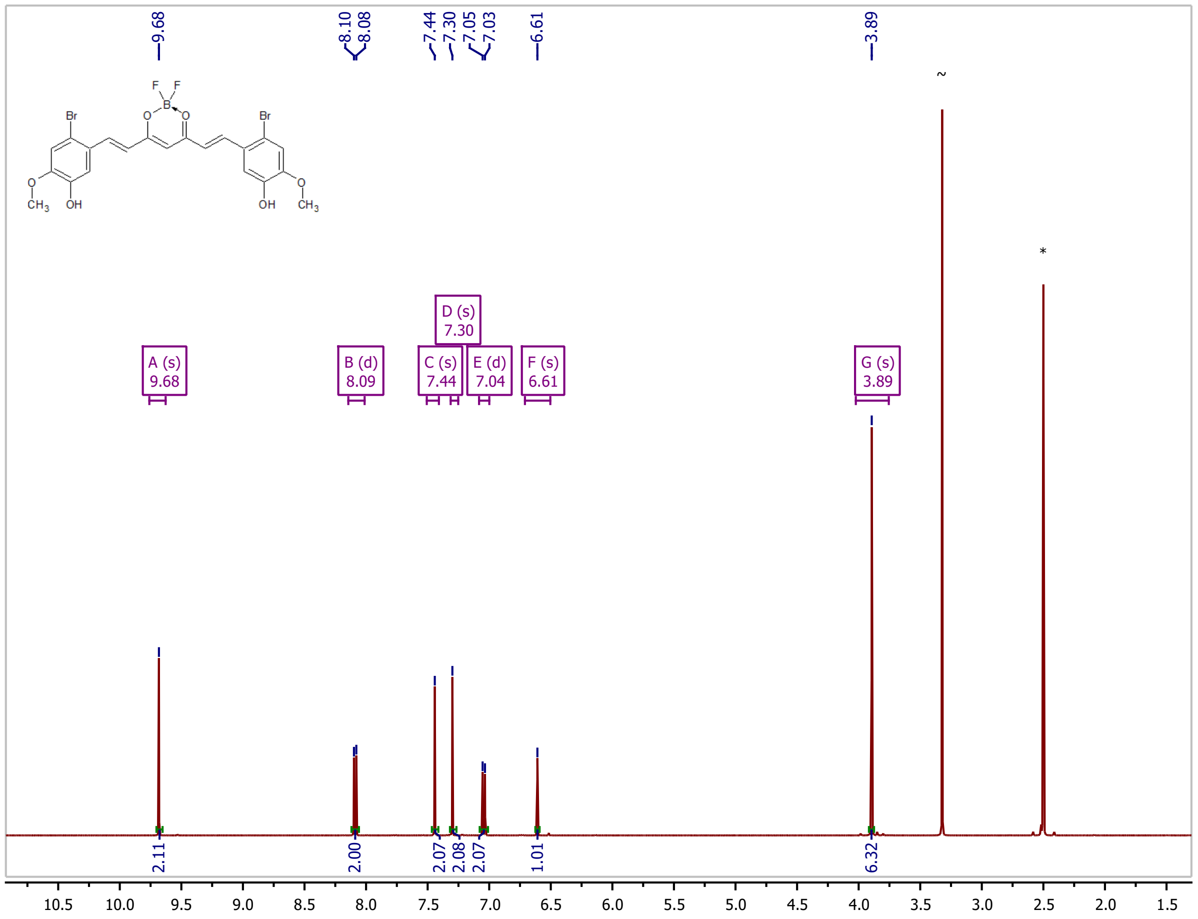

Figure S51. ^{13}C NMR of curcumin **4a**.

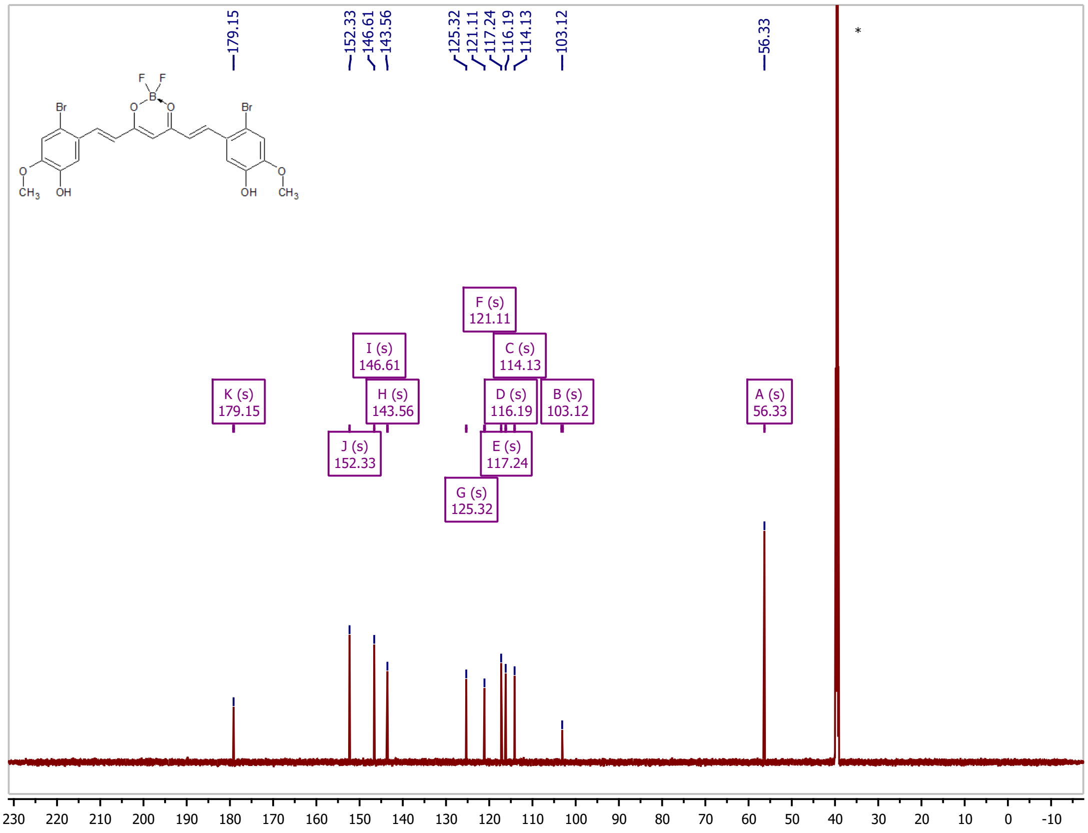

Figure S52. ^{1}H-^{1}H COSY of curcumin **4a**.

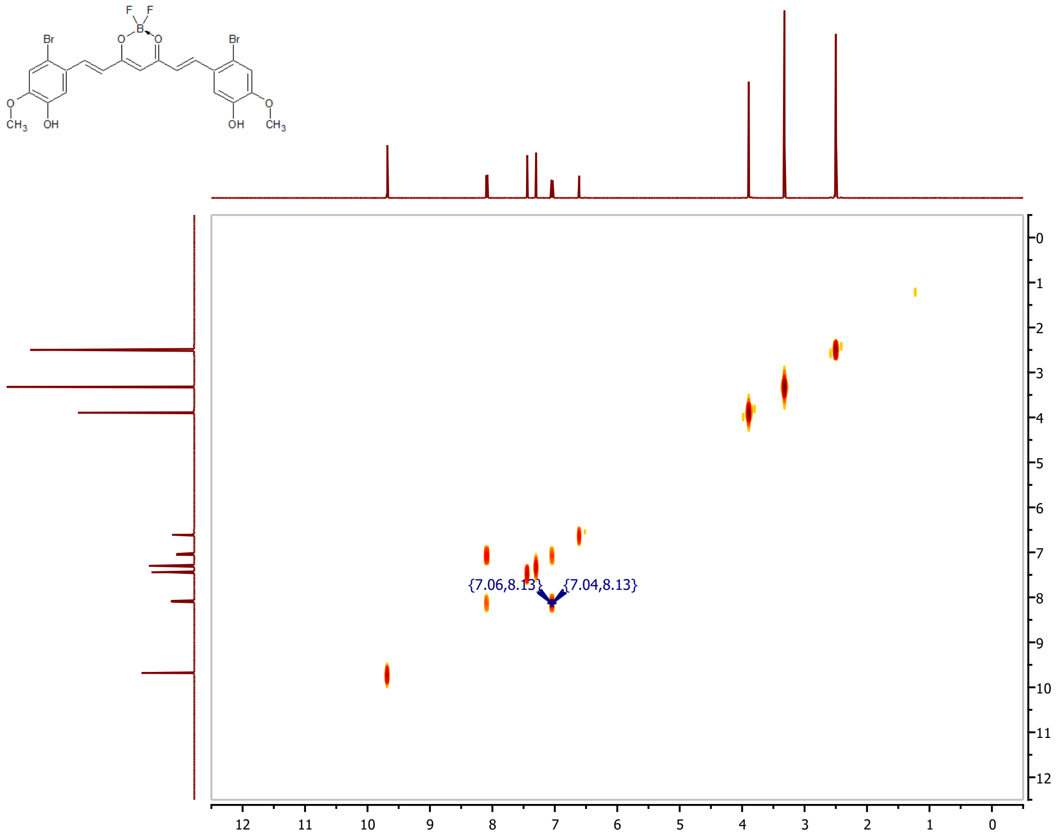

Figure S53. ^{1}H-^{13}C HSQC of curcumin **4a**.

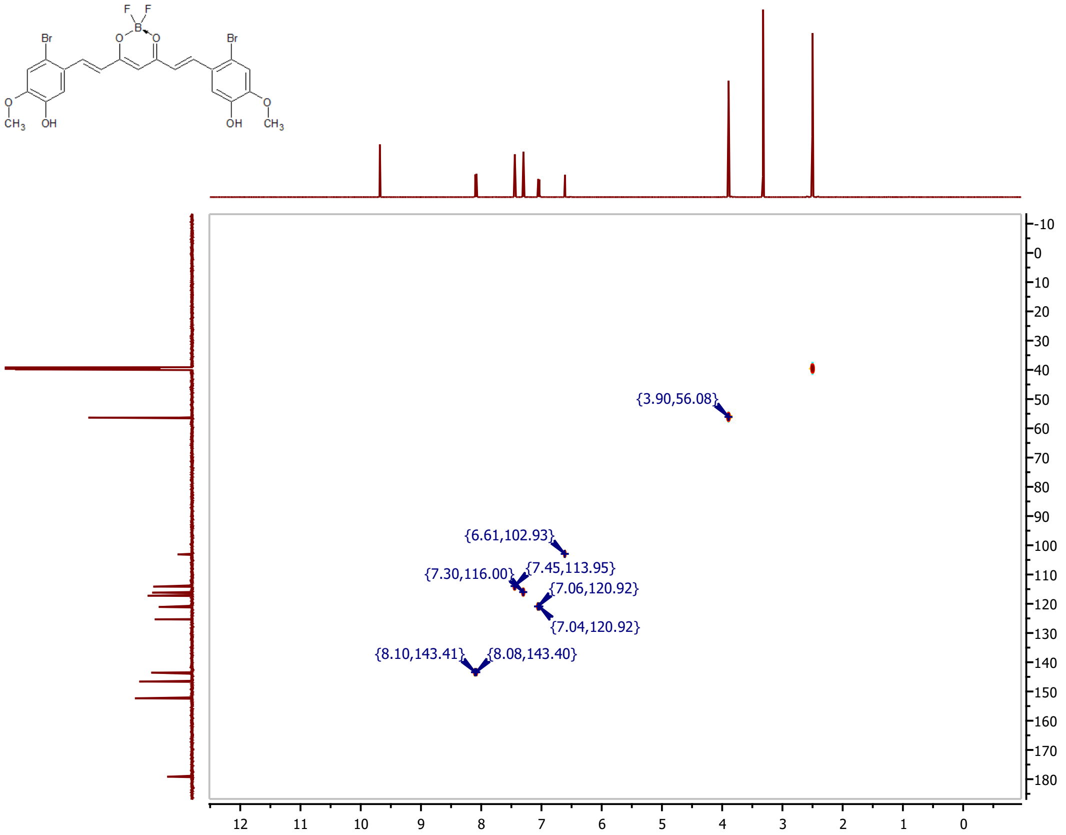

Figure S54. ^{1}H-^{13}C HMBC of curcumin **4a.**

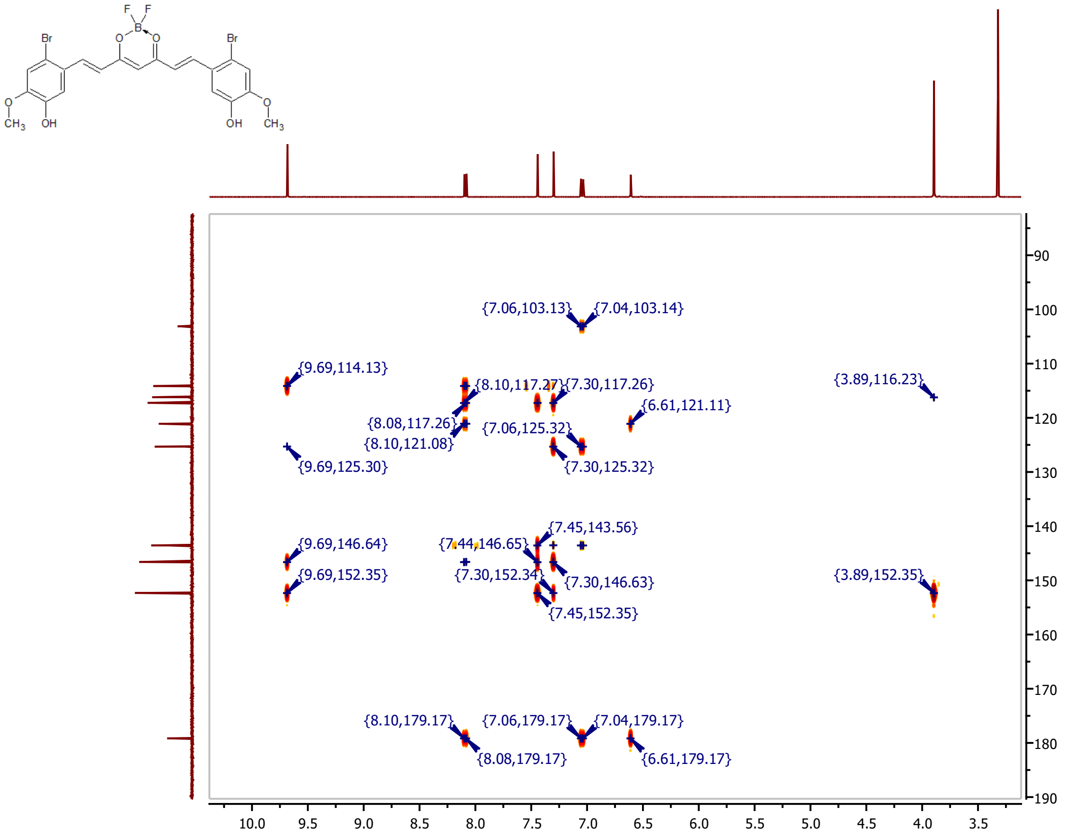

Figure S55. NMR experiments of compound **4b**.

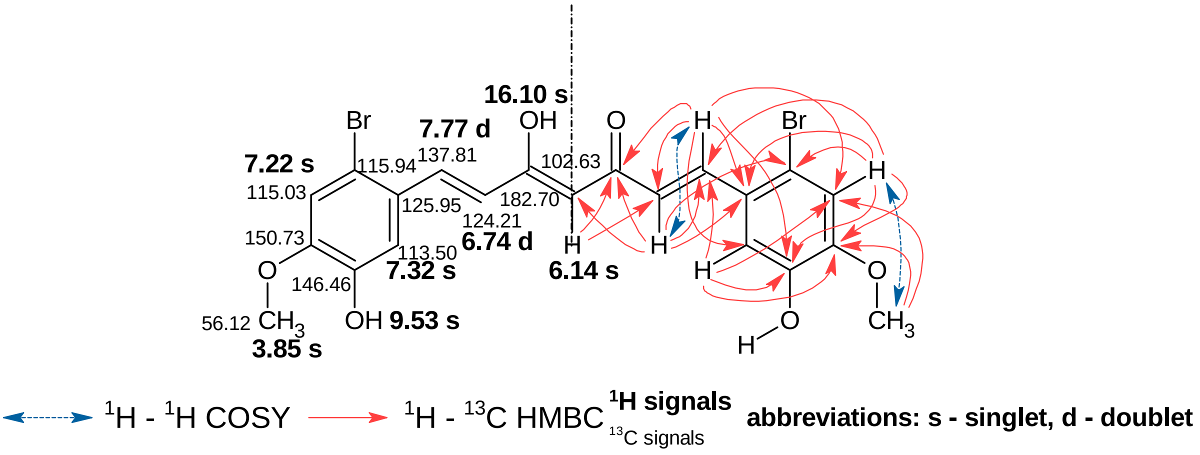

Figure S56. ^{1}H NMR of curcumin **4b**.

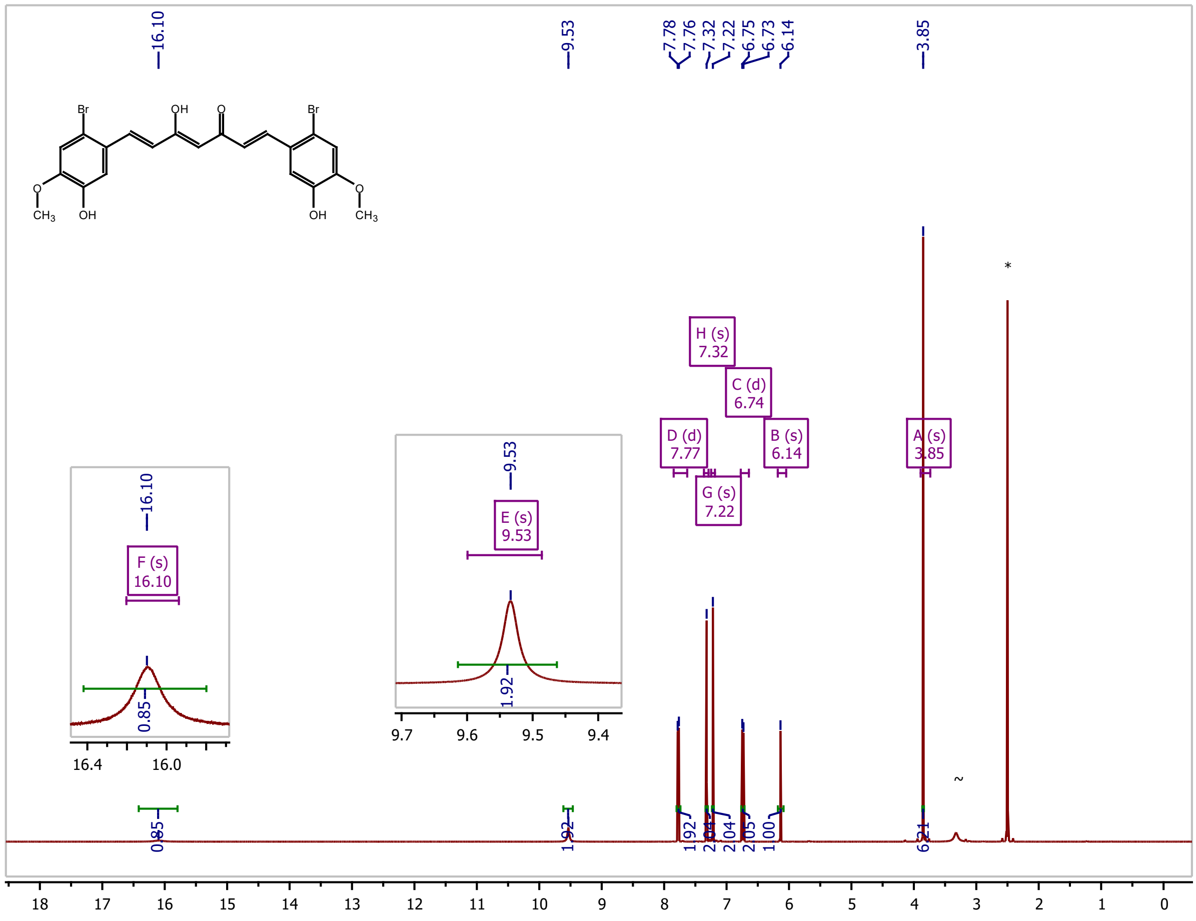

Figure S57. ^{13}C NMR of curcumin **4b**.

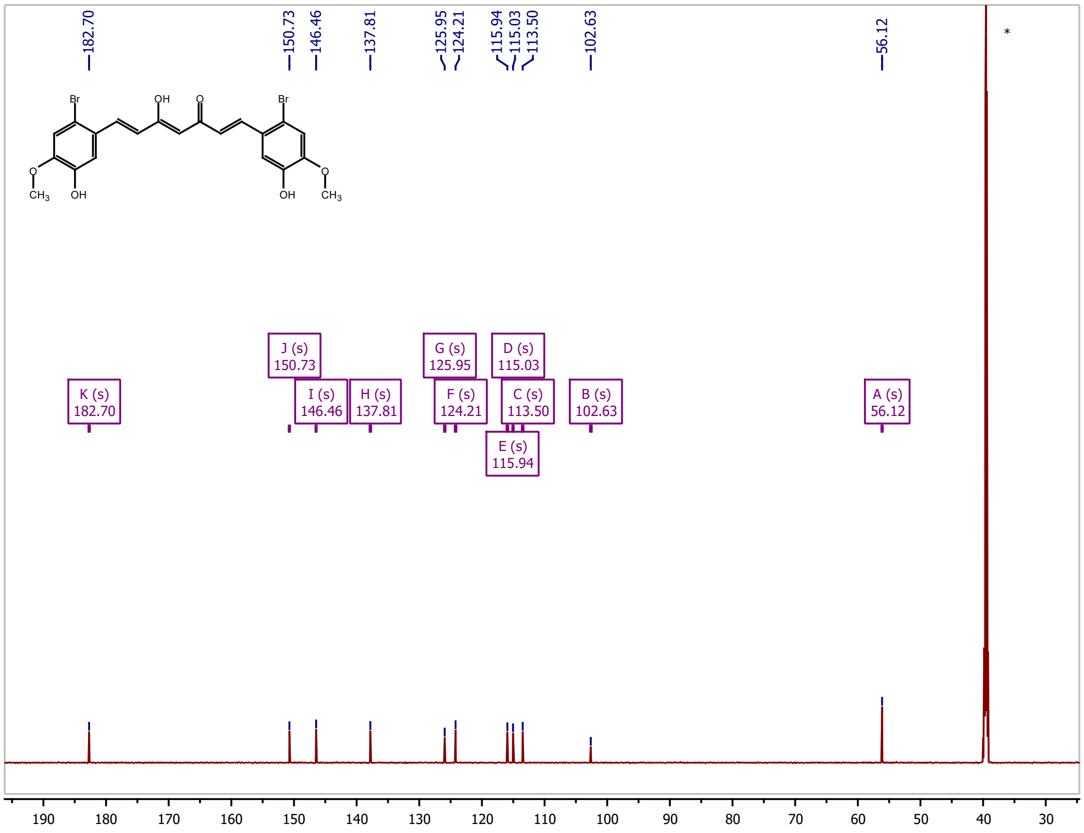

Figure S58. ^{1}H-^{1}H COSY of curcumin **4b**.

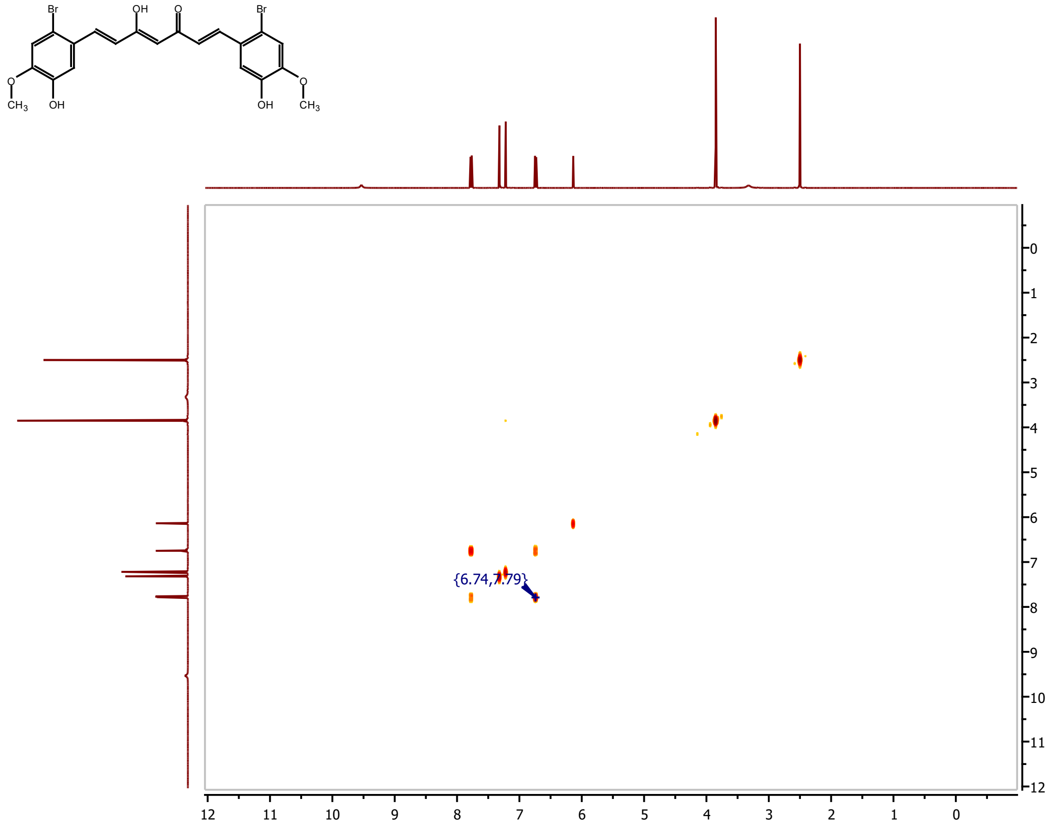

Figure S59. ^{1}H-^{13}C HSQC of curcumin **4b**.

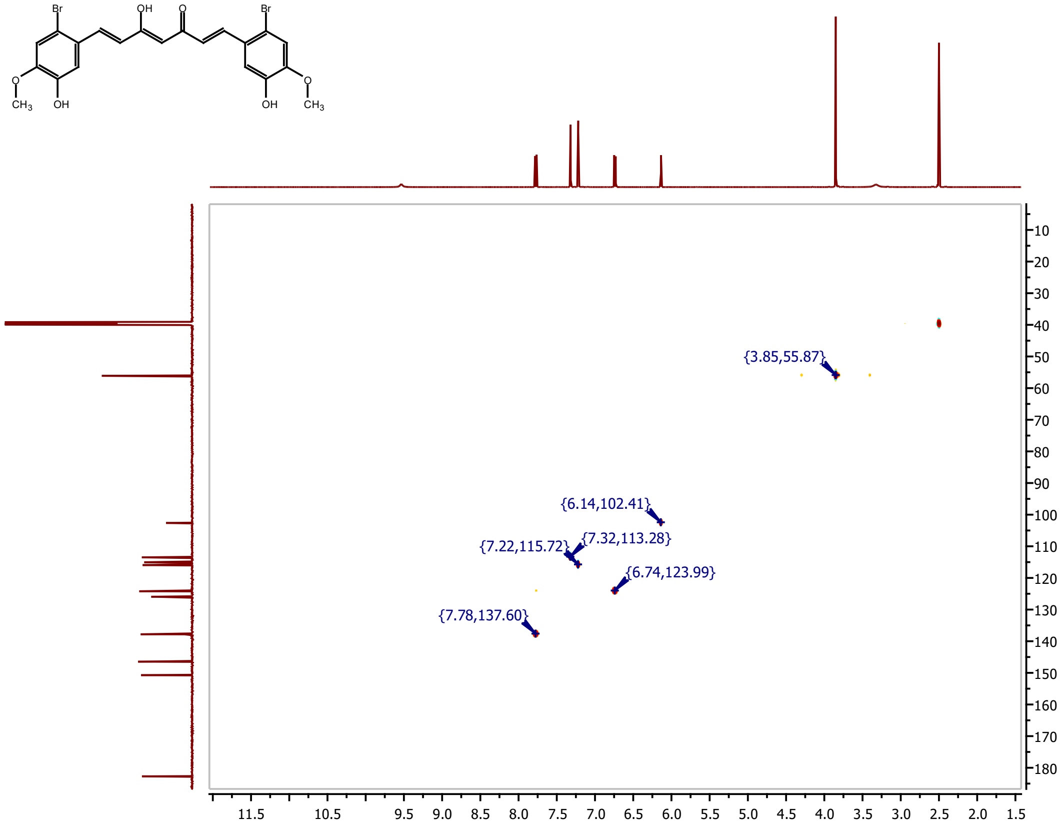

Figure S60. ^{1}H-^{13}C HMBC of curcumin **4b.**

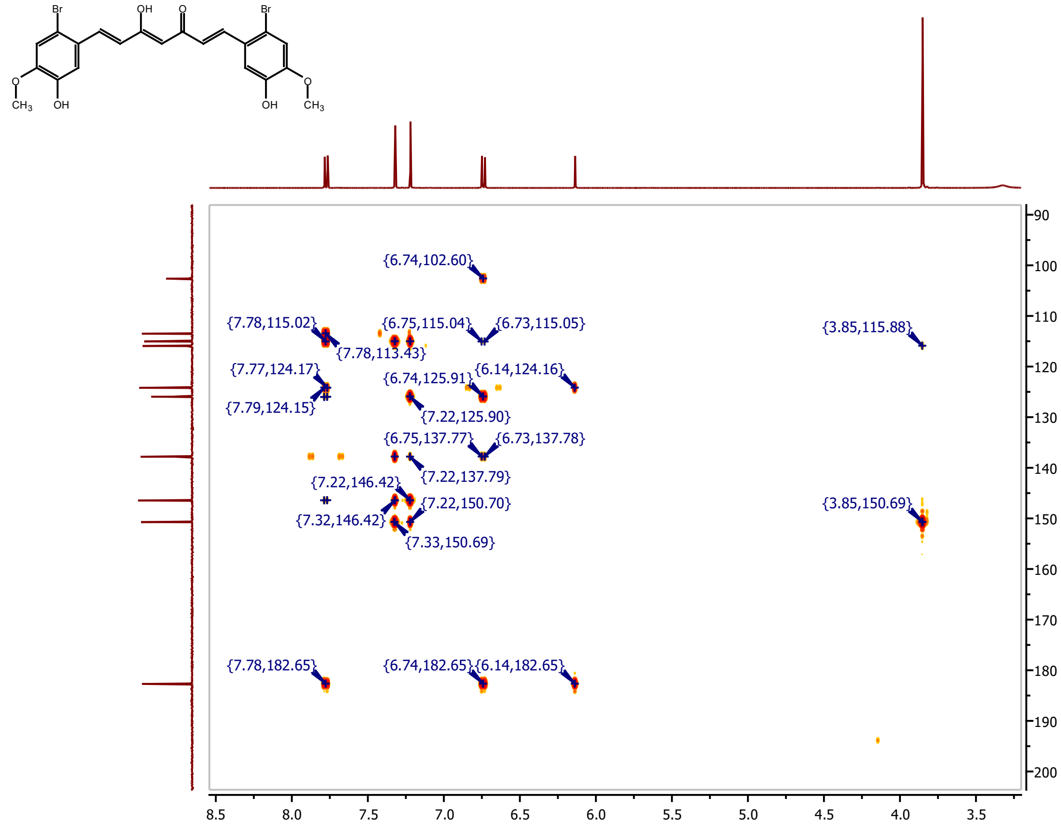

Figure S61. NMR experiments of compound **5a**.

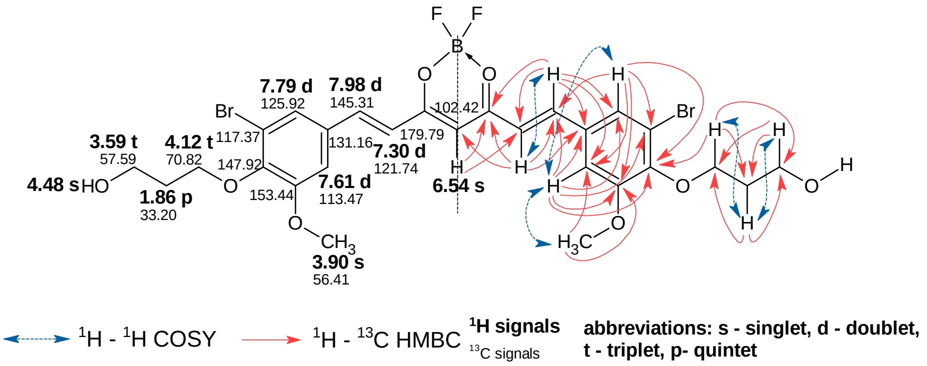

Figure S62. ¹H NMR of curcumin **5a**.

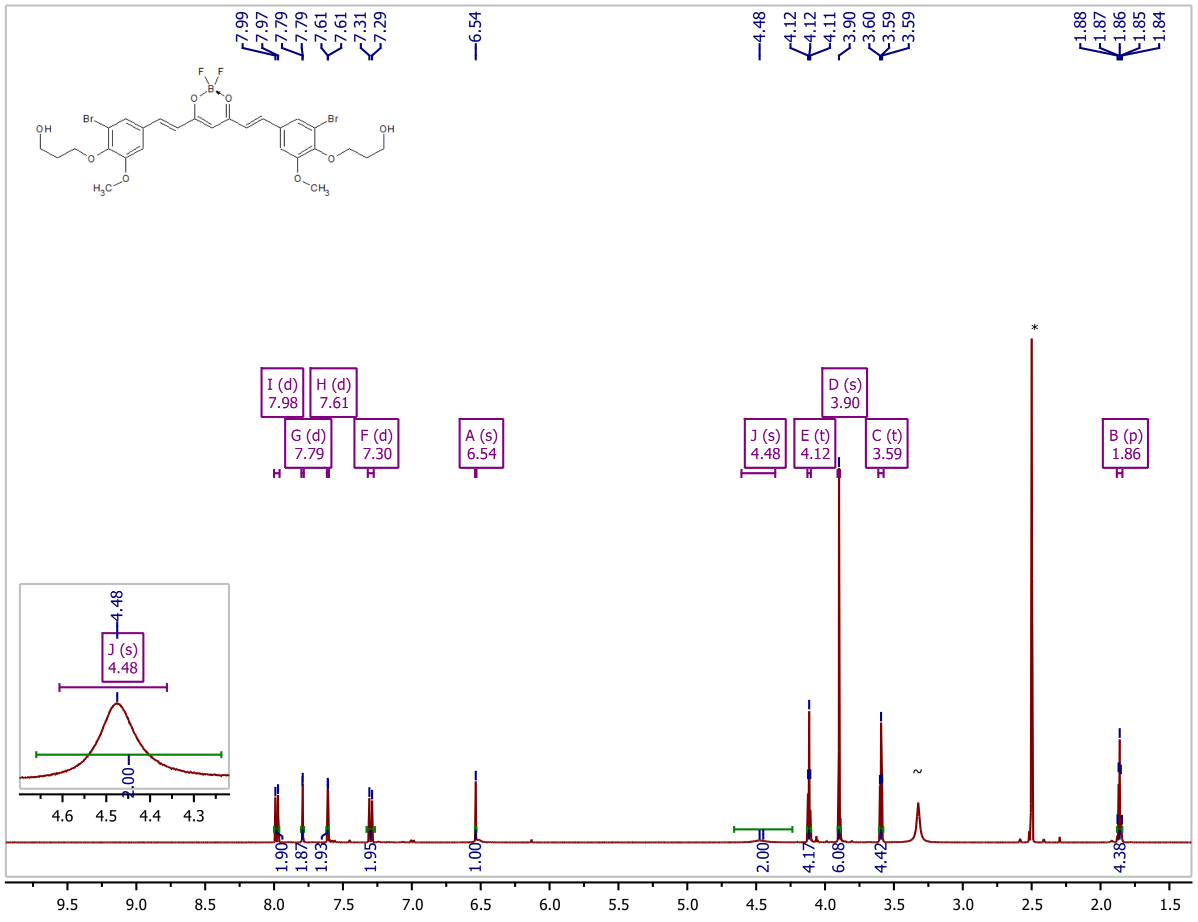

Figure S63. ^{13}C NMR of curcumin **5a**.

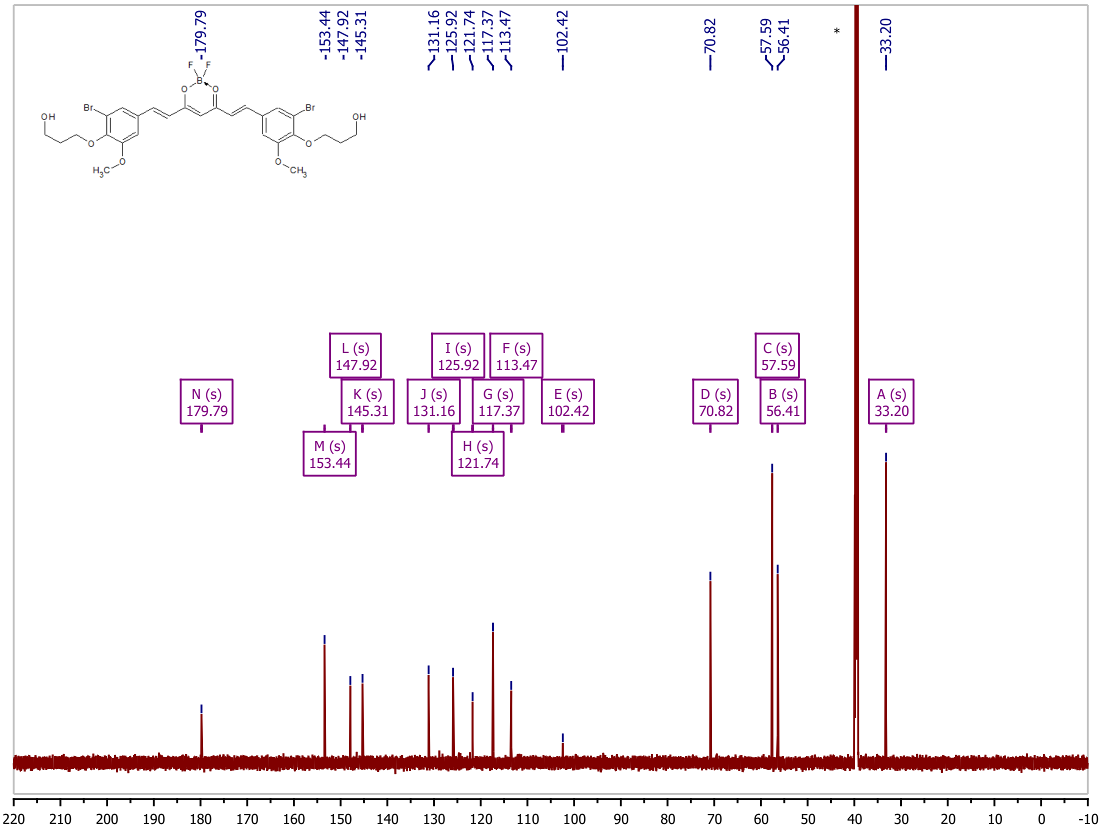

Figure S64. ^{1}H-^{1}H COSY of curcumin **5a**.

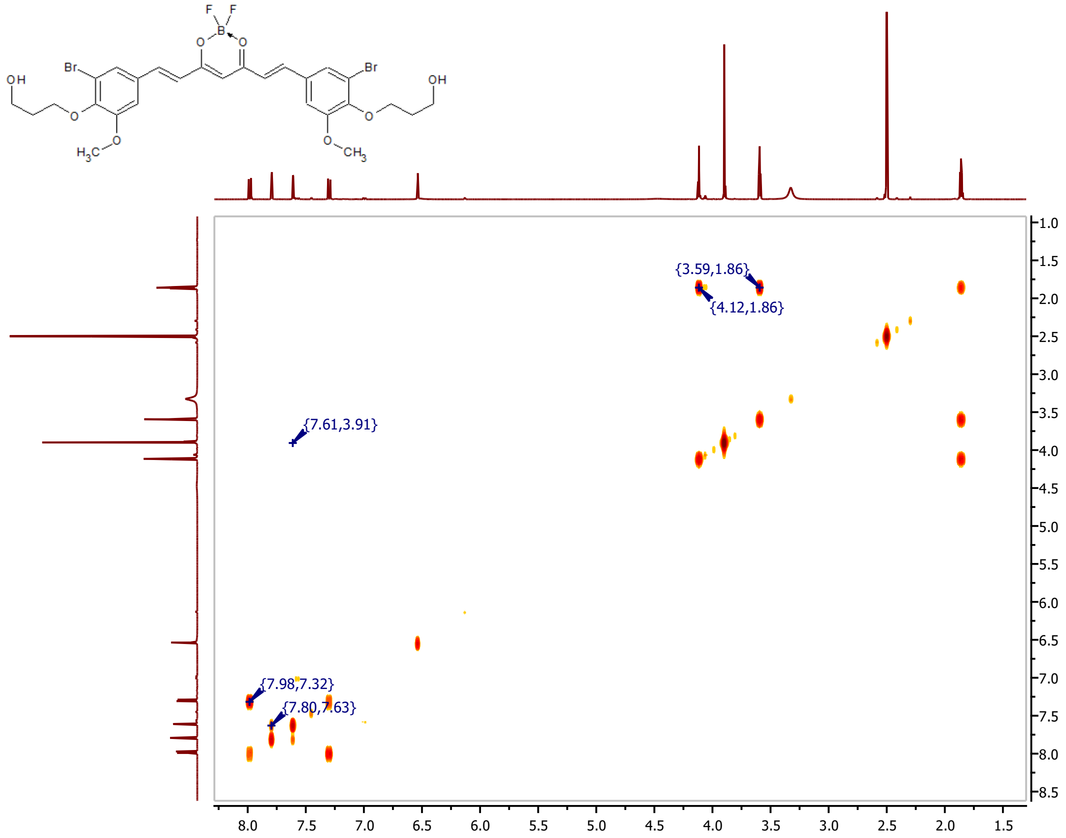

Figure S65. ^{1}H-^{13}C HSQC of curcumin **5a**.

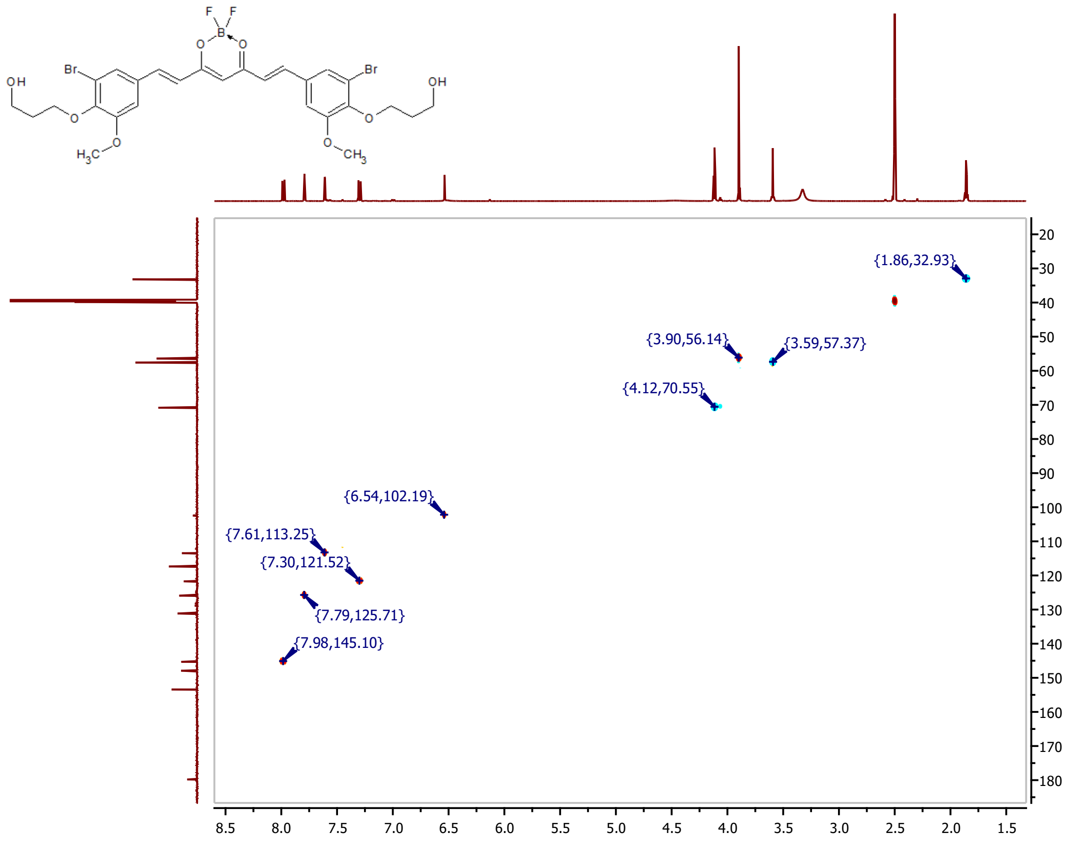

Figure S66. ^{1}H-^{13}C HMBC of curcumin **5a.**

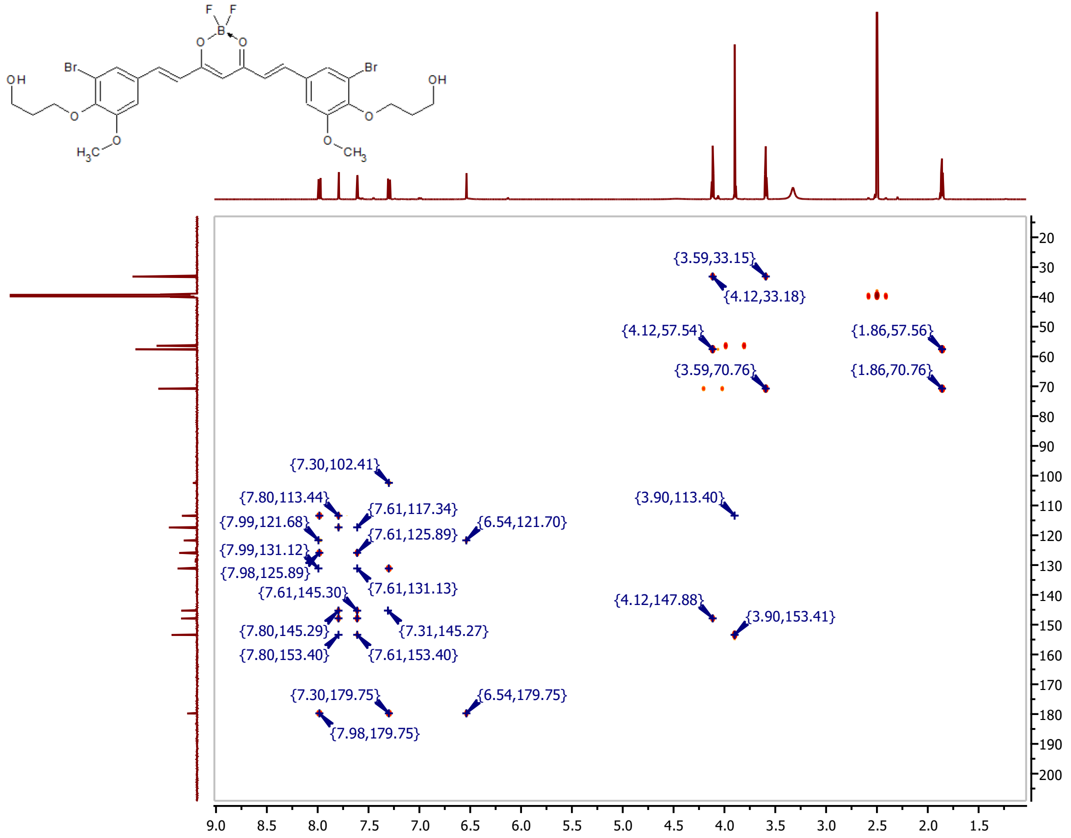

Figure S67. NMR experiments of compound **5b**.

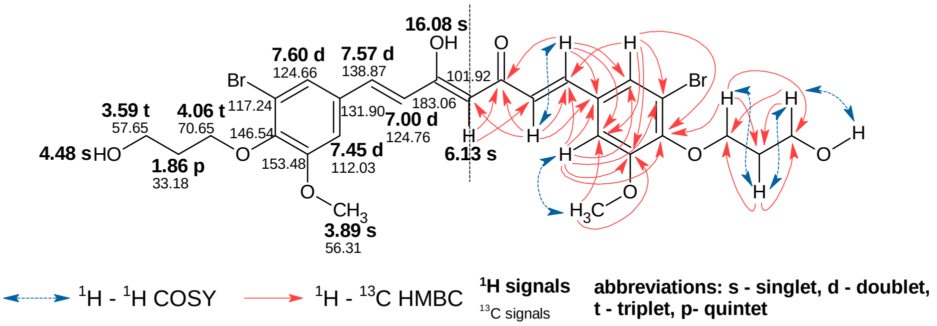

Figure S68. ^{1}H NMR of curcumin **5b**.

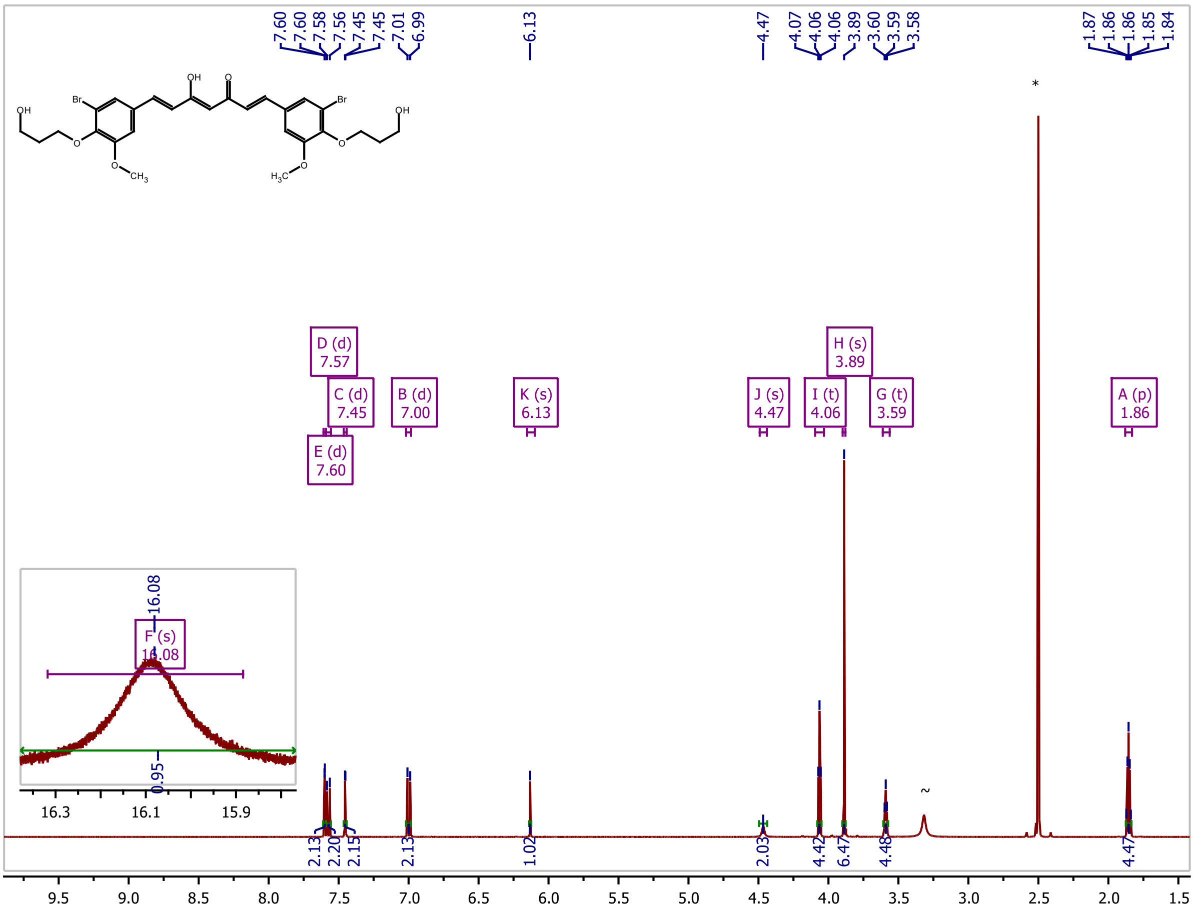

Figure S69. ^{13}C NMR of curcumin **5b**.

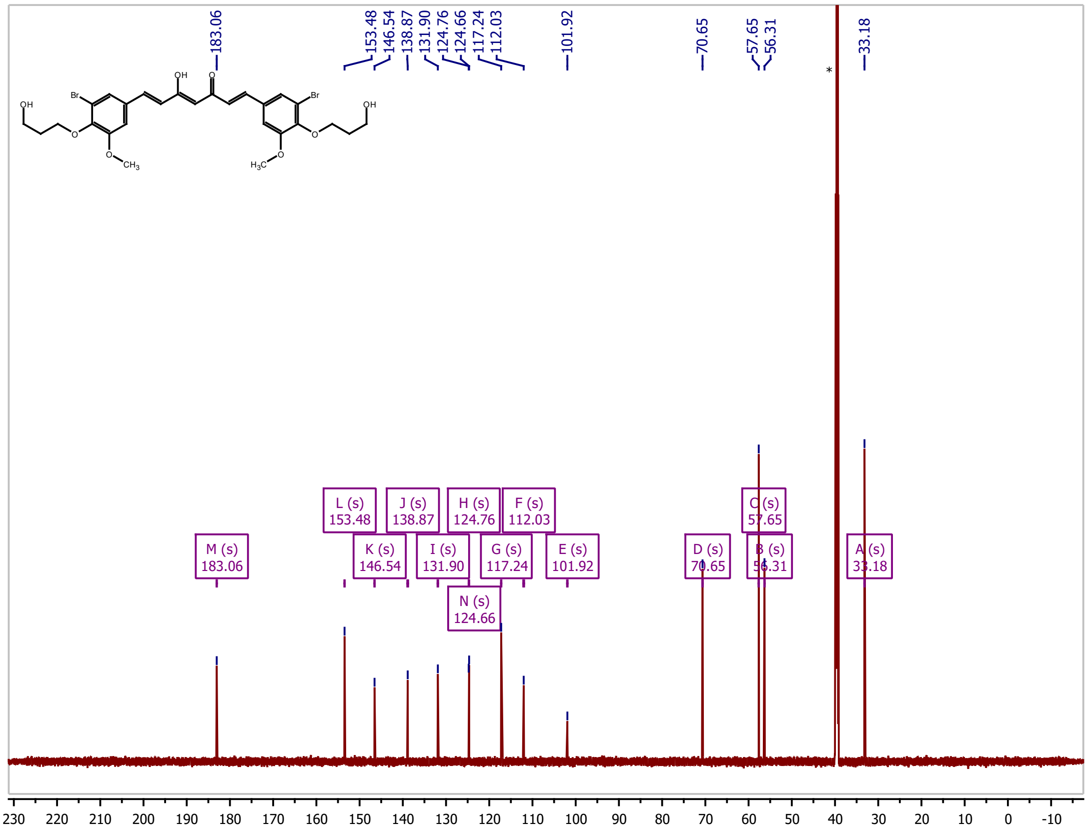

Figure S70. ^{1}H-^{1}H COSY of curcumin **5b**.

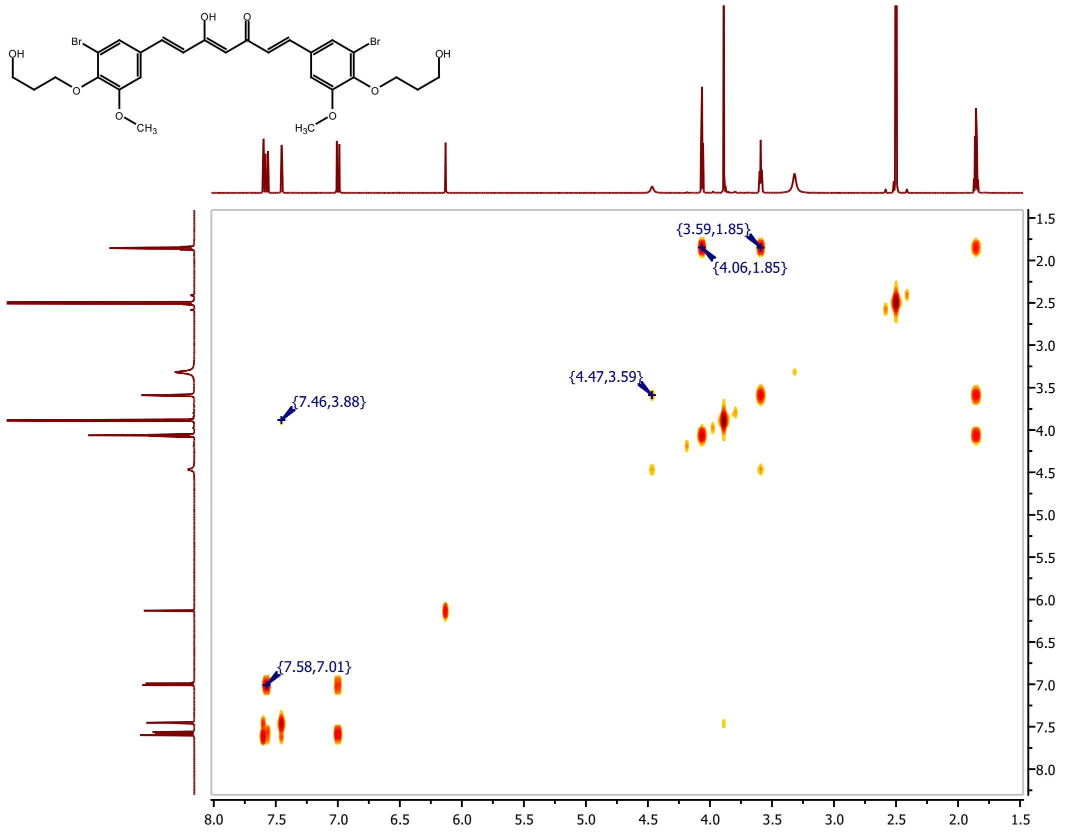

Figure S71. ^{1}H-^{13}C HSQC of curcumin **5b**.

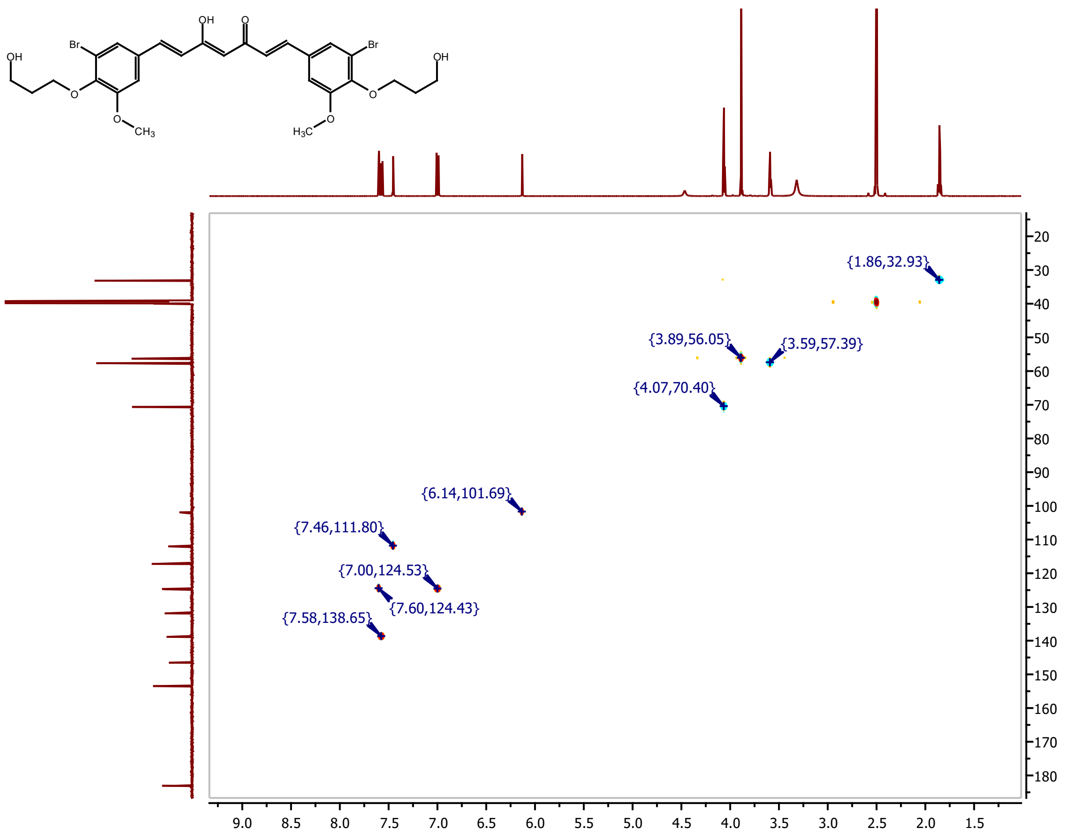

Figure S72. ^{1}H-^{13}C HMBC of curcumin **5b.**

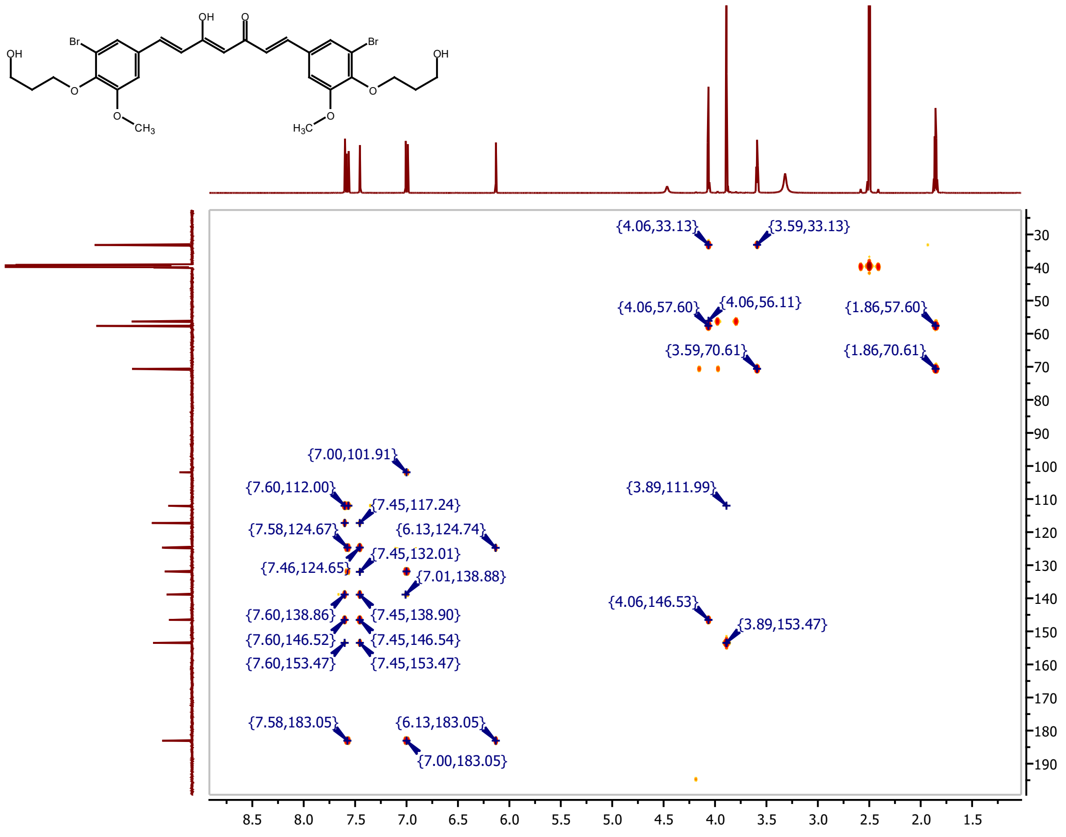

Figure S73. NMR experiments of compound **6a**.

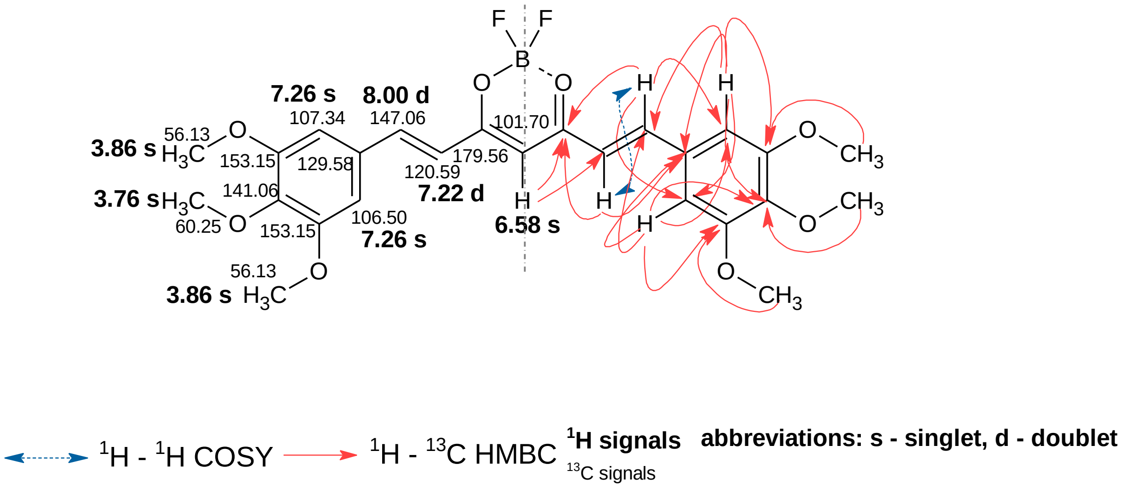

Figure S74. ¹H NMR of curcumin **6a**.

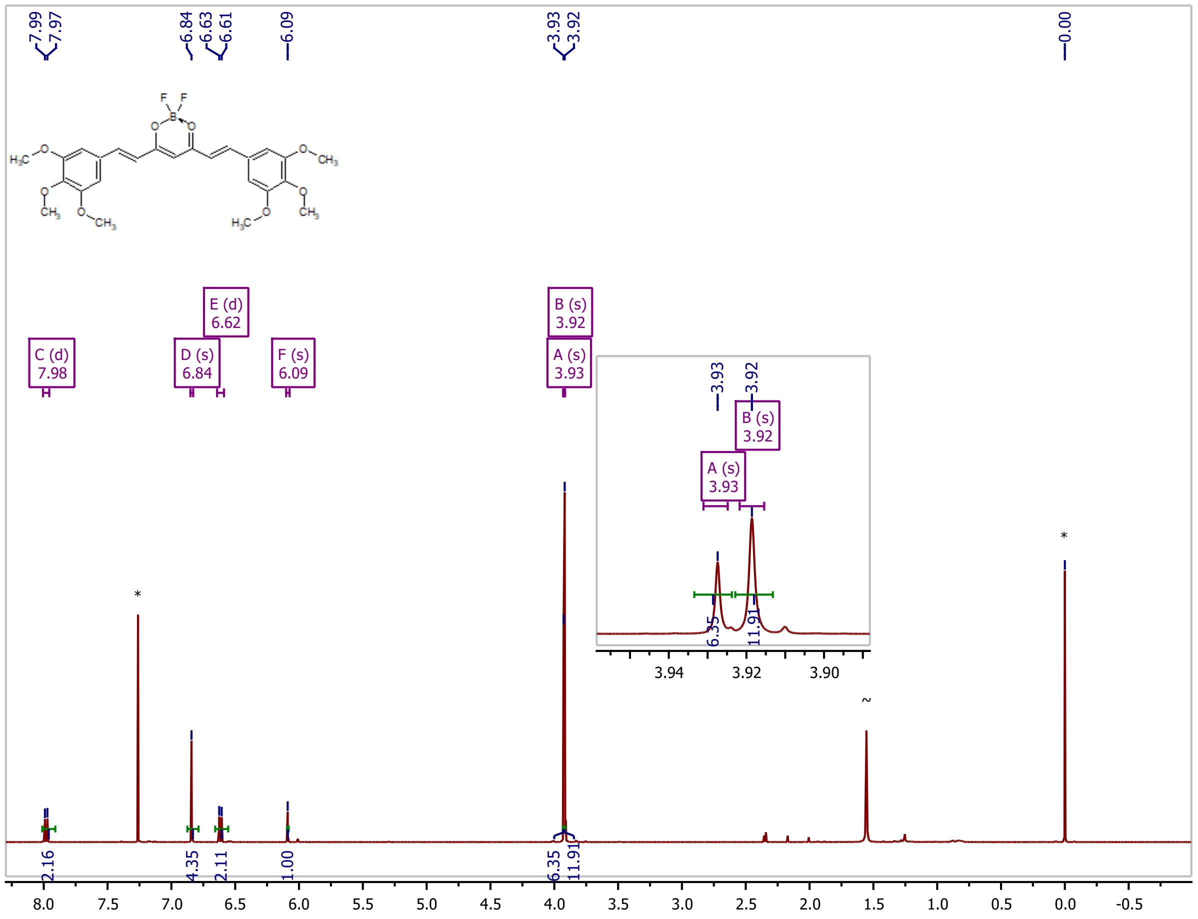

Figure S75. ^{13}C NMR of curcumin **6a**.

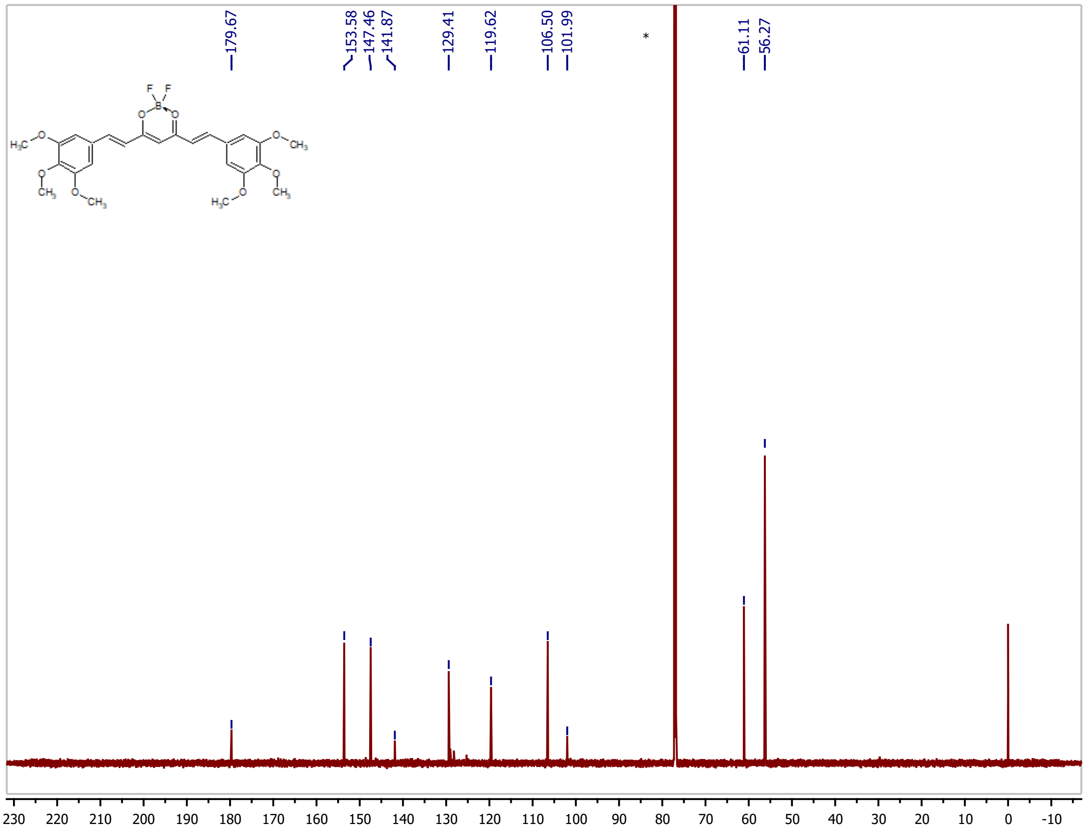

Figure S76. ^{1}H-^{1}H COSY of curcumin **6a**.

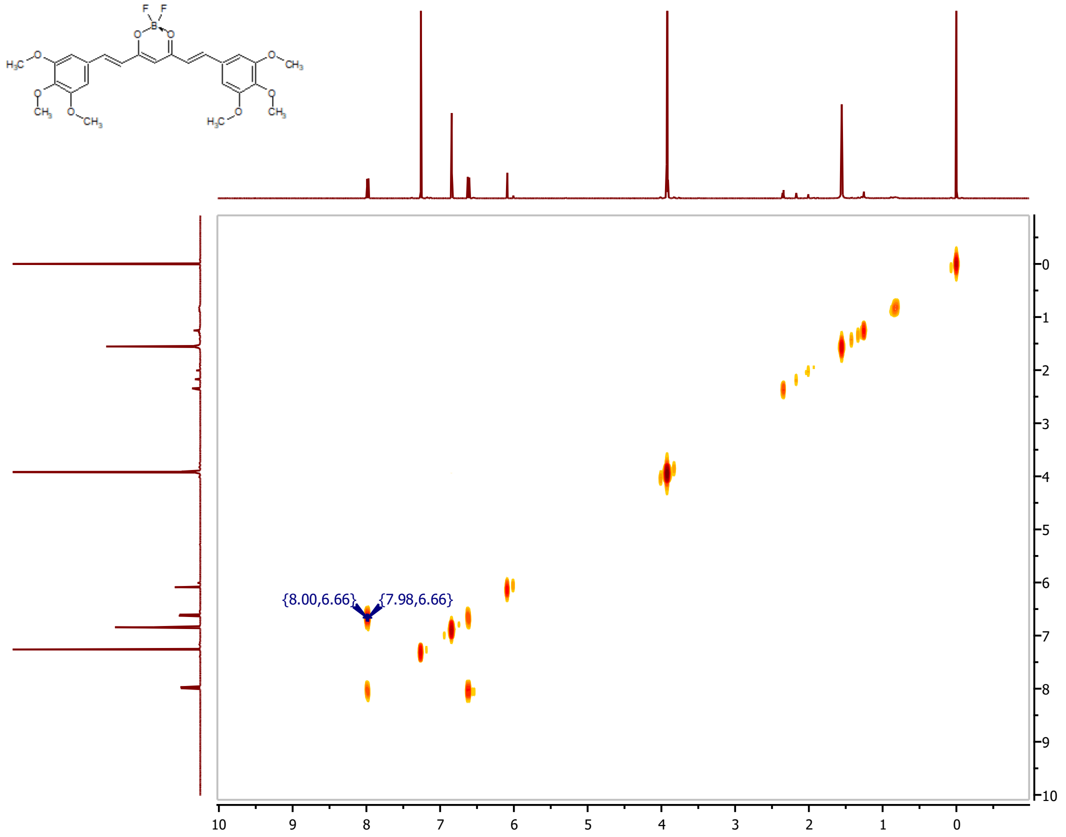

Figure S77. ^{1}H-^{13}C HSQC of curcumin **6a**.

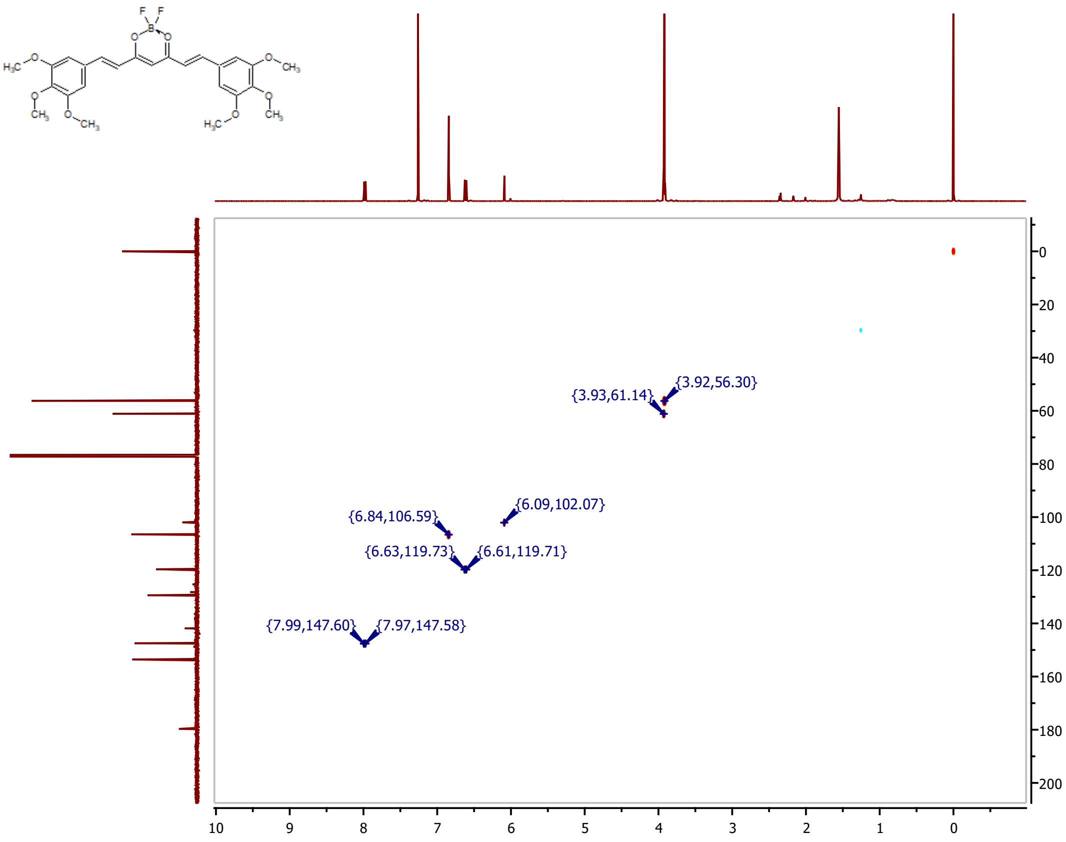

Figure S78. ^{1}H-^{13}C HMBC of curcumin **6a.**

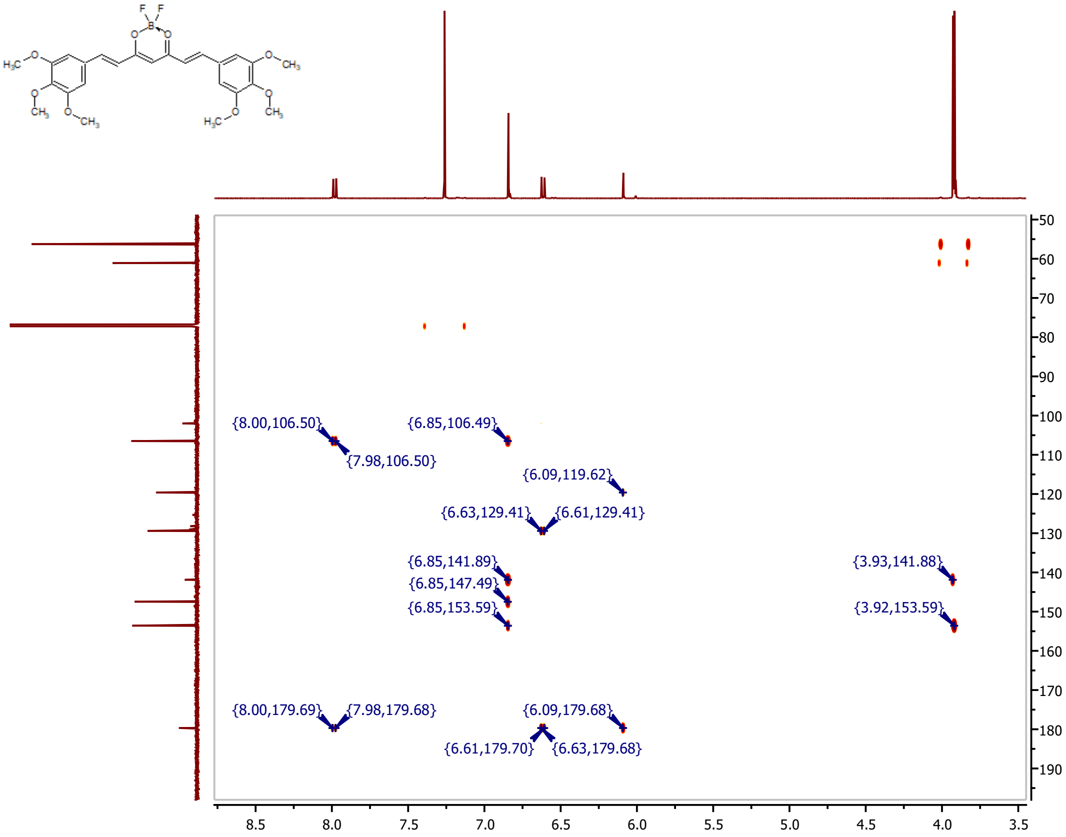

Figure S79. NMR experiments of compound **6b**.

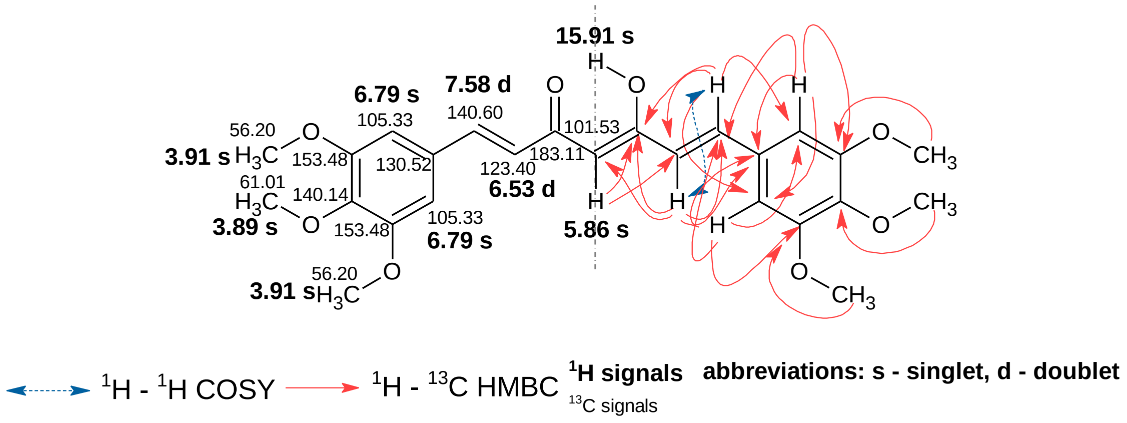

Figure S80. ¹H NMR of curcumin **6b**.

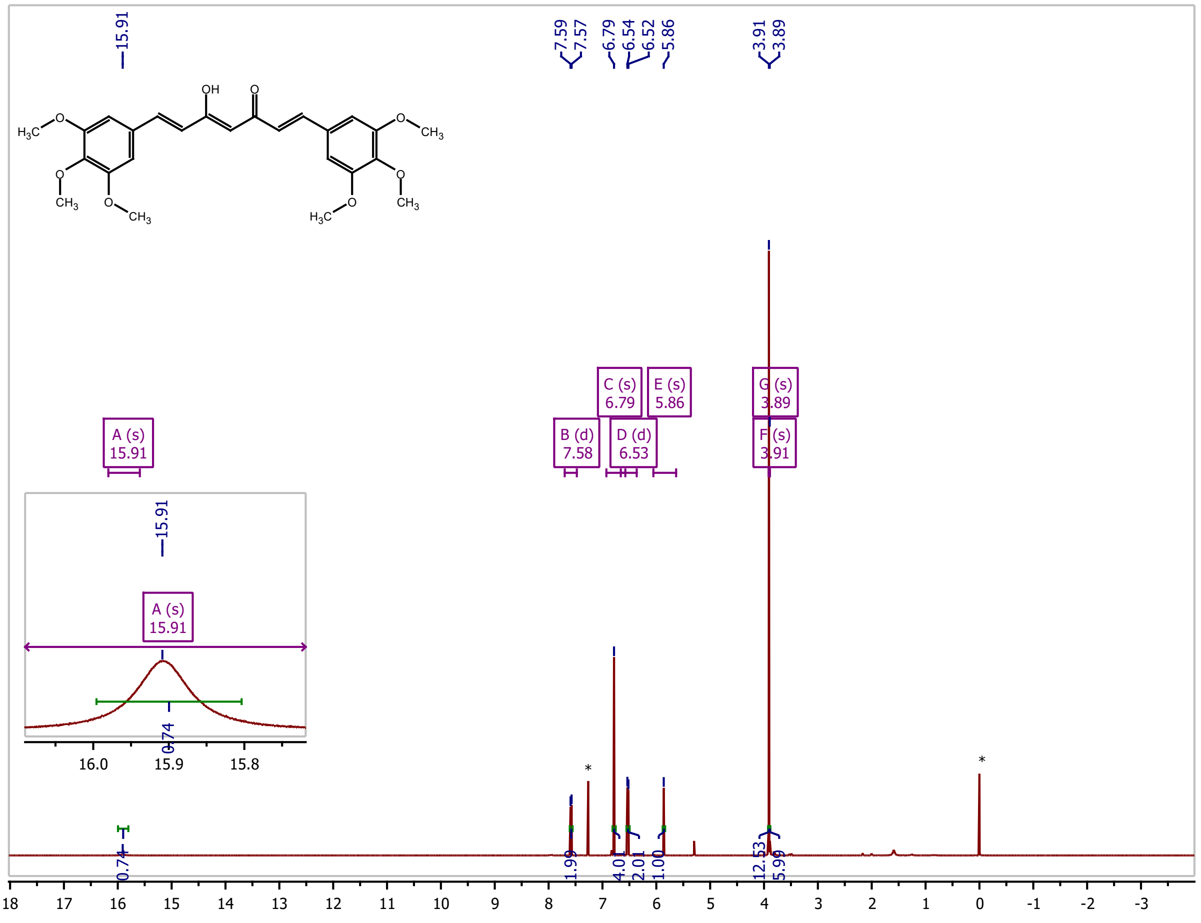

Figure S81. ^{13}C NMR of curcumin **6b**.

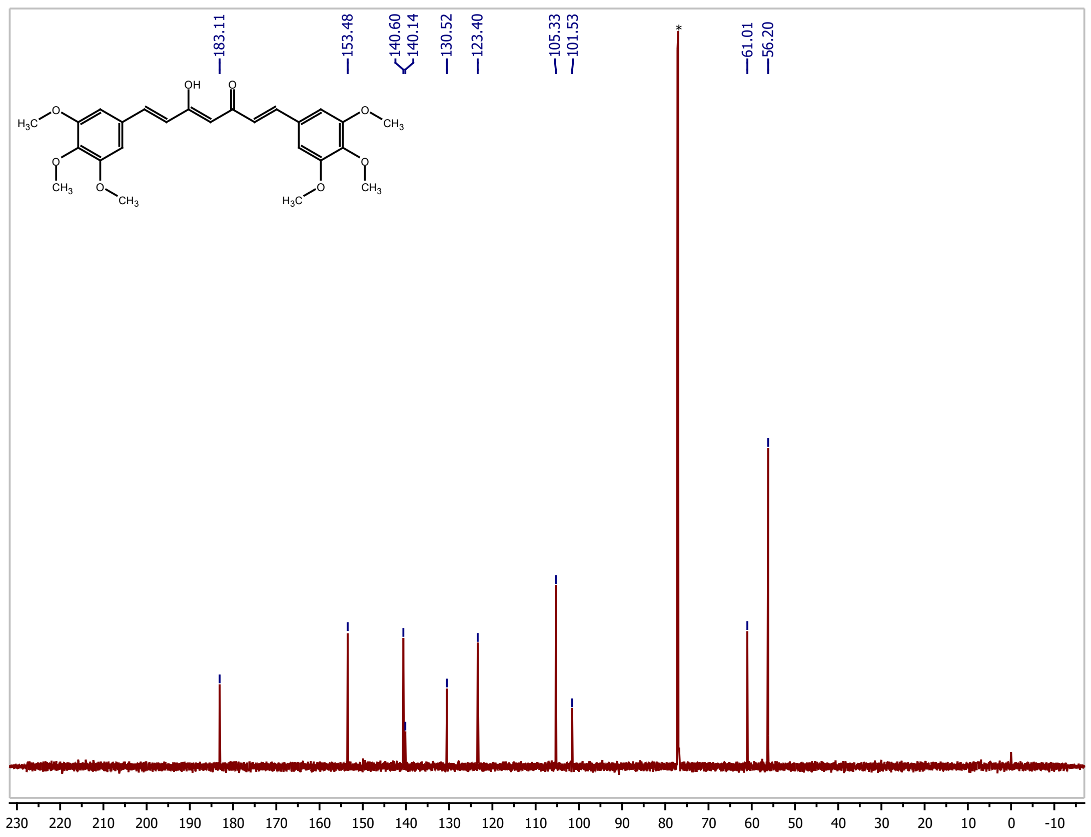

Figure S82. ^{1}H-^{1}H COSY of curcumin **6b**.

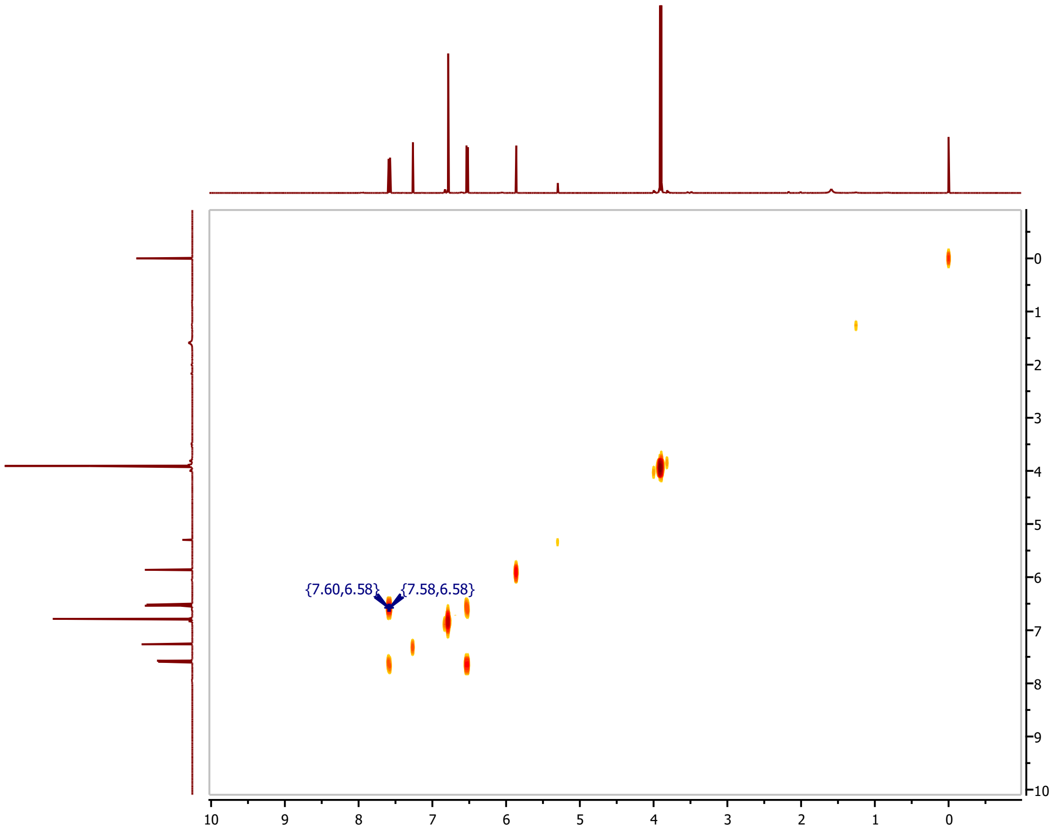

Figure S83. ^{1}H-^{13}C HSQC of curcumin **6b**.

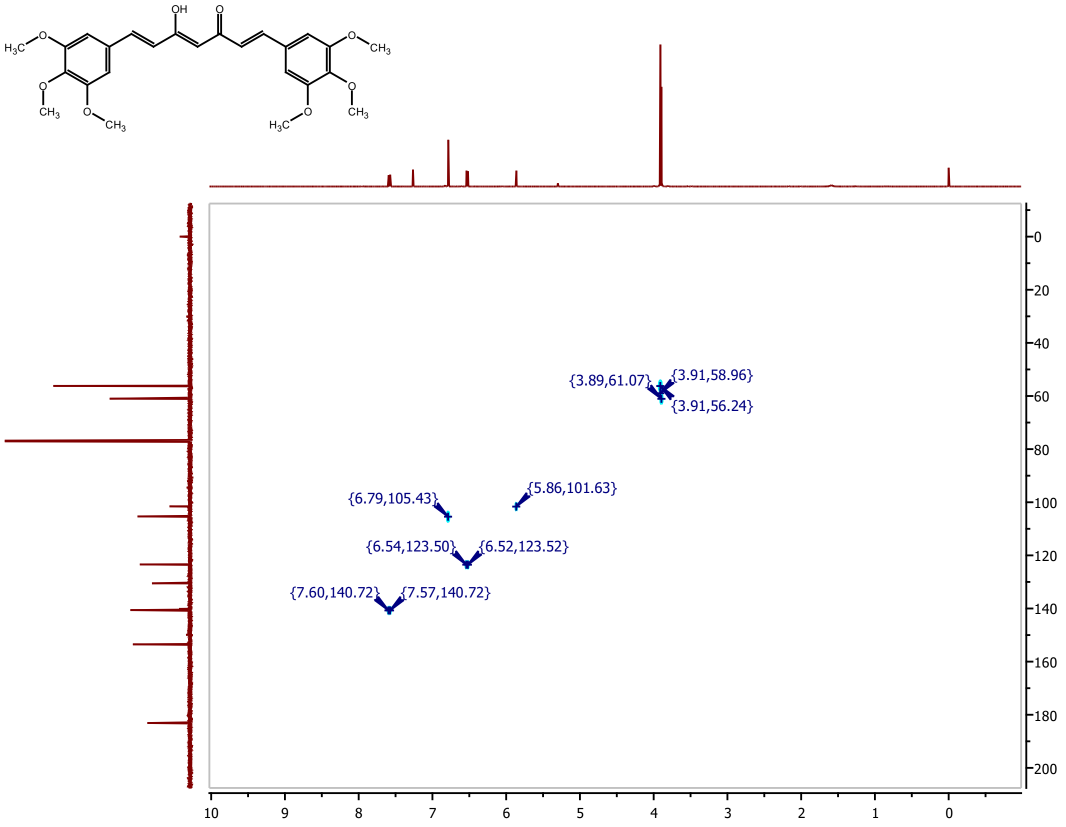

Figure S84. ^{1}H-^{13}C HMBC of curcumin **6b.**

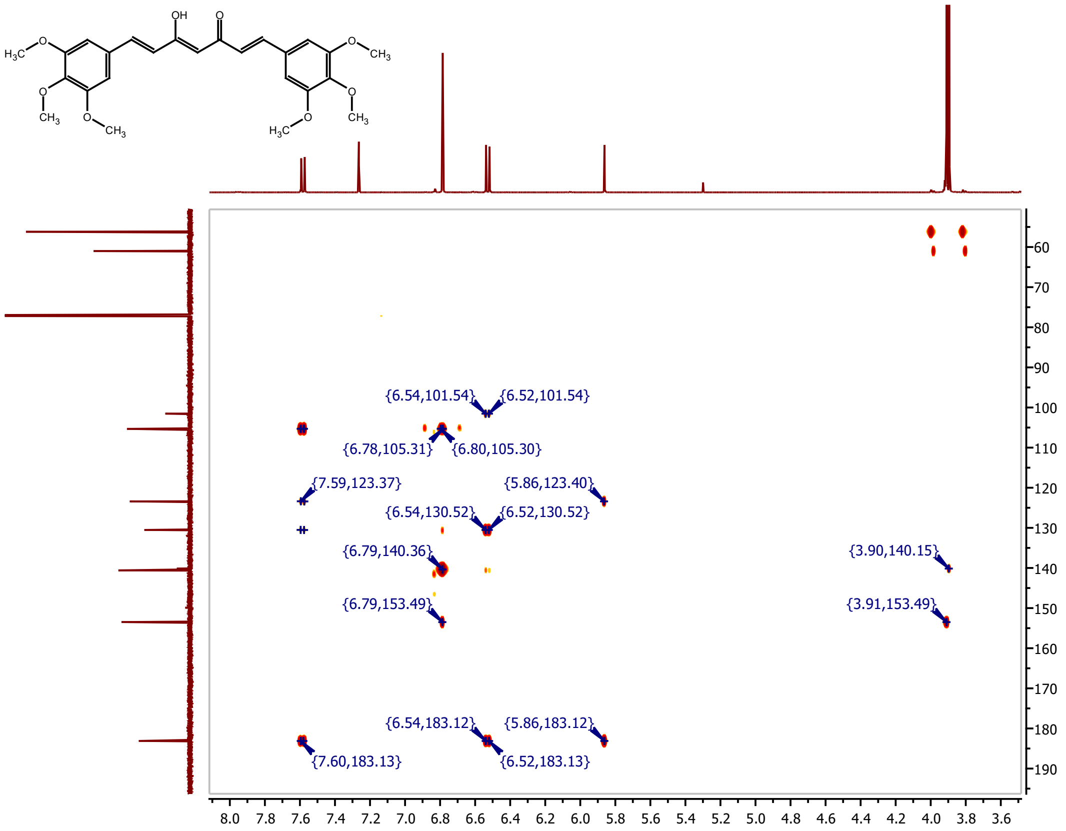

Figure S85. NMR experiments of compound **7a**

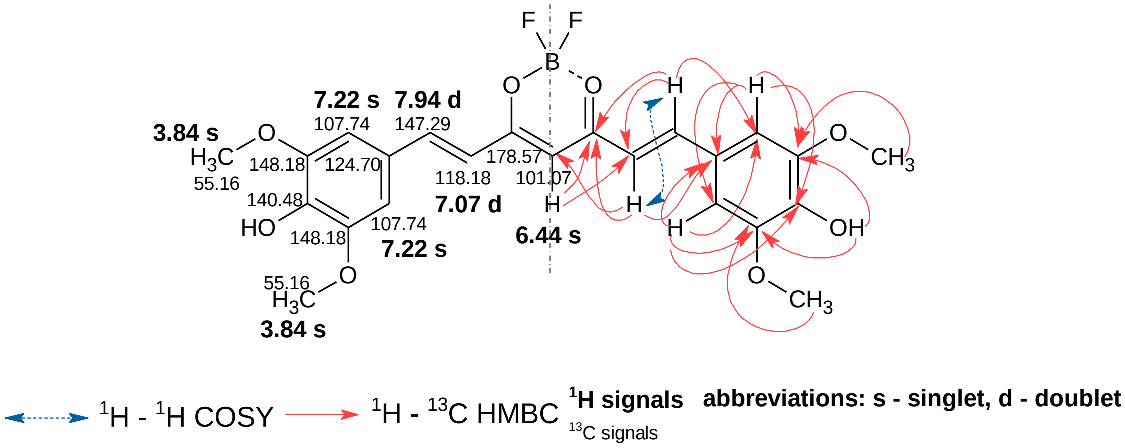

Figure S86. ¹H NMR of curcumin **7a**.

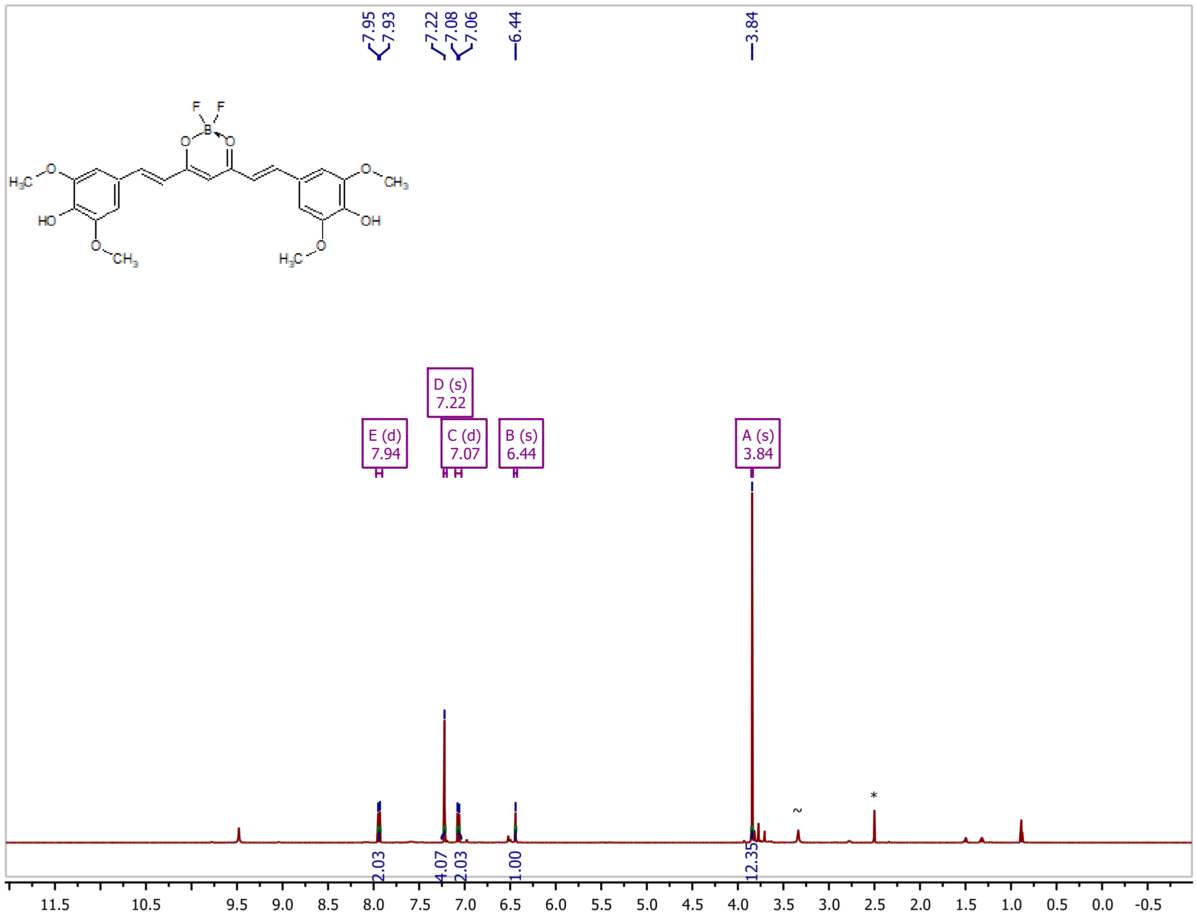

Figure S87. ^{13}C NMR of curcumin **7a**.

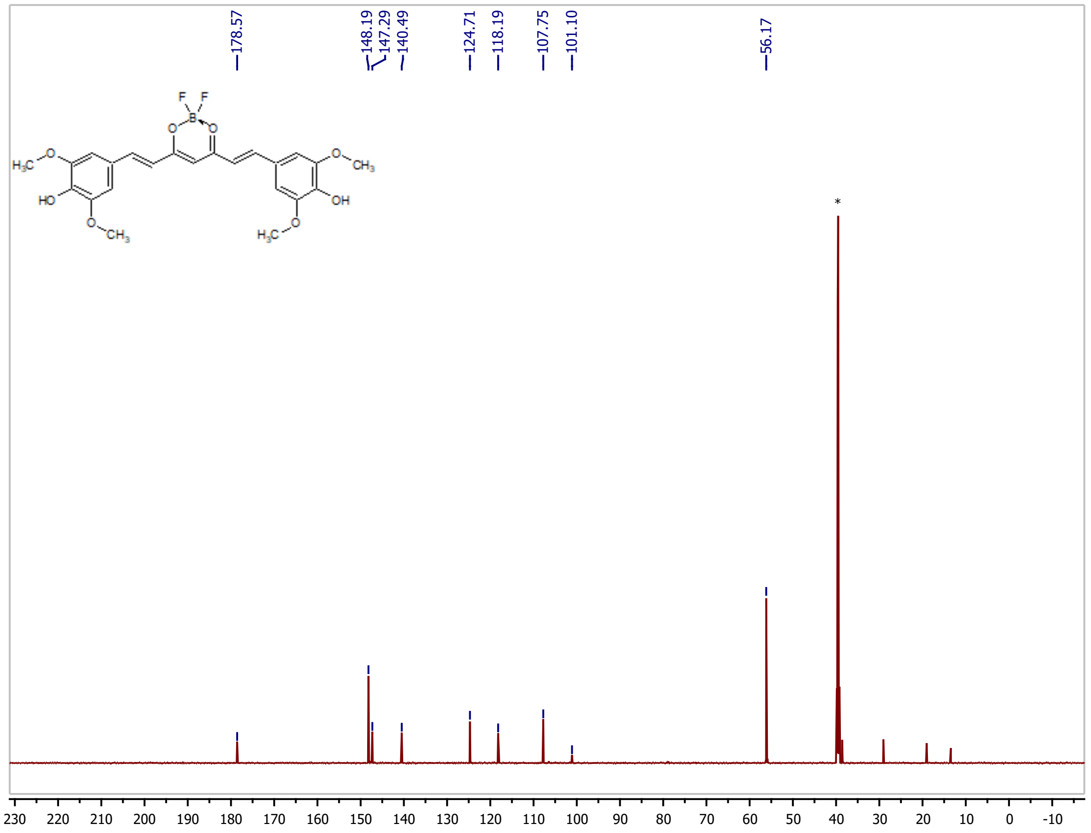

Figure S88. ^1H-^1H COSY of curcumin **7a**.

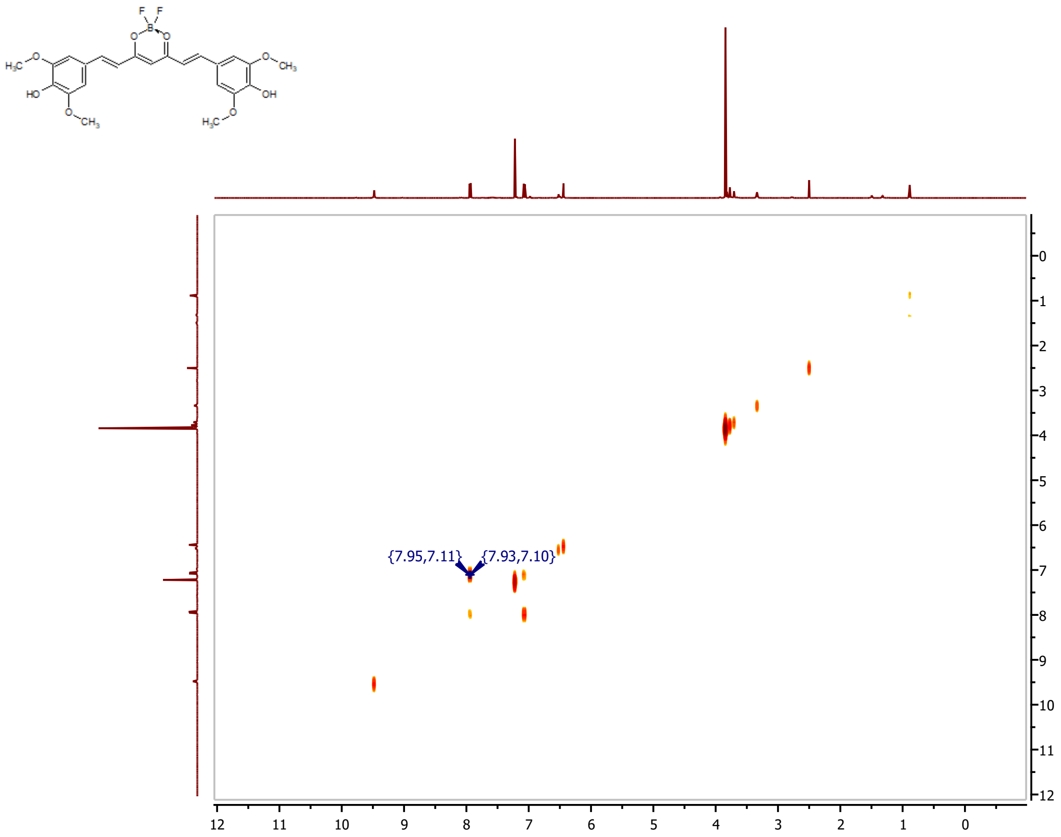

Figure S89. ^{1}H-^{13}C HSQC of curcumin **7a**.

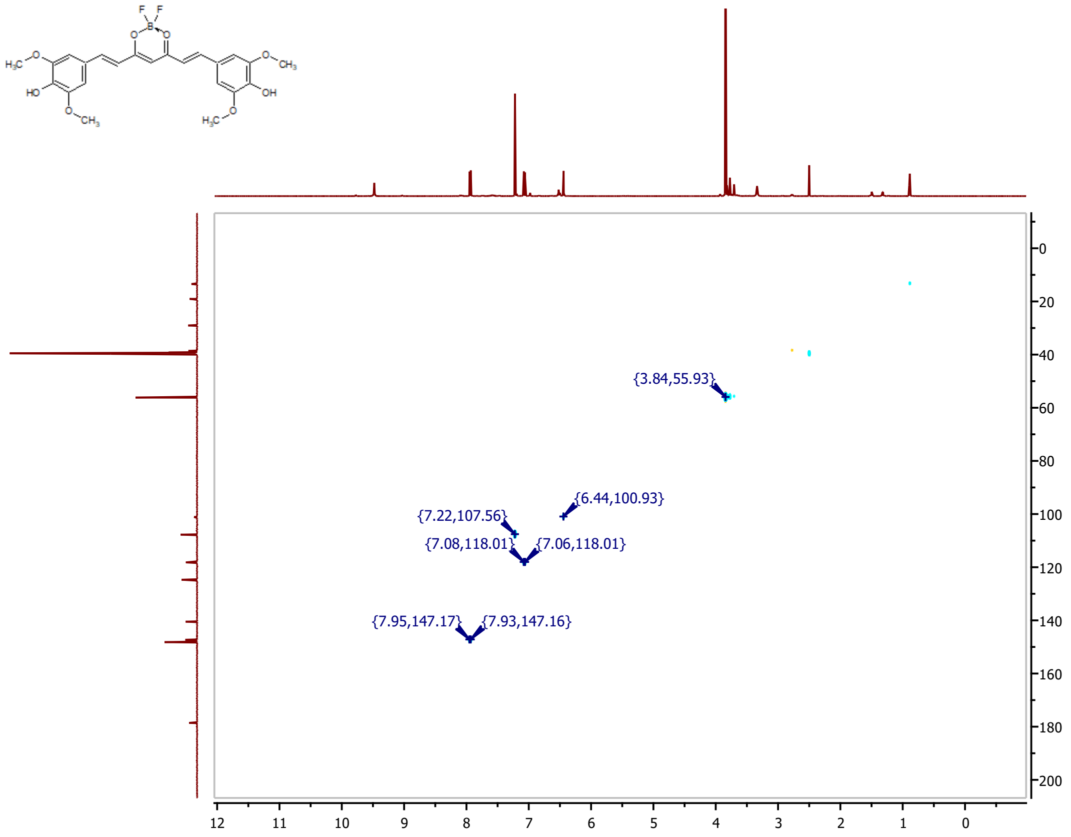

Figure S90. ^{1}H-^{13}C HMBC of curcumin **7a.**

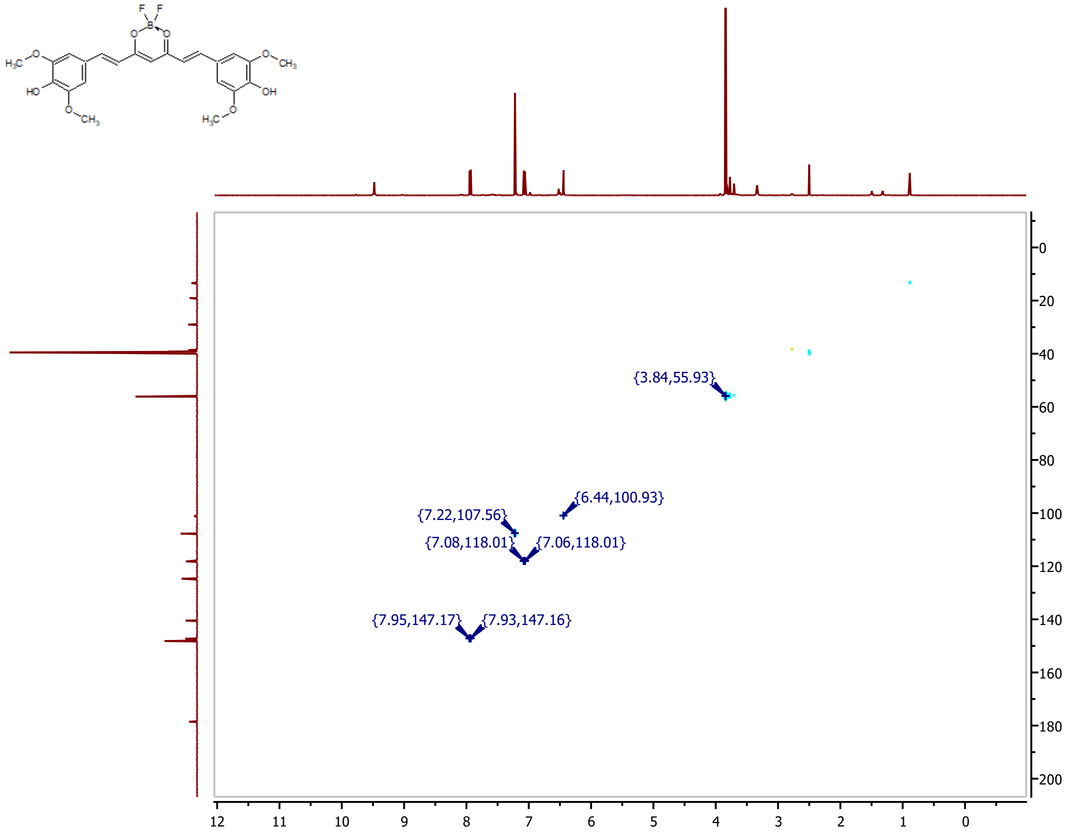

Figure S91. NMR experiments of compound **7b**

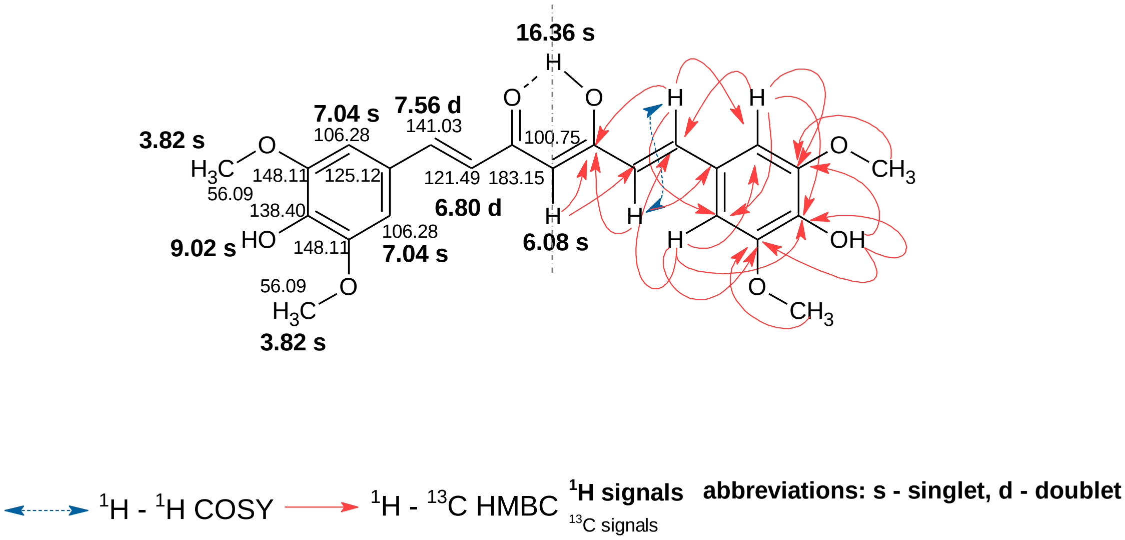

Figure S92. ¹H NMR of curcumin **7b**.

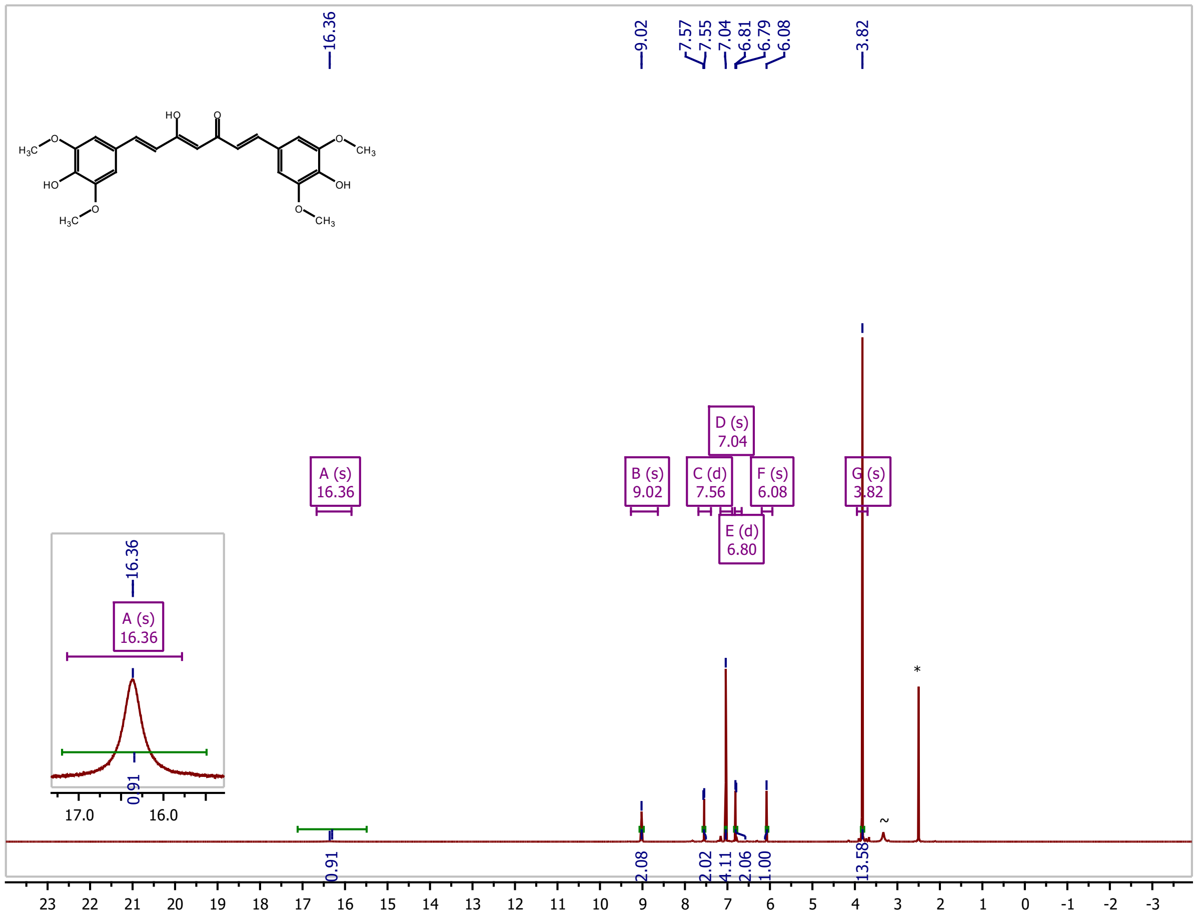

Figure S93. ^{13}C NMR of curcumin **7b**.

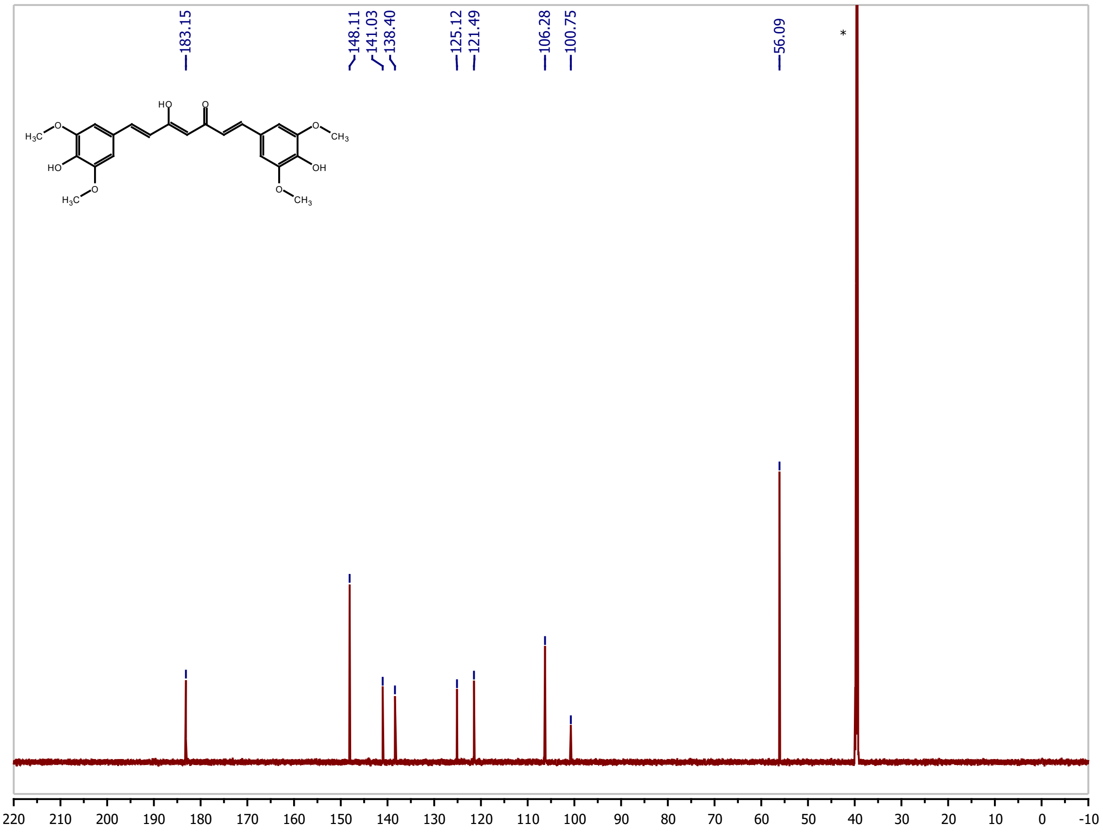

Figure S94. ^{1}H-^{1}H COSY of curcumin **7b**.

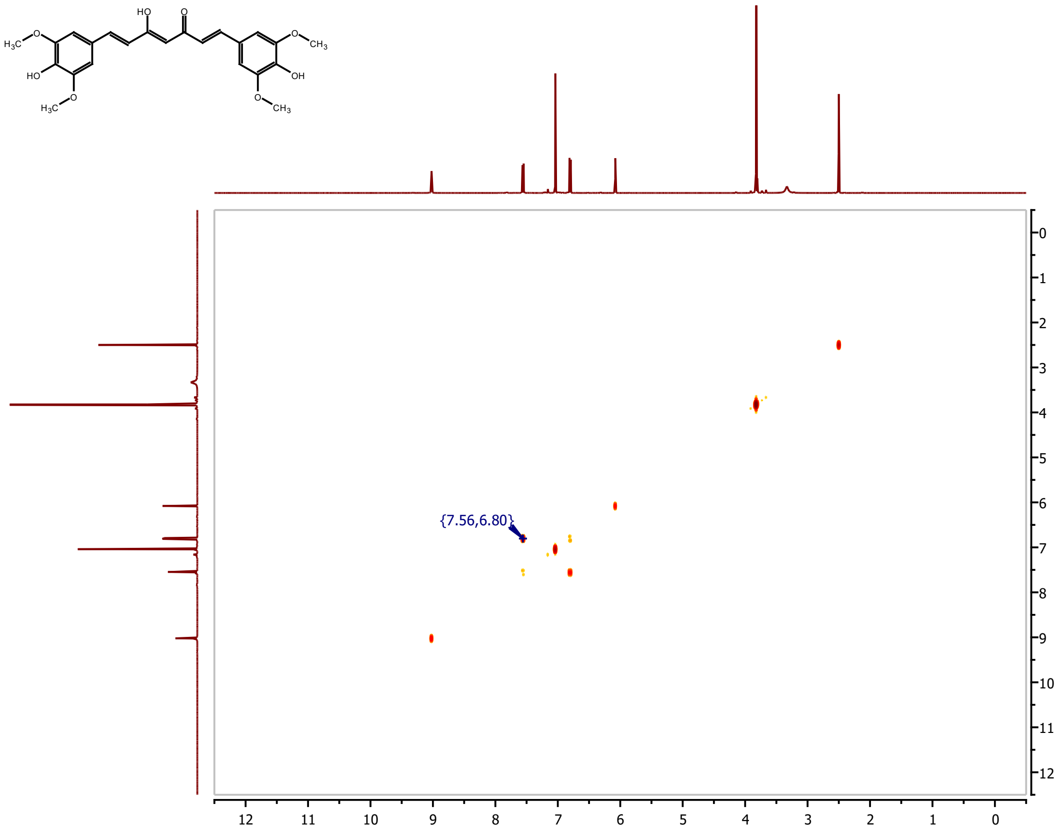

Figure S95. ^{1}H-^{13}C HSQC of curcumin **7b**.

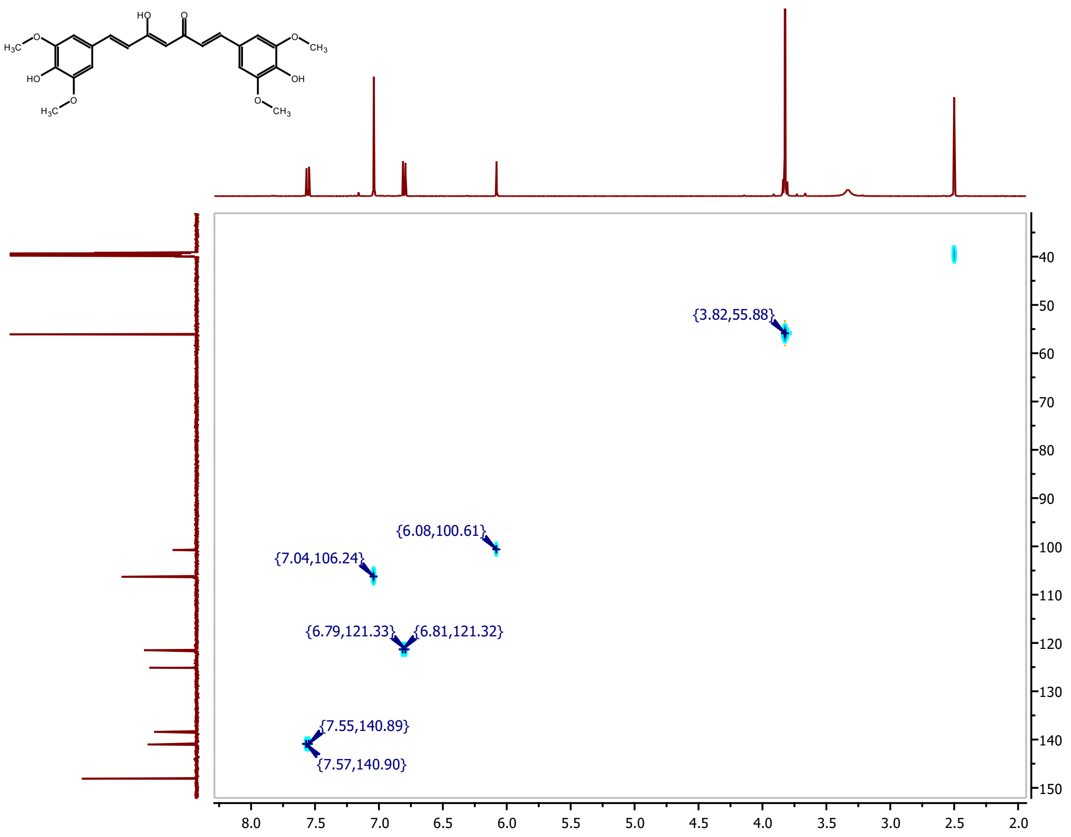

Figure S96. ^{1}H-^{13}C HMBC of curcumin **7b**

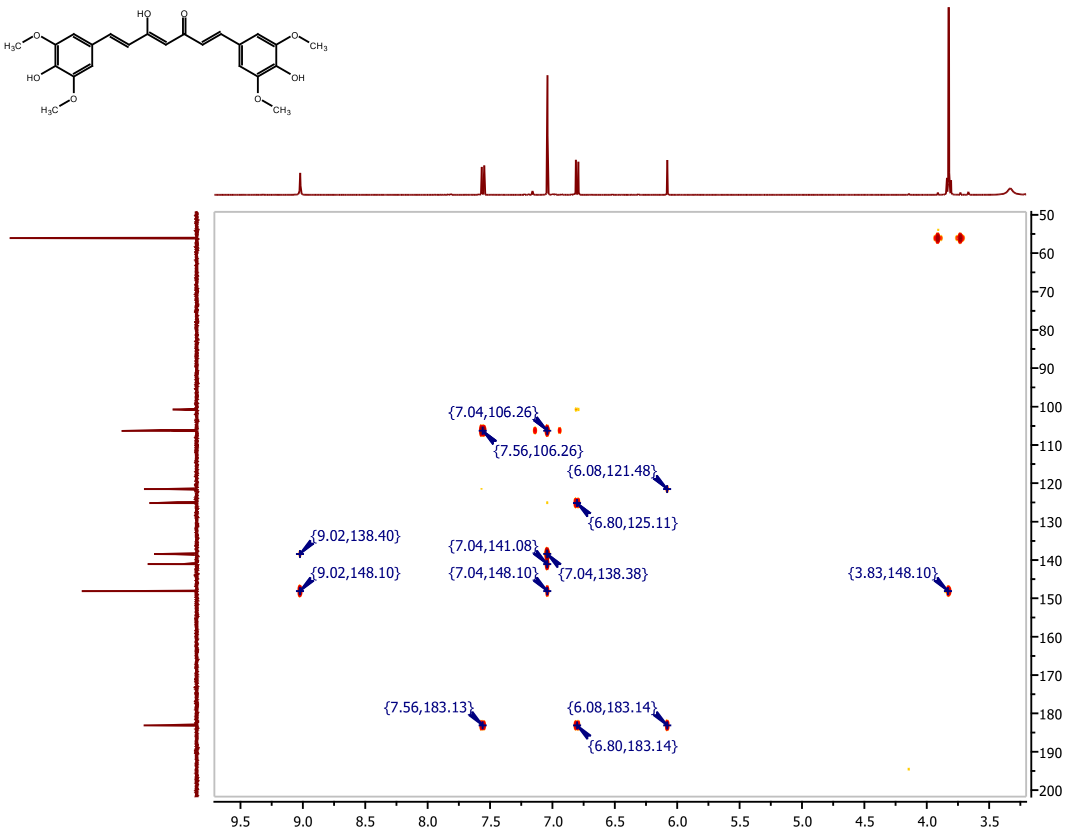

Figure S97. NMR experiments of compound **8a**

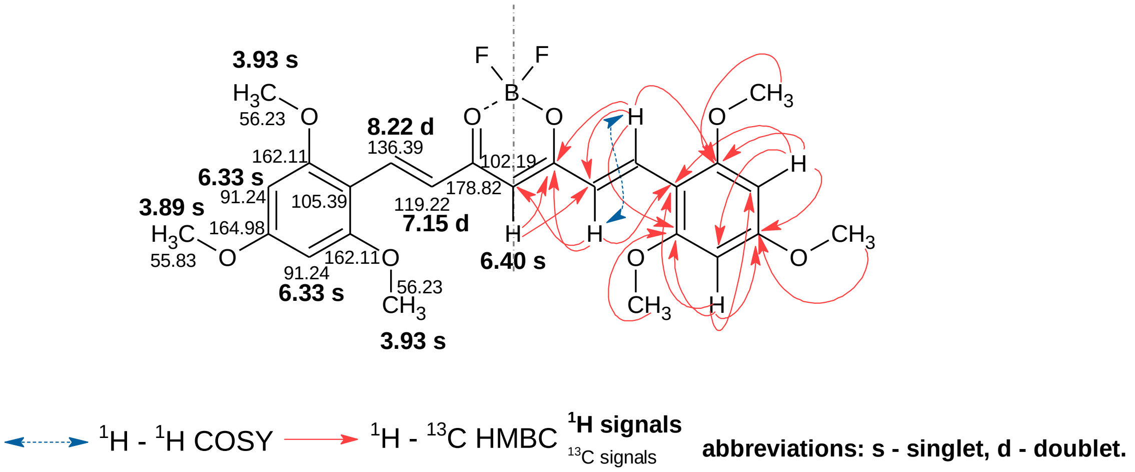

Figure S98. ¹H NMR of curcumin **8a**.

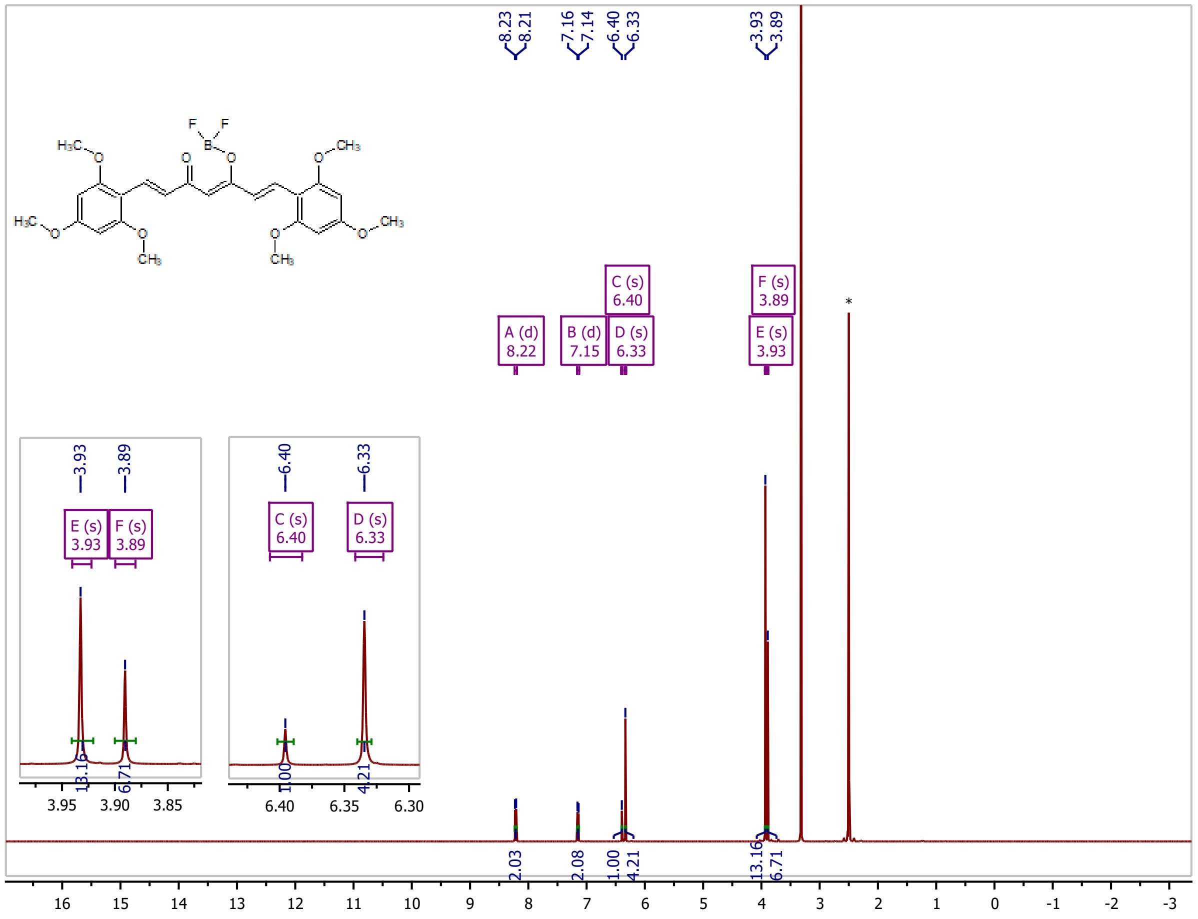

Figure S99. ^{13}C NMR of curcumin **8a**.

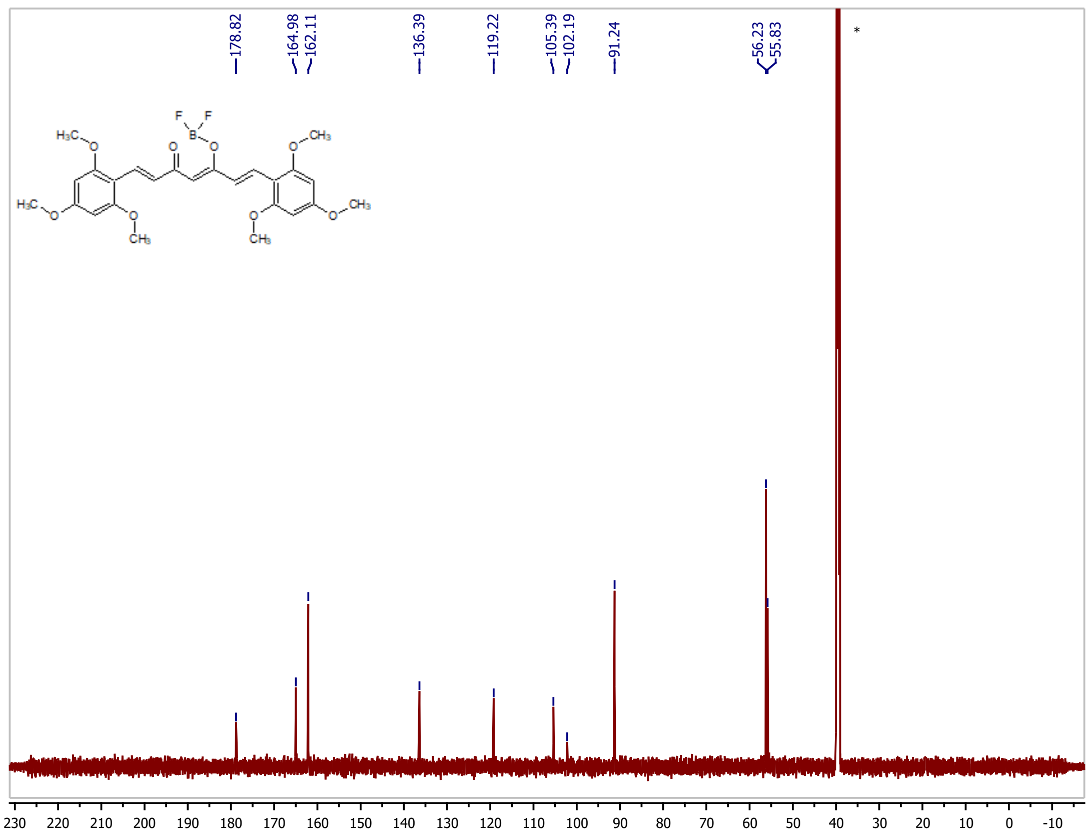

Figure S100. ^{1}H-^{1}H COSY of curcumin **8a**.

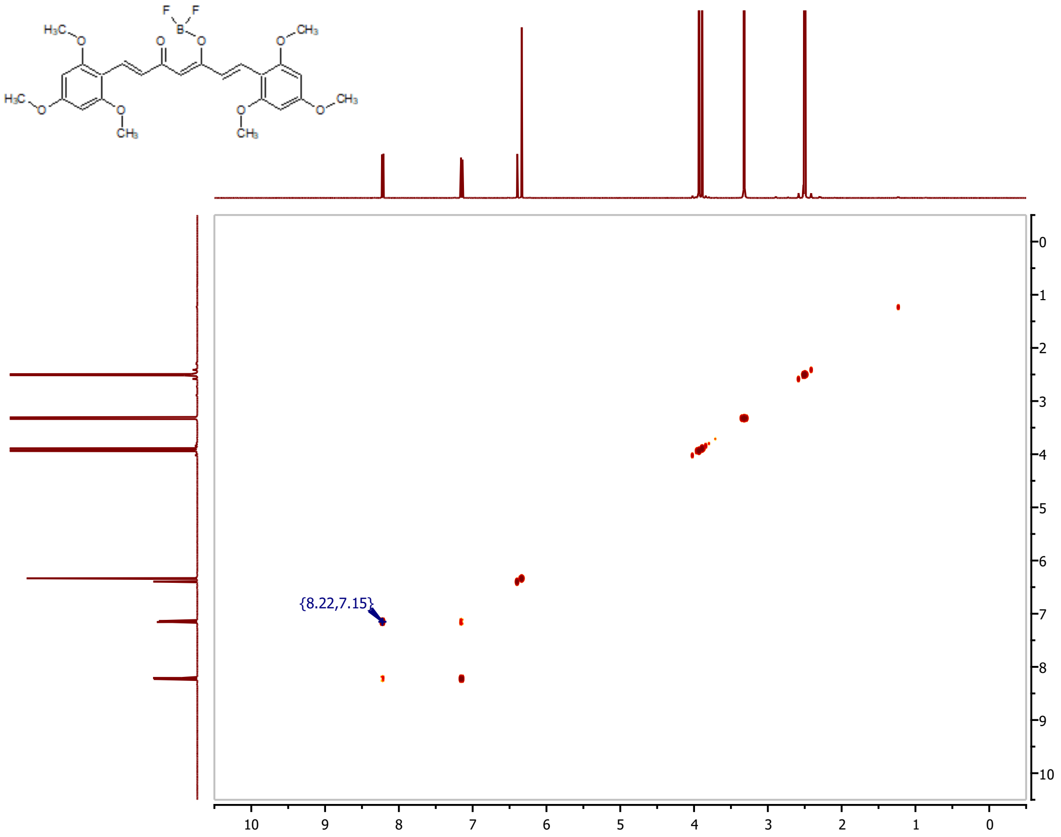

Figure S101. ^{1}H-^{13}C HSQC of curcumin **8a**.

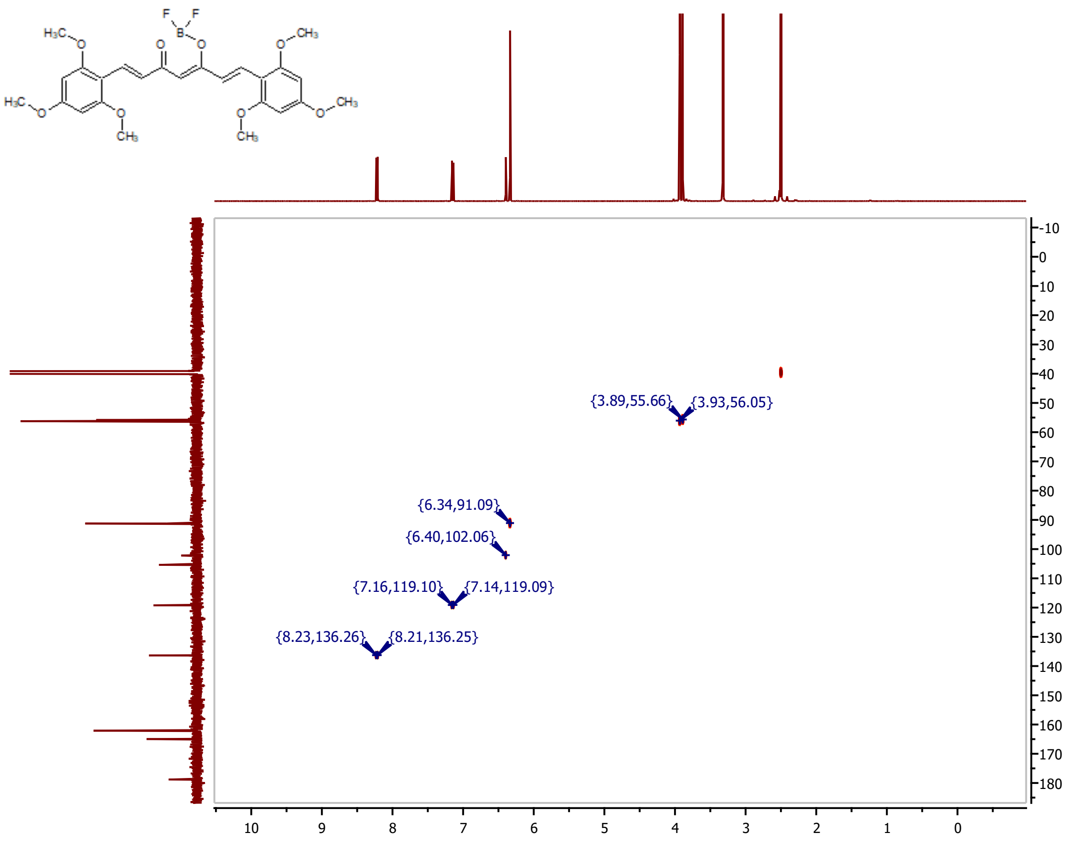

Figure S102. ^{1}H-^{13}C HMBC of curcumin **8a**

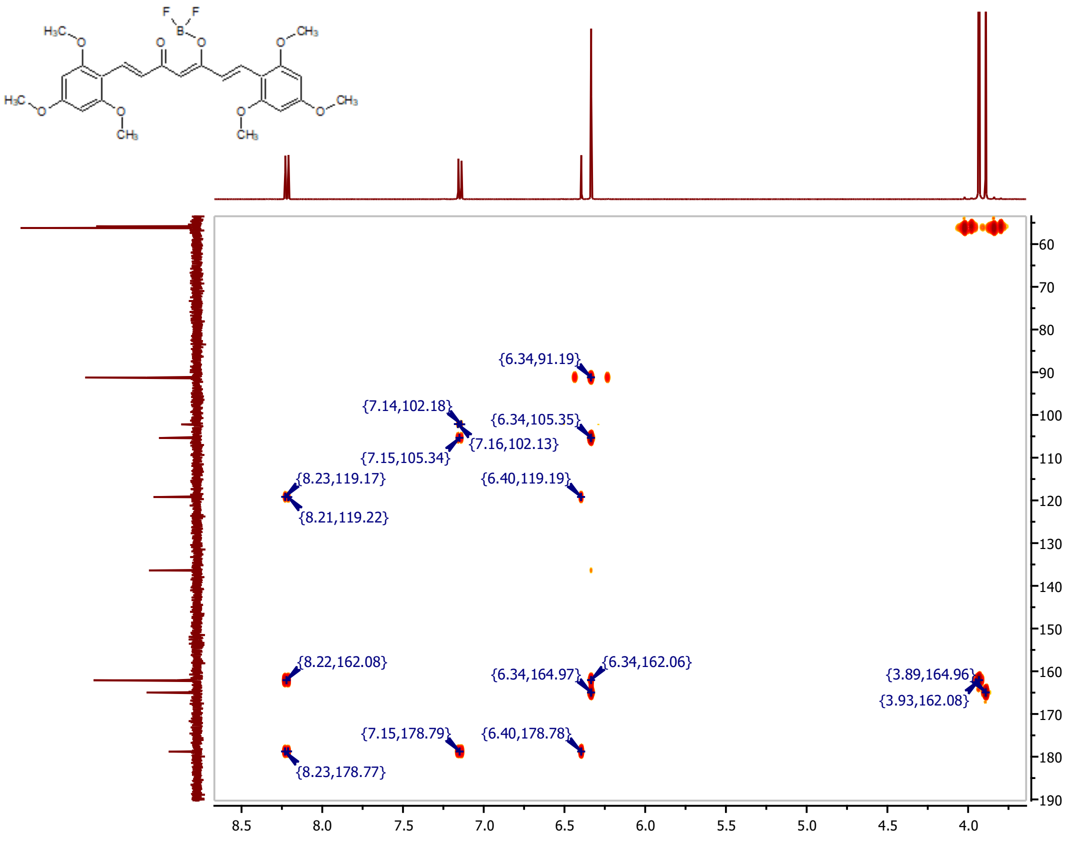

Figure S103. NMR experiments of compound **8b**

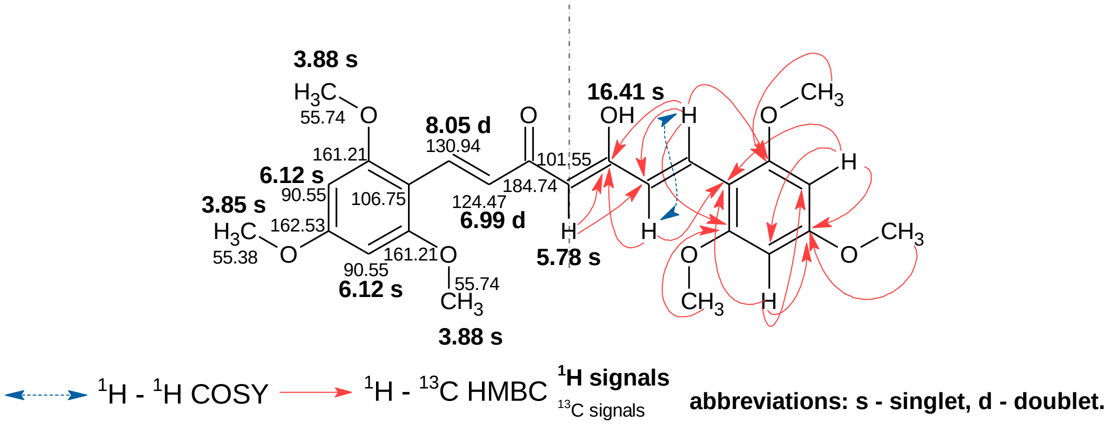

Figure S104. ^{1}H NMR of curcumin **8b**.

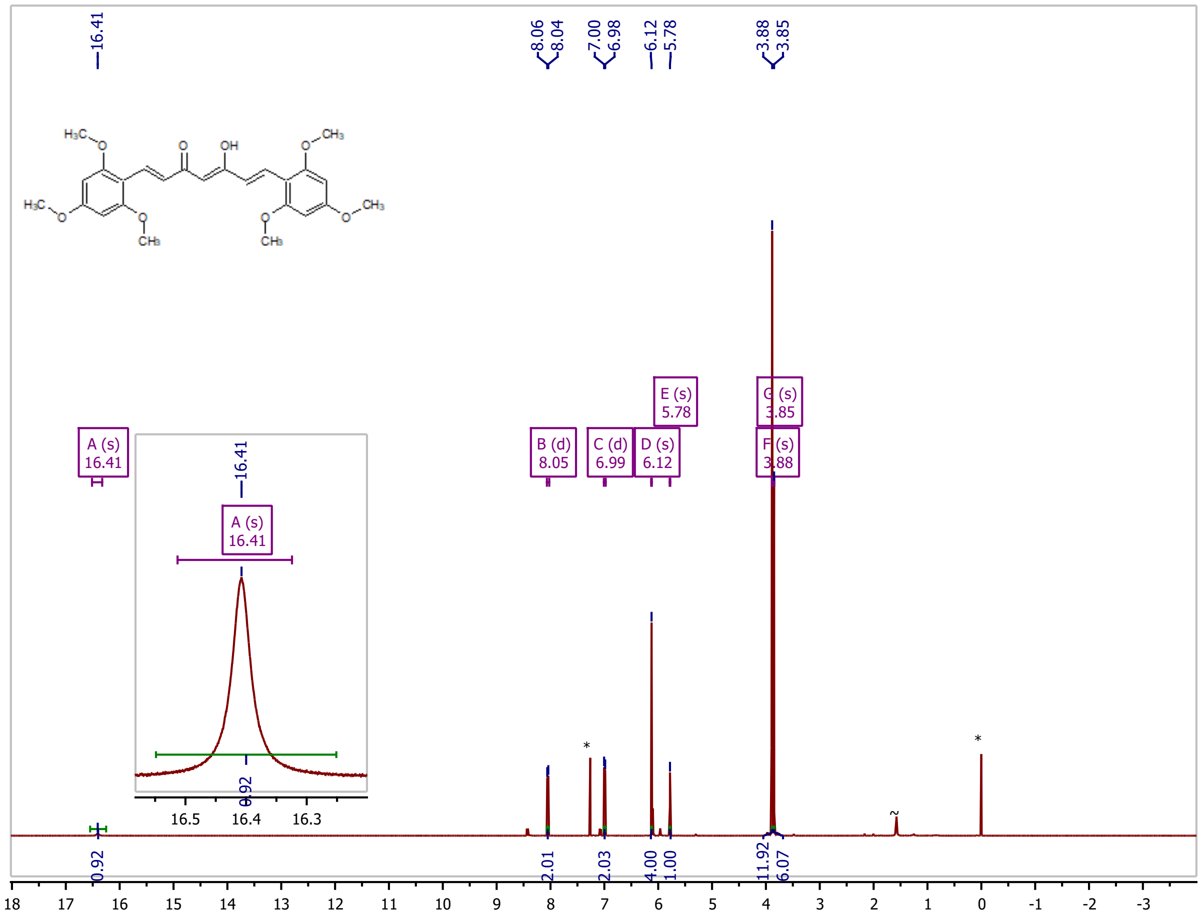

Figure S105. ^{13}C NMR of curcumin **8b**.

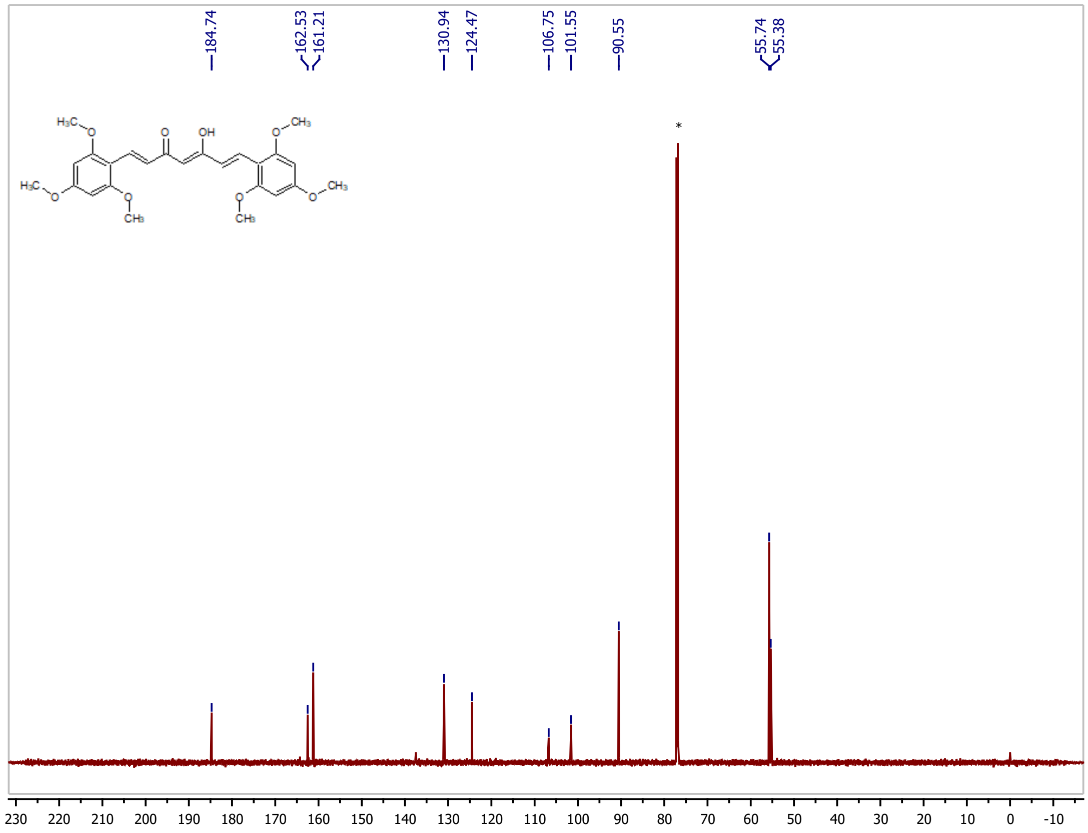

Figure S106. ^{1}H-^{1}H COSY of curcumin **8b**.

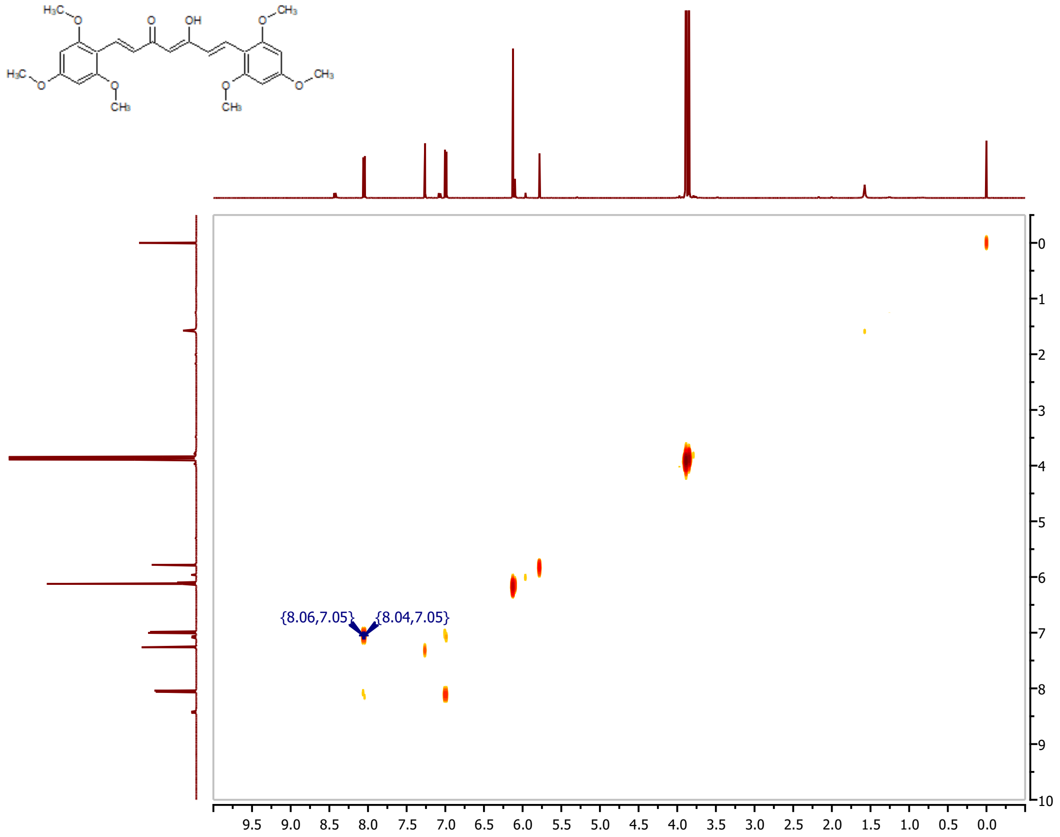

Figure S107. ^{1}H-^{13}C HSQC of curcumin **8b**.

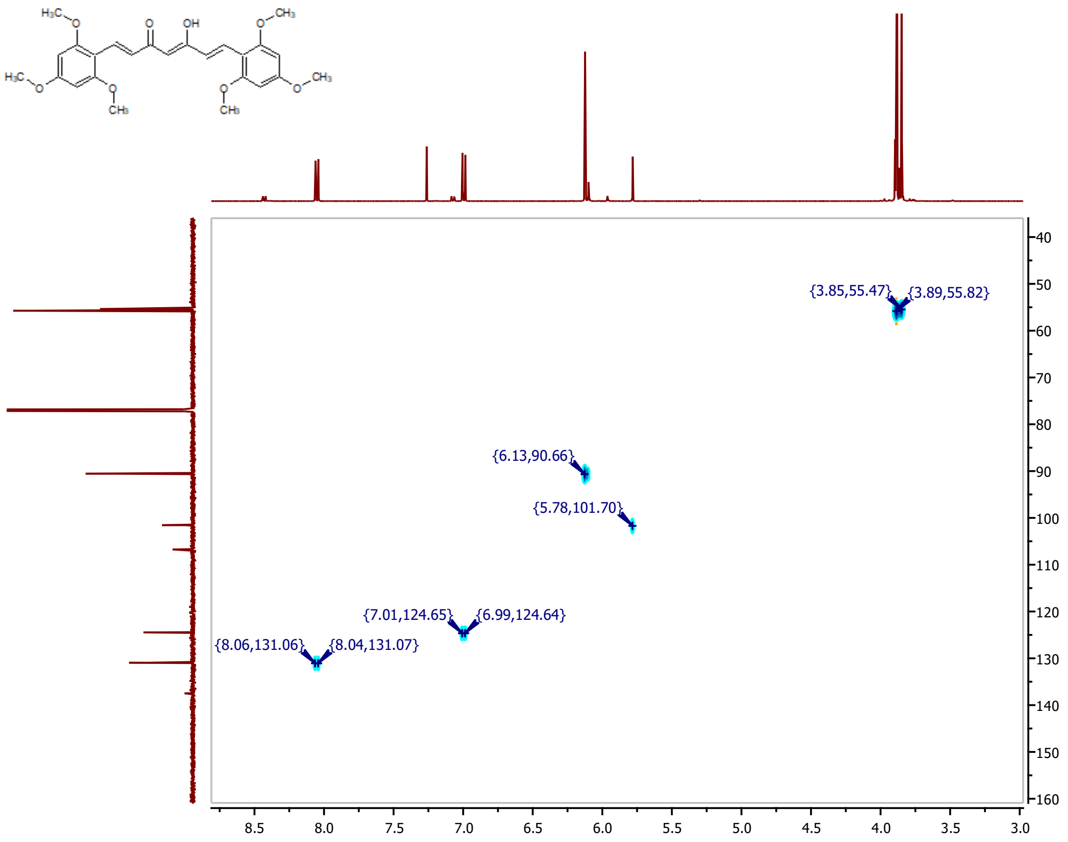

Figure S108. ^{1}H-^{13}C HMBC of curcumin **8b**

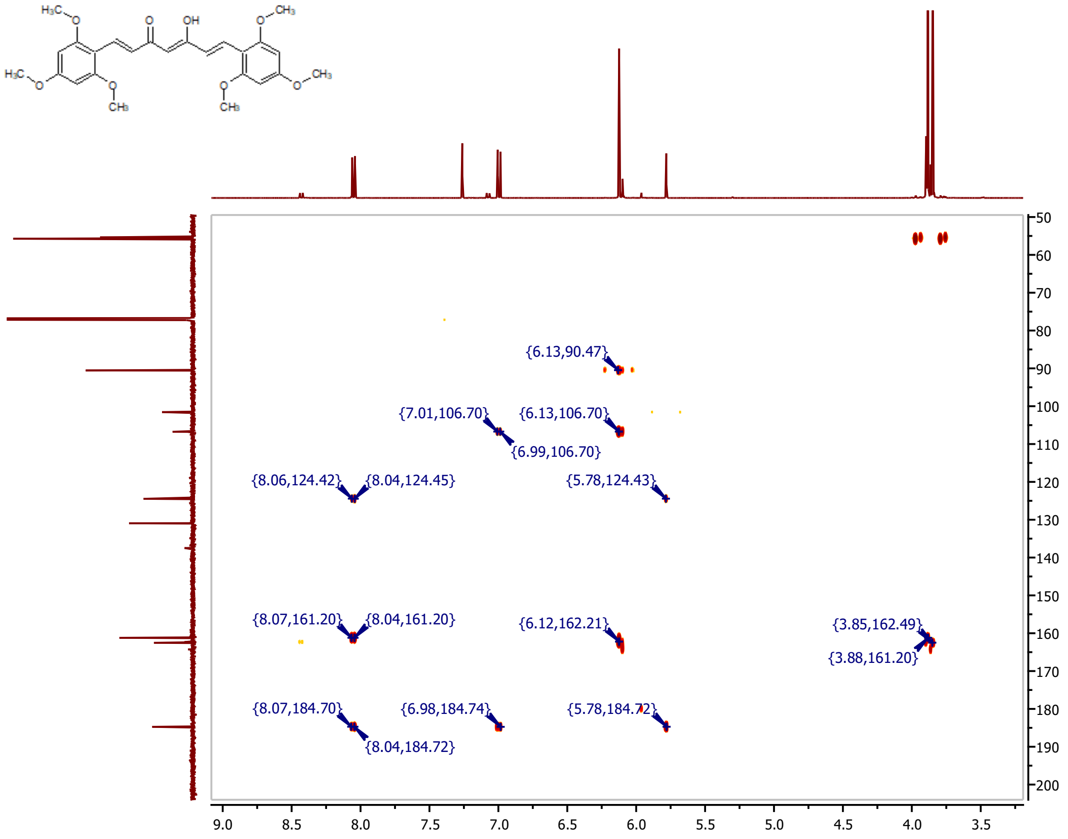

Figure S109. NMR experiments of compound **9a**

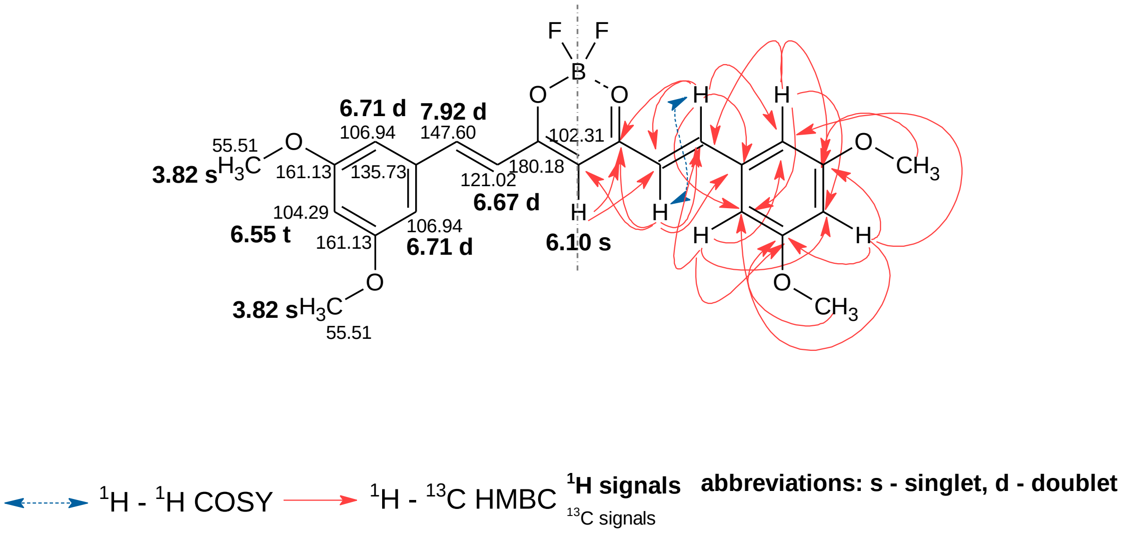

Figure S110. ¹H NMR of curcumin **9a**.

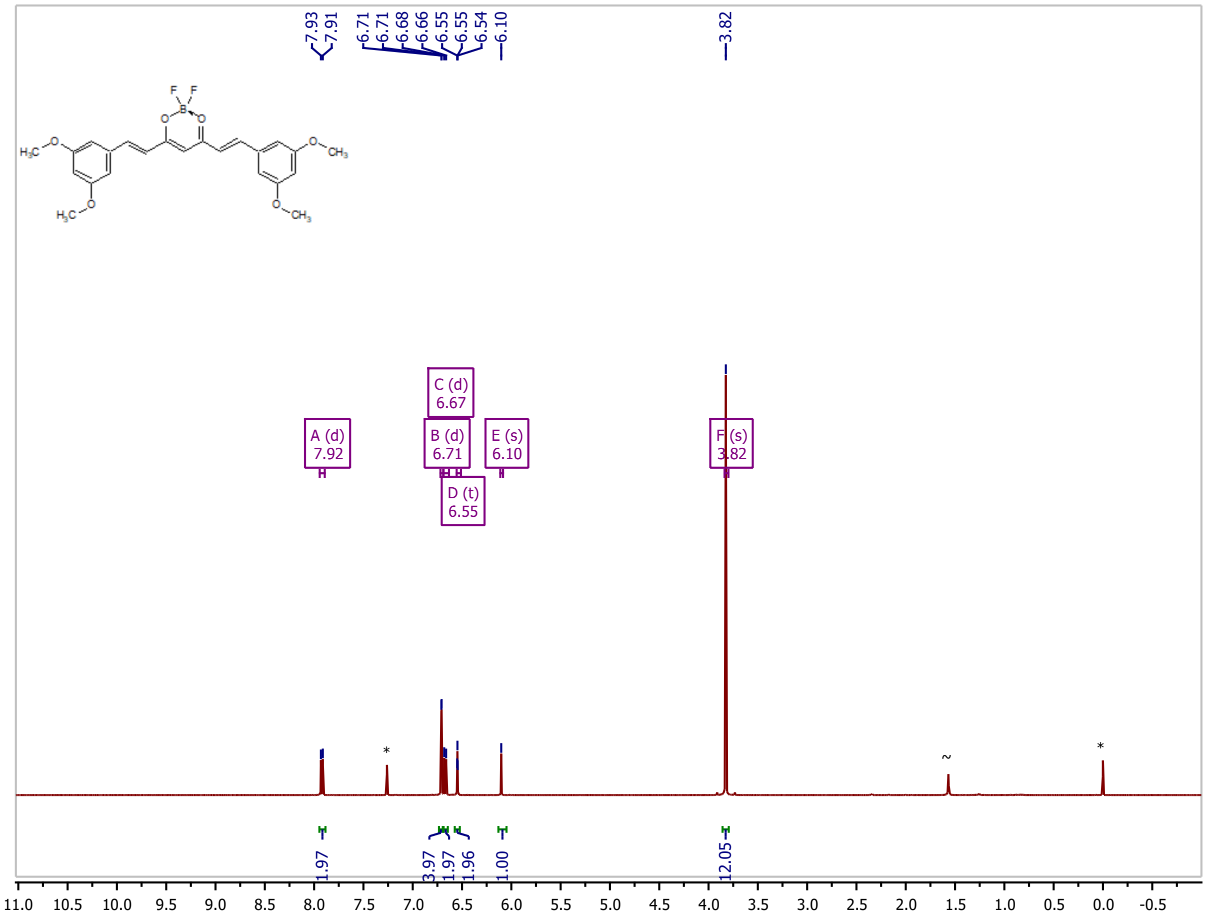

Figure S111. ^{13}C NMR of curcumin **9a**.

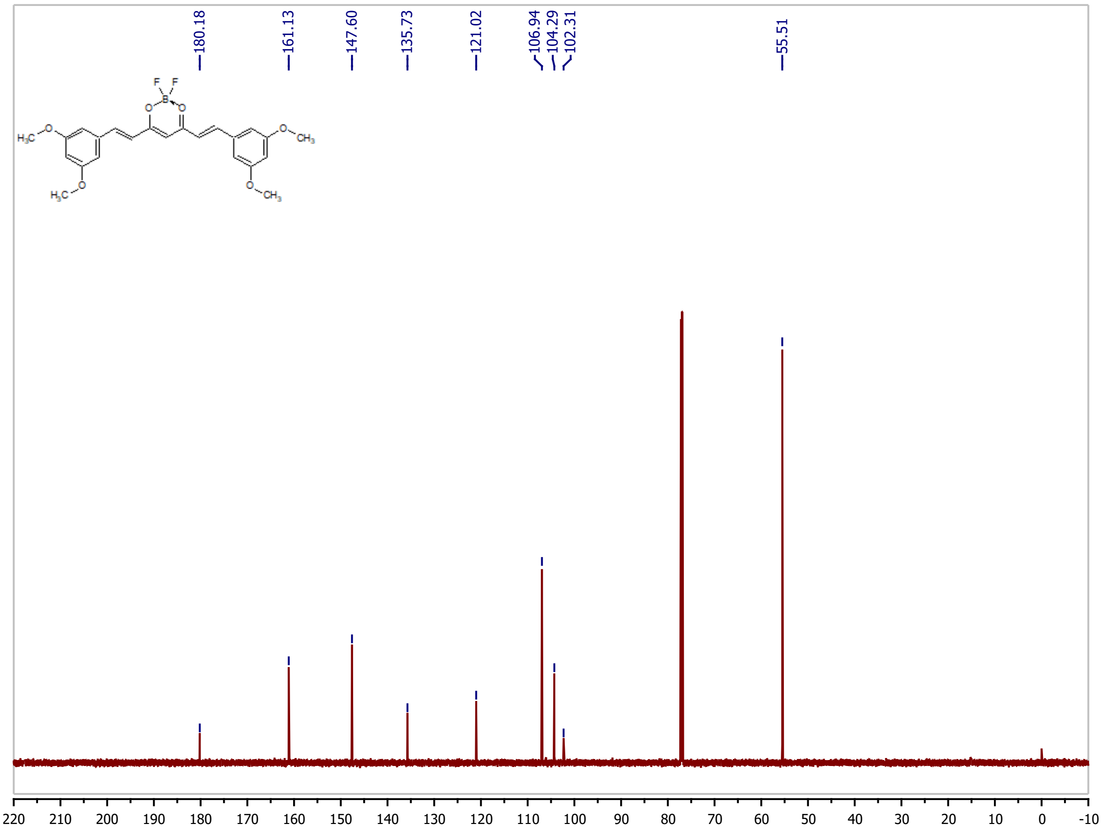

Figure S112. ^{1}H-^{1}H COSY of curcumin **9a**.

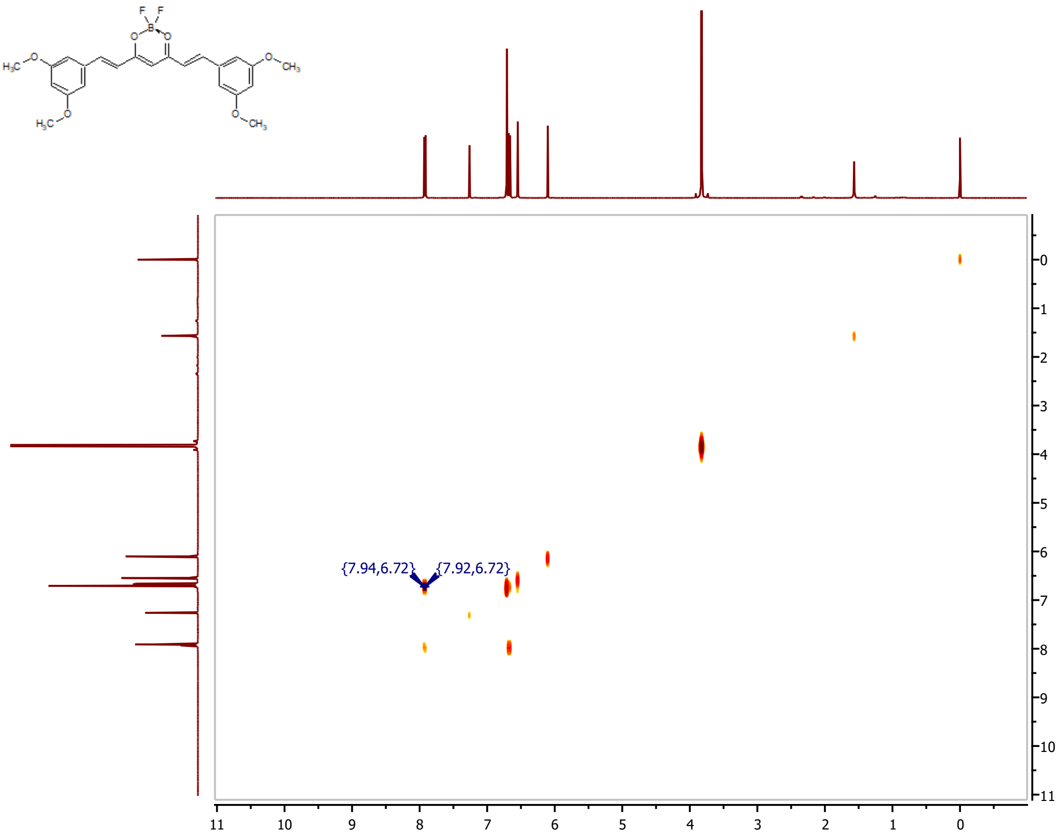

Figure S113. ^{1}H-^{13}C HSQC of curcumin **9a**.

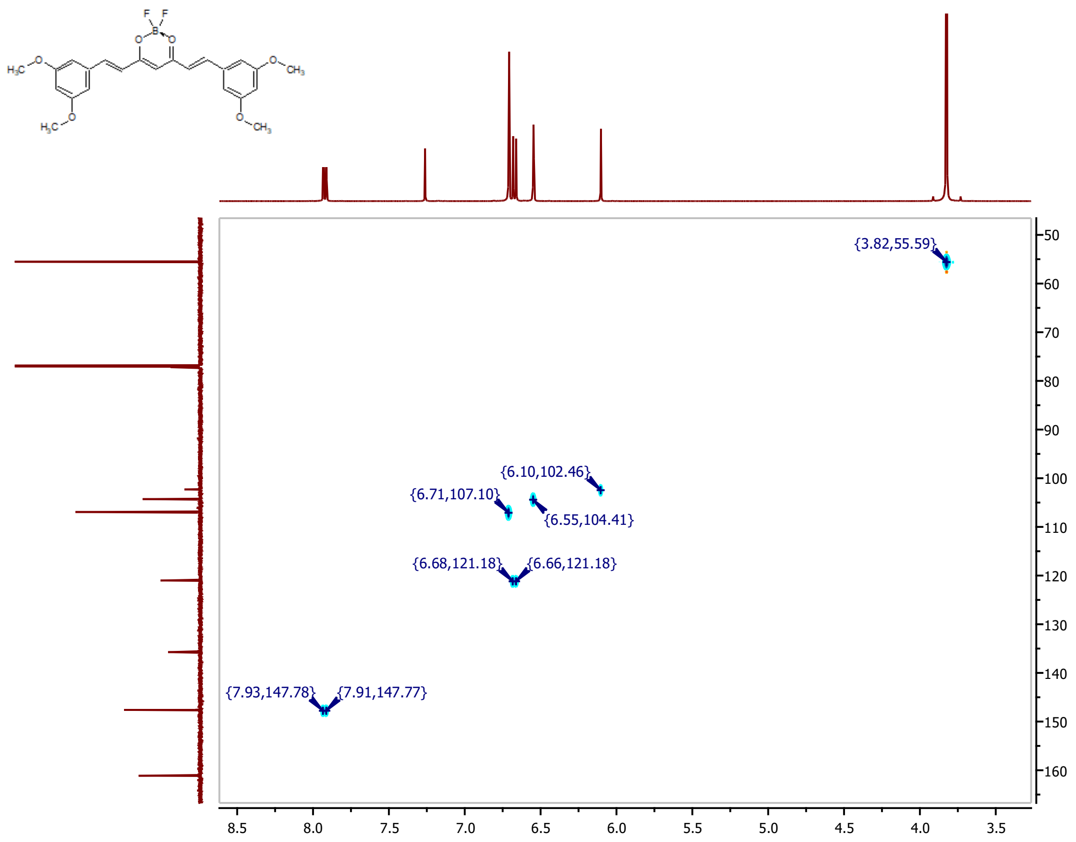

Figure S114. ^{1}H-^{13}C HMBC of curcumin **9a**

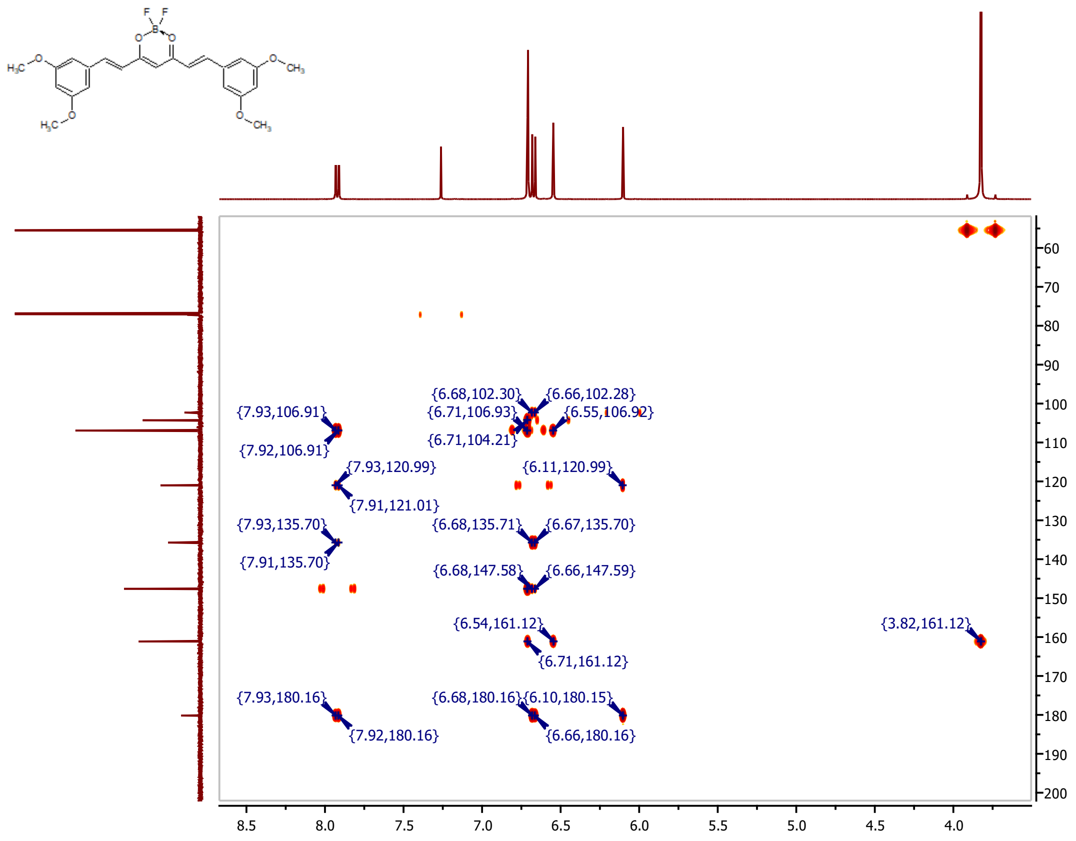

Figure S115. NMR experiments of compound **9b**

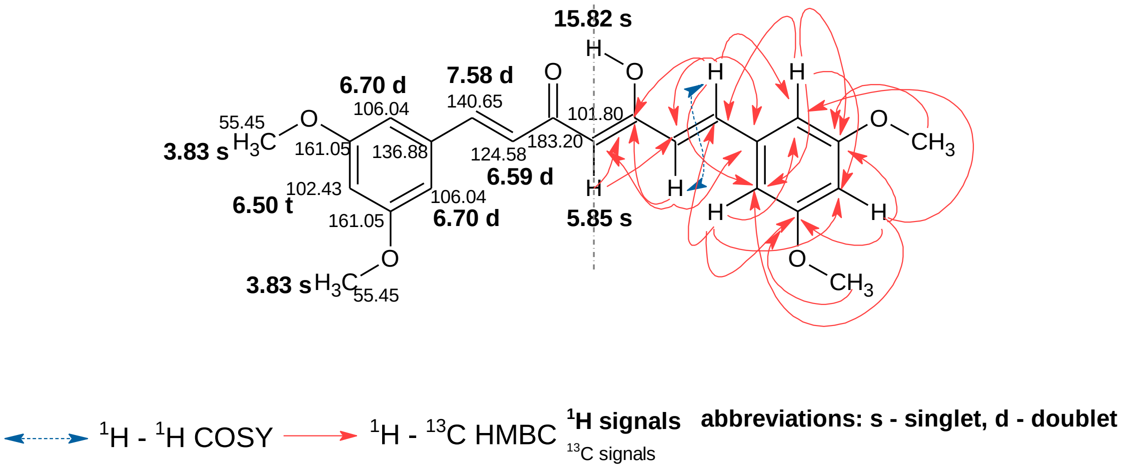

Figure S116. ^{1}H NMR of curcumin **9b**.

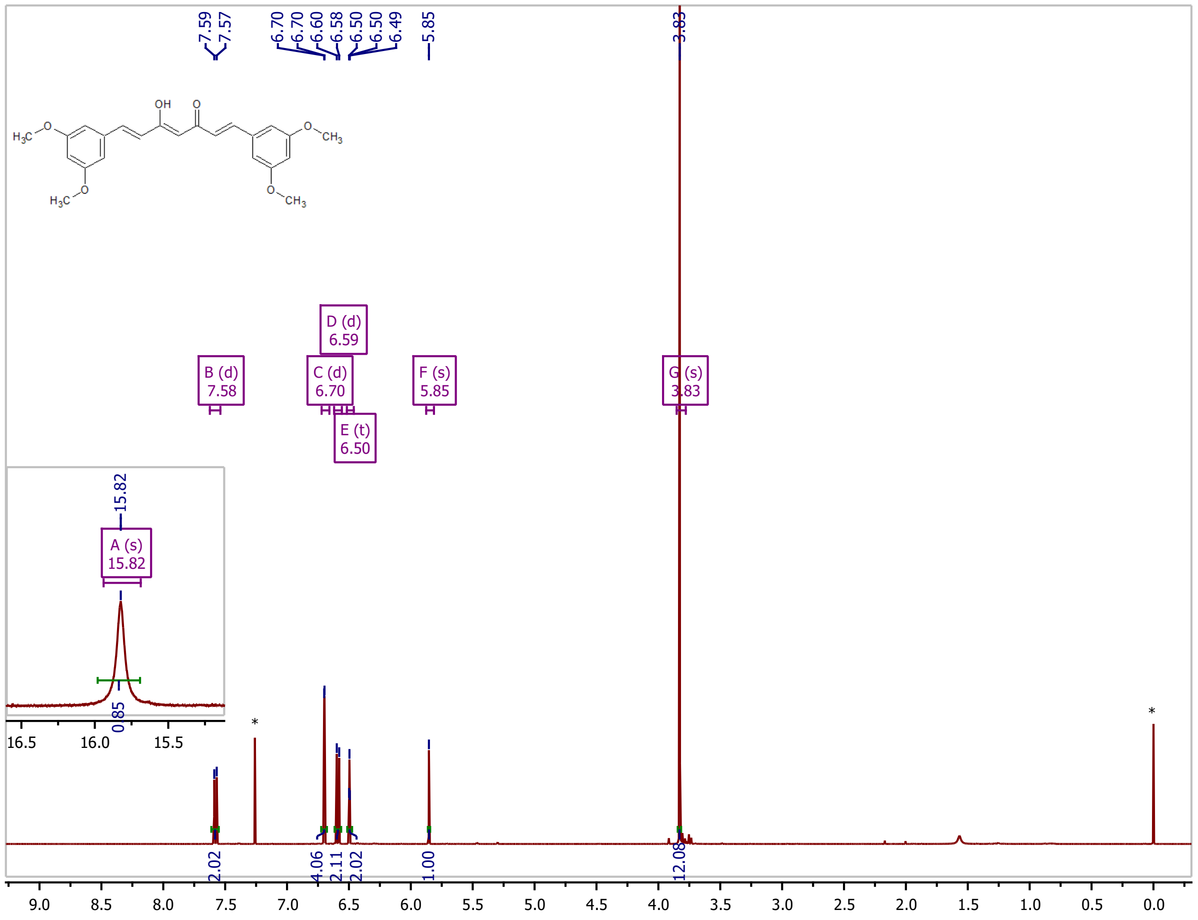

Figure S117. ^{13}C NMR of curcumin **9b**.

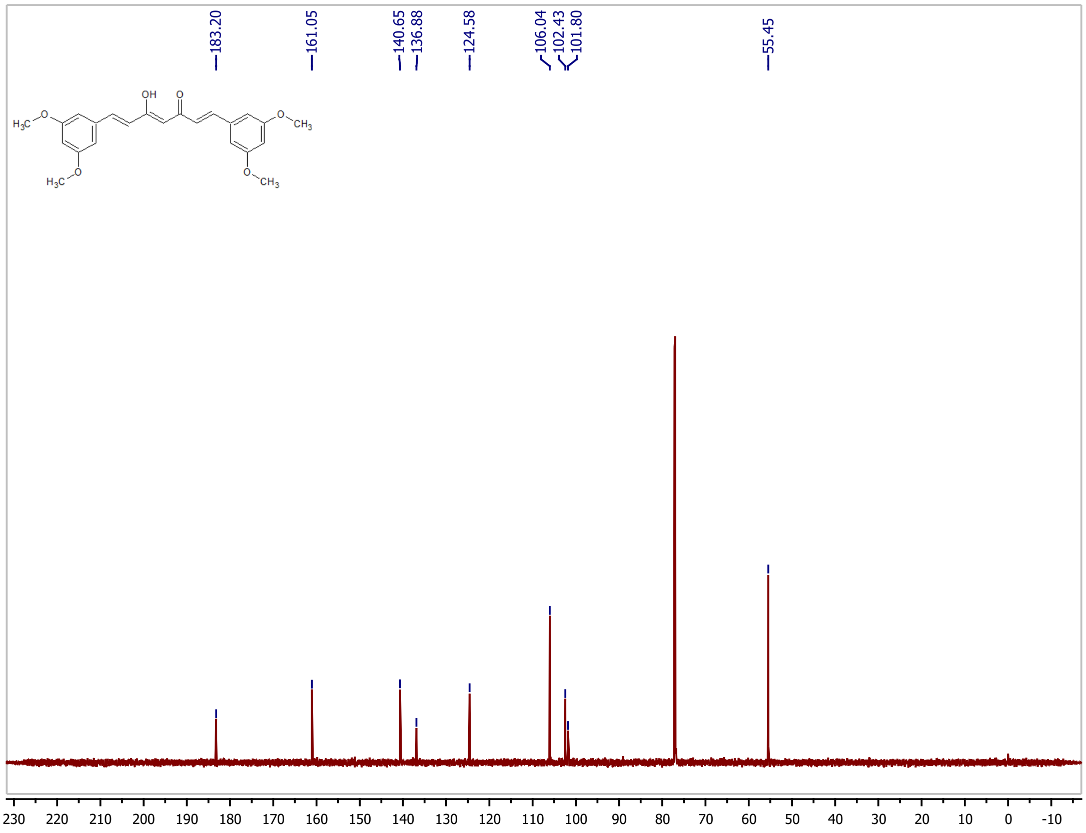

Figure S118. ^{1}H-^{1}H COSY of curcumin **9b**.

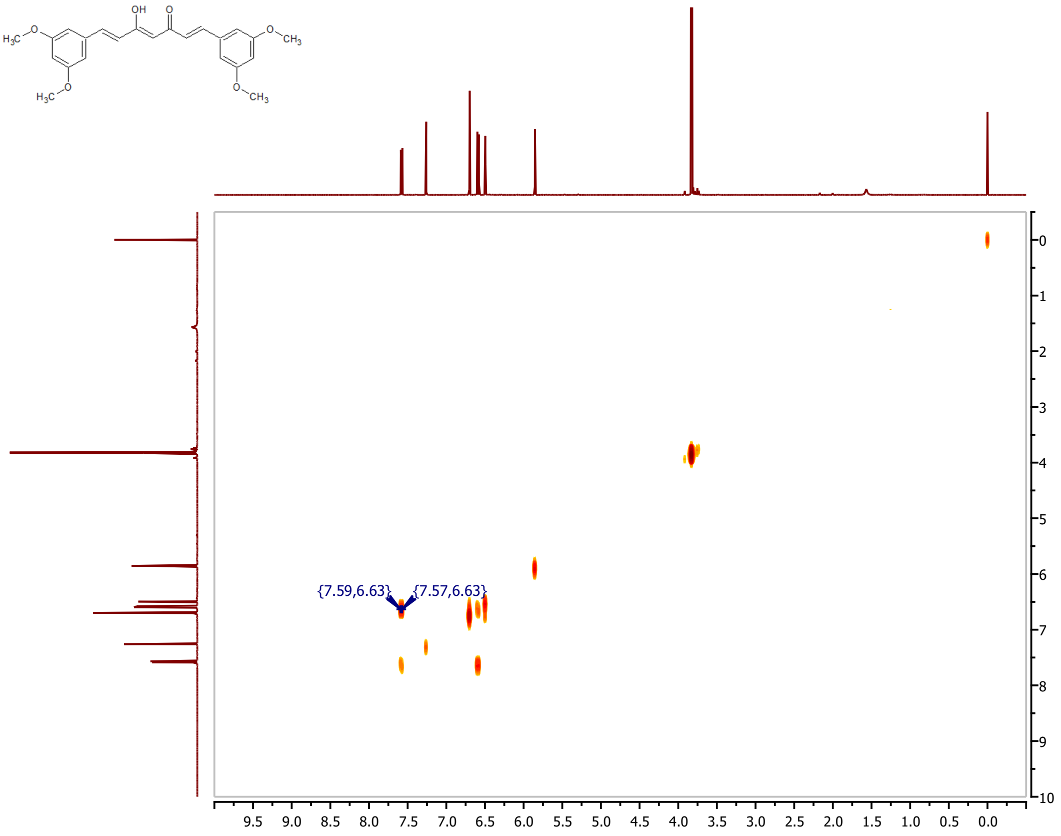

Figure S119. ^{1}H-^{13}C HSQC of curcumin **9b**.

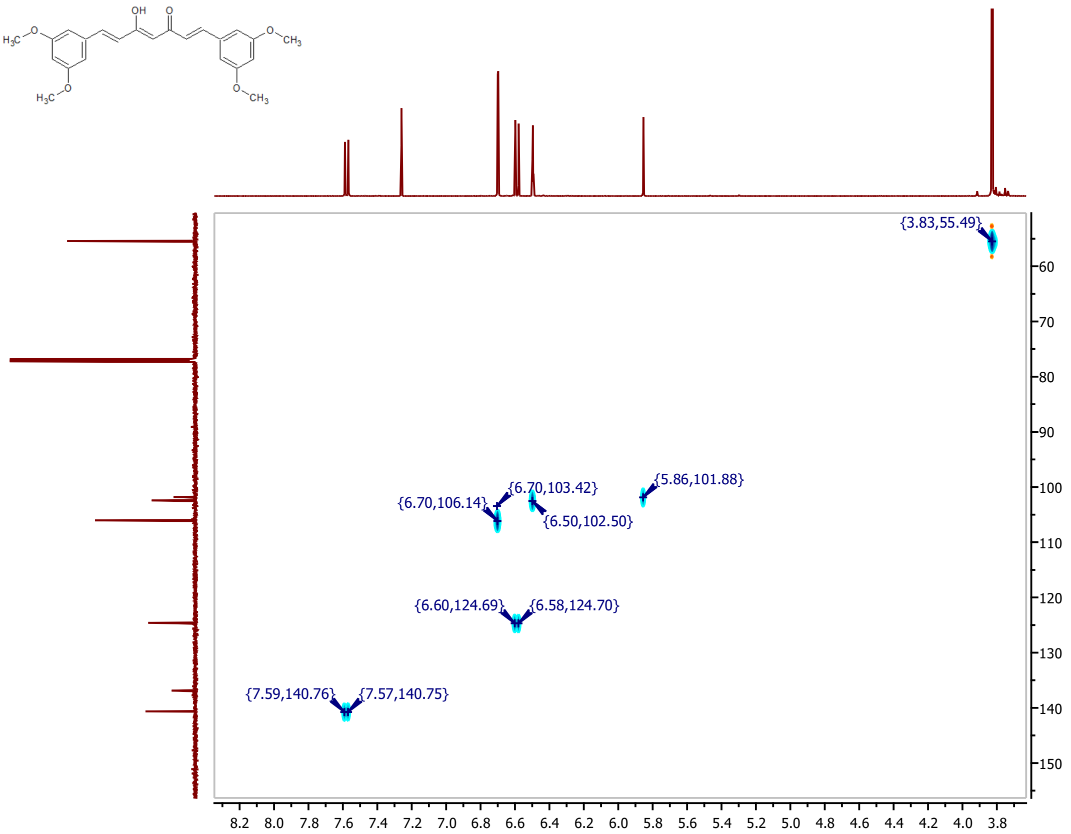

Figure S120. ^{1}H-^{13}C HMBC of curcumin **9b**

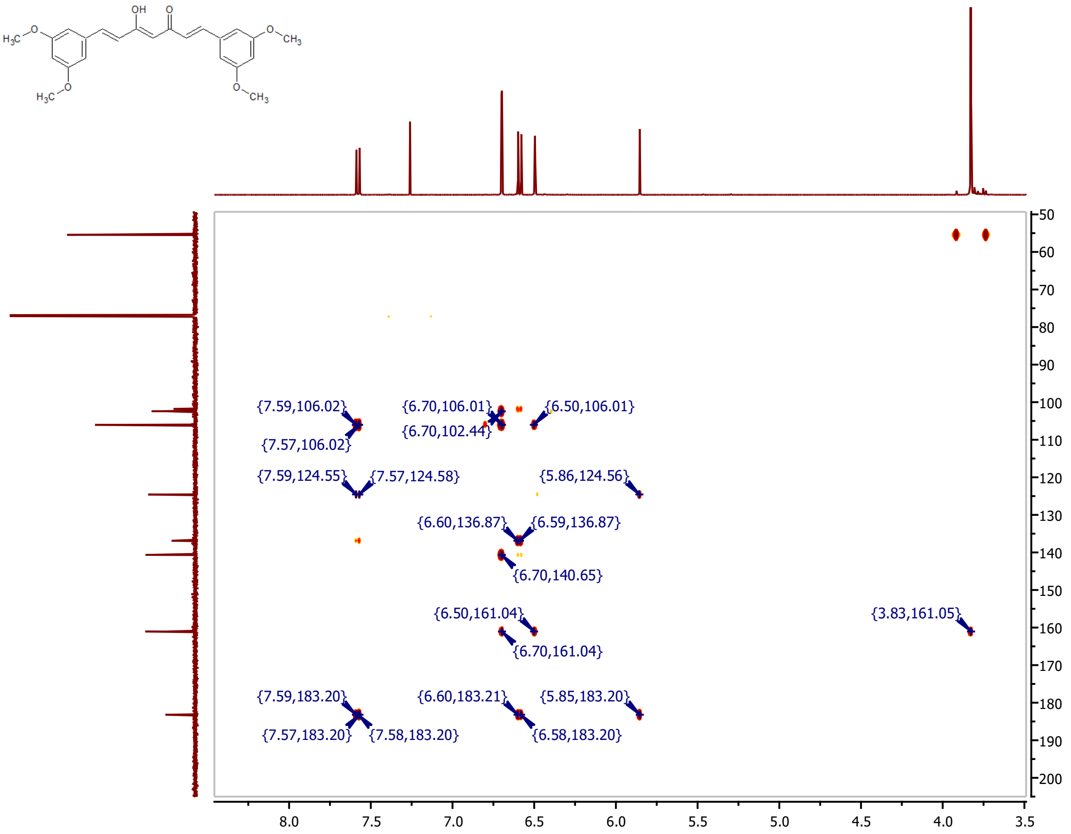

Figure S121. NMR experiments of compound **10a**

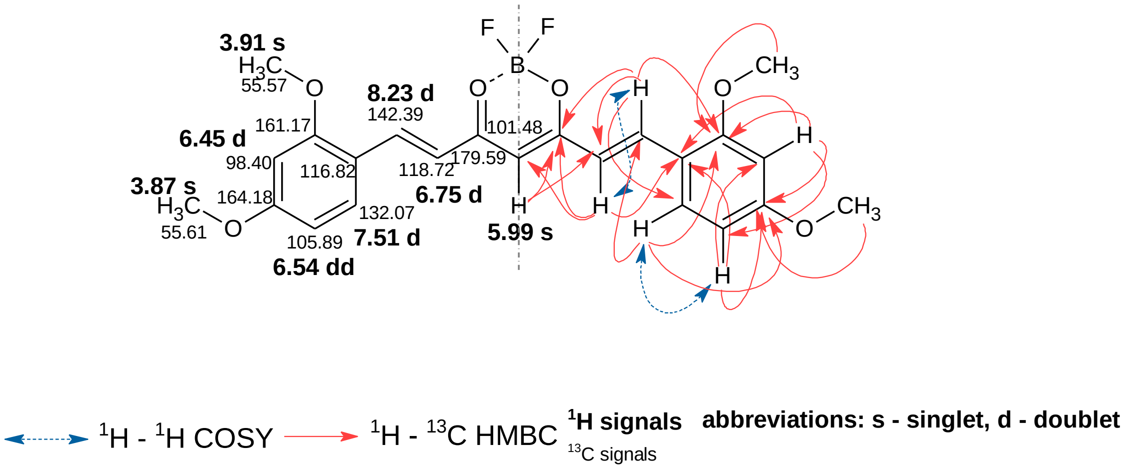

Figure S122. ^{1}H NMR of curcumin **10a**.

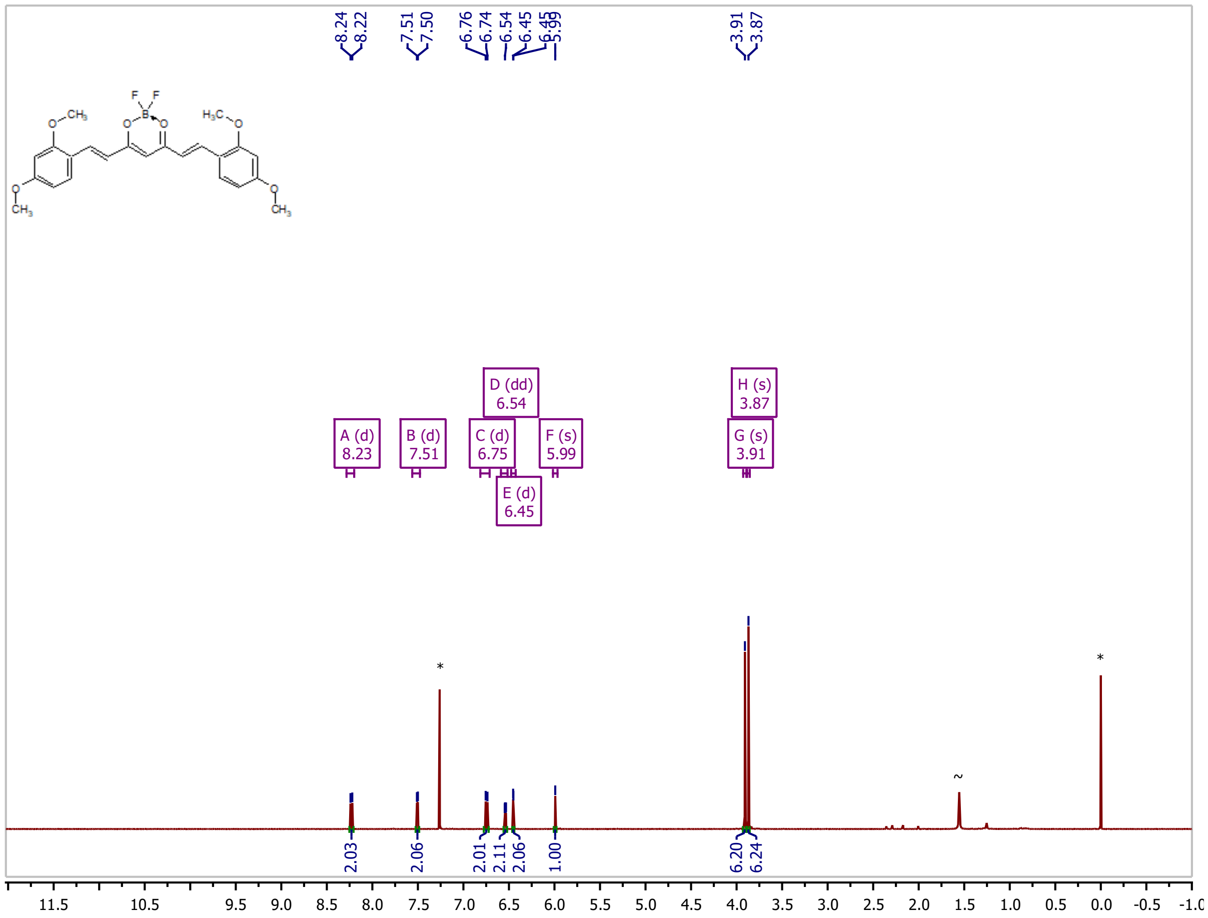

Figure S123. ^{13}C NMR of curcumin **10a**.

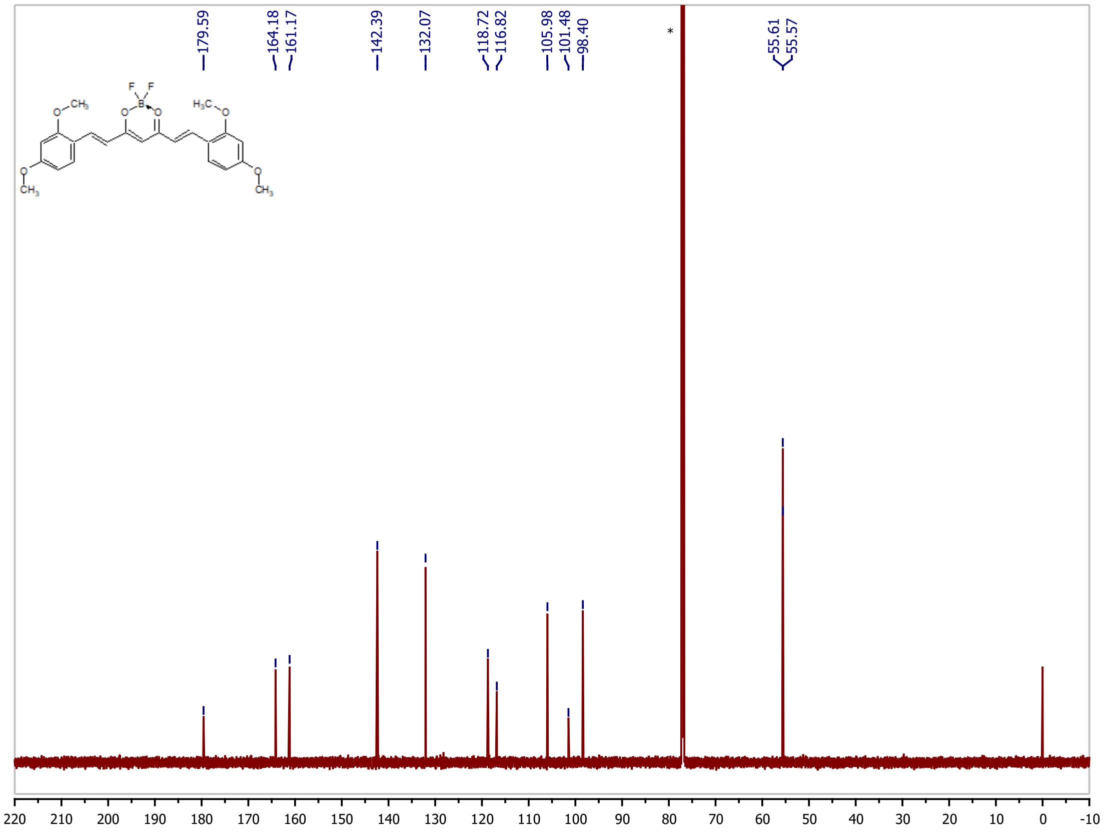

Figure S124. ^{1}H-^{1}H COSY of curcumin **10a**.

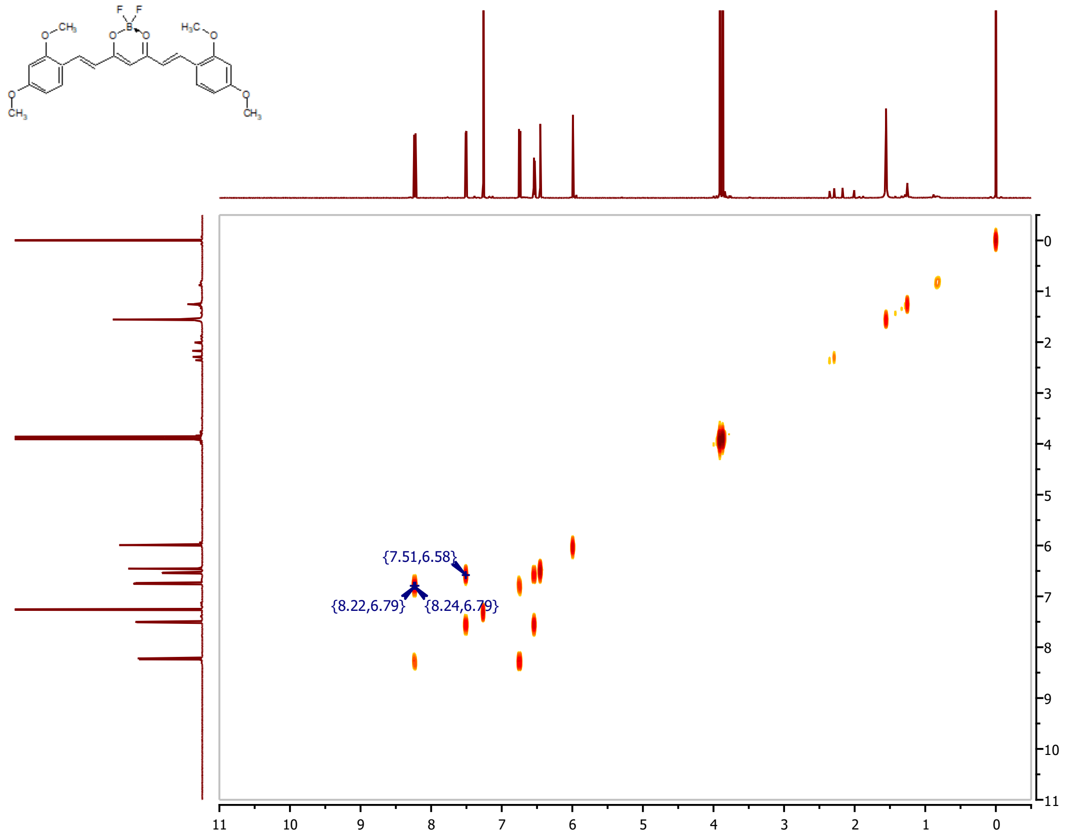

Figure S125. ^{1}H-^{13}C HSQC of curcumin **10a**.

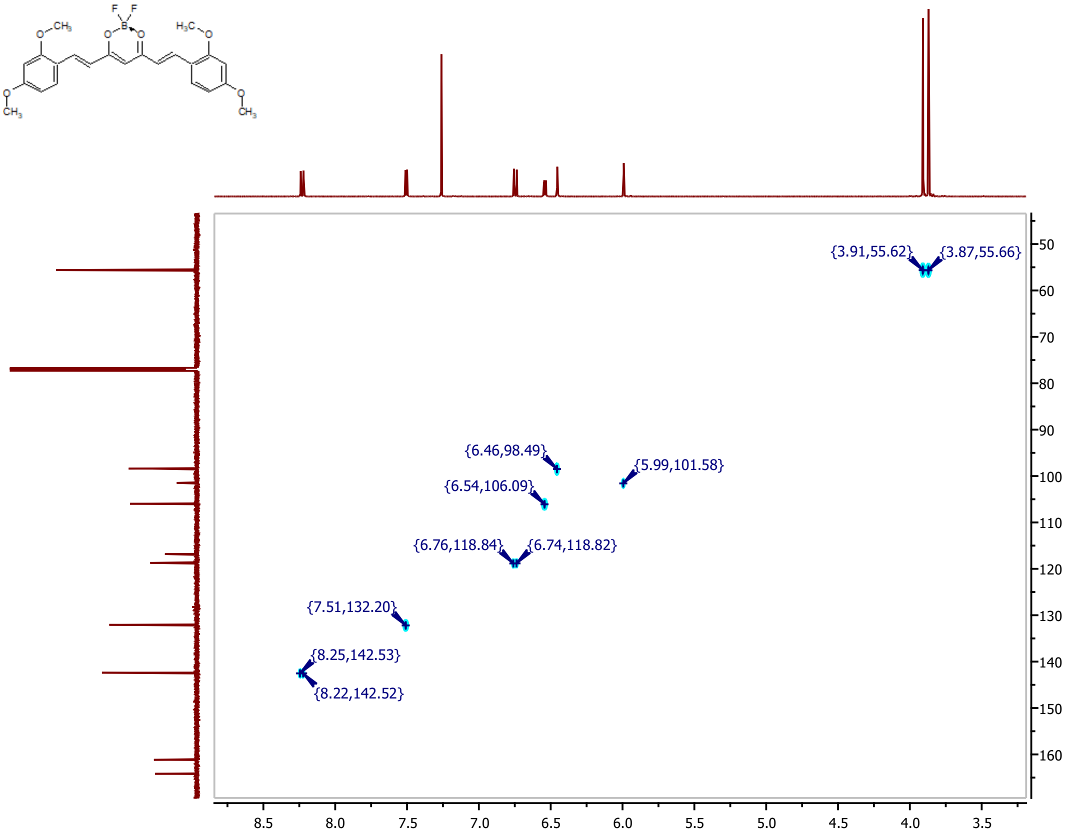

Figure S126. ^{1}H-^{13}C HMBC of curcumin **10a**

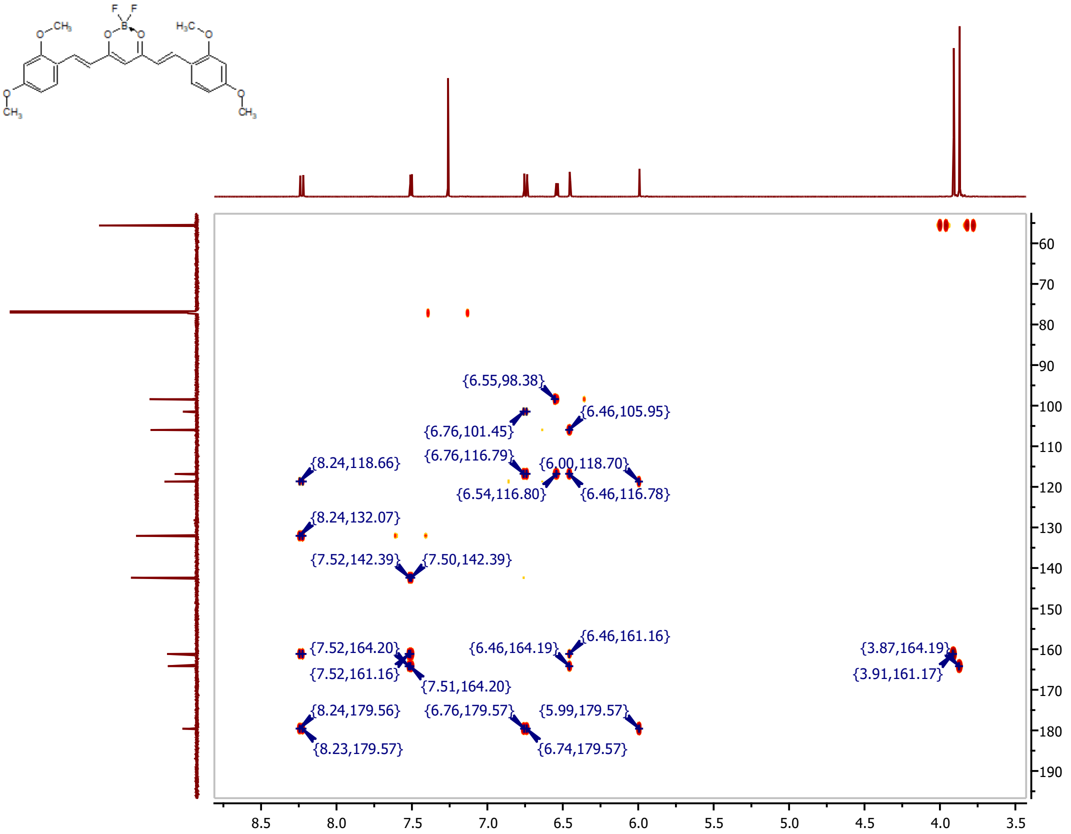

Figure S127. NMR experiments of compound **10b**

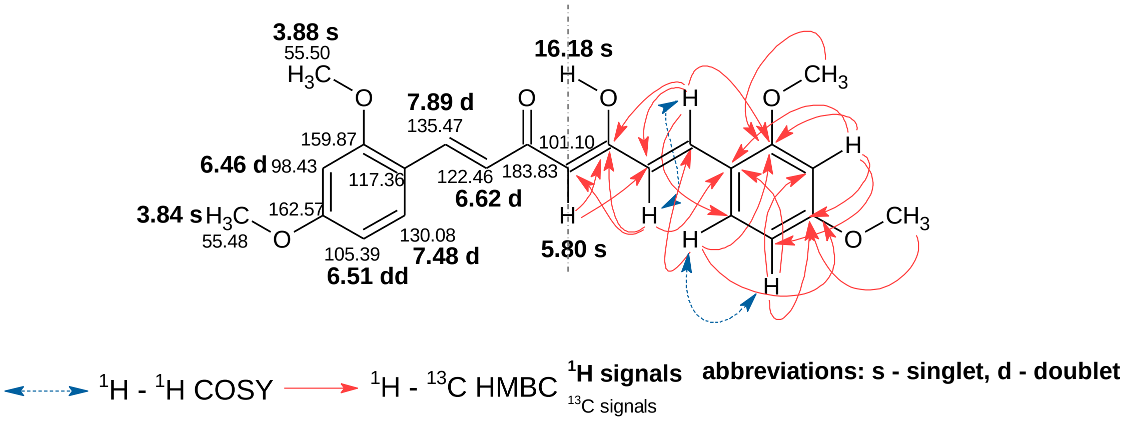

Figure S128. ^{1}H NMR of curcumin **10b**.

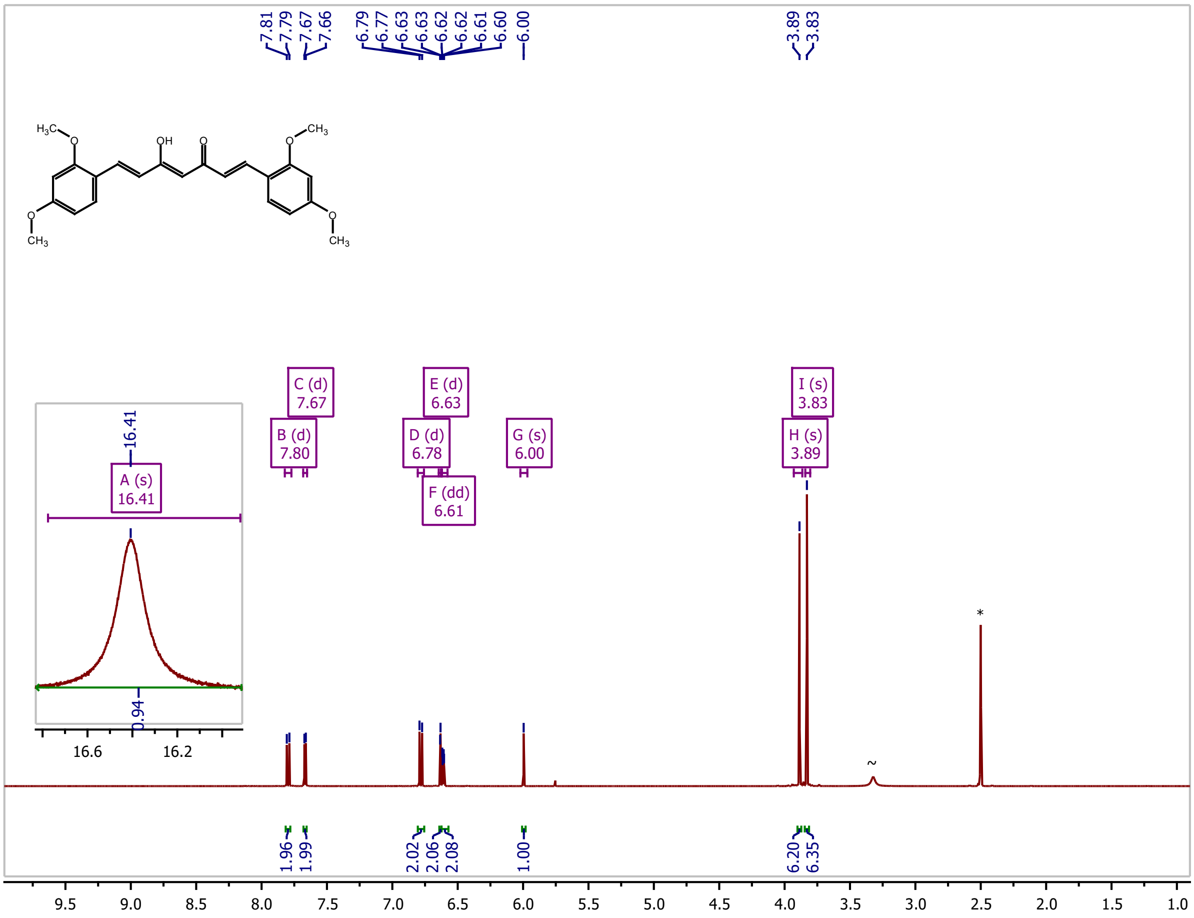

Figure S129. ${}^{13}C$ NMR of curcumin **10b**.

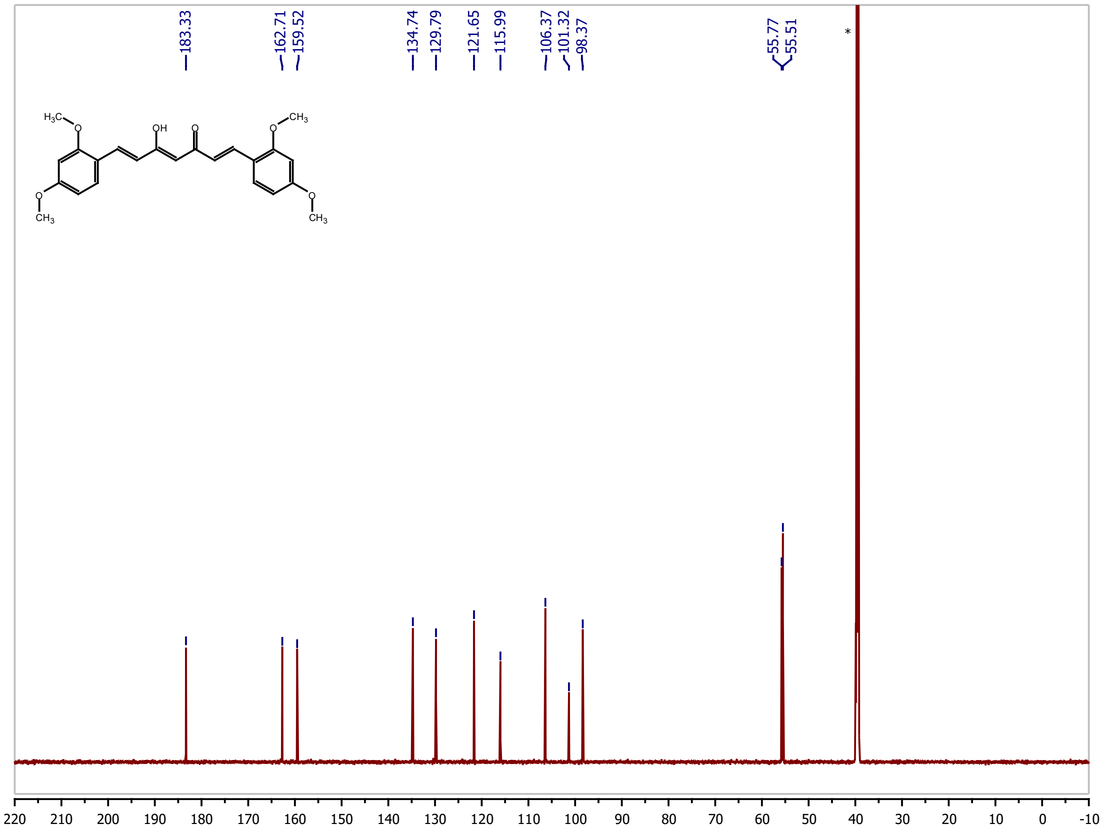

Figure S130. ${}^{1}H$-${}^{1}H$ COSY of curcumin **10b**.

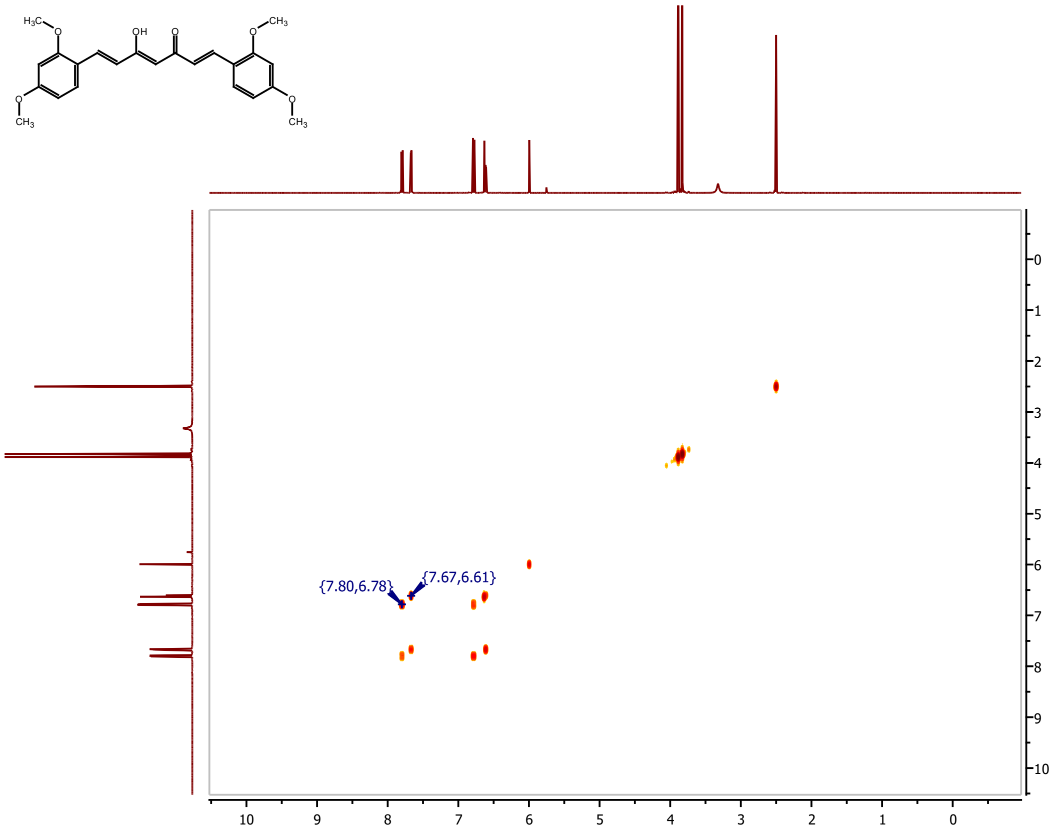

Figure S131. ^{1}H-^{13}C HSQC of curcumin **10b**.

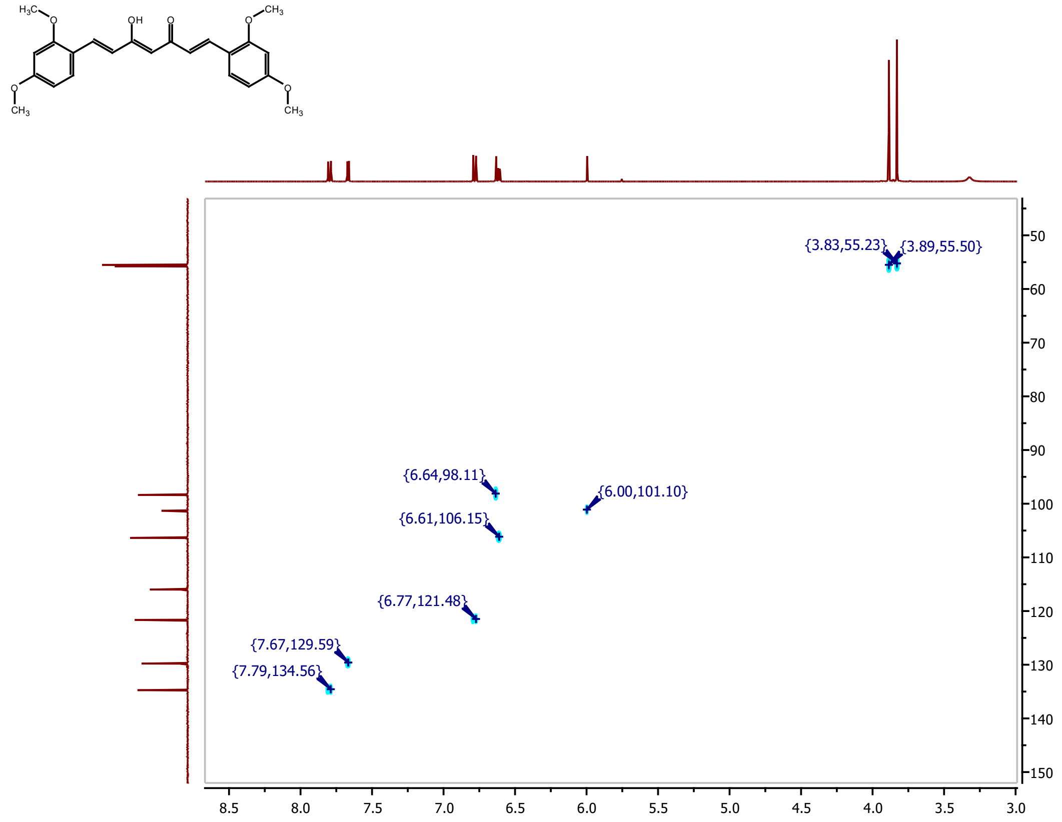

Figure S132. ^{1}H-^{13}C HMBC of curcumin **10b**

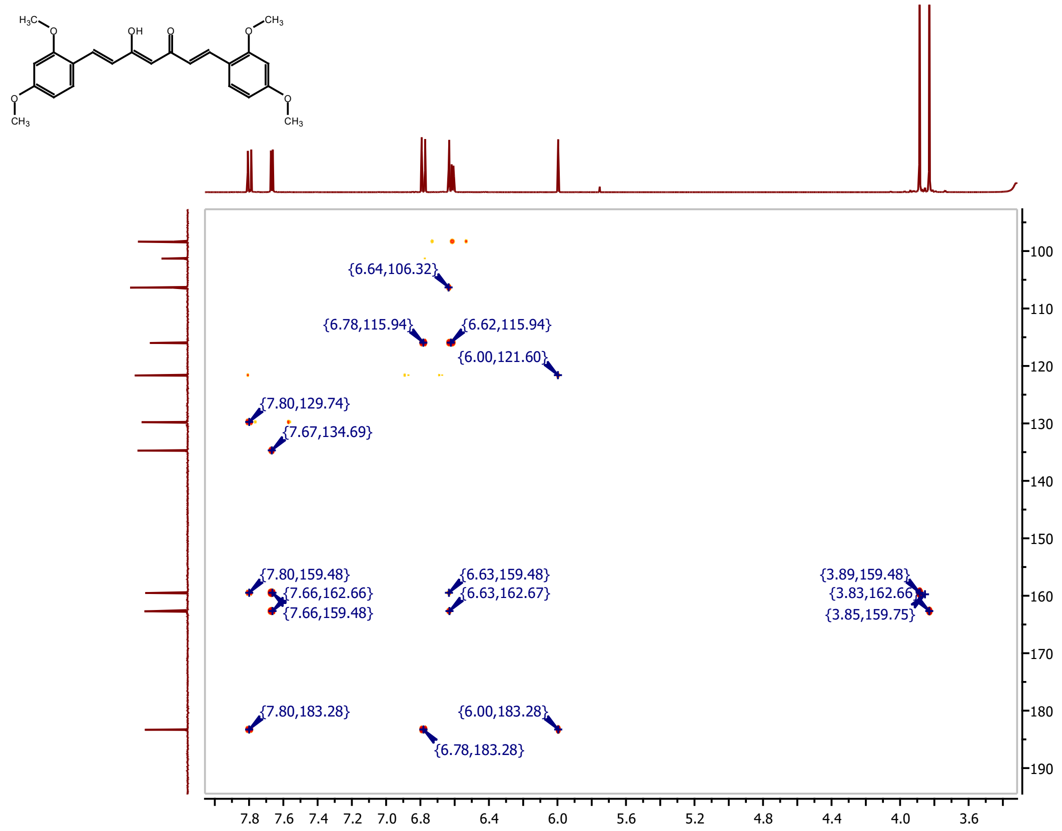

2. MS ESI spectrometry data

Figure S133. Mass spectra of compound **1a,b**.

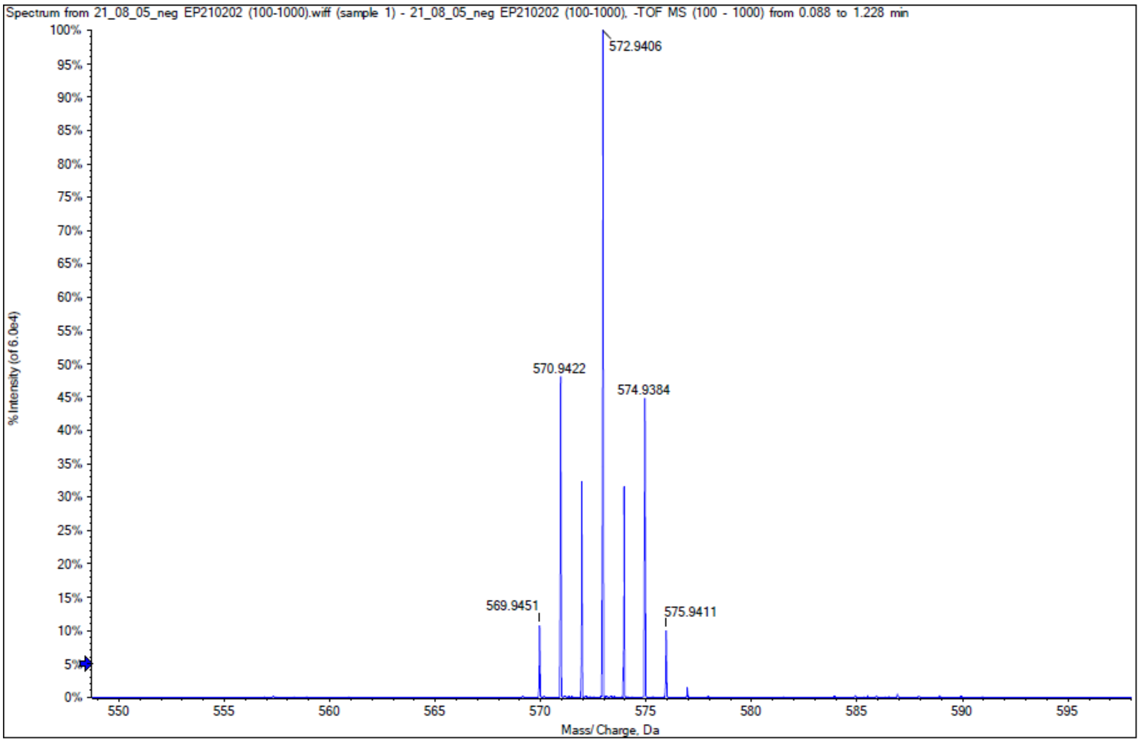

(a)

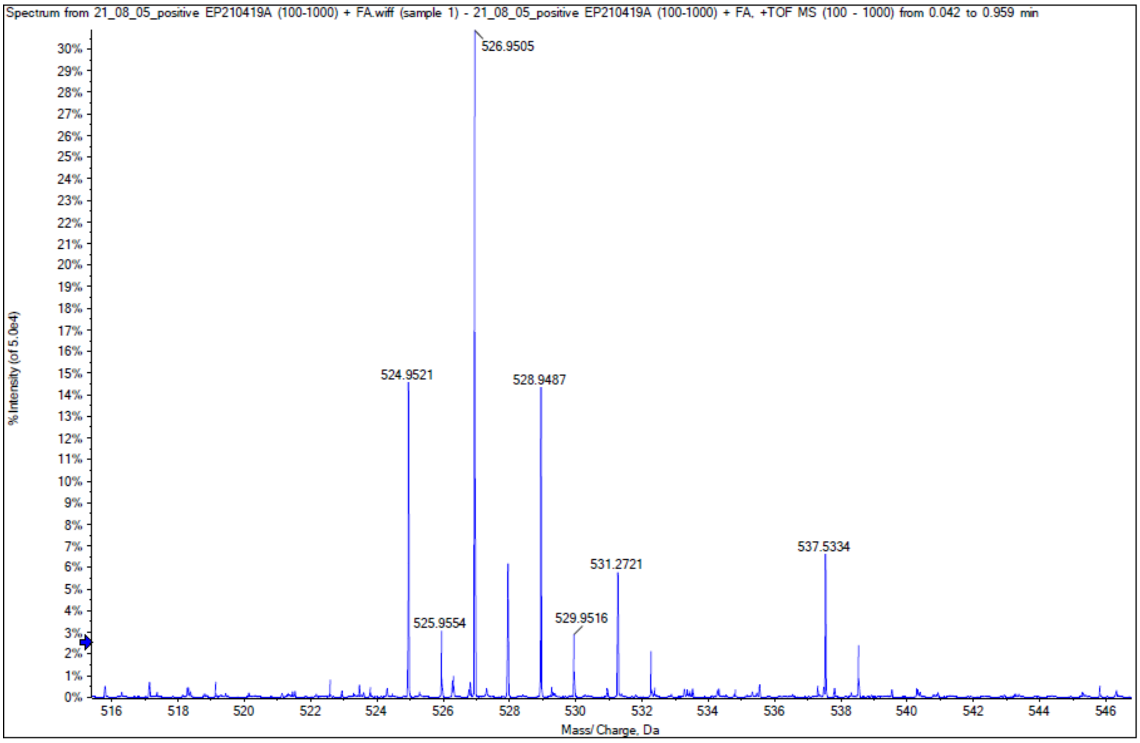

(b)

Figure S134. Mass spectra of compound **2a**

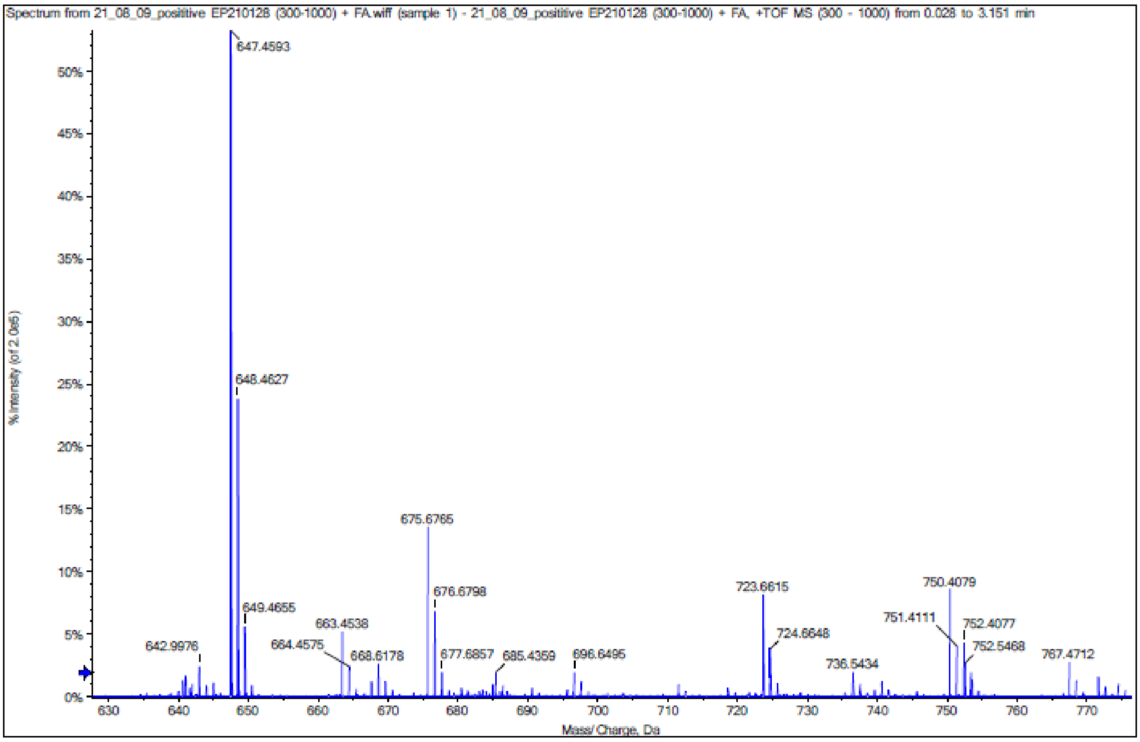

Figure S135. Mass spectra of compound **2b.**

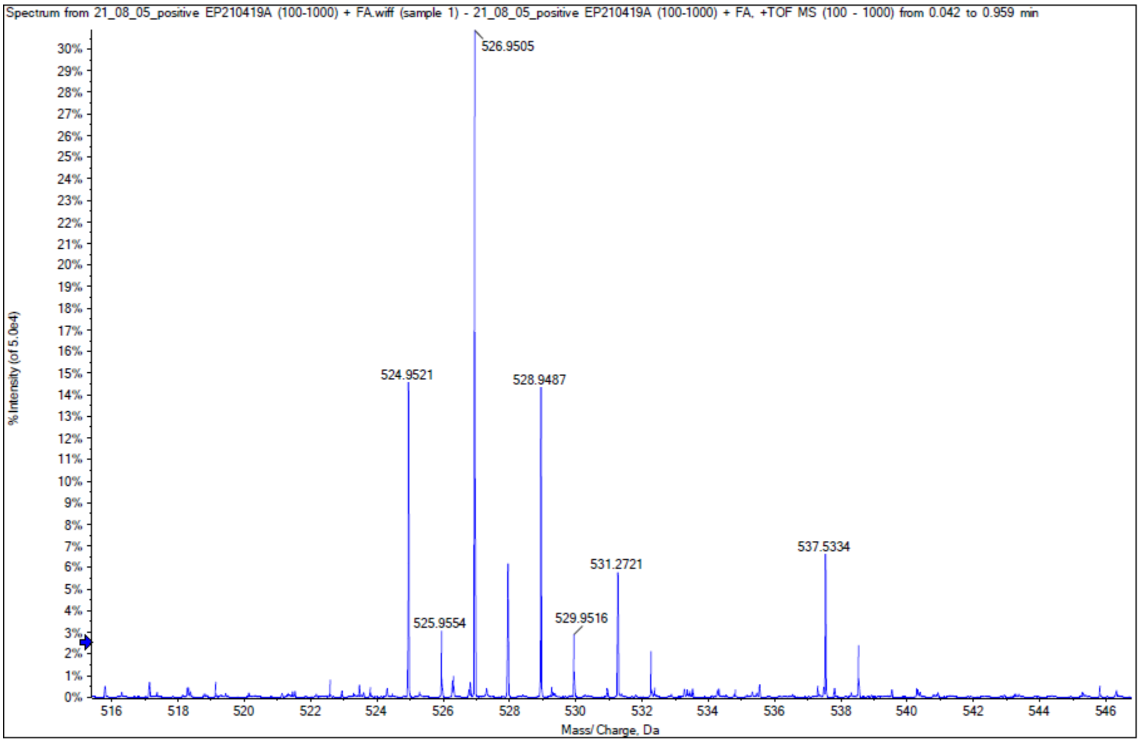

Figure S136. Mass spectra of compound **3a.**

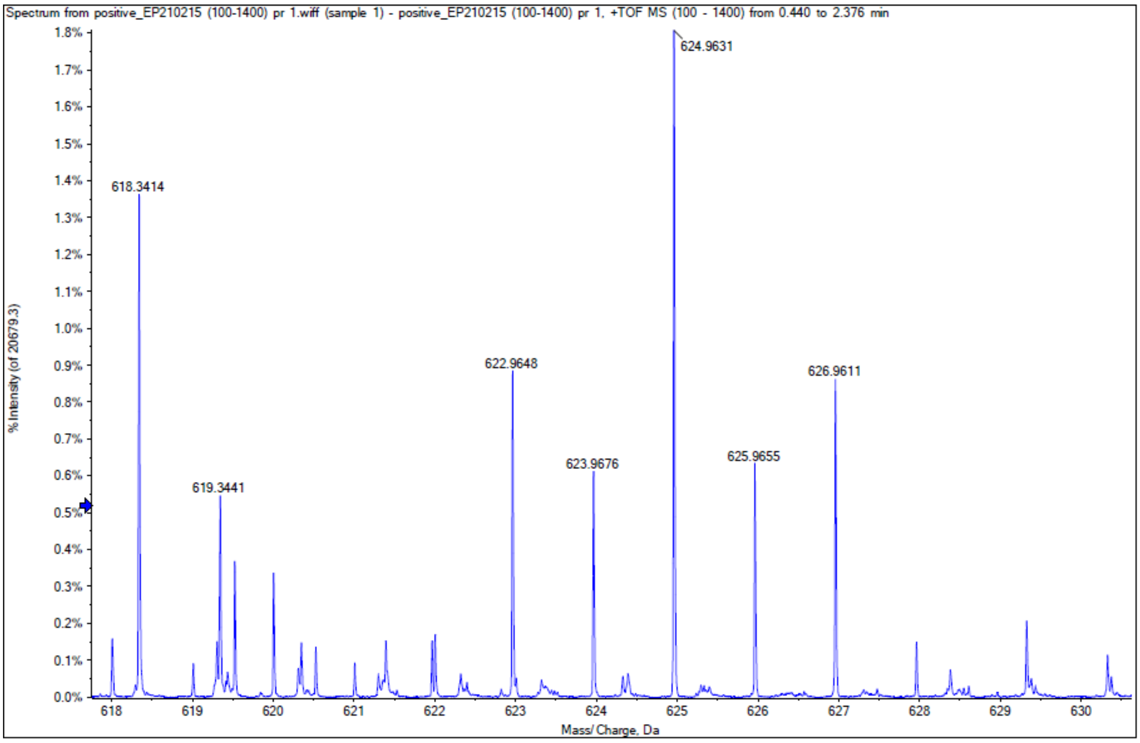

Figure S137. Mass spectra of compound **3b.**

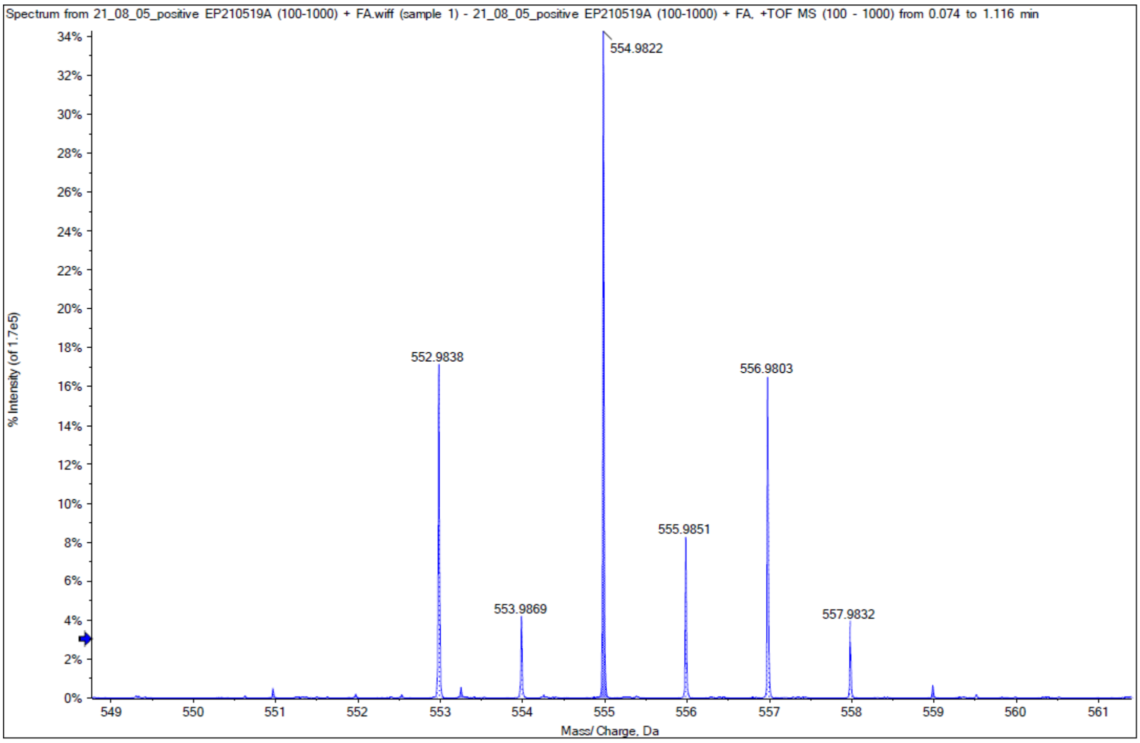

Figure S138. Mass spectra of compound **4a.**

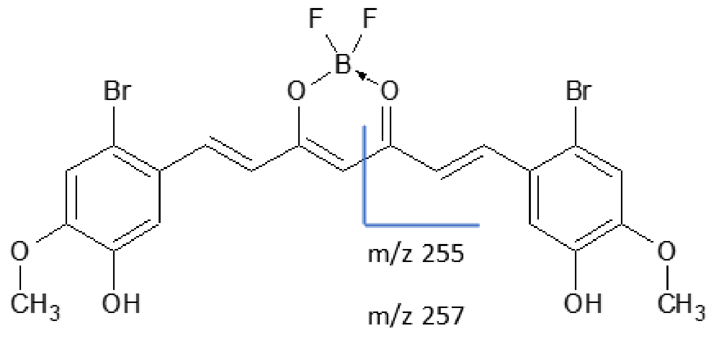
F
F
B
O
O
Br
Br
O
CH3
OH
OH
O
CH3
m/z 255
m/z 257

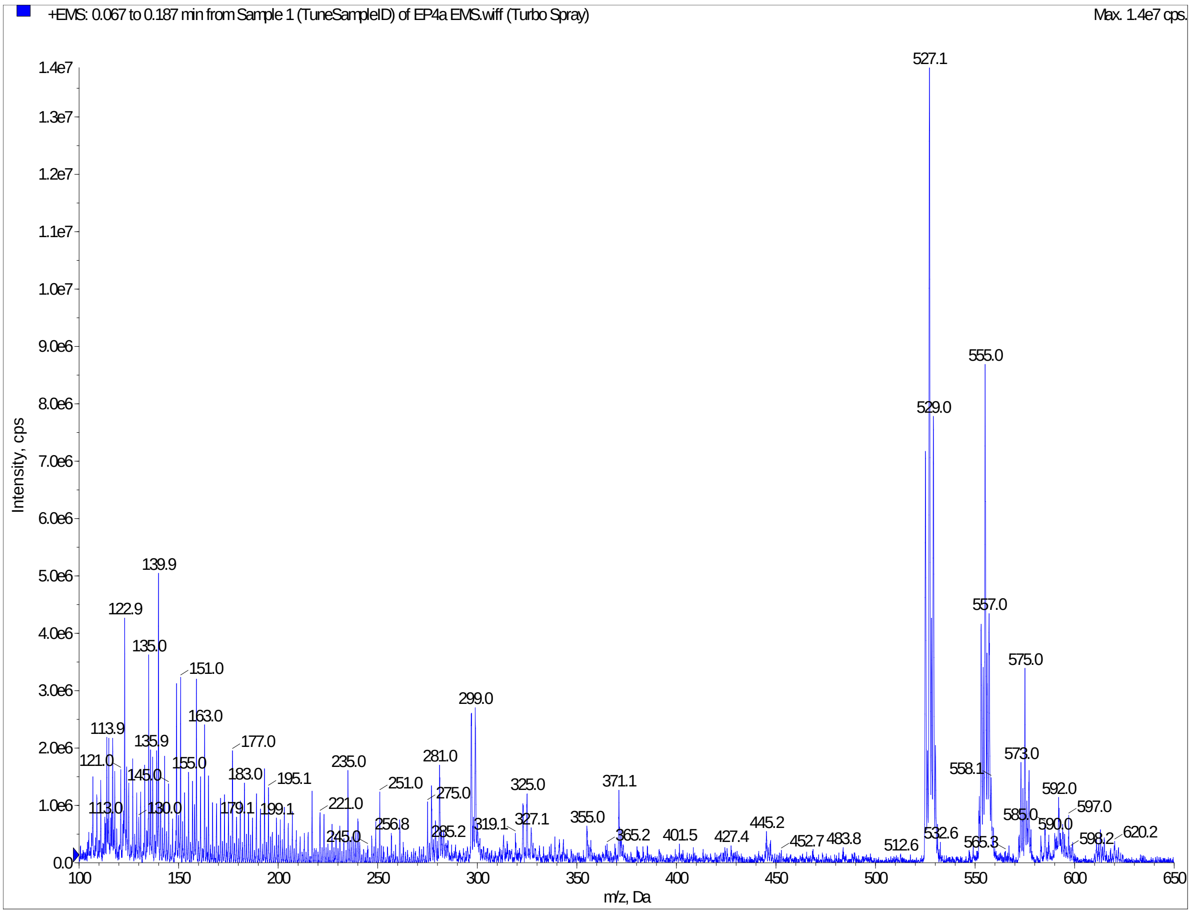
+EMS: 0.067 to 0.187 min from Sample 1 (TuneSampleID) of EP4a EMS.wiff (Turbo Spray)
Max. 1.4e7 cps.
Intensity, cps
m/z, Da
527.1
555.0
529.0
557.0
575.0
573.0
558.1
592.0
597.0
585.0
590.0
598.2
620.2
532.6
565.3
512.6
139.9
122.9
135.0
151.0
163.0
113.9
135.9
177.0
121.0
155.0
145.0
183.0
195.1
235.0
281.0
299.0
251.0
275.0
325.0
371.1
113.0
130.0
179.1
199.1
221.0
245.0
256.8
285.2
319.1
327.1
355.0
365.2
401.5
427.4
445.2
452.7
483.8

Figure S139. The compound **4a** [M+H]+ ion fragmentation.

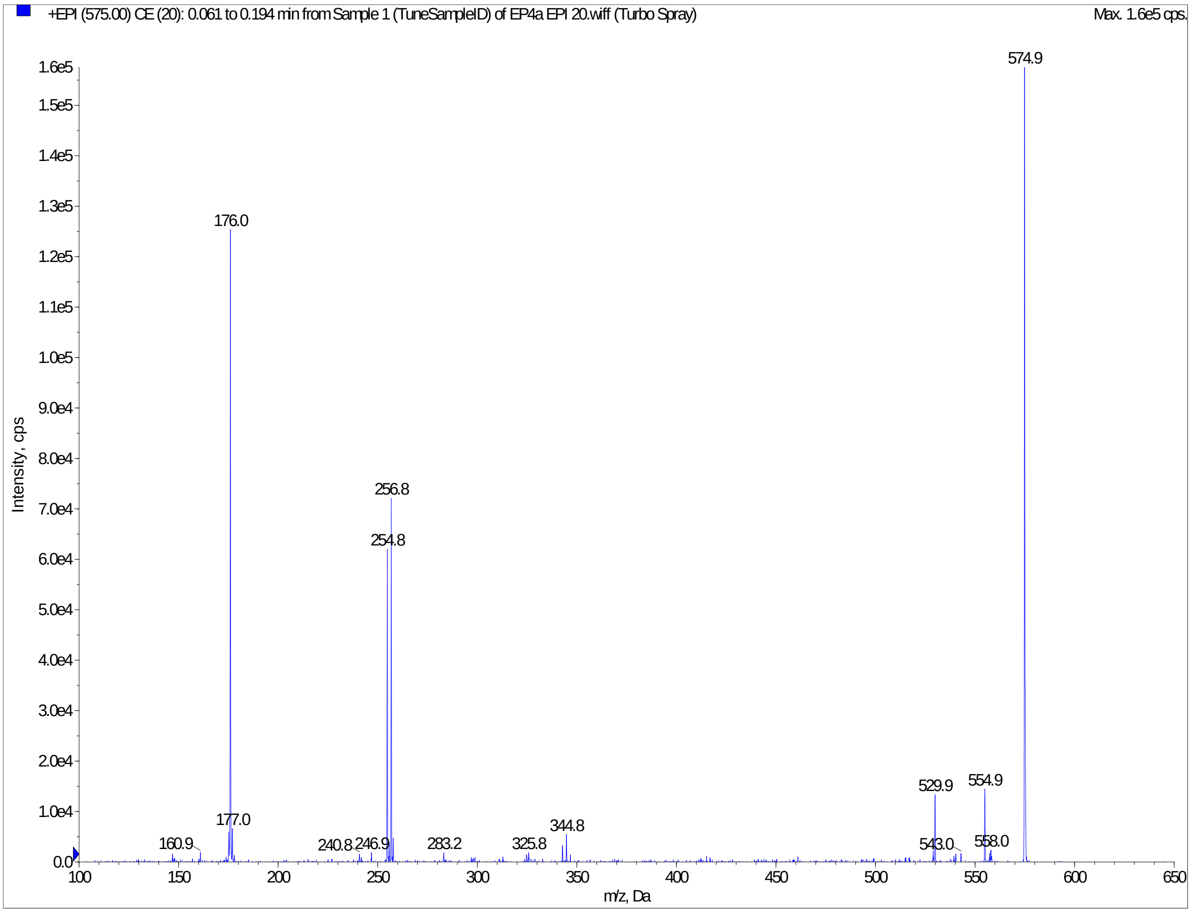

Figure S140. Mass spectra of compound **4b.**

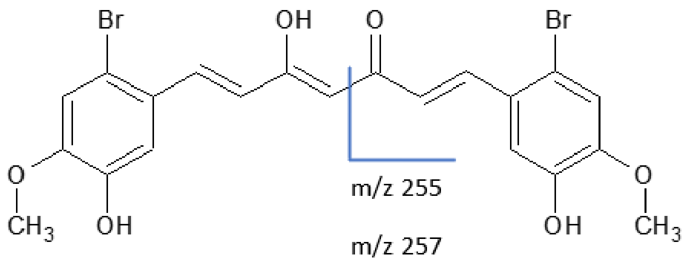

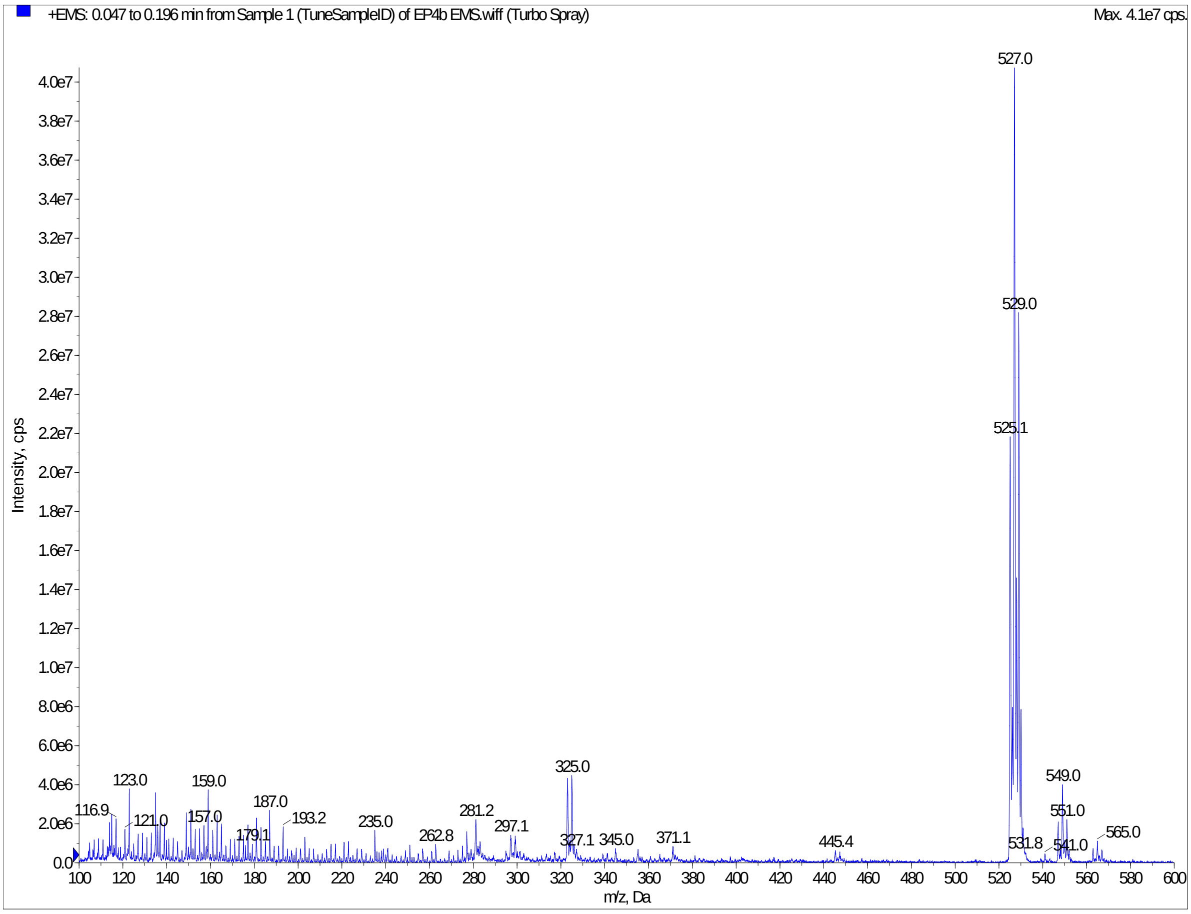

Figure S141. The compound **4b** $[M+H]^+$ ion fragmentation.

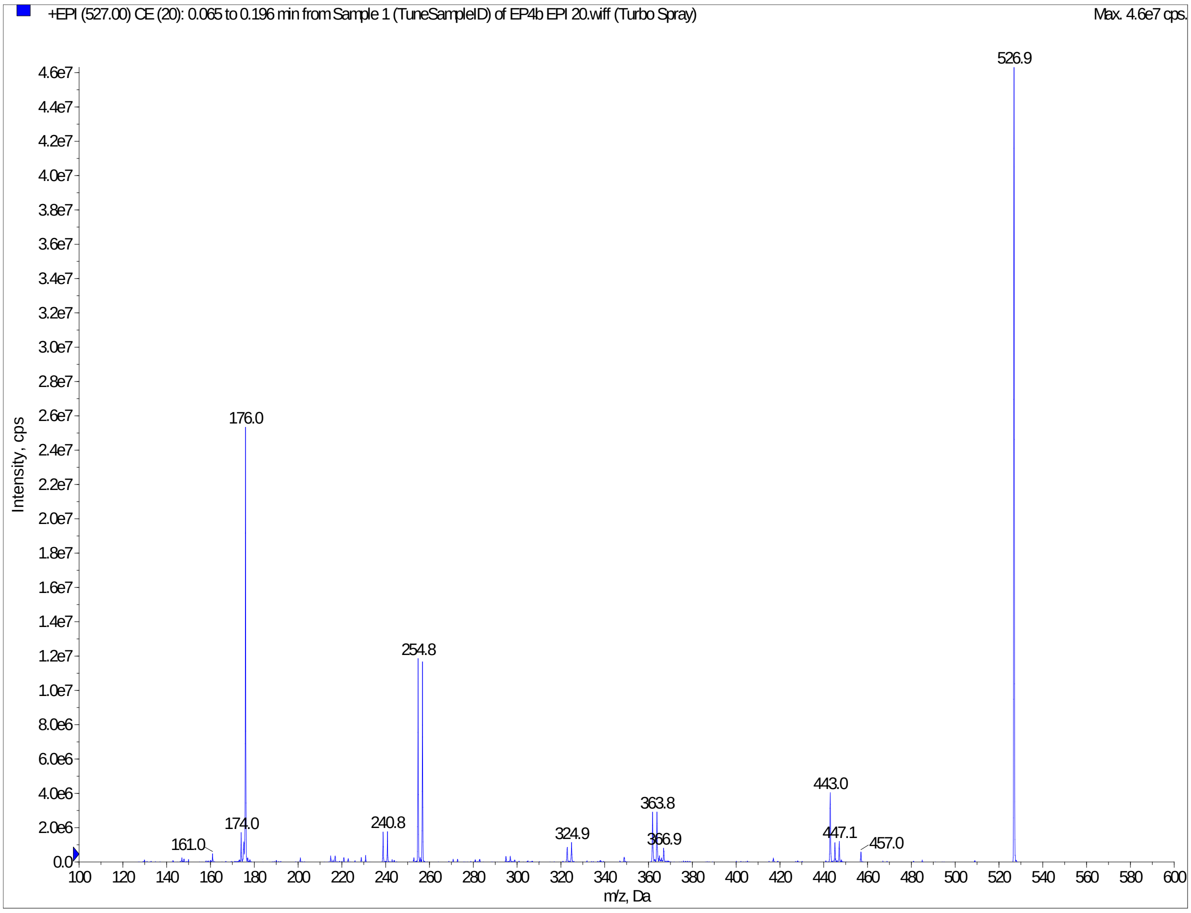

Figure S142. Mass spectra of compound **5a.**

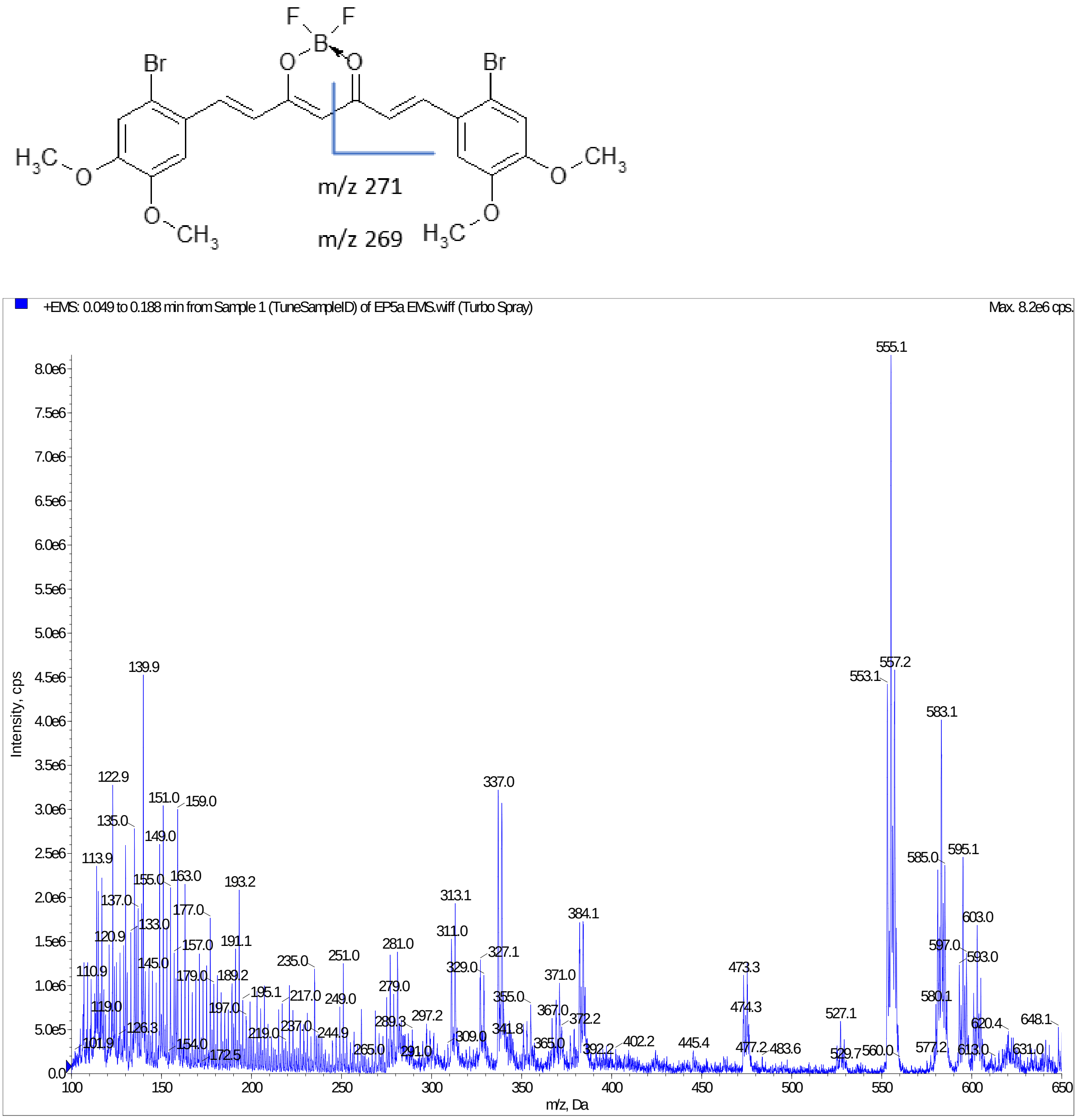

Figure S143. The compound **5a** $[M+H]^+$ ion fragmentation**.**

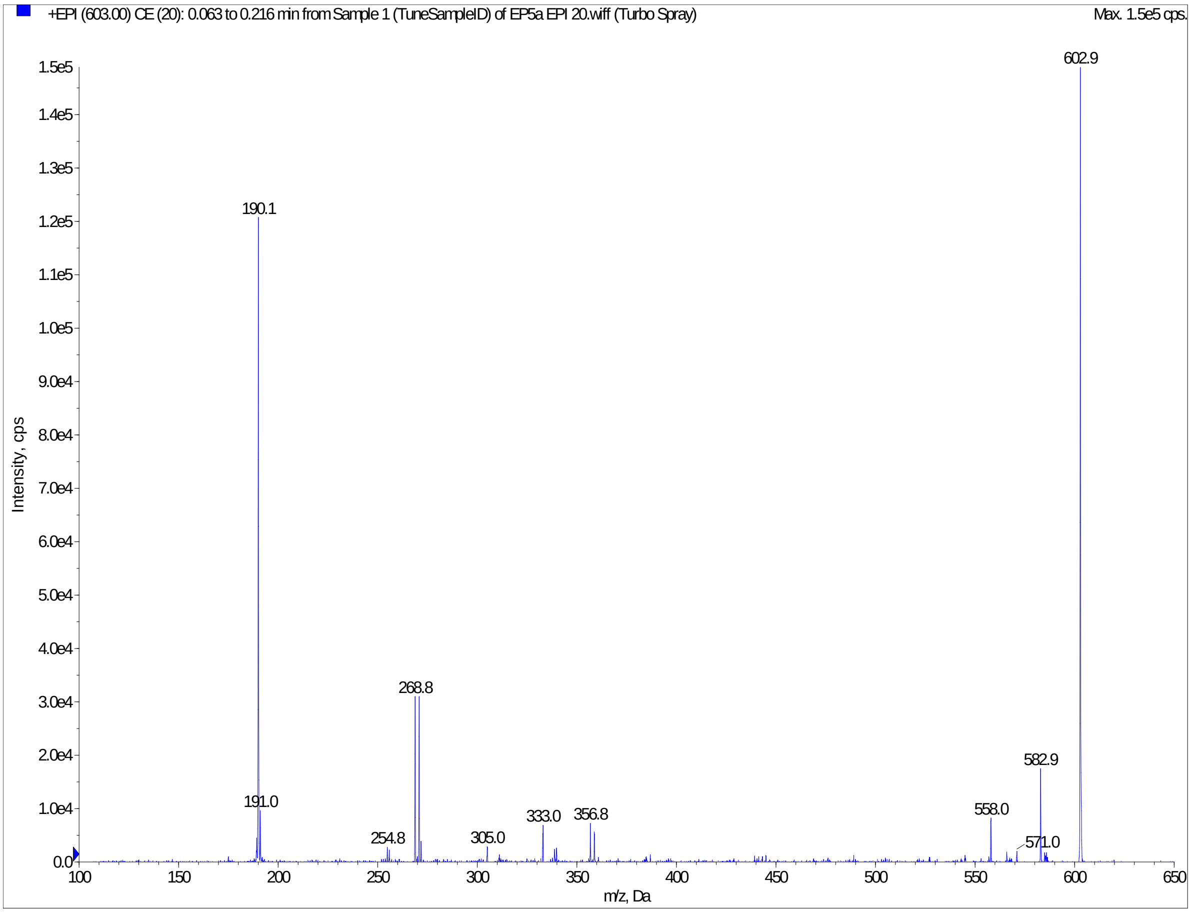

Figure S144. Mass spectra of compound **5b.**

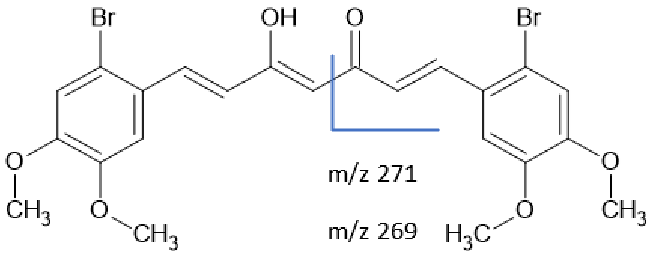

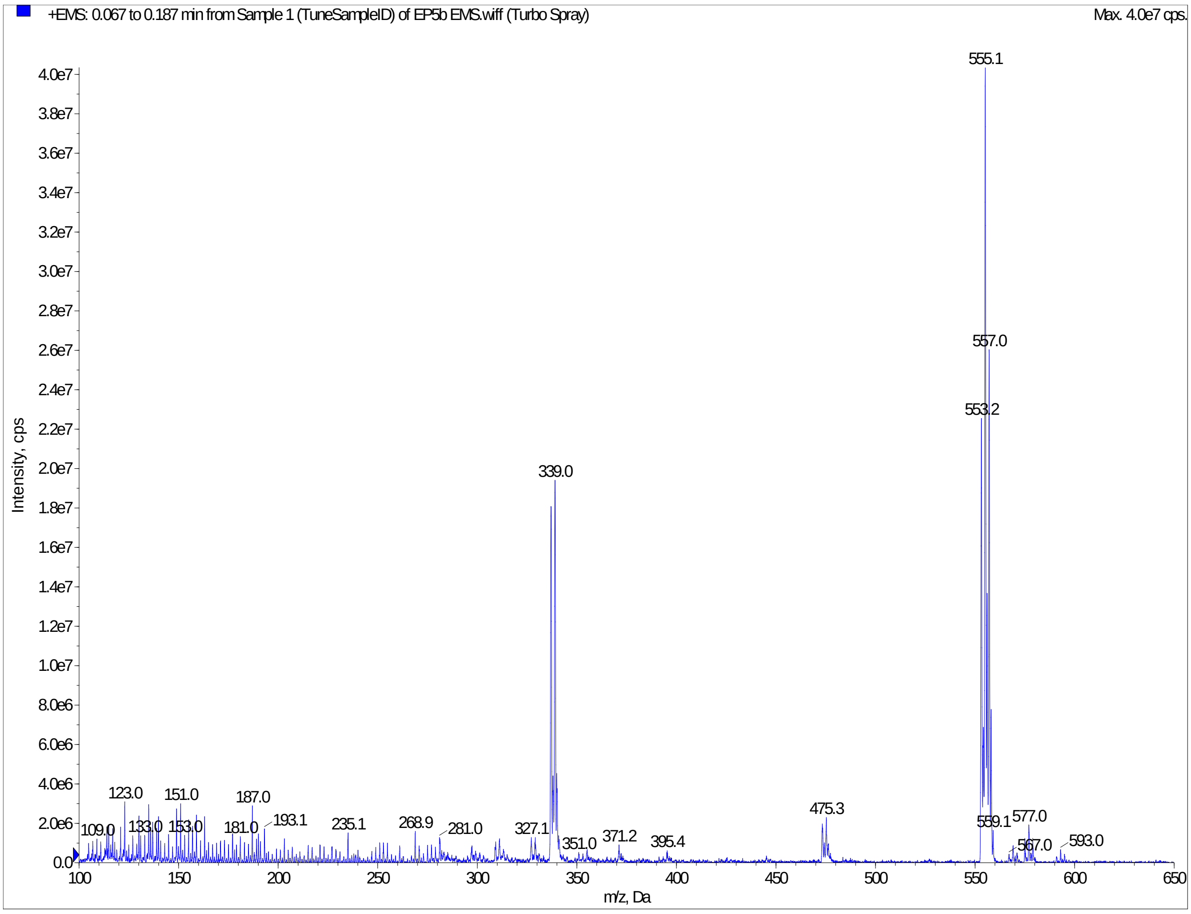

Figure S145. The compound **5b** $[M+H]^+$ ion fragmentation.

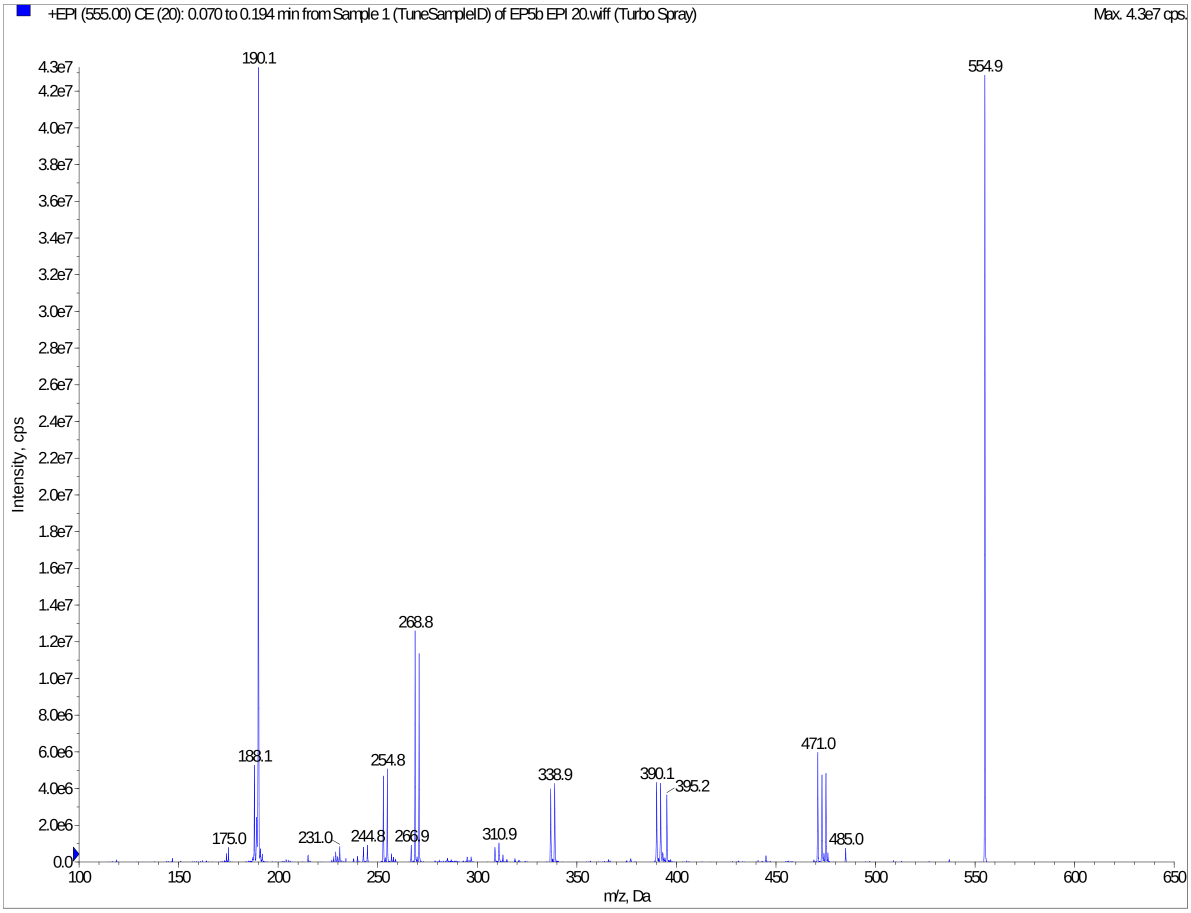

Figure S146. Mass spectra of compound **6a.**

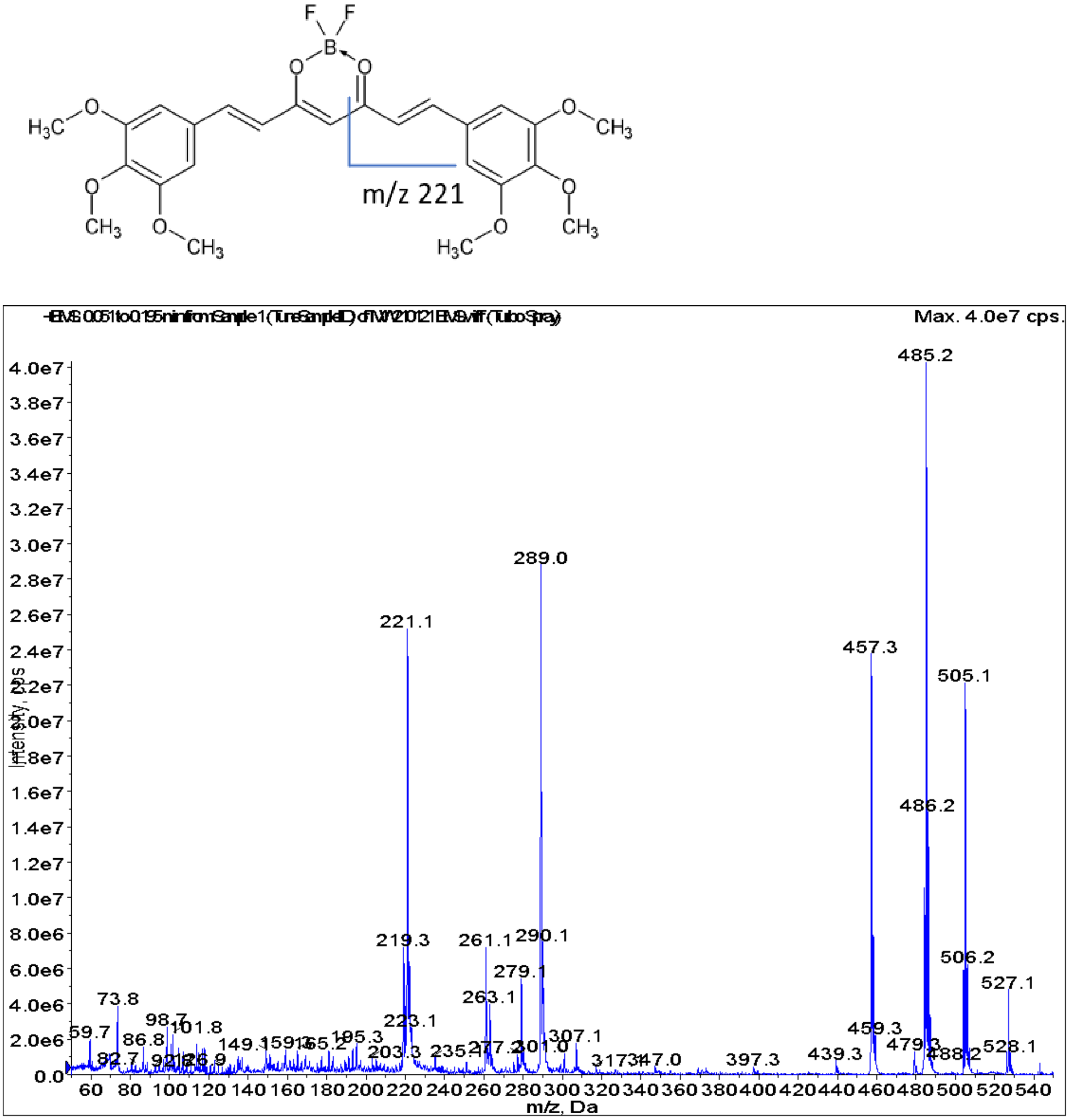

Figure S147. The compound **6a** $[M+H]^+$ ion fragmentation.

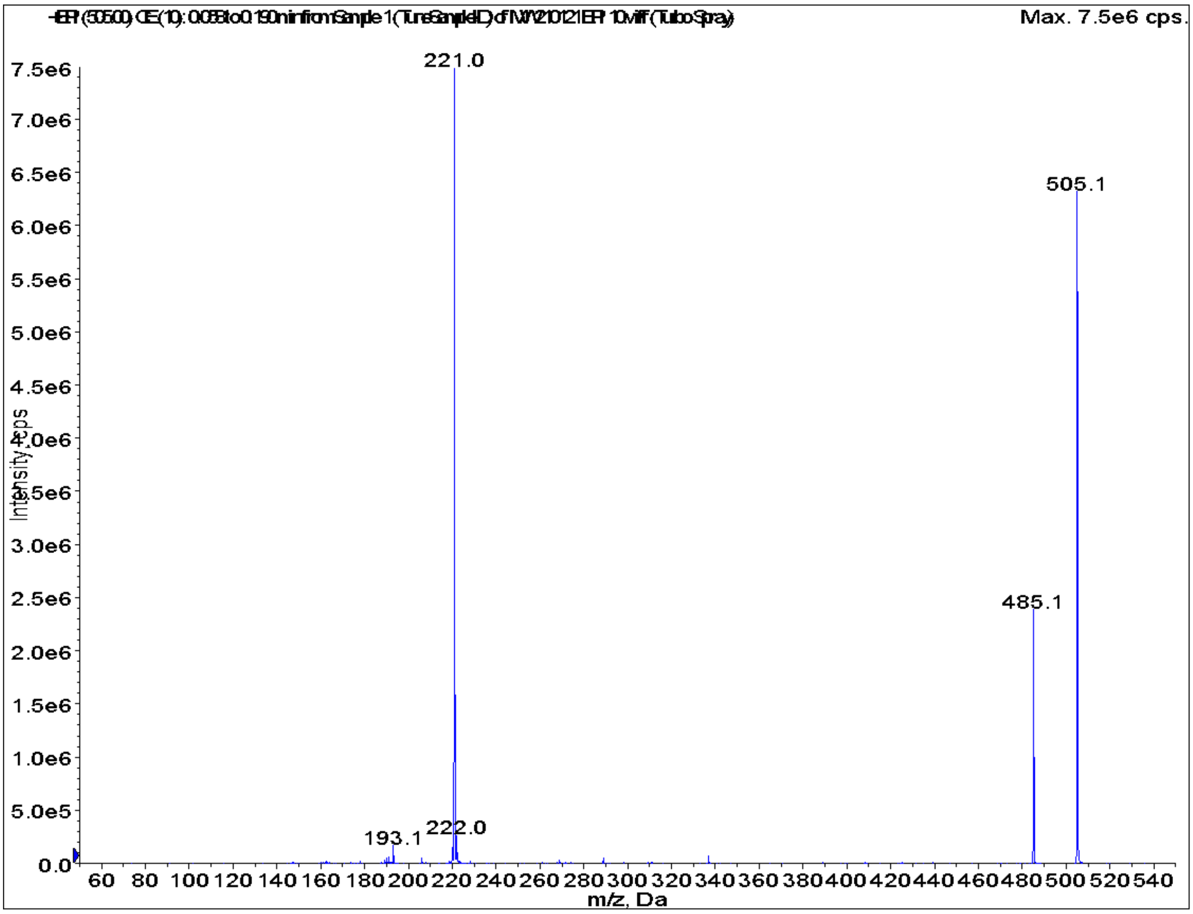

Figure S148. Mass spectra of compound **6b.**

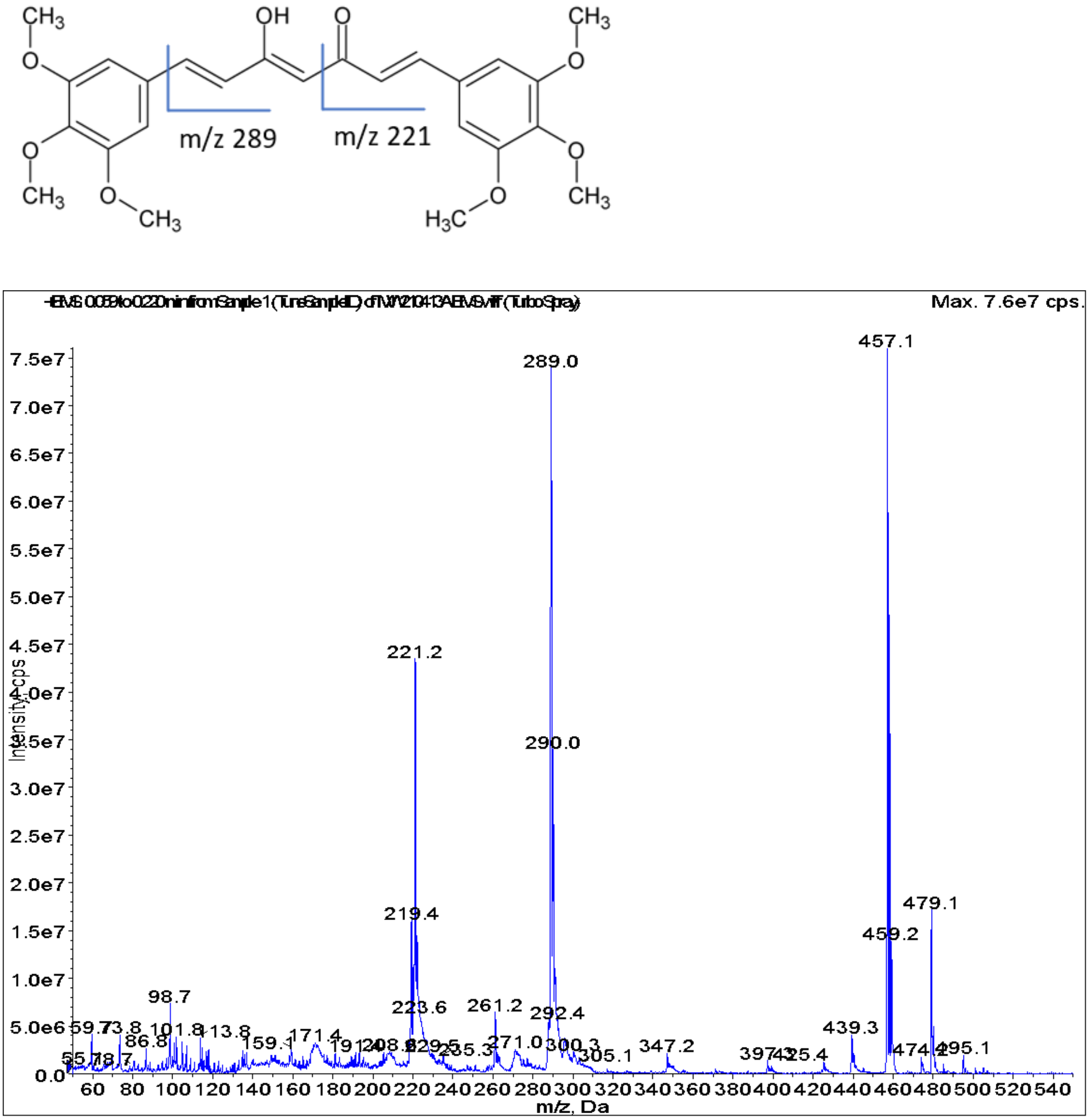

Figure S149. The compound **6b** $[M+H]^+$ ion fragmentation.

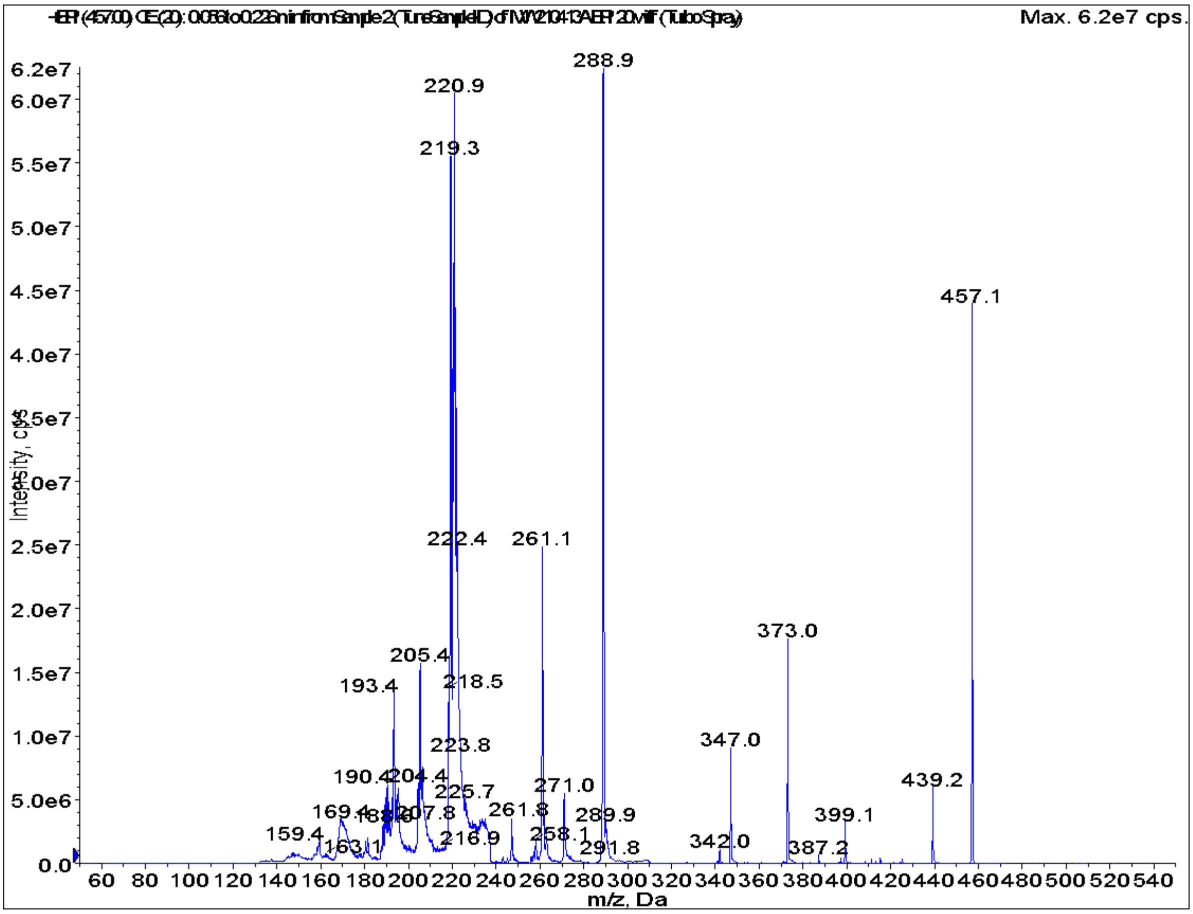

Figure S150. Mass spectra of compound **7a.**

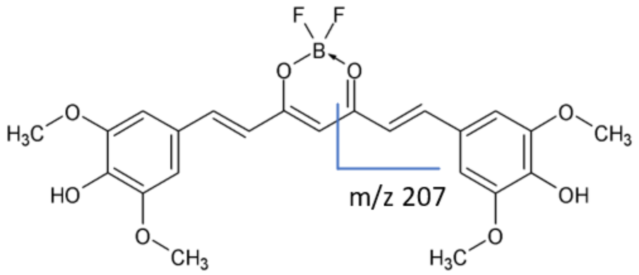

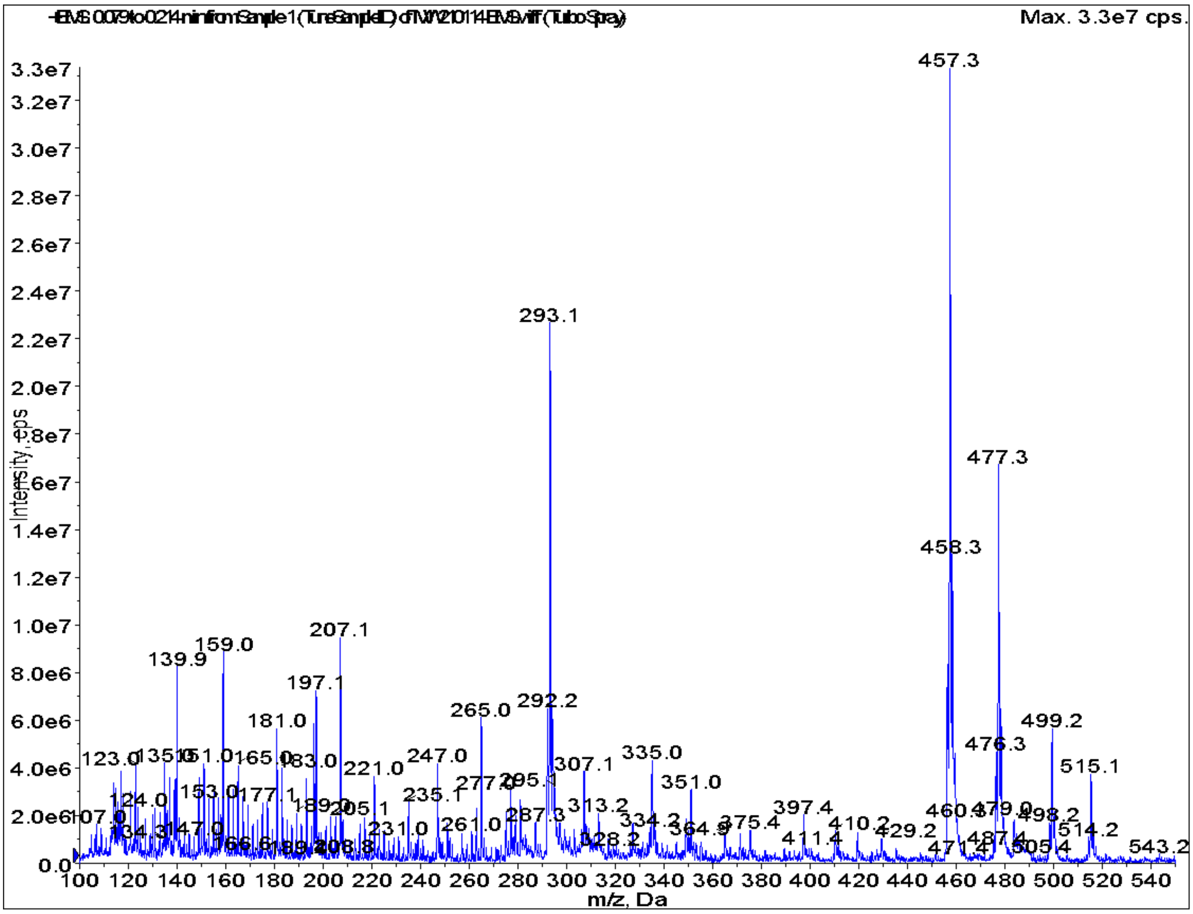

Figure S151. The compound **7a** $[M+H]^+$ ion fragmentation.

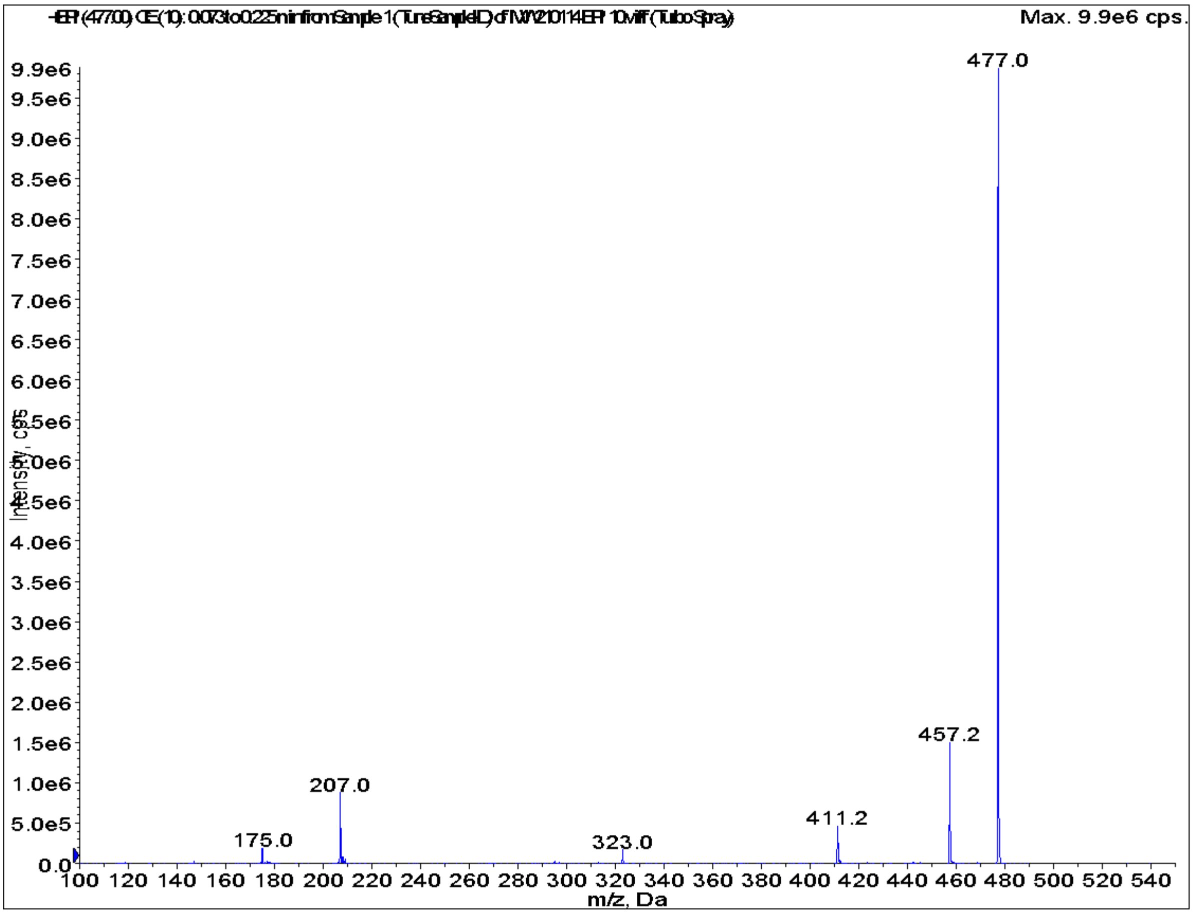

Figure S152. Mass spectra of compound **7b.**

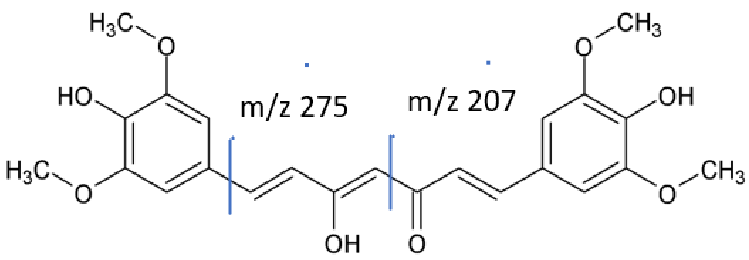

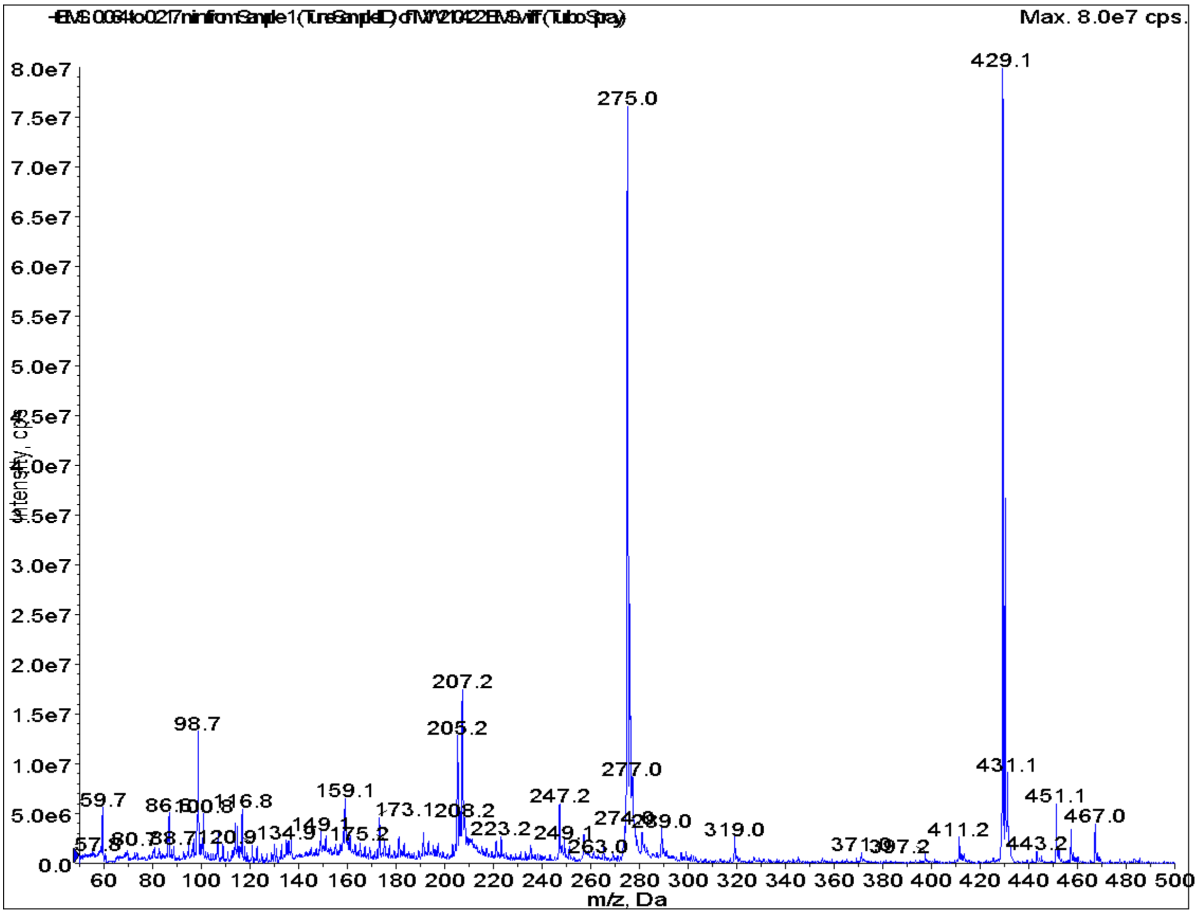

Figure S153. The compound **7b** [M+H]+ ion fragmentation.

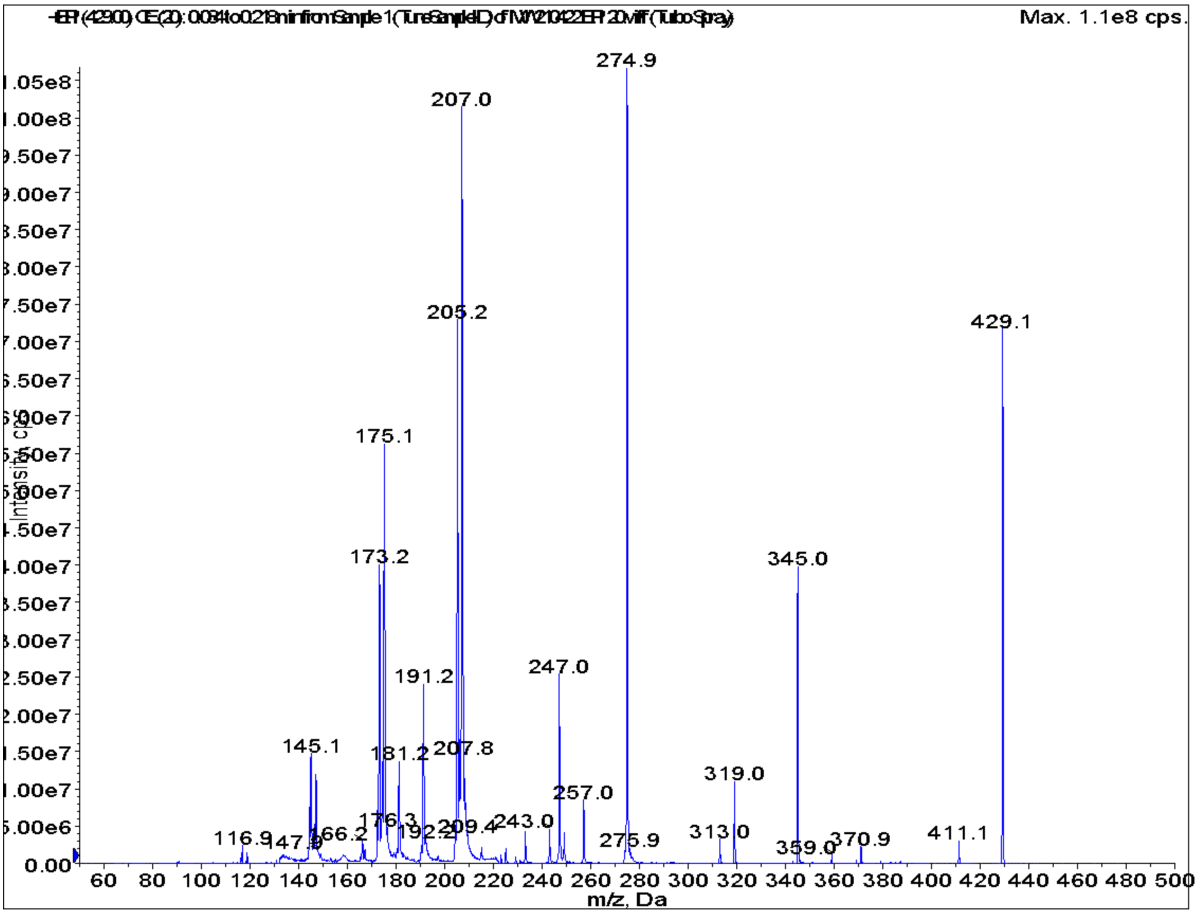

Figure S154. Mass spectra of compound **8a.**

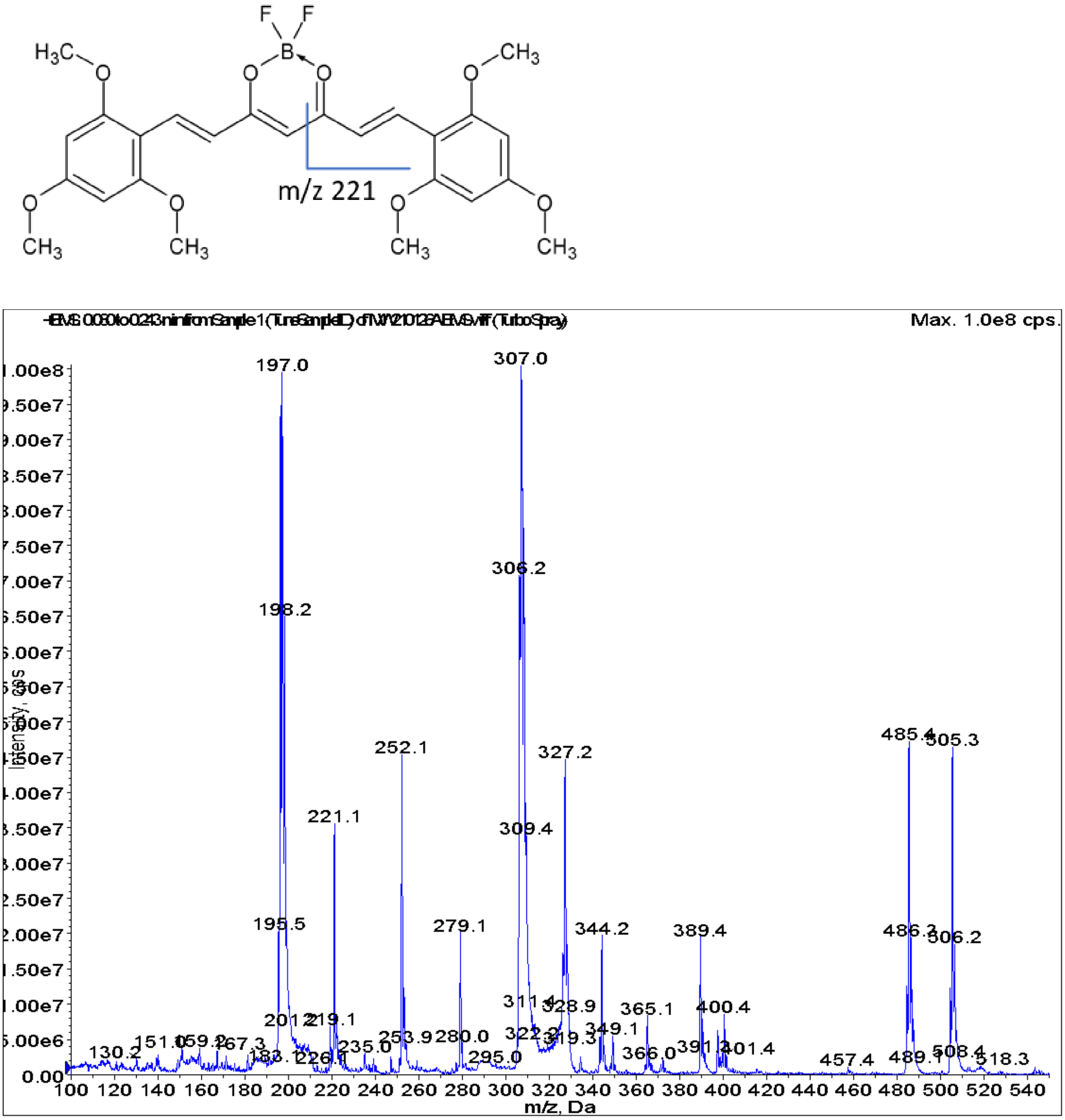

Figure S155. The compound **8a** $[M+H]^+$ ion fragmentation.

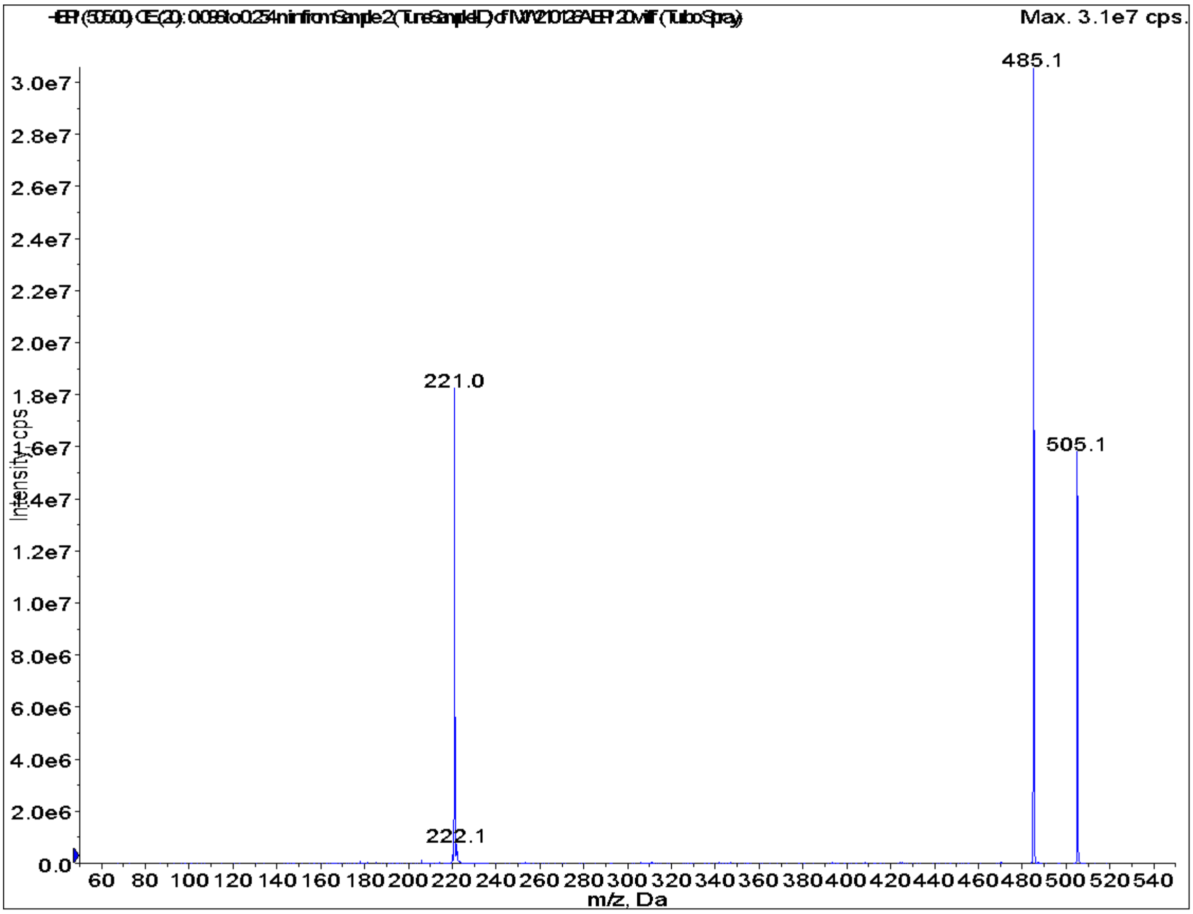

Figure S156. Mass spectra of compound **8b.**

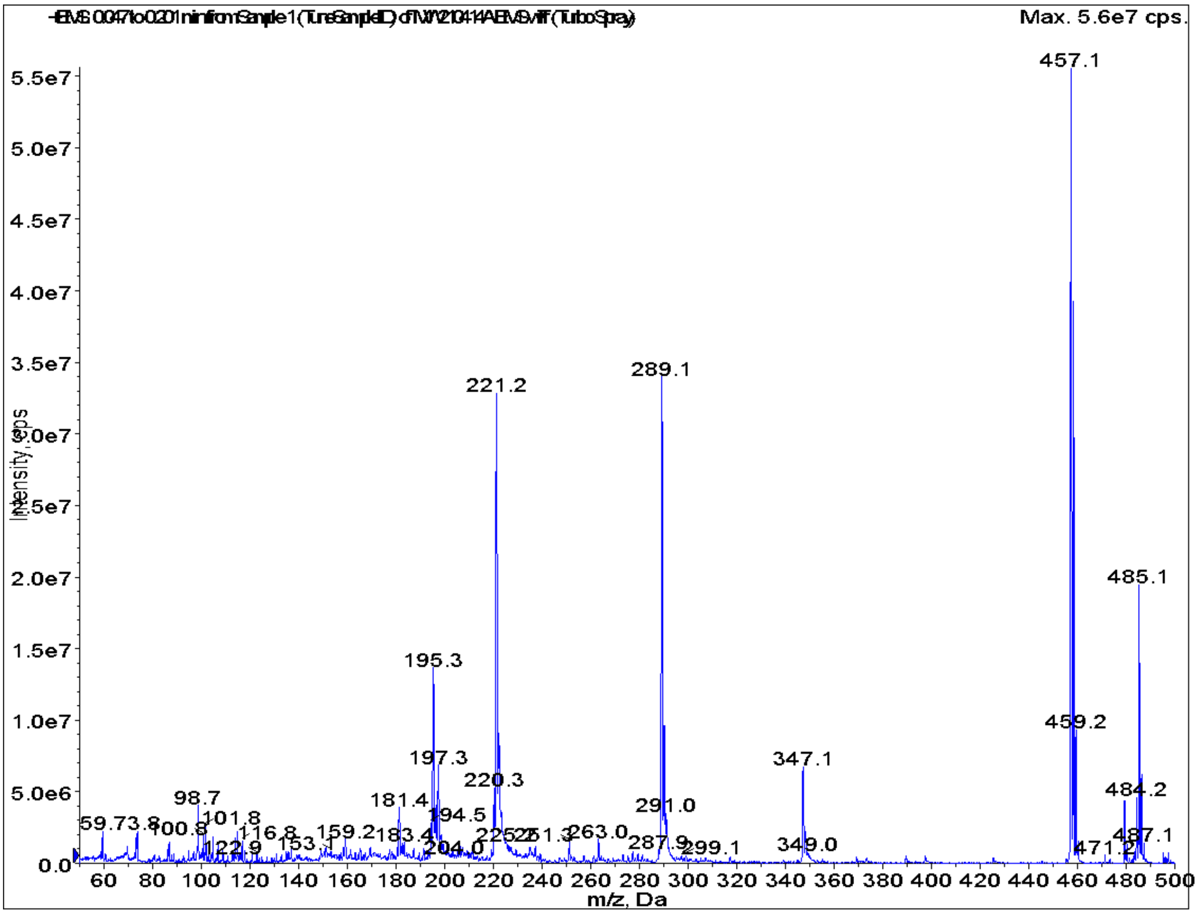

Figure S157. Mass spectra of compound **9a.**

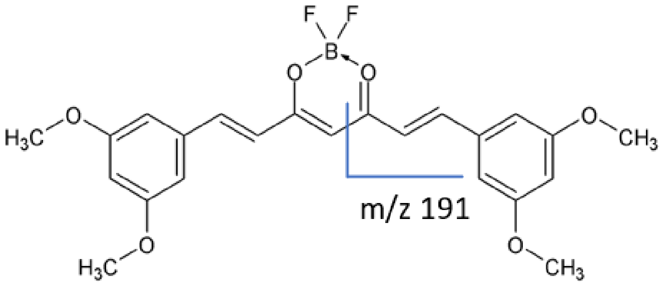

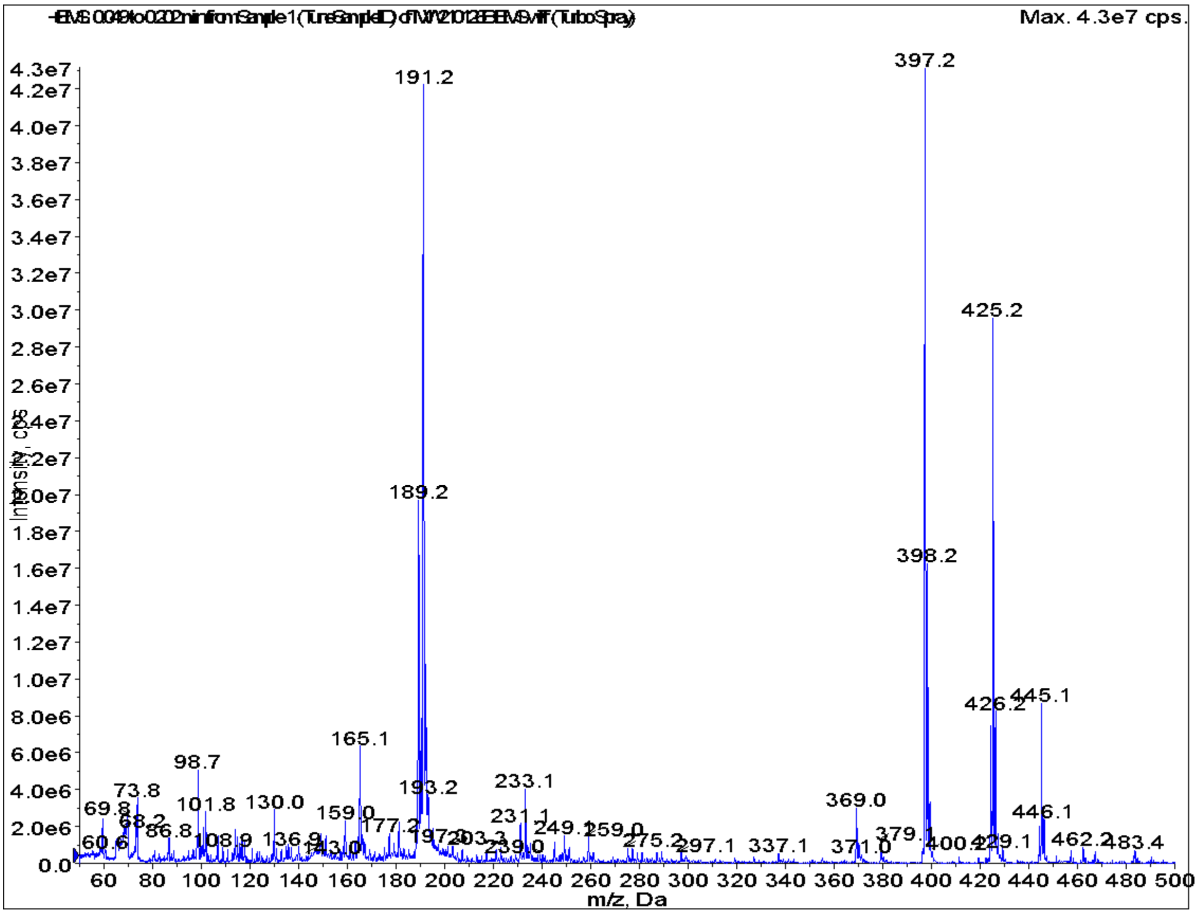

Figure S158. The compound **9a** $[M+H]^+$ ion fragmentation.

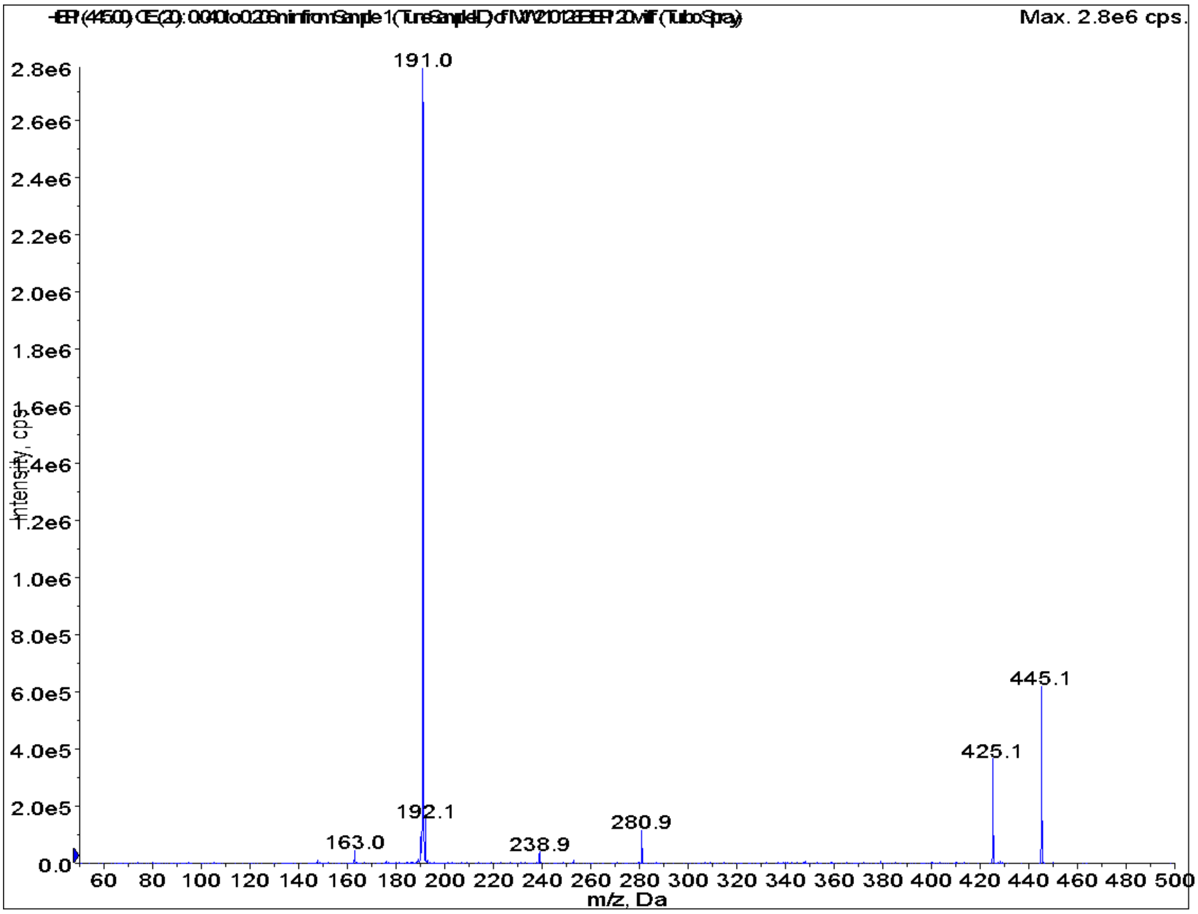

Figure S159. Mass spectra of compound **9b.**

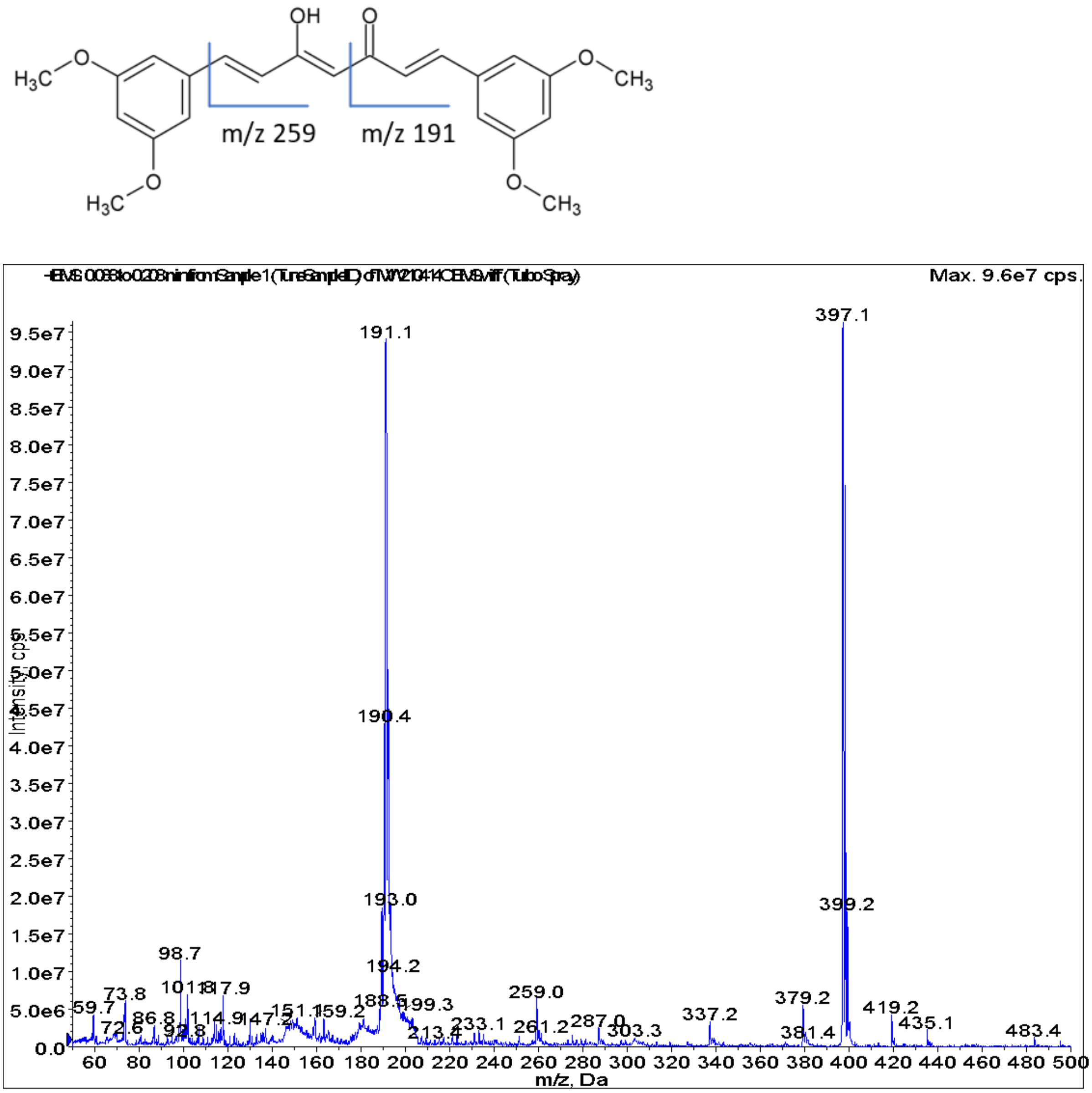

Figure S160. The compound **9b** [M+H]+ ion fragmentation.

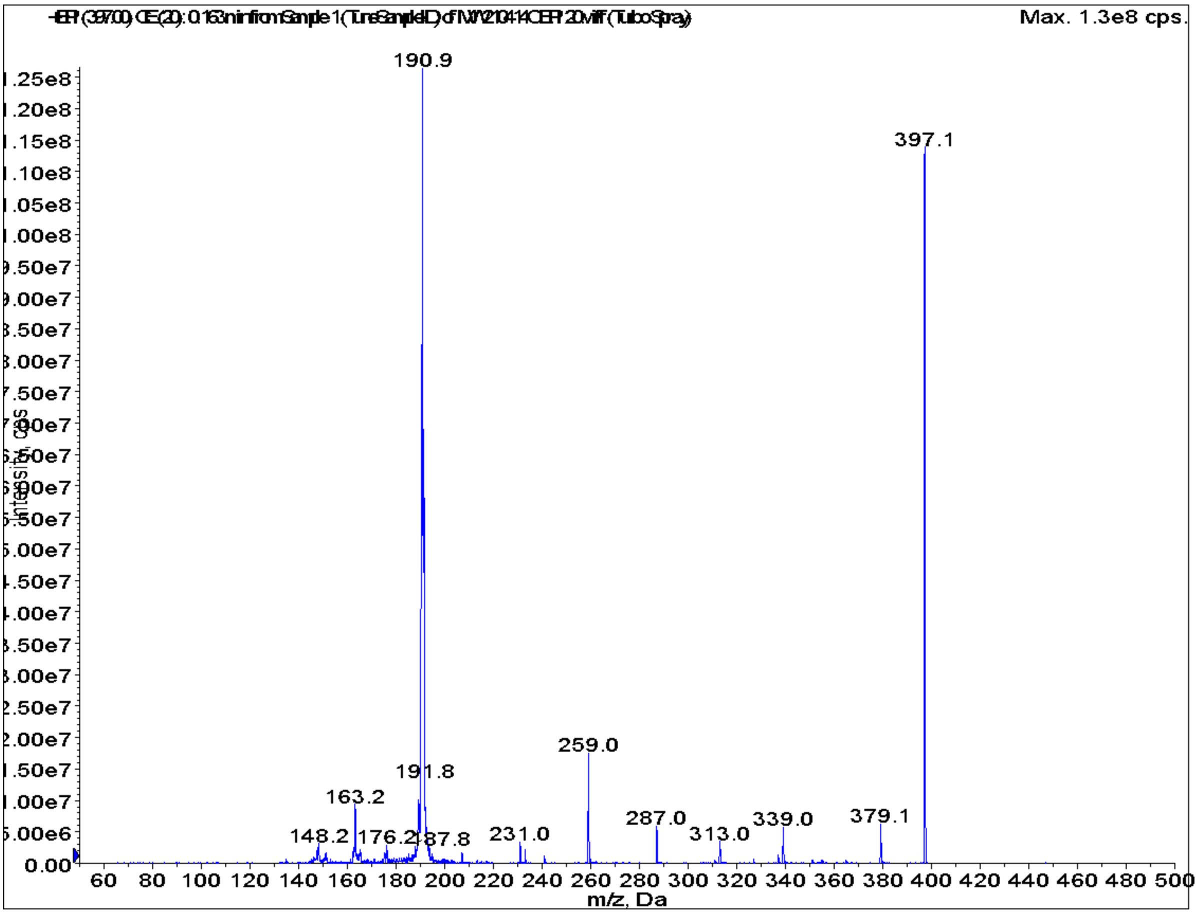

Figure S161. Mass spectra of compound **10a.**

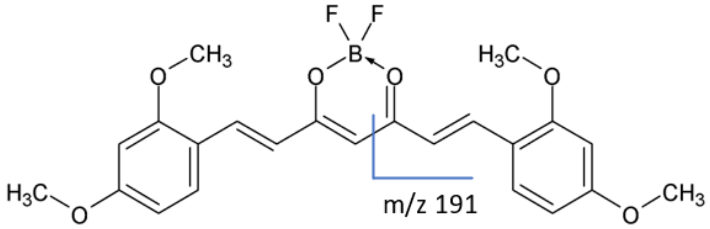

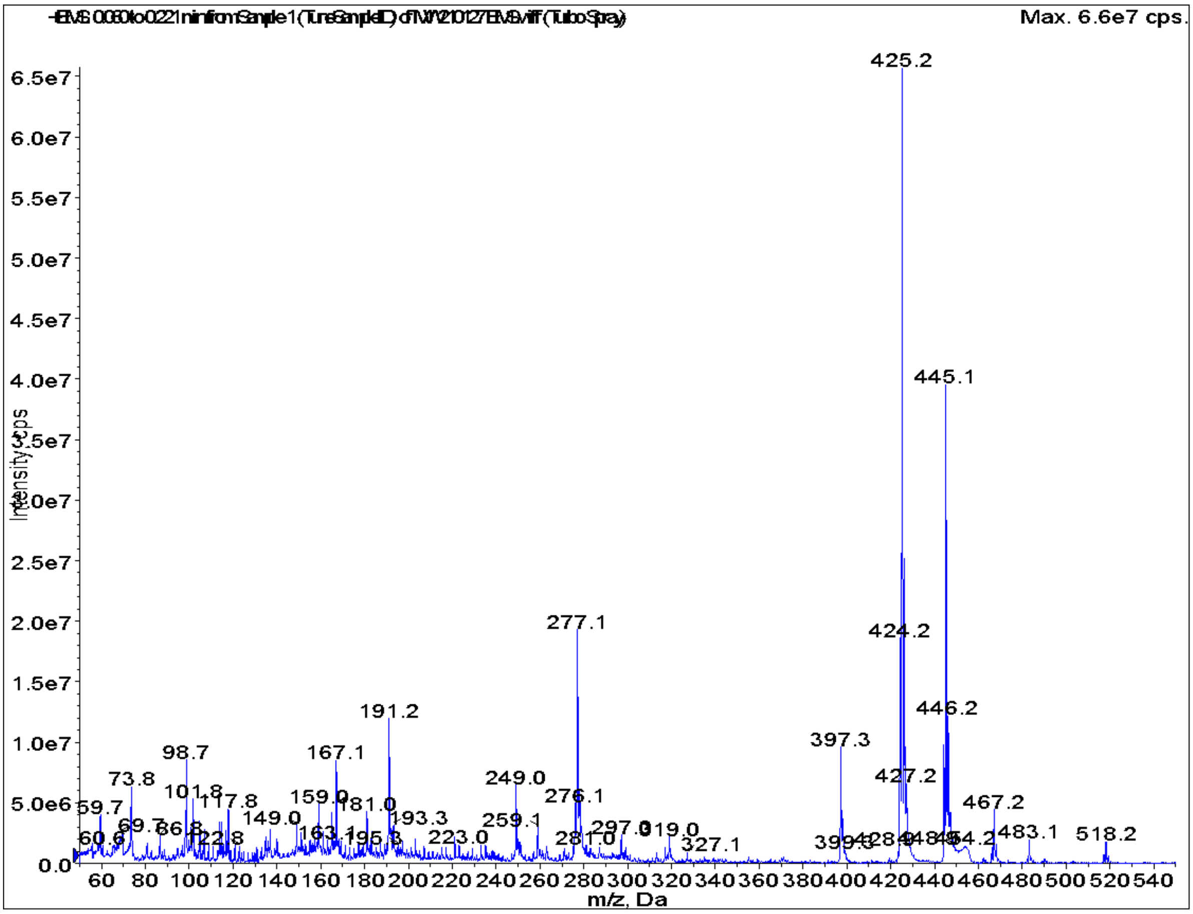

Figure S162. The compound **10a** [M+H]+ ion fragmentation.

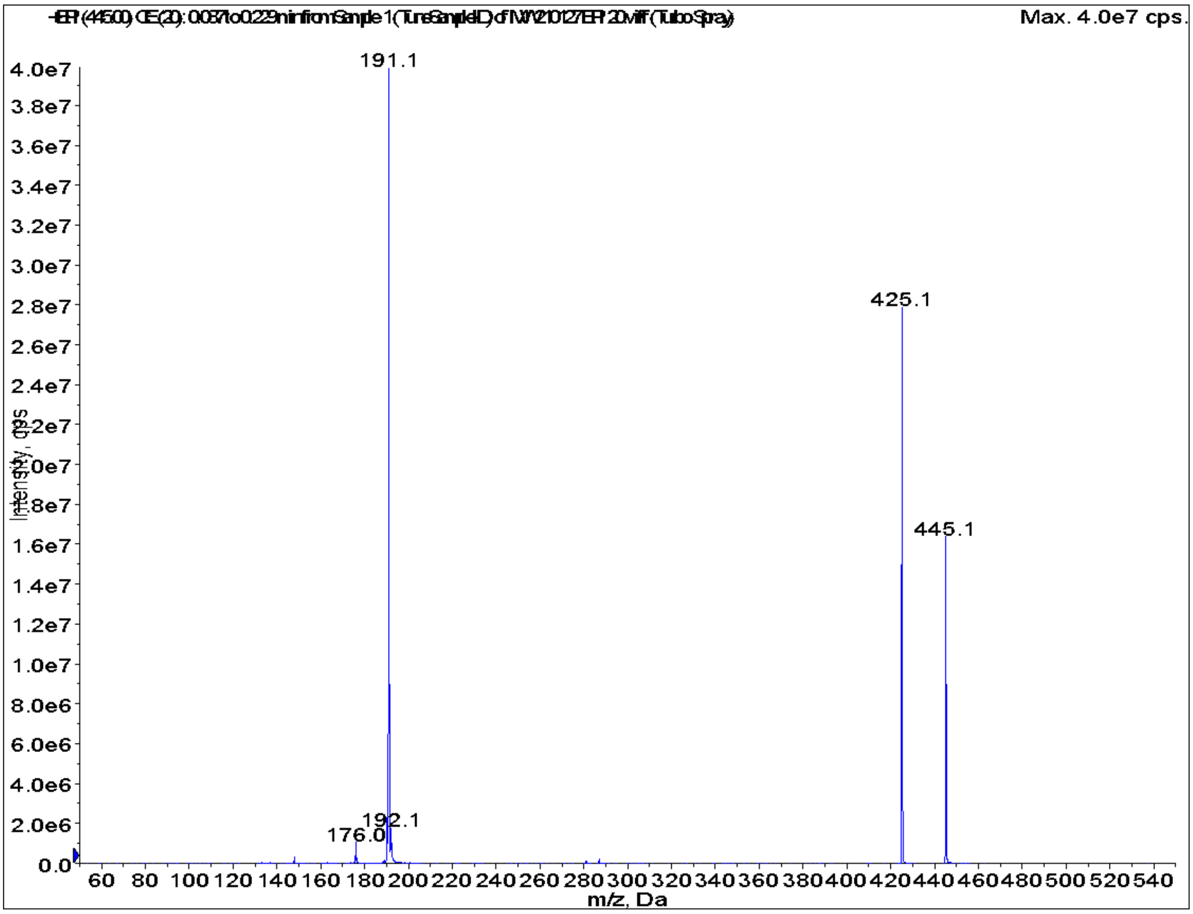

Figure S163. Mass spectra of compound **10b.**

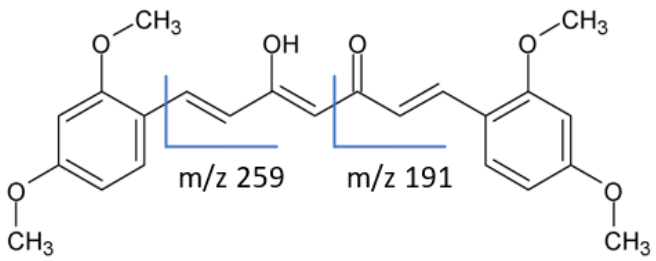

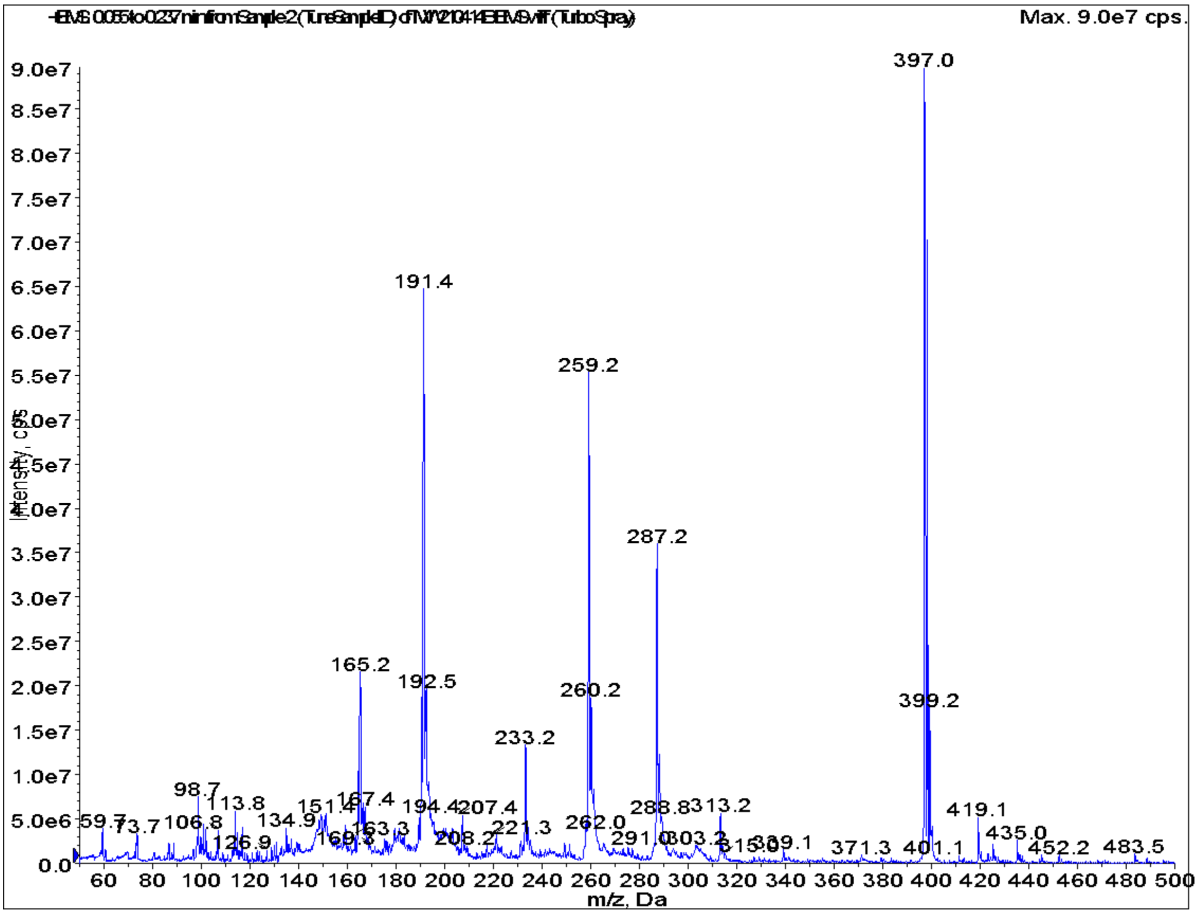

Figure S164. The compound **10b** $[M+H]^+$ ion fragmentation.

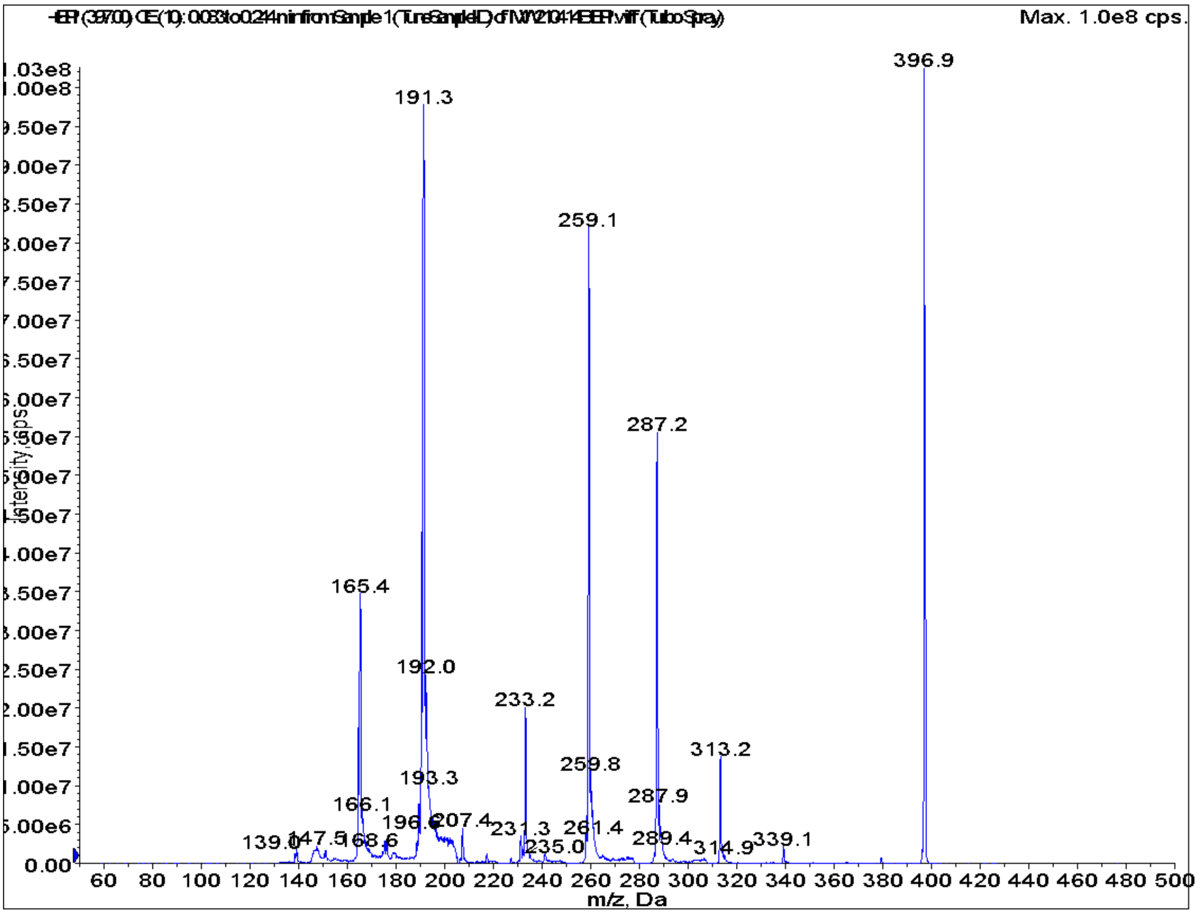

3. Crystallographic data

Table S1. Fractional Atomic Coordinates (×10^4) and Equivalent Isotropic Displacement Parameters (Å^2×10^3) for **8a**. U_{eq} is defined as 1/3 of the trace of the orthogonalised U_{IJ} tensor.

Atom	*x*	*y*	*z*	U(eq)
F35	7177.3(5)	5004.2(11)	6409.7(4)	21.9(2)
F36	7914.8(5)	2454.4(11)	6356.7(4)	21.2(2)
O15	7184.1(6)	5920.8(14)	2905.6(5)	18.3(2)
O31	3286.3(6)	1263.4(14)	4946.5(5)	18.3(2)
O8	6455.0(6)	2528.5(13)	5924.3(5)	18.4(2)
O33	7516.8(6)	4067.0(14)	5381.6(5)	18.3(2)
O29	1696.6(6)	-1412.2(14)	6451.0(5)	20.8(2)
O19	9589.4(6)	7010.0(15)	4570.9(5)	24.0(3)
O27	4667.3(6)	162.0(15)	7160.0(5)	23.5(3)
O17	9723.1(6)	8591.4(14)	2319.6(5)	21.7(2)
C9	8394.0(8)	6401.8(19)	3737.3(7)	14.8(3)
C14	8001.8(8)	6536.3(19)	3056.7(7)	15.0(3)
C22	3230.9(9)	574.8(18)	5559.5(7)	14.6(3)
C2	5022.8(9)	2128.1(19)	5373.5(7)	16.3(3)
C1	4804.0(9)	1344.7(19)	5922.8(7)	15.4(3)
C4	6095.2(9)	3674(2)	4830.2(7)	17.0(3)
C24	2463.9(9)	-767.8(19)	6352.8(7)	16.1(3)
C5	6933.7(9)	4244.4(19)	4833.3(7)	15.2(3)
C26	3934.9(8)	14.1(19)	6692.3(7)	15.9(3)
C23	2483.1(9)	-143.6(19)	5706.0(7)	16.2(3)
C12	9256.5(9)	7859.3(19)	2758.7(7)	17.0(3)
C6	7197.1(9)	5005.7(19)	4250.9(7)	16.5(3)
C3	5874.9(9)	2790.8(19)	5378.3(7)	15.5(3)
C7	8002.6(9)	5611.8(19)	4260.8(7)	15.5(3)
C10	9238.9(9)	7098.5(19)	3904.4(7)	17.2(3)
C25	3187.0(9)	-693.4(19)	6853.6(7)	16.6(3)
C21	3995.3(9)	648.3(19)	6042.3(7)	14.7(3)
C13	8427.2(9)	7239.5(19)	2566.9(7)	16.9(3)
C11	9670.9(9)	7807(2)	3425.7(7)	18.4(3)
C32	2511.9(9)	1382(2)	4462.3(7)	19.3(3)
C30	1631.6(9)	-1976(2)	7121.3(7)	21.7(3)
C16	6775.6(9)	5940(2)	2213.6(7)	23.9(4)
C28	4660.9(10)	-497(2)	7822.5(7)	26.6(4)
C18	9374.0(10)	8509(2)	1617.7(7)	25.5(4)
C20	10438.8(9)	7692(2)	4774.1(8)	27.1(4)
B34	7269.7(10)	3515(2)	6026.5(8)	16.8(3)

Table S2. Anisotropic Displacement Parameters (Å^2×10^3) for **8a**. The Anisotropic displacement factor exponent takes the form: $-2\pi^2[h^2a^{*2}U_{11}+2hka^*b^*U_{12}+\ldots]$.

Atom	U_{11}	U_{22}	U_{33}	U_{23}	U_{13}	U_{12}
F35	25.6(5)	22.2(5)	19.4(4)	-3.4(4)	7.9(3)	-3.9(4)

F36	18.4(4)	24.9(5)	19.5(4)	2.2(4)	0.9(3)	-1.0(4)
O15	14.1(5)	26.7(6)	14.2(5)	0.3(4)	2.7(4)	-5.3(4)
O31	17.2(5)	24.6(6)	13.0(5)	2.1(4)	1.8(4)	-3.4(4)
O8	16.6(5)	23.3(6)	15.3(5)	2.8(4)	2.5(4)	-4.1(4)
O33	15.6(5)	25.1(6)	14.4(5)	0.5(4)	2.9(4)	-4.4(4)
O29	14.9(5)	28.4(6)	19.7(5)	1.6(5)	5.0(4)	-5.7(4)
O19	16.1(5)	36.8(7)	18.4(5)	1.2(5)	1.0(4)	-6.5(5)
O27	14.0(5)	39.3(7)	16.7(5)	9.8(5)	0.9(4)	-3.4(5)
O17	14.9(5)	28.5(6)	23.2(5)	6.9(5)	7.8(4)	-2.5(4)
C9	14.3(7)	14.3(7)	16.9(7)	-1.0(6)	5.2(5)	1.7(6)
C14	12.1(7)	13.7(7)	19.7(7)	-0.7(6)	4.3(5)	0.8(6)
C22	18.3(7)	12.5(7)	14.0(7)	-0.8(6)	5.3(5)	1.1(6)
C2	15.6(7)	17.3(7)	15.9(7)	-0.1(6)	2.0(5)	0.4(6)
C1	14.8(7)	15.4(7)	16.0(7)	-3.1(6)	2.3(5)	-0.3(6)
C4	16.2(7)	19.2(8)	15.5(7)	2.2(6)	2.5(5)	0.0(6)
C24	13.9(7)	13.8(7)	21.9(7)	-1.8(6)	6.4(6)	-0.8(6)
C5	17.5(7)	12.8(7)	15.5(7)	-2.8(6)	3.9(5)	0.4(6)
C26	12.4(7)	18.1(8)	16.6(7)	0.5(6)	0.3(5)	2.1(6)
C23	14.3(7)	17.1(8)	16.7(7)	-1.4(6)	1.0(5)	0.0(6)
C12	14.9(7)	14.7(7)	23.7(7)	2.8(6)	9.9(6)	2.4(6)
C6	15.8(7)	19.7(8)	14.5(7)	-0.5(6)	3.8(5)	0.7(6)
C3	17.0(7)	14.5(7)	15.3(7)	-3.1(6)	3.9(5)	1.7(6)
C7	18.0(7)	14.7(7)	14.0(7)	-1.5(6)	3.7(5)	1.8(6)
C10	15.9(7)	17.3(8)	18.2(7)	-0.4(6)	2.6(6)	2.6(6)
C25	16.1(7)	18.4(8)	16.1(7)	2.8(6)	5.2(5)	0.9(6)
C21	15.1(7)	12.9(7)	16.7(7)	-0.7(6)	4.7(5)	0.6(6)
C13	16.2(7)	18.9(8)	16.2(7)	2.1(6)	4.3(5)	2.9(6)
C11	11.9(7)	19.5(8)	24.4(8)	0.5(6)	4.3(6)	-1.7(6)
C32	20.4(7)	22.1(8)	14.3(7)	-0.2(6)	-0.2(6)	-0.4(6)
C30	18.7(7)	26.9(9)	20.9(7)	3.2(7)	7.5(6)	-4.2(6)
C16	17.7(7)	37.7(10)	15.6(7)	1.3(7)	1.1(6)	-6.6(7)
C28	19.0(8)	44.7(11)	15.5(7)	9.0(7)	1.3(6)	-1.5(7)
C18	21.1(8)	35.0(10)	22.2(8)	9.4(7)	8.8(6)	0.4(7)
C20	15.0(7)	39.6(10)	25.2(8)	-0.2(8)	-1.0(6)	-5.5(7)
B34	15.6(8)	20.4(9)	14.9(7)	-0.4(7)	4.4(6)	-3.0(7)

Table S3. Bond Lengths for **8a**.

**Atom**	**Atom**	**Length/Å**		**Atom**	**Atom**	**Length/Å**
F35	B34	1.3917(19)		C9	C7	1.4420(19)
F36	B34	1.3790(18)		C9	C10	1.4225(19)
O15	C14	1.3592(16)		C14	C13	1.3923(19)
O15	C16	1.4343(16)		C22	C23	1.3786(19)
O31	C22	1.3587(16)		C22	C21	1.4176(19)
O31	C32	1.4326(16)		C2	C1	1.3531(19)
O8	C3	1.3224(16)		C2	C3	1.4361(19)
O8	B34	1.4738(18)		C1	C21	1.4409(19)
O33	C5	1.3200(16)		C4	C5	1.3933(19)

O33	B34	1.4805(18)		C4	C3	1.3870(19)
O29	C24	1.3532(16)		C24	C23	1.3930(19)
O29	C30	1.4379(17)		C24	C25	1.3913(19)
O19	C10	1.3653(16)		C5	C6	1.4328(19)
O19	C20	1.4321(17)		C26	C25	1.3865(19)
O27	C26	1.3675(16)		C26	C21	1.4148(19)
O27	C28	1.4284(16)		C12	C13	1.384(2)
O17	C12	1.3636(16)		C12	C11	1.393(2)
O17	C18	1.4302(17)		C6	C7	1.3507(19)
C9	C14	1.4102(19)		C10	C11	1.383(2)

Table S4. Bond Angles for **8a**.

Atom	Atom	Atom	Angle/°		Atom	Atom	Atom	Angle/°
C14	O15	C16	117.89(10)		O27	C26	C21	115.36(12)
C22	O31	C32	117.67(11)		C25	C26	C21	122.95(12)
C3	O8	B34	120.33(11)		C22	C23	C24	119.44(13)
C5	O33	B34	120.91(11)		O17	C12	C13	123.24(13)
C24	O29	C30	117.02(11)		O17	C12	C11	114.91(12)
C10	O19	C20	117.85(11)		C13	C12	C11	121.83(13)
C26	O27	C28	118.45(11)		C7	C6	C5	122.00(13)
C12	O17	C18	117.68(11)		O8	C3	C2	117.98(12)
C14	C9	C7	124.77(12)		O8	C3	C4	120.25(12)
C14	C9	C10	116.29(12)		C4	C3	C2	121.77(12)
C10	C9	C7	118.94(12)		C6	C7	C9	130.67(13)
O15	C14	C9	115.90(12)		O19	C10	C9	114.96(12)
O15	C14	C13	121.91(12)		O19	C10	C11	122.72(13)
C13	C14	C9	122.19(12)		C11	C10	C9	122.32(13)
O31	C22	C23	122.53(12)		C26	C25	C24	118.24(13)
O31	C22	C21	115.48(12)		C22	C21	C1	125.17(12)
C23	C22	C21	121.99(12)		C26	C21	C22	116.03(12)
C1	C2	C3	121.24(13)		C26	C21	C1	118.79(12)
C2	C1	C21	130.99(13)		C12	C13	C14	118.73(13)
C3	C4	C5	120.97(13)		C10	C11	C12	118.57(13)
O29	C24	C23	115.03(12)		F35	B34	O8	108.65(11)
O29	C24	C25	123.67(12)		F35	B34	O33	109.00(12)
C25	C24	C23	121.30(13)		F36	B34	F35	110.25(12)
O33	C5	C4	119.98(12)		F36	B34	O8	109.04(12)
O33	C5	C6	117.96(12)		F36	B34	O33	107.81(12)
C4	C5	C6	122.05(12)		O8	B34	O33	112.08(11)
O27	C26	C25	121.69(12)					

Table S5. Torsion Angles for **8a**.

A	B	C	D	Angle/°		A	B	C	D	Angle/°
O15	C14	C13	C12	179.29(13)		C3	O8	B34	O33	-26.68(18)
O31	C22	C23	C24	-177.66(13)		C3	C2	C1	C21	177.49(14)

O31	C22	C21	C1	-1.5(2)		C3	C4	C5	O33	-5.2(2)
O31	C22	C21	C26	177.02(12)		C3	C4	C5	C6	173.83(13)
O33	C5	C6	C7	-3.2(2)		C7	C9	C14	O15	2.5(2)
O29	C24	C23	C22	178.60(13)		C7	C9	C14	C13	-176.90(13)
O29	C24	C25	C26	-179.33(13)		C7	C9	C10	O19	-2.71(19)
O19	C10	C11	C12	-179.25(13)		C7	C9	C10	C11	177.16(14)
O27	C26	C25	C24	178.78(13)		C10	C9	C14	O15	-177.88(12)
O27	C26	C21	C22	-177.34(12)		C10	C9	C14	C13	2.7(2)
O27	C26	C21	C1	1.29(19)		C10	C9	C7	C6	173.40(15)
O17	C12	C13	C14	-179.13(13)		C25	C24	C23	C22	-0.5(2)
O17	C12	C11	C10	179.46(13)		C25	C26	C21	C22	2.0(2)
C9	C14	C13	C12	-1.4(2)		C25	C26	C21	C1	-179.41(14)
C9	C10	C11	C12	0.9(2)		C21	C22	C23	C24	2.1(2)
C14	C9	C7	C6	-7.0(2)		C21	C26	C25	C24	-0.5(2)
C14	C9	C10	O19	177.62(12)		C13	C12	C11	C10	0.7(2)
C14	C9	C10	C11	-2.5(2)		C11	C12	C13	C14	-0.4(2)
C2	C1	C21	C22	3.4(3)		C32	O31	C22	C23	5.91(19)
C2	C1	C21	C26	-175.12(15)		C32	O31	C22	C21	-173.89(12)
C1	C2	C3	O8	3.6(2)		C30	O29	C24	C23	-176.42(13)
C1	C2	C3	C4	-176.73(14)		C30	O29	C24	C25	2.7(2)
C4	C5	C6	C7	177.76(14)		C16	O15	C14	C9	-176.61(13)
C5	O33	B34	F35	-96.40(14)		C16	O15	C14	C13	2.8(2)
C5	O33	B34	F36	143.92(12)		C28	O27	C26	C25	2.4(2)
C5	O33	B34	O8	23.91(18)		C28	O27	C26	C21	-178.24(13)
C5	C4	C3	O8	2.3(2)		C18	O17	C12	C13	-8.0(2)
C5	C4	C3	C2	-177.35(13)		C18	O17	C12	C11	173.23(13)
C5	C6	C7	C9	179.55(14)		C20	O19	C10	C9	-179.89(13)
C23	C22	C21	C1	178.68(14)		C20	O19	C10	C11	0.2(2)
C23	C22	C21	C26	-2.8(2)		B34	O8	C3	C2	-165.58(12)
C23	C24	C25	C26	-0.3(2)		B34	O8	C3	C4	14.76(19)
C3	O8	B34	F35	93.84(14)		B34	O33	C5	C4	-9.2(2)
C3	O8	B34	F36	-145.97(12)		B34	O33	C5	C6	171.79(13)

Table S6. Hydrogen Atom Coordinates (Å×10^4) and Isotropic Displacement Parameters (Å^2×10^3) for **8a**.

**Atom**	***x***	***y***	***z***	**U(eq)**
H2	4604.87	2241.79	4977.17	20
H1	5261.57	1235.15	6292.67	19
H4	5668.11	3892.82	4447.14	20
H23	1985.13	-212.28	5368.68	19
H6	6792.78	5086.67	3845.48	20
H7	8372.51	5499.02	4682.42	19
H25	3168.83	-1116.18	7294.32	20
H13	8153.55	7292.94	2109.75	20
H11	10238.1	8249.74	3549.23	22
H32A	2624.7	2034.42	4067.22	29
H32B	2071.85	1997.76	4662.44	29

H32C	2309.23	196.41	4326.2	29
H30A	1041.24	-2349.04	7135.09	33
H30B	1785.22	-998.48	7435.96	33
H30C	2024.02	-2963.2	7249.82	33
H16A	7108.56	5221.96	1946.86	36
H16B	6745.11	7152.46	2045.63	36
H16C	6193.88	5459.19	2175.51	36
H28A	4516.88	-1752.52	7798.28	40
H28B	4231.91	143.43	8026.39	40
H28C	5229.48	-335.28	8097.53	40
H18A	9790.26	8990.47	1356.98	38
H18B	8842.88	9199.44	1527.99	38
H18C	9249.5	7282.11	1487.1	38
H20A	10839.81	7046.91	4542.57	41
H20B	10610.06	7552.48	5261.72	41
H20C	10447.82	8942.41	4657.17	41

4. HPLC data

Table S7. HPLC gradient used for the analysis of new synthesized compounds.

**Time, min**	**A**	**B**
0	10	90
10	30	70
13	50	50
15	10	90

Table S8. Purity data for synthesized curcuminoids.

Symbol	C, mg/ml	λ_{max}, nm	t_R, min	%
1a	0.1	489	1.95	100.00
1b	0.1	414	2.58	100.00
2a	0.1	466	2.06	100.00
2b	0.1	403	2.54	95.37
3a	0.1	465	3.85	99.07
3b	0.1	402	6.9	100.00
4a	0.1	495	2.097	99.82
4b	0.1	417	2.79	95.12
5a	0.1	468	2.19	97.97
5b	0.1	404	2.77	100.00
6a	0.1	482	2.22	97.19
6b	0.1	410	2.68	96.14
7a	0.1	512	1.7	100.00
7b	0.1	426	1.82	98.00
8a	0.1	525	2.58	95.01
8b	0.1	431	3.1	95.03
9a	0.1	453	2.59	100.00
9b	0.1	394	3.43	97.00
10a	0.1	510	2.54	100.00
10b	0.1	425	3.37	100.00

Figure S165. HPLC chromatogram for **1a**.

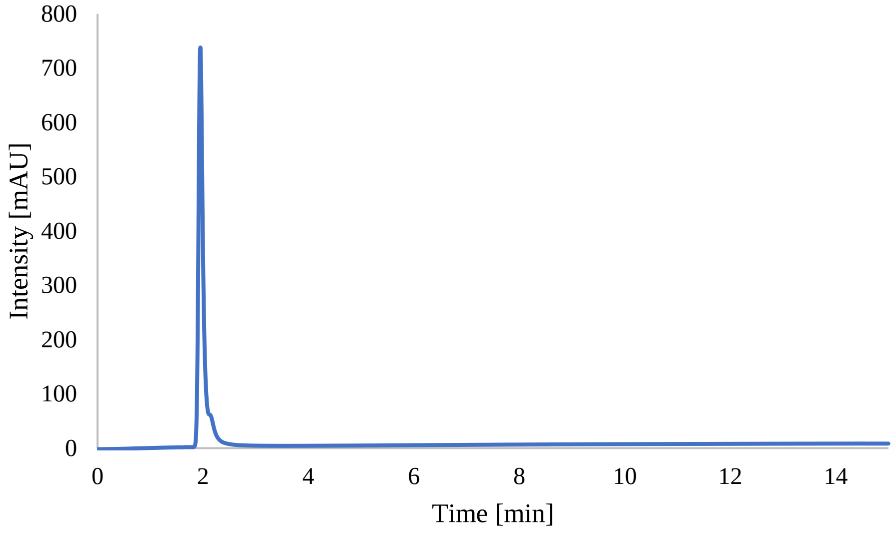

Figure S166. HPLC chromatogram for **1b**.

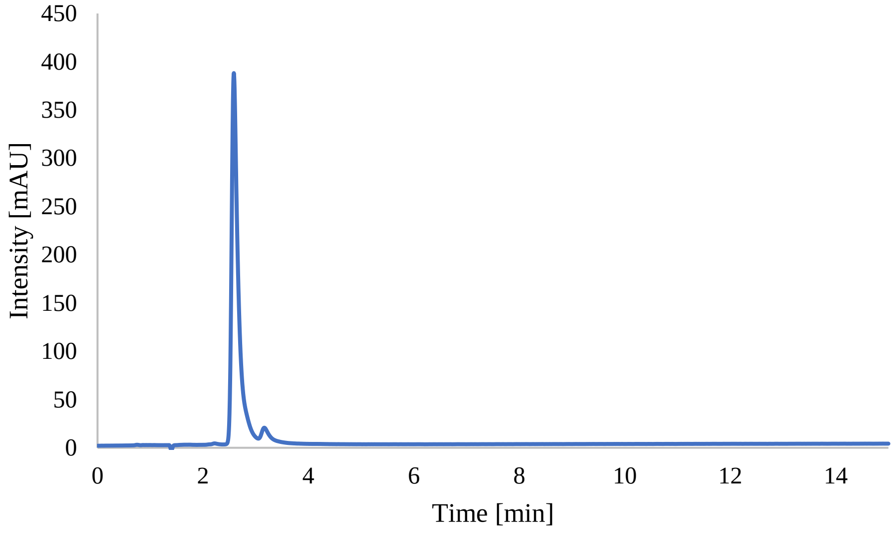

Figure S167. HPLC chromatogram for **2a**.

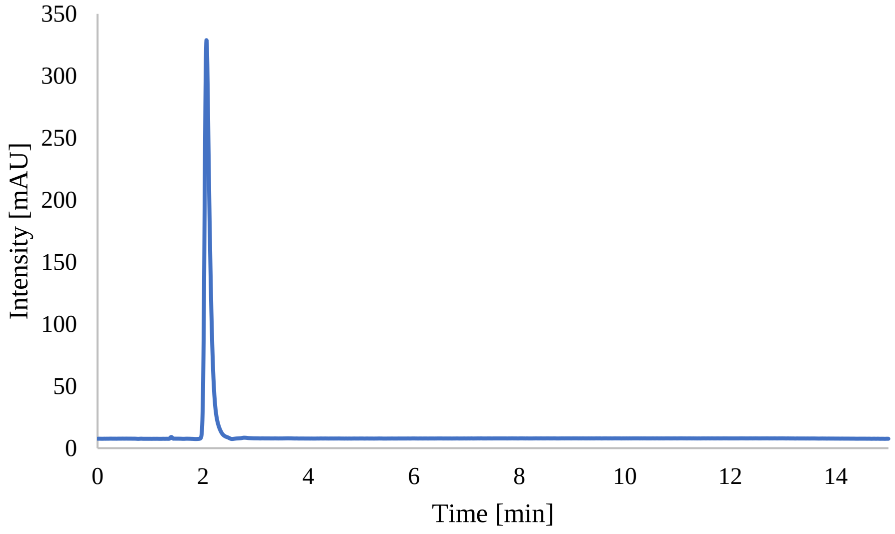

Figure S168. HPLC chromatogram for **2b**.

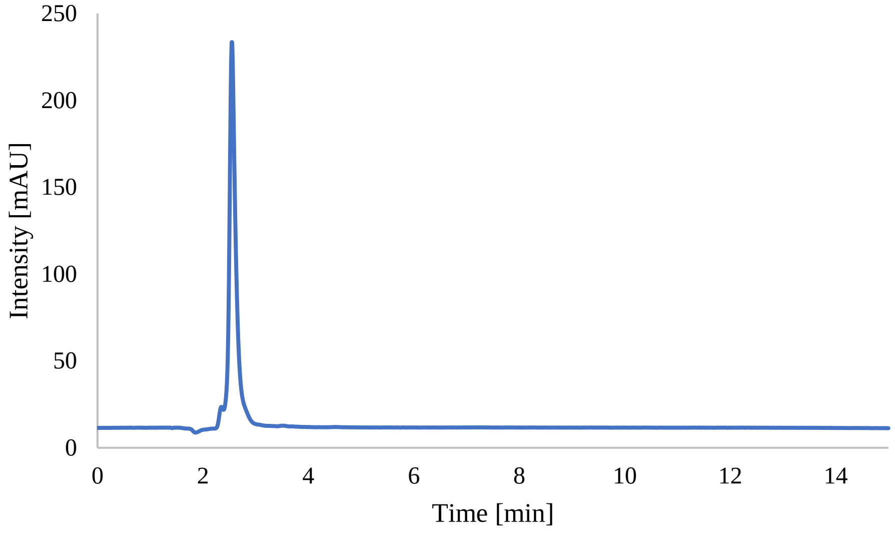

Figure S169. HPLC chromatogram for **3a**.

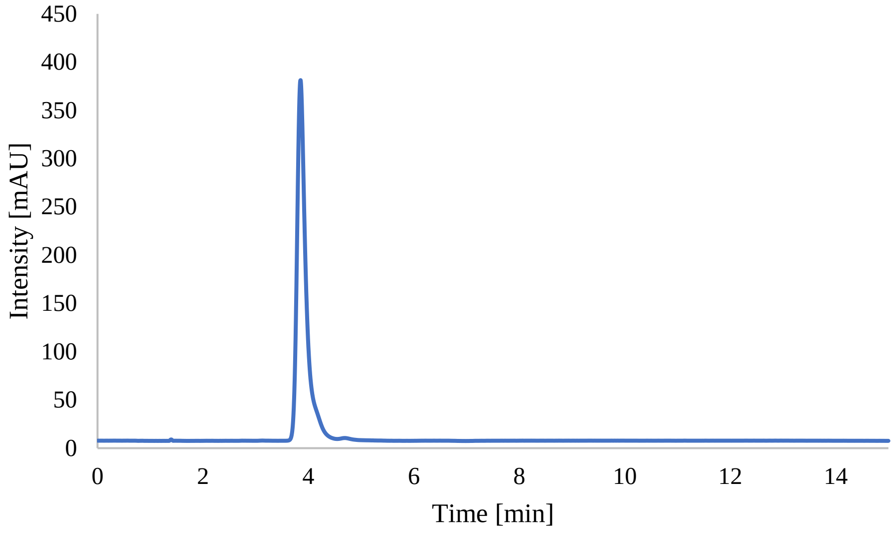

Figure S170. HPLC chromatogram for **3b**.

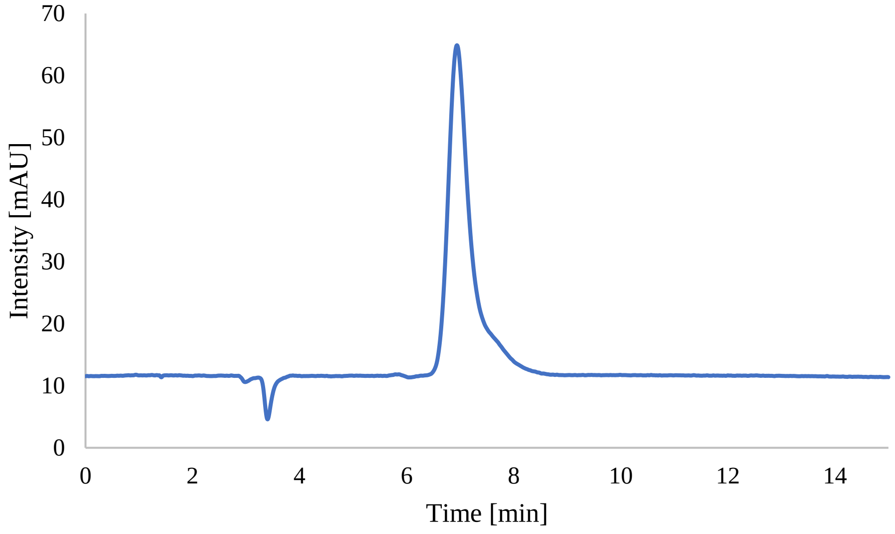

Figure S171. HPLC chromatogram for **4a**.

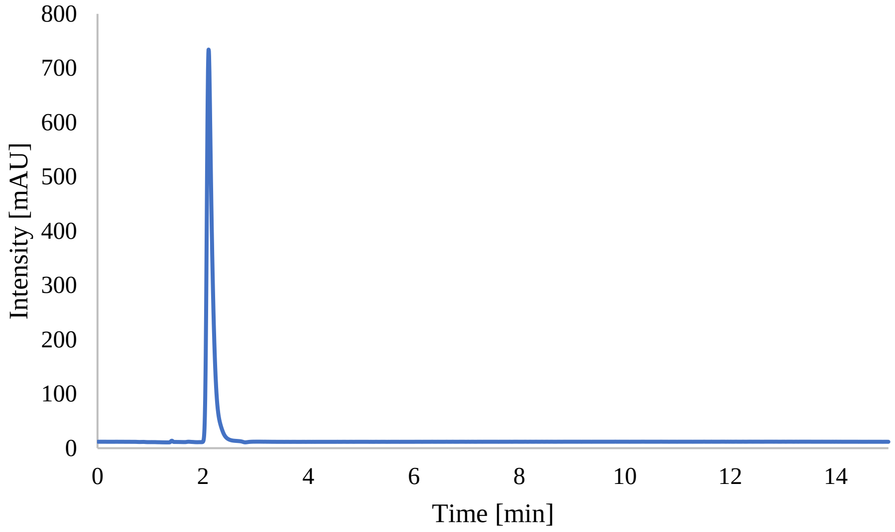

Figure S172. HPLC chromatogram for **4b**.

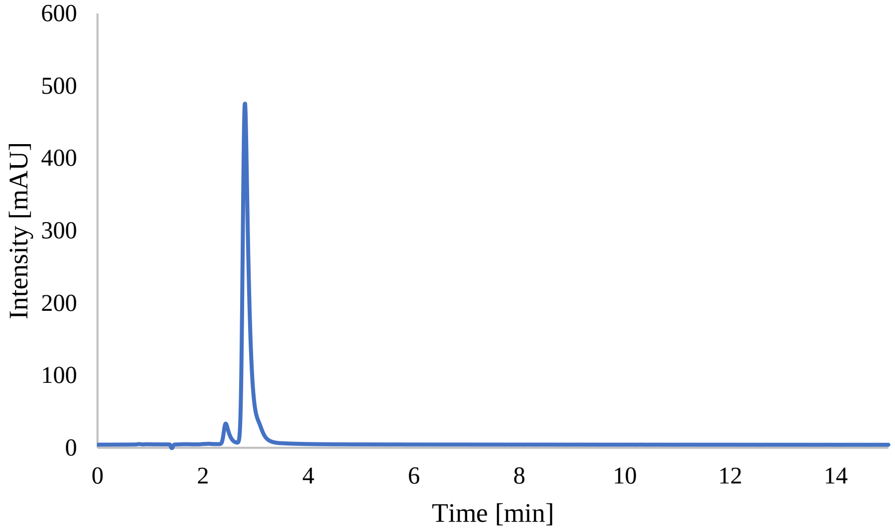

Figure S173. HPLC chromatogram for **5a**.

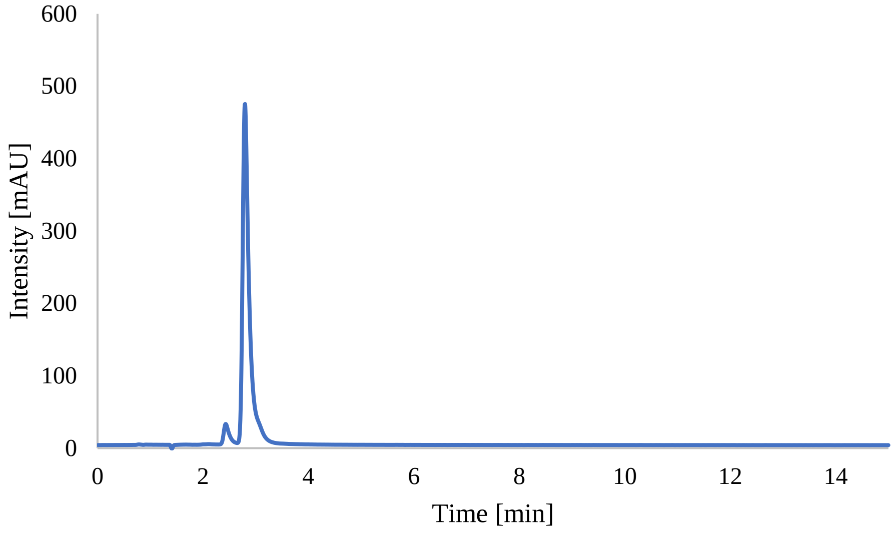

Figure S174. HPLC chromatogram for **5b**.

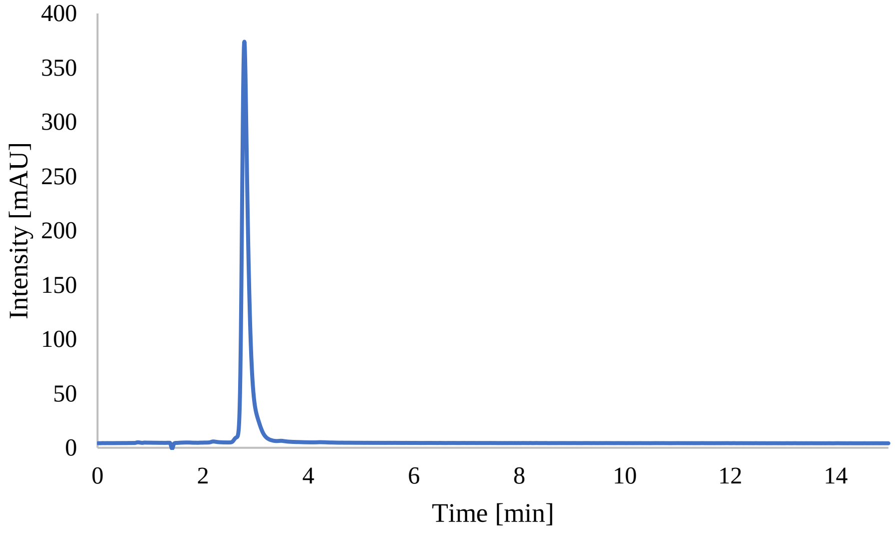

Figure S175. HPLC chromatogram for **6a**.

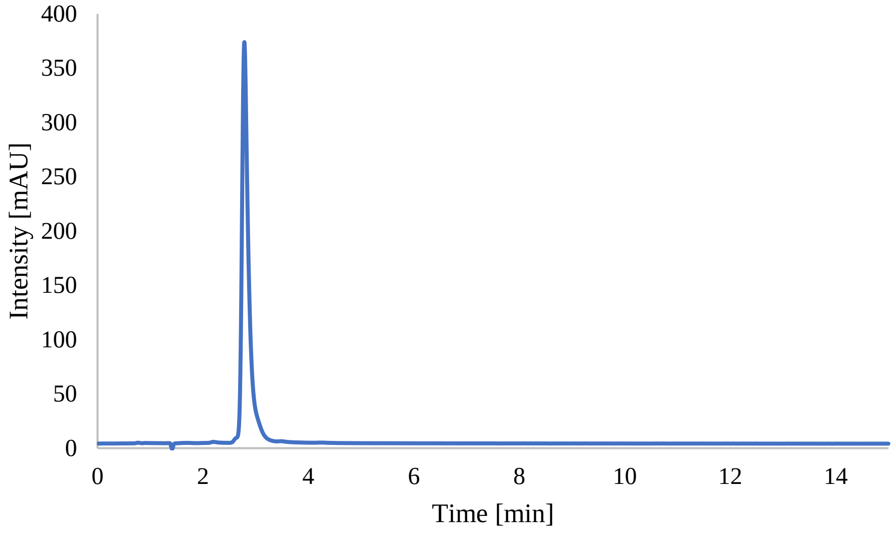

Figure S176. HPLC chromatogram for **6b**.

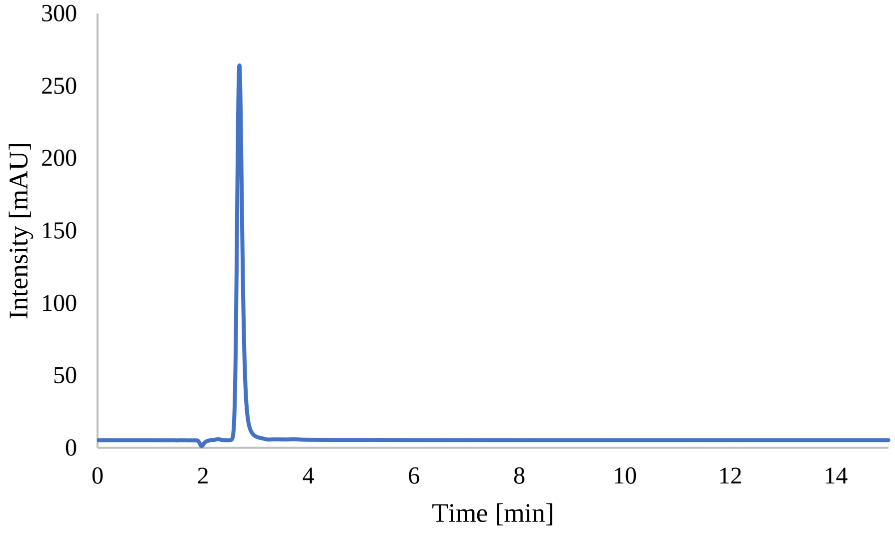

Figure S177. HPLC chromatogram for **7a**.

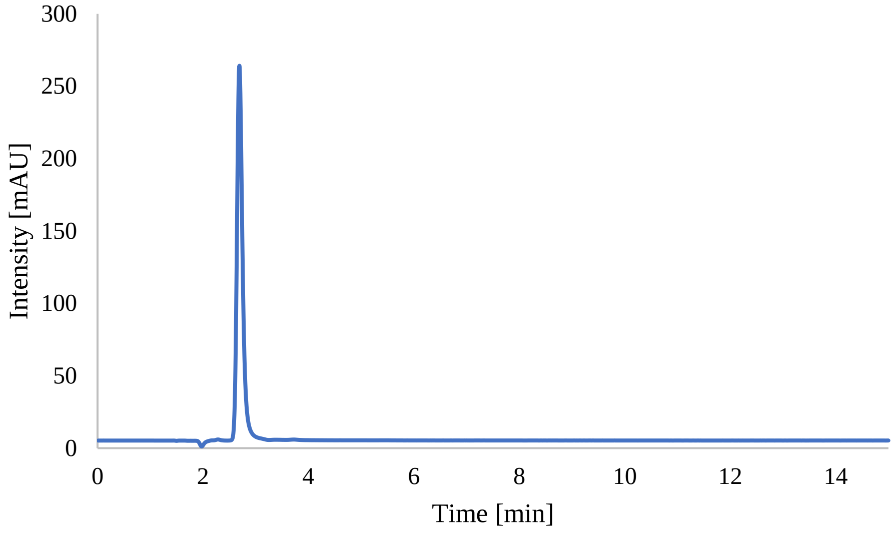

Figure S178. HPLC chromatogram for **7b**.

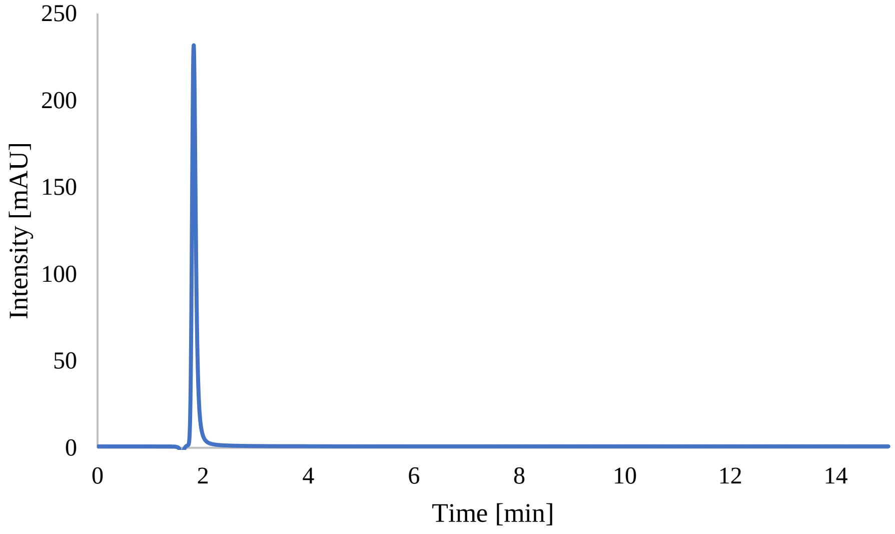

Figure S179. HPLC chromatogram for **8a**.

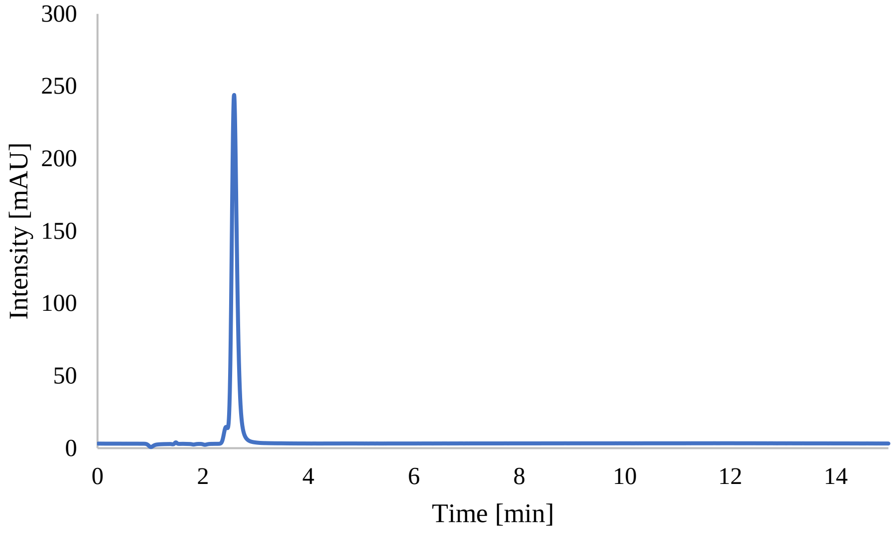

Figure S180. HPLC chromatogram for **8b**.

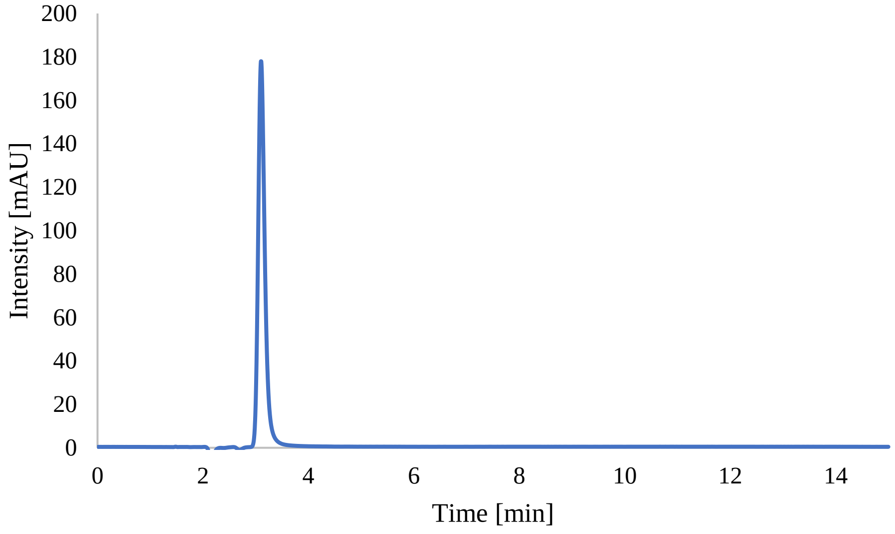

Figure S181. HPLC chromatogram for **9a**.

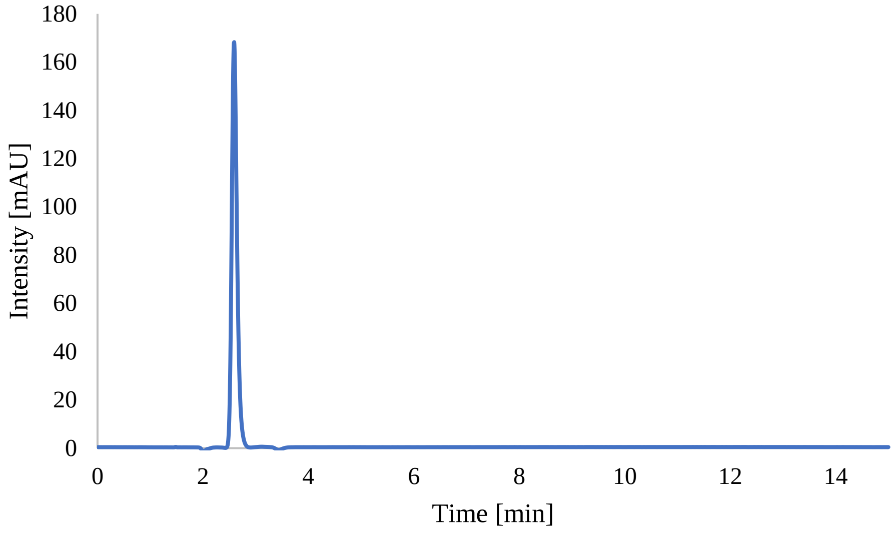

Figure S182. HPLC chromatogram for **9b**.

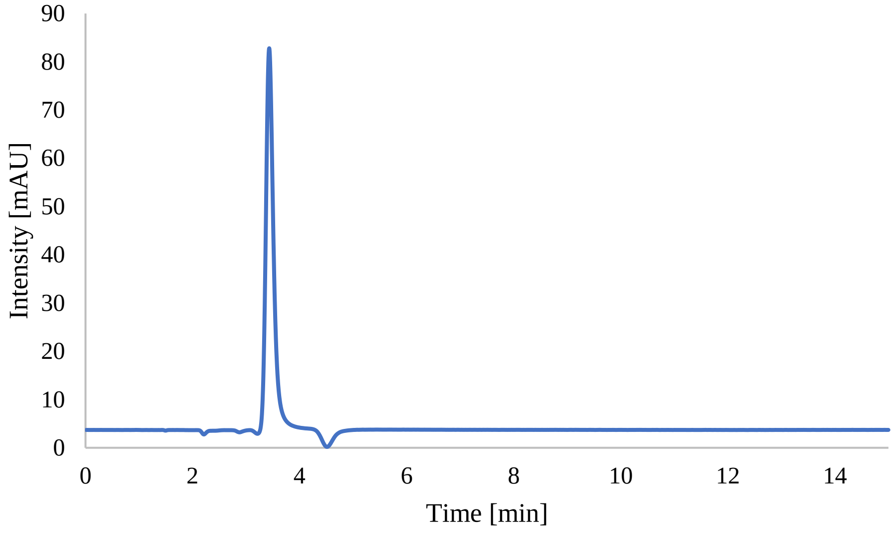

Figure S183. HPLC chromatogram for **10a**.

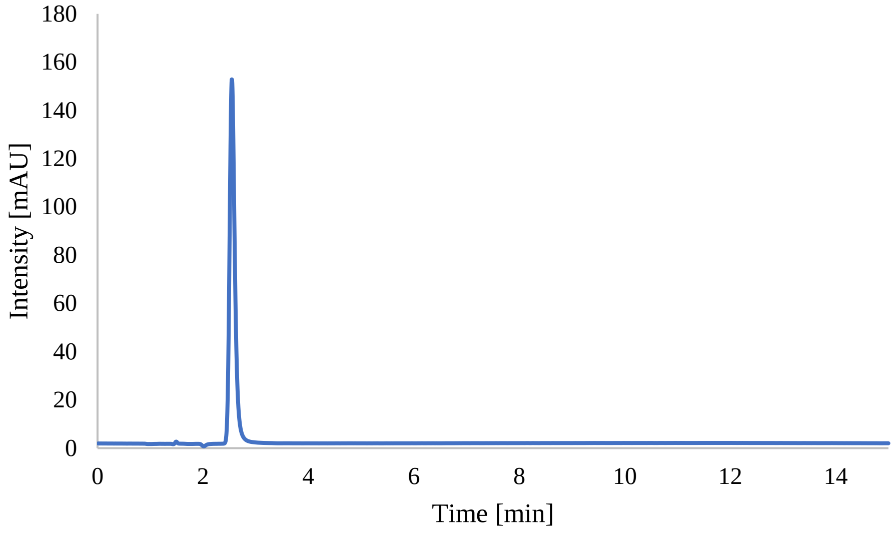

Figure S184. HPLC chromatogram for **10b**.

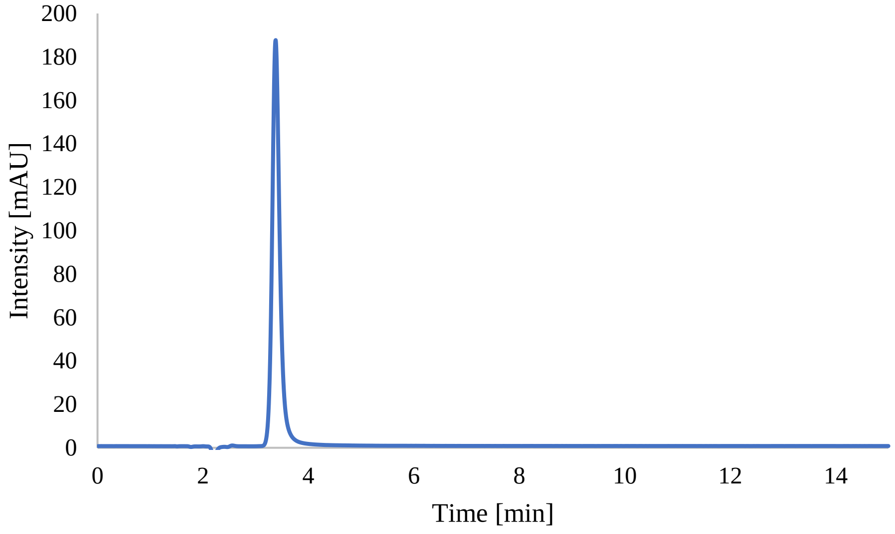

5. Biological activity of tested compounds

Figure S185. Activity charts in the 72-hour MTT test.

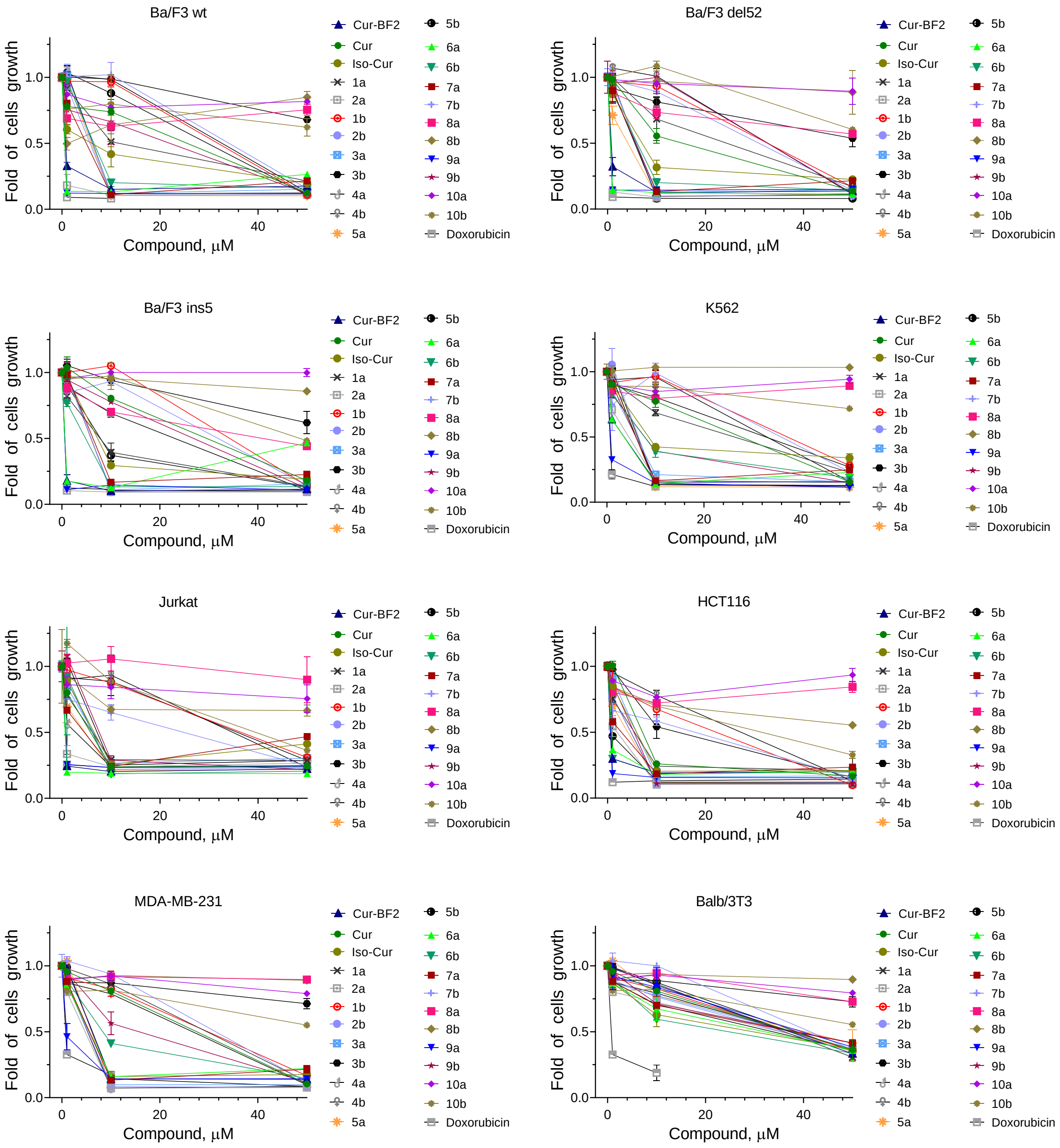

Supplement: Supplementary file 1 [file molecules-30-04609-s001.zip › molecules-3962719-supplementary.pdf]
